# Genome-Wide Identification and Characterization of Polygalacturonase Gene Family in Maize (*Zea mays* L.)

**DOI:** 10.3390/ijms221910722

**Published:** 2021-10-03

**Authors:** Lu Lu, Quancan Hou, Linlin Wang, Tianye Zhang, Wei Zhao, Tingwei Yan, Lina Zhao, Jinping Li, Xiangyuan Wan

**Affiliations:** 1 Zhongzhi International Institute of Agricultural Biosciences, Shunde Graduate School, Research Center of Biology and Agriculture, University of Science and Technology Beijing (USTB), Beijing 100024, China; g20198927@xs.ustb.edu.cn (L.L.); s20200896@xs.ustb.edu.cn (L.W.); s20200906@xs.ustb.edu.cn (T.Z.); b20190396@xs.ustb.edu.cn (W.Z.); b20190395@xs.ustb.edu.cn (T.Y.); ln_zhao@163.com (L.Z.); 2 Beijing Engineering Laboratory of Main Crop Bio-Tech Breeding, Beijing Solidwill Sci-Tech Co., Ltd., Beijing International Science and Technology Cooperation Base of Bio-Tech Breeding, Beijing 100192, China; lijinping@sjlhtech.com

**Keywords:** maize (*Zea mays* L.), polygalacturonase (PG), gene family, phylogenetic analysis, gene expression

## Abstract

Polygalacturonase (PG, EC 3.2.1.15) is a crucial enzyme for pectin degradation and is involved in various developmental processes such as fruit ripening, pollen development, cell expansion, and organ abscission. However, information on the *PG* gene family in the maize (*Zea mays* L.) genome and the specific members involved in maize anther development are still lacking. In this study, we identified 55 *PG* family genes from the maize genome and further characterized their evolutionary relationship and expression patterns. Phylogenetic analysis revealed that *ZmPGs* are grouped into six Clades, and gene structures of the same Clade are highly conserved, suggesting their functional conservation. The *ZmPGs* are randomly distributed across maize chromosomes, and collinearity analysis showed that many *ZmPGs* might be derived from tandem duplications and segmental duplications, and these genes are under purifying selection. Furthermore, gene expression analysis provided insights into possible functional divergence among *ZmPGs*. Based on the RNA-seq data analysis, we found that many *ZmPGs* are expressed in various tissues while 18 *ZmPGs* are highly expressed in maize anther, and their detailed expression profiles in different anther developmental stages were further investigated by using RT-qPCR analysis. These results provide valuable information for further functional characterization and application of the *ZmPGs* in maize.

## 1. Introduction

Polygalacturonases (PGs, EC 3.2.1.15) belong to one of the largest hydrolase families, which are polysaccharide lyases, and catalyze α-1,4 linkages among D-galacturonic acid residues in homogalacturonan [1,2]. PGs are mainly divided into three categories: endo-PGs, exo-PGs, and rhamno-PGs. Generally, rhamno-PGs present from algae to land plants, endo-PGs appear in terrestrial plants, while exo-PGs only exist in angiosperms [3]. There are four conserved domains in plant PG proteins, and the core amino acid sequences of domains I and II are SPNTDG and GDDC, respectively. The three aspartic acids (D) in domain I and domain II may be the components of the catalytic sites [4]. Domain III is composed of CGPGHG, of which the histidine residue (H) is supposed to be involved in catalytic reaction [5]. The amino acid sequence of domain IV is RIK, which may be related to ion interaction at the carboxyl end of substrates [5]. Generally, proteins that have the above four conserved domains are identified as PGs, but domain III is relatively less conservative [6]. A previous study in *Arabidopsis* revealed that more than 90% of PGs are predicted to have a signal peptide upstream of the hydrolysis domain, which suggests that most PGs are located in the apoplast [7,8]. Crystal structural differences between exo-PGs and endo-PGs determine their substrate preferences and the modes of action. The active site of an endo-PG is a surface channel, which opens up to both ends, enabling the enzyme to attack internal polysaccharides, ultimately producing oligosaccharide products varying in polymerization [9,10]. In contrast, the active site of exo-PGs is a closed pocket that only binds to the ends of pectin [11]. Rhamno-PGs can also be exo- or endo-type and catalyze the hydrolysis of galacturonic acid-rhamnose bonds in Rhamnogalacturonan I (RGI) [12].

In addition to pectinate lyase, beta-galactosidase, xylanase, and glucosidase, PG is one of the key factors for cell wall degradation [13]. These enzymes function at different processes of plant development, such as organ abscission, fruit ripening, anther dehiscence, and pollen ripening [14,15,16]. Brummell and Harpster found that PG is critical for the degradation of the primary cell wall and middle lamella [13]. In *Arabidopsis*, knock-out *PGX1* reduces hypocotyl elongation and displays higher proportions of flowers with extra petals, suggesting that PGX1 is involved in floral organ patterning [17]. The knockdown of *QUARTET2 (QRT2*) and *QRT3* impairs microspore isolation [18,19]. Several *PG* genes were reported to be involved in the intine development of pollen in Brassica (*Brassica campestris*) such as *BcMF6*, *BcMF16*, and *BcMF17* [20,21,22]. A single tomato nucleotide mutation in *PS-2* caused anther immaturity and male infertility [23]. In strawberries, silencing of the ripening-associated *FaPG1* gene reduces the breakdown of the middle layer and slows fruit softening [24]. Softening and nucleation processes of peach are also mainly controlled by endo-PGs [25].

Thus far, many *PG* genes have been identified from different plant species. The number of *PG* genes vary a lot among species. Generally, lower plants have fewer *PG* genes than higher plants. For instance, the *Physcomitrella patens* genome encodes only 11 *PG* genes, while higher plant species exploded the *PG* gene number in varying degrees (Table S1) [14,26,27,28,29,30,31,32,33,34,35,36,37,38,39]. In monocotyledonous plants, 113 and 44 *PG* genes in wheat (*Triticum aestivum*) and rice (*Oryza sativa*) have been identified, respectively [28,29]. In dicotyledonous, 66 *PG* genes were identified from the *Arabidopsis* genome, which are grouped into five different Clades. Previous studies have reported two classification types: one classified *PGs* into three or more Clades based on sequence identity, gene structure, or expression profiles [3,39,40], while the other classified *PGs* into three Clades [14,30]. Maize is one of the world’s leading crops and is of considerable value to feed, food, pharmaceutical, and other industries [41]. However, the *PG* gene family in maize has not been extensively studied, including their function in controlling anther development. Previous work in *Arabidopsis* has revealed that several *PGs* are critical for male gamete development, and we assume some maize *PGs* should also play a similar role as in *Arabidopsis* [18,42]. Identifying and characterizing *PGs* from maize will provide potential gene resources for hybrid seed production using different breeding systems such as the multi-control sterility system [43]. In this study, we identified 55 *ZmPGs* from the maize genome and performed comprehensive phylogenetic, gene structure, conserved motif and gene expression analysis. These results will contribute to a better understanding of the complexity of the *ZmPGs* and provide insights for further biological functional studies.

## 2. Results

### 2.1. Identification of PG Genes from Maize Genome

Accurate identification and a unified nomenclature are essential for future research into the *PG* gene family in maize. Here, we identified a total of 55 *PG* genes from the maize genome and named them from *ZmPG1* to *ZmPG55* according to their chromosomal locations (Table 1). The 55 *ZmPG* genes are randomly distributed across 10 chromosomes, while only one gene is scattered on a constitutive chromosome (Table 1). Fourteen genes (25.5%) are distributed on chromosome 6, which contains the largest number of *ZmPGs*, while the smallest number of *ZmPGs* appears on chromosomes 2 and 10 and Contig 206. Clade E and Clade D *ZmPG* genes are mapped to seven chromosomes, while all Clade C *ZmPG* genes are mapped to chromosome 3. *ZmPG* genes in Clade A are located on chromosomes 1, 3, 5, 6, and 9, and in Clade B, they are located on chromosomes 3, 8, and 9, whereas one Clade G gene is clustered on chromosome 2.

The length of maize PG proteins ranged from 148 to 766 amino acids and the molecular weight ranged from 15.42 kDa to 79.73 kDa with the predicted isoelectric point ranging from 4.93 to 9.22. Subcellular localization prediction showed that the 55 PG proteins are localized in seven different cellular compartments, including extracellular space, organelle membrane, chloroplast, plasma membrane, nucleus, mitochondrion, and an anchored component of the plasma membrane. Notably, 37 out of 55 maize PG proteins are localized at extracellular space, followed by 9 predicted to be localized at the plasma membrane. Interestingly, semi-autonomous organelles mitochondria and chloroplasts each have one PG protein (Table 1).

### 2.2. Phylogenetic Analysis of ZmPGs

**Figure 1:**
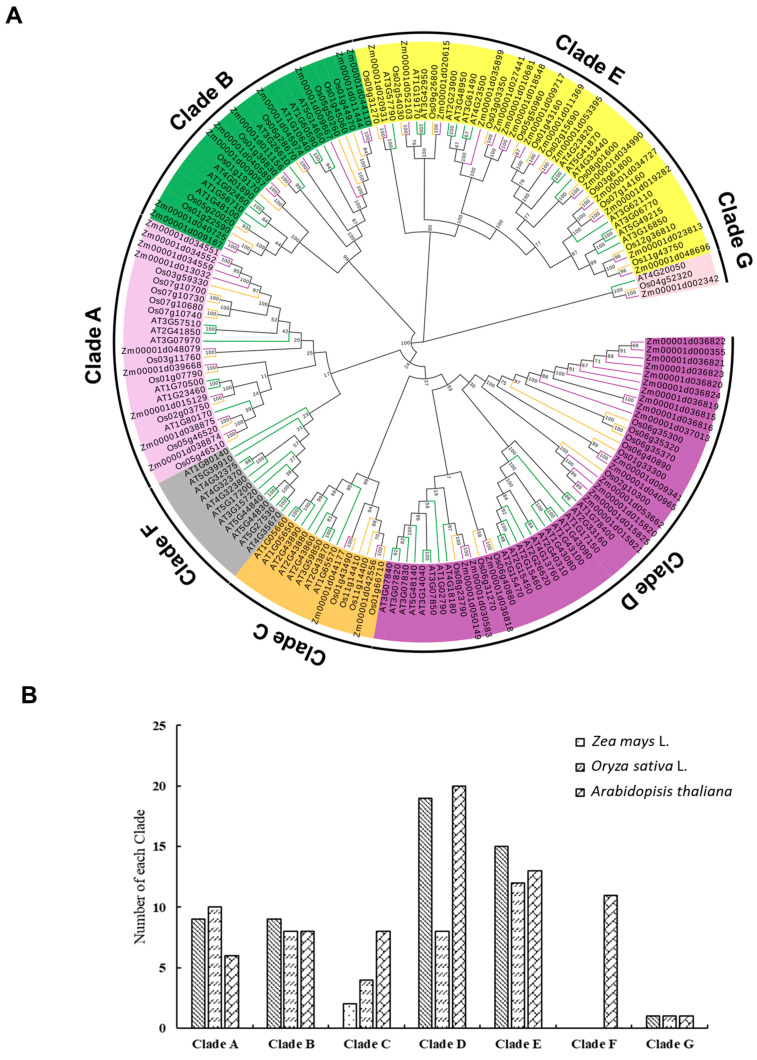
Phylogenetic analysis of *PG* genes from *Arabidopsis thaliana*, rice, and maize. (**A**) An unrooted phylogenetic tree was constructed by using full-length protein sequences. The different color shades are used to distinguish different branches, and Clades A–G indicate the *PG* gene family classifications. (**B**) *PG* gene numbers of each Clade in *Arabidopsis thaliana*, rice, and maize.

The molecular evolution of the *PG* family is decided mainly by the evolution of increasingly sophisticated organs in plants [30,44]. To investigate the phylogenetic relationship of the *PG* gene family in maize, an unrooted phylogenetic tree was constructed from the alignment of full-length PG proteins of maize, *Arabidopsis thaliana*, and rice (Figure 1A, File S1). The results showed that 165 *PGs* are grouped into seven Clades and correspondingly named Clades A to G based on a previous study [3]. Clade A and Clade B each contain 9 *ZmPGs*, and Clade C to Clade E contain 2, 19, and 15 *ZmPGs*, respectively (Figure 1B, Table S2). The gene numbers of the three species in Clade B and Clade G are roughly the same, and *PG* genes of the three species are evenly distributed in each branch. The numbers of maize and *Arabidopsis PG* genes in Clade D are approximately twice those of rice, suggesting that the duplication of these Clade *PG* genes in maize and *Arabidopsis* occurred after specification. Interestingly, *Arabidopsis* has eleven members in Clade F, while maize and rice are absent from this Clade, indicating that these genes probably emerged after monocot and dicot plant separation. In contrast, *Arabidopsis* has fewer *PG* members in Clade A than maize and rice do. It is speculated that these monocot-specific or dicot-proliferated *PG* genes are probably functionalized for monocots and dicots development during evolution (Figure 1B).

**Figure 2:**
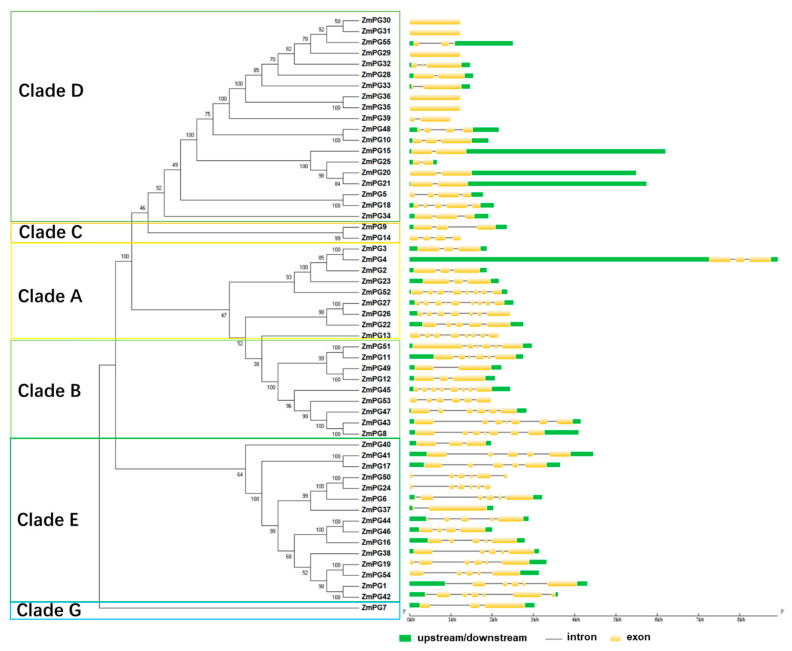
Phylogenetic and gene structure analysis of *ZmPG* genes. The left panel shows the phylogeny of *ZmPG* genes. The right panel illustrates the intron/exon configurations of the corresponding *ZmPG* genes.

An unrooted phylogenetic tree was reconstructed using full-length protein sequences of the 55 ZmPGs with the neighbor-joining (NJ) method. The tree was divided into six main Clades (Clades A to G; Clade F was not included herein and hereafter, as maize does not have a Clade F member) (Figure 2). To explore the relationship of gene structure and phylogeny, we investigated gene structures by GSDS [45]. The results showed that there are a different number of exons in *ZmPG* genes (Figure 2), ranging from 1 to 9. *ZmPGs* in Clades C and D containing exo-PGs have shorter gene sequences and fewer exons, while the *ZmPGs* in Clade E containing oligo-PGs generally have longer intron sequences. Consistent with the phylogenetic relationship, closely related genes usually have common gene structures and intron lengths. However, some *ZmPGs* in Clade B show a significant difference in gene structural arrangements. For instance, *ZmPG49* contains only two exons, while *ZmPG45* contains 8 exons (Figure 2).

### 2.3. Collinearity and Amino Acid Substitution Selection Pressure Analyses

Segmental duplications are long DNA fragments that are nearly identical and present in distant chromosome locations. They occur most frequently in plants because most plants are diploidized polyploids and retain a great deal of duplicated chromosomal blocks within their genomes [46,47]. Tandem duplications mainly occur in the region of chromosome recombination [48]. Gene family members generated from tandem replications are usually closely arranged on the same chromosome, forming a gene cluster with similar sequences and similar functions [49].

**Figure 3:**
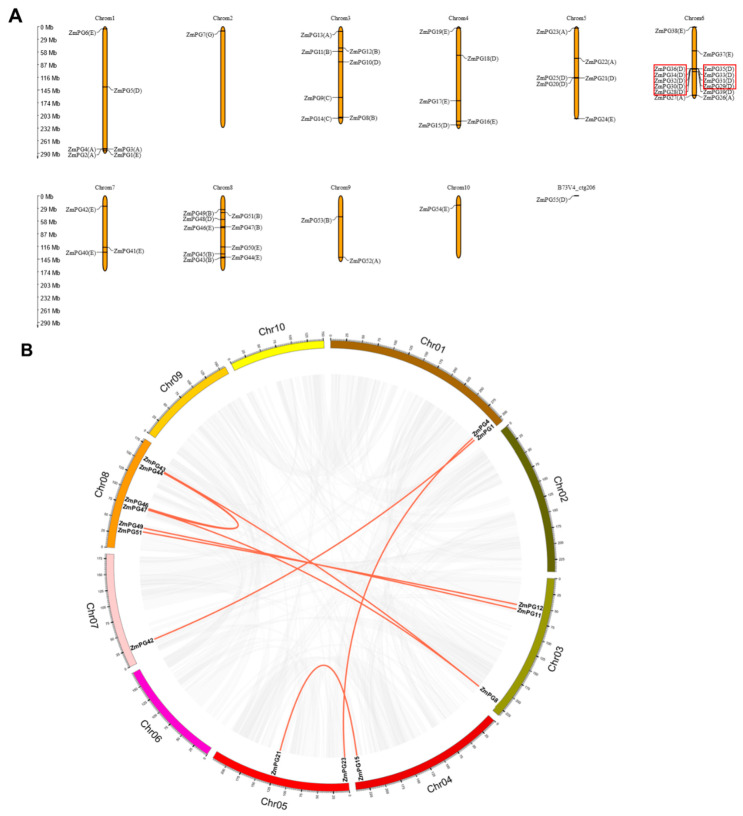
Chromosomal location and synteny analysis of *ZmPGs* in maize genome. (**A**) Chromosomal locations of *ZmPGs*. Tandem-duplicated genes are indicated with red boxes, the chromosome number is indicated above each chromosome. The scale is in megabases (Mb). (**B**) Syntenic relationship of *ZmPGs*. The annotations on the fragments represent different chromosomes, and the numbers in the outermost circle represent the positions on the corresponding chromosomes. The *ZmPGs* involved in segmental duplications in the *ZmPG* gene family are mapped to their respective locations of the maize genome in the circular diagram. The red lines represent the segmental duplication pairs between the *ZmPGs* and the gray lines represent the segmental duplication pairs in the whole maize genome.

Segmental duplications and tandem duplications of the 55 *ZmPGs* were investigated by MEGAX and McscanX. Our analysis revealed 24 tandem duplication pairs in *ZmPGs* (Table 2). These tandem duplication pairs formed from 10 genes, which are located on chromosome 6 and belong to Clade D. In addition to the tandem duplication pairs, 9 segmental duplication pairs were identified (Figure 3B, Table 2). A total of 13 *ZmPGs* with 9 pairs associated with segmental duplications account for 23.63% (13/55) of all the *ZmPGs*, and 9 *ZmPGs* with 24 pairs associated with tandem duplications account for 16.36% (9/55) of all the *ZmPGs.* The total duplication ratio of *ZmPGs* is 39.99%, which is much lower than the maize genome duplication ratio (25,000 Mb, 60–80%), suggesting that segmental and tandem duplications contribute little to the expansion of the *ZmPG* gene family.

Ks (synonymous substitution rate) and Ka (nonsynonymous substitution rate) parameters of duplication events were calculated through KaKs Calculator, and the date of the duplication events were calculated (T) using the formula T = Ks/2λ (λ represents the estimated clock-like rate of synonymous substitution, which is 1.65 × 10^−8^ substitutions/synonymous site/year for cereals) [50,51,52]. Codon alignment of duplicated genes was performed by MEGAX [53]. The approximate dates of the estimated duplication events are shown in Table 2. The origin of the 24 tandem duplication pairs of *ZmPGs* on the same chromosome was 0.521 to 18.424 million years ago. The dates of other segmental duplication pairs were 4.151 to 56.732 million years ago. In addition, the Ka/Ks ratios of the 24 pairs of *ZmPG* tandem duplications are less than 1, and the Ka/Ks ratios of most *ZmPG* segmental duplications are also less than 1. As the Ka/Ks ratio gives an indication of what selection has been placed on this gene, these results indicate that the duplicated maize genes are under purifying selection, and this selection would eliminate deleterious mutations in the species.

### 2.4. Expression Analysis of Maize PG Genes in Different Tissues and Developmental Anthers

**Figure 4:**
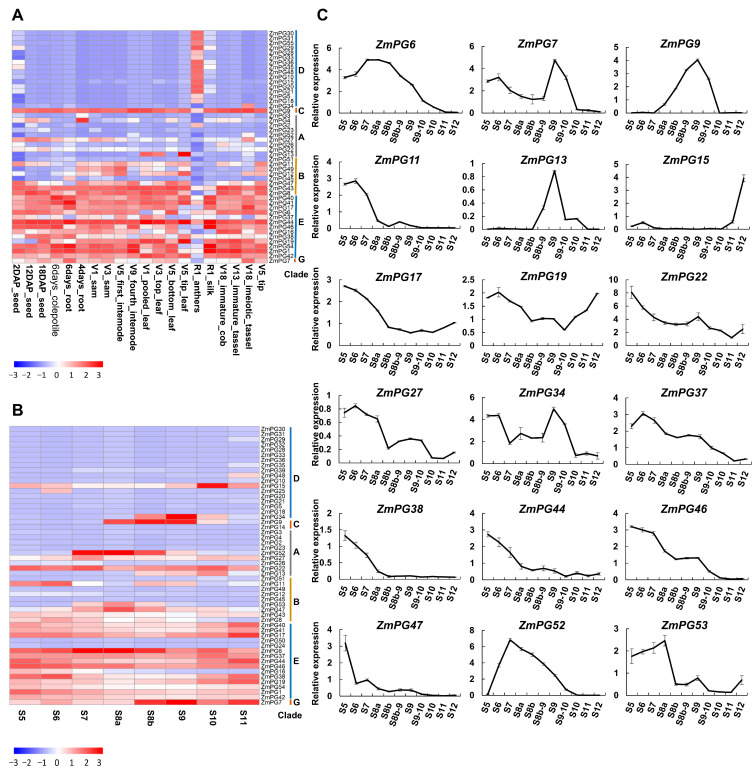
Expression profiles of *ZmPGs*. (**A**) Hierarchical clustering and heat map showing the expression levels of *ZmPGs* from 20 maize tissues. The vertical bar on the right illustrates the six groups of *ZmPGs*. (**B**) Hierarchical clustering and heat map showing the expression levels of *ZmPGs* at eight stages of anther development by transcriptome analysis. The vertical bar on the right illustrates the six groups of *ZmPGs*. The color scale bars represent the relative expression level of (**A**,**B**). (**C**) Relative expression of 18 *ZmPGs* from developmental anther stages 5 to 12 (S5–S12) detected by RT-qPCR. Each bar represents the mean and SD of three repeats. Similar results were obtained from three independent biological experiments.

To investigate the expression patterns of *ZmPGs*, RNA-seq data of 20 maize tissues were retrieved from the SRA (Sequence Read Archive) database, and RNA-seq analysis was further performed to obtain FPKM values of *ZmPGs* (Table S3). The results showed that 48 *ZmPG* genes were expressed in at least one tissue and the expression levels of the 48 genes were represented by a heatmap, as shown in Figure 4. However, there are 7 genes (*ZmPG14, ZmPG24, ZmPG25, ZmPG32, ZmPG39, ZmPG50,* and *ZmPG53*) that had no detectable expression or low expression (FPKM less than 1 in both tissues) were filtered out. As shown in Figure 4A, *ZmPG* genes in Clade E were constitutively expressed in various tissues, while *ZmPG* genes in Clade D were specifically expressed in anther, and *ZmPG34* was specifically expressed in the meiotic tassel. Clade C has only two members, *ZmPG9* was constitutively expressed at high levels, but the expression of the other member *ZmPG14* could not be detected in any of the analyzed tissues, indicating their variations in *cis*-regulation. The orphan gene *ZmPG7* of Clade G was highly expressed in most analyzed tissues, including the meiotic tassel.

As many *ZmPG* genes are specifically expressed in anther, we further analyzed the expression profiles in developmental anthers. According to the cytological characteristics of maize anthers, the development of maize anthers can be divided into 14 stages, the meiosis starts from stage 7 and ends at stage 8b, and the microspore undergoes its first mitosis at stage 11 [54]. The RNA_seq data from S5 to S11 were used for the analysis (Figure 4B, Table S4). Consistent with the constitutive expression pattern in different tissues, most Clade E *ZmPGs* were also constitutively expressed at all the analyzed anther developmental stages. Several anther-specific Clade D *ZmPG* genes peaked their expression at specific anther developmental stages: *ZmPG34* (S8b–S10), *ZmPG9* (S8a–S10), *ZmPG52* (S7–S9), *ZmPG13* (S8b–S10), and *ZmPG53* (S7–S8b).

### 2.5. Validation of the PG Gene Expression in Developmental Anthers via RT-qPCR

To further investigate the *ZmPGs* involved in anther development, 18 *ZmPGs* that showed expression in developmental anthers according to RNA_seq were validated via RT-qPCR. Consistent with the RNA_seq data, all the 18 genes were expressed in anthers (Figure 4C). According to their expression peak occurrences at early (S5–S6), middle (S7–S9), and late (S10–S12) stages, these genes can be divided into four groups. Eleven of the 18 analyzed genes, *ZmPG6*, *ZmPG11, ZmPG17, ZmPG22, ZmPG27, ZmPG37, ZmPG38, ZmPG44, ZmPG46, ZmPG47*, and *ZmPG53,* showed high expression at early stages in anther development, while *ZmPG15* was highly expressed at late stages. The expression of *ZmPG7*, *ZmPG9, ZmPG13, ZmPG34,* and *ZmPG52* peaked at the middle stages (S7–S9) of anther development. *ZmPG19* expressed highly at both early and late stages but not at the middle stage.

### 2.6. Cis-Regulatory Motif Analysis of ZmPG Genes

**Figure 5:**
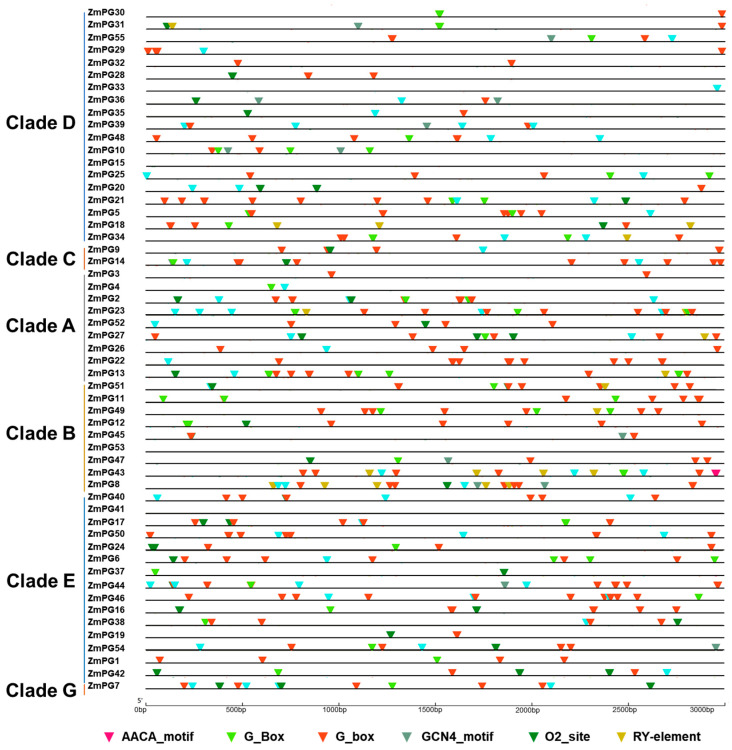
Schematic model of seven *cis*-elements in the promoter sequences of *ZmPGs*. AACA_motif is associated with endosperm-specific negative expression, the G-box/G-Box *cis*-acting regulatory element is involved in light responsiveness, GCN4_motif is involved in endosperm expression, MBS represents a MYB binding site, O2-site represents a *cis*-acting regulatory element, which is involved in zein metabolism and regulation, and RY-element is involved in seed-specific regulation.

The expression analysis above showed variations in expression patterns of the maize *PG* genes. As *cis*-elements are important in gene expression regulation, we analyzed the *cis*-elements in the *ZmPG* promoters by using Plant CARE [55] (Figure 5). Generally, two categories can be distinguished according to their expression from life beginning to end: one is that the proteins are encoded by the early genes, mainly including cytoskeletal proteins, as well as proteins related to cell wall synthesis, starch accumulation, the other Clades including late genes, which encode proteins involved in pollen tube growth and pollen maturation [56]. In *Arabidopsis*, MYB transcription factor MS188 directly regulates *QRT3* to affect pectin wall degradation and pollen exine synthesis [45]. It is suggested that the *MS188* homologous gene *ZmMYB84* may regulate *PGs* in maize. Indeed, as we found that 35 *ZmPG* promoter regions have MBS (MYB binding site CAACTG_motif). There are 35 *ZmPG* genes containing (GCN4_motif) and one gene containing (AACA_motif) that are involved in endosperm expression. In addition, we also identified 32 (GA(T)TGA(T)C(T)A(G)TGG(A)_motif) and 11 (CACGTT_motif) *cis*-acting regulatory elements involved in zein metabolism (O2-site) and seed-specific regulation (RY-element), respectively. In maize *PG* gene promoters, *cis*-acting regulatory elements involved in light responsiveness (G-box/G-Box) were identified in 52 *PG* genes. These results indicated that *ZmPG* genes might be involved in different biological processes of plant development.

### 2.7. Conserved Motif and Structure Prediction of the Maize PG Proteins

**Figure 6:**
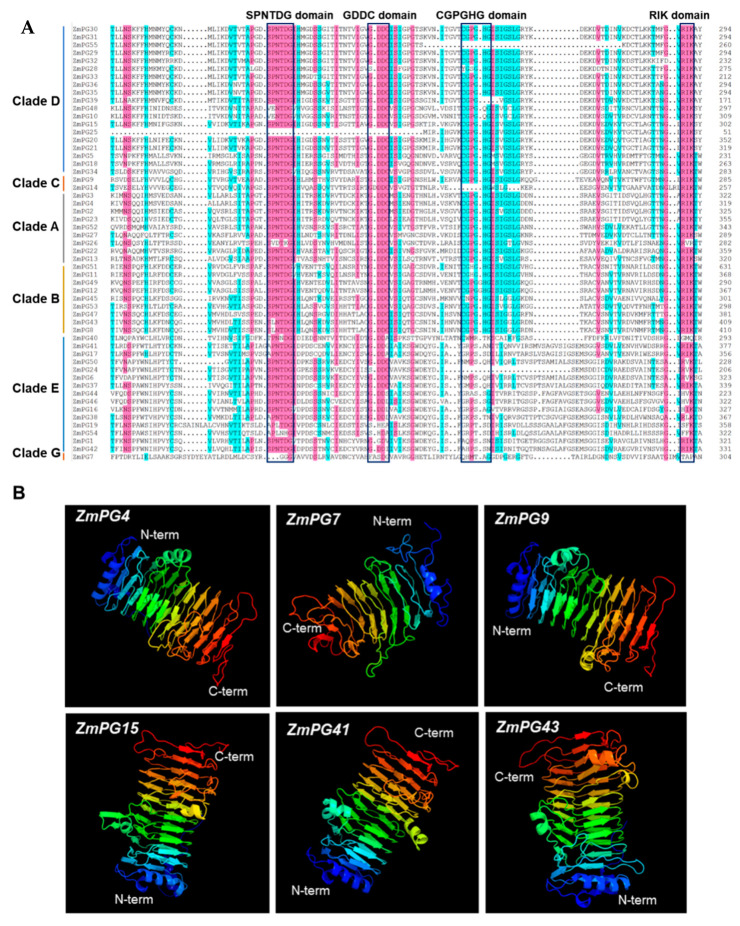
Conserved domain and structural analysis of ZmPGs. (**A**) Amino acid sequences alignment of four conserved domains in ZmPGs. The conserved sequences of the four conserved domains are shown at the top. The annotations on the left indicate the different classifications of PGs. (**B**) Three-dimensional structures of six representative ZmPG proteins.

Amino acid sequence alignment indicated that the vast majority of ZmPGs contain four conserved domains (I: SPNTDGI, II: GDDC, III: CGPGHGISIGSLG, and IV: RIK) (Figure 6A). However, not all ZmPGs have all the four conserved domains, e.g., in Clade D, ZmPG25 lacks domains I and II, while ZmPG55 lacks domain III. All PG proteins of Clade E lack the conserved III domain, which is also the case in apple [36]. In addition, individual amino acid substitutions are found in the conserved domains. For instance, the serine (S) of the conserved domain I is substituted by alanine (A) or threonine (T) in many Clade E PGs. Overall, the Clade E ZmPGs are the most variable members of the family. It is worth noting that the Clade G member ZmPG7 does not contain any of the four conserved domains. We then used MEME to scan conserved motifs in ZmPG proteins. Ten conserved motifs are identified (Figure S1) and none of the ZmPGs contain all 10 motifs. Genes in the same group tend to share common motifs. For example, the PGs in Clade D contain eight same motifs, except for ZmPG20. Motif 5 covers conserved domain I and a portion of conserved domain II, and motif 10 covers conserved domain III and domain IV. Besides these two conserved motifs, motif 8 and motif 4 are the most conservative that present in the majority of PGs, including the Clade E PGs. Next, we predicted three-dimensional structures of ZmPG proteins. The results showed that ZmPGs are structurally conserved and have a single-stranded right-handed beta-helix structure (Figure 6B and Figure S2), also known as a pectin lyase-like CATH superfamily 5 [54,57]. This superfamily is mostly found in bacteria, plants, and fungus, and scarcely on invertebrates and environmental samples. This is consistent with the structure of polygalacturonase from *Erwinia carotovora* and *Aspergillus niger* [58,59]. Parallel β-helically folded enzymes can recognize and hydrolyze large polysaccharides [60]. Consistent with structure conservation, the GO annotations showed that the functions of ZmPGs are highly conserved. Almost all genes are involved in the process of pectin catabolism in the biological process and associated with the cell wall in the cellular component (Table S5). All ZmPG proteins may have hydrolyase and polygalacturonase activity (Figure S3).

## 3. Discussion

Plant *PG* genes were first identified more than 50 years ago, and the gene products are multifunctional proteins that play an important role in the decomposition of pectin. Previous studies have genome-wide identified *PG* gene family members from several plant species. In the current study, we identified 55 *ZmPG* genes, and they are randomly distributed on 11 chromosomes. Subcellular localization prediction analysis indicated that the 55 ZmPG proteins are localized in different cellular compartments. Most of them are predicted to be localized at the extracellular space, suggesting that they are secretory proteins and are associated with the degradation of the cell wall.

Phylogenetic analysis revealed that the 55 maize *PG* genes are grouped into six Clades, and members in the same group have similar gene structures. The number of *ZmPG* genes in Clade B is approximately the same in the three species, and they are evenly distributed in various branches of the phylogenetic tree, suggesting that duplication of the *PG* genes in Clade B may have occurred before monocots and dicots separation. Meanwhile, multiple *PG* genes in Clade D are clustered together in the same species, indicating that duplication of these genes occurred after specification.

Homologous genes distributed on farther locations are usually referred to as segmental duplication events, while those located together are considered as tandem duplication events [51]. Our analysis showed that the total segmental and tandem duplication ratio (39.99%) is much lower than the maize whole-genome duplication ratio. Therefore, tandem and segmental duplication events have little effect on *ZmPGs* expansion. However, tandem duplication-generated *ZmPGs* are mostly presented in Clade D, which is similar to other species [30,39], suggesting that a biased expansion occurred in Clade D genes. Our analysis showed that the KaKs ratio of 6 pairs of segmental duplications and 24 pairs of tandem duplications of *ZmPG* genes are less than 1. This indicates that the duplication of the *ZmPG* genes occurred through purifying selection, and the corresponding ZmPG proteins are considered to be relatively conserved [50,51,52,53,55,61]. Thus far, the origin of maize has not been extensively studied. It is not clear whether duplication events of the *PG* gene family predated the formation of Maize Species. However, the predicted earliest dates of duplication events in the 9 maize *PG* gene segmental duplication pairs ranged from 4.151 to 56.732 million years ago, and 24 tandem duplication pairs ranged from 0.521 to 18.424 million years ago. These results suggest that this is an ancient gene family.

Generally, PG proteins contain four conserved domains except for the Clade E members, which are less conserved [36]. Consistent with the previous studies, all ZmPGs in Clade E lack the conserved domain III. Clade G is a special category because it does not have any of the four typical domains of ZmPGs, but only has two conserved motifs. However, Clade E and G PGs are widely found in different organisms [3]. Therefore, they may have undergone extensive natural selection during the long evolutionary process. ZmPGs of each Clade may have their specific biochemical activity. It is speculated that Clade A and Clade B contain endo-PGs, Clade C and Clade D contain exo-PGs, Clade E contains rhamno PGs, and Clade F cannot be defined as exo-PGs or endo-PGs [3]. Different types of PGs have different substrates (such as HG (homogalacturonan, HG) and RG (Homogalacturonan, HG)) and products (such as OGS (oligogalacturonides, OGs) and RHA (rhamno-polygalacturonase, RHA)) [3]. Therefore, the evolutionary differences among Clades may indicate the variations in pectin components that they catalyze. Structural models may contribute to understanding the evolutionary history and biological function of ZmPGs. Important aspects to explore the structural study further are to disclose their catalytic active sites, as well as to characterize their substrates and kinetic properties.

Gene expression patterns are significant clues for clarifying gene function. In this study, we analyzed expression profiles of the 55 *ZmPG* genes. Previous studies showed that *PG* genes in Clade E present from algae to flowering plants, while the *PGs* in Clades C, D, and F present only in flowering plants [3]. Coding sequences of Clade E *PG* genes are highly conserved and most of the Clade E *PG* genes tend to be constitutively expressed, which reflect their important roles in plant development. These characteristics are similar to those of housekeeping genes (HK) in humans and mice [62]. Many HK genes have early origins, and the slower evolution rate of these very early originated ancient proteins is a typical feature [63]. These results suggest that members of Clade E are probably ancient proteins, whereas members of Clade C, Clade D, and Clade F may be critical for the development of specific organs in flowering plants. Most *ZmPGs* in Clade D are highly and specifically expressed in anthers, as well as in *Arabidopsis*, poplar, and cucumber [14,30,39], indicating the functional conservation of *PG* genes in male reproductive development across species.

Pectin is the main component of the pollen wall in angiosperms, and the pollen tube wall is an extension of the pollen inner wall [64]. In addition to the presence of pectin, many pectin-degrading enzymes have been found in plant anthers, including pectinase, polymethylgalacturonase, and pectin methylesterase [65,66,67]. Previous studies have shown that the expression of *PGs* is higher in the late stage of plant anther development [65,68,69,70]. During pollen development, meiosis-generated tetrads require a separation event to form independent microspores. In *Arabidopsis*, microspores fail to separate in *qrt1-* and *qrt2*-deficient mutants, where callose can be degraded normally during tetrad pollen formation. However, pectin still exists after the degradation of callose, thereby demonstrating that QRT1 and QRT2 are required for the pectin degradation during microspore separation [19]. In our study, we showed that *ZmPG52(Zm00001d048079)* shares high homology with *Arabidopsis QRT2(At3g07970)* and is highly expressed in developmental anthers, suggesting that *ZmPG52* could also be involved in maize tetrad separation. Polygalacturonase is required for the degradation of the cell wall of the pollen mother cell. In *Arabidopsis thaliana*, microspore isolation is impaired by the knocking out of *QUARTET2* (*QRT2*) and *QRT3* [18,19]. *ZmPG7* (*Zm00001d002342*) shares high homology with *QRT3*, RNA-seq and RT-qPCR indicated that its expression peaks at the beginning of stage 7 of meiosis, suggesting that *ZmPG7* may play the same role as its orthologs in *Arabidopsis*. Studies have shown that *PGA4* is involved in the pollen development process and pollen tube growth [71]. *ZmPG34* (*Zm00001d006818*) shares high homology with *PGA4* and is highly expressed at the trinucleate stage in the fertile anthers, so it is speculated that *ZmPG34* may also be involved in pollen tube growth in the same manner [28]. Notably, *ZmPG7*, *ZmPG34*, and *ZmPG52* promoters contain MBS. In *Arabidopsis*, MYB transcription factor MS188 directly regulates *QRT3* to affect pectin wall degradation and pollen exine synthesis [45]. It is suggested that MYB84 may regulate *PGs* in maize. Further validation of their functions will greatly advance our understanding of male sterility in maize.

## 4. Materials and Methods

### 4.1. Genome-Wide Identification of PG Genes in Maize

Two methods and a four-step analysis were conducted to identify *PG* genes from the maize genome. First, *Arabidopsis* PG protein sequences obtained from the TAIR website (https://www.arabidopsis.org/, accessed on 1 April 2020) were used as probes to search in the maize genome with blastP on the Gramene website (http://ensembl.gramene.org/Zea_mays/Info/Index, accessed on 4 April 2020) [1]. Second, the maize genome was scanned and predicted for proteins corresponding to the Pfam *PG* family (PF00295) using Hmmer V3 (http://pfam.xfam.org/, accessed on 11 April 2020) [72]. Candidates were obtained from the original PG HMM, the high-quality proteome (E value < 1·e^−10^) was aligned with the manual verification of the complete PG domain, and hmmbuild was used to construct the maize-specific PG HMM. Putative *PG* genes were selected from maize-specific HMM results with E-values below 0.01. Genes acquired from the two above methods were taken as maize candidate *PG* genes. Then, the isolated candidate *PG* genes were further confirmed via online tools Pfam (http://pfam.sanger.ac.uk/search, accessed on 20 April 2020) and SMART (http://smart.embl-heidelberg.de/, accessed on 20 April 2020). In addition to the *PG* genes obtained by using the methods above, an *Arabidopsis PG* gene At4g20050 had been investigated and was used to search for orthologs in the maize database, although it did not contain any domain of classic PG proteins [18]. After deduplication, the genes left were considered as maize *PG* genes. To determine the physical and chemical parameters of each maize PG protein, ExPASY (https://web.expasy.org/protparam/, accessed on 1 May 2020) was used to calculate molecular weight (MW), isoelectric point (PI), and the number of amino acids [73]. BUSCA was used to predict protein subcellular localization (http://busca.biocomp.unibo.it/, accessed on 1 May 2020) [74].

### 4.2. Phylogenetic Analysis and Chromosomal Location of Maize PG Genes

Multiple alignments of maize, *Arabidopsis*, and rice PG protein sequences were performed by ClustalW of MEGAX [75]. Phylogenetic trees were constructed with MEGAX using the neighbor-joining (NJ) method, and bootstrap values were based on 500 replicates. Information of chromosome length and chromosomal location of maize *PG* genes were obtained from the Ensemble the Plants online website (http://ensembl.gramene.org/Zea_mays/Info/Index, accessed on 2 February 2021) and displayed by using the online software MA2C (http://mg2c.iask.in/mg2c_v2.0/, accessed on 3 February 2021).

### 4.3. Gene Structure and Conserved Motif Analysis

Sequence and chromosome annotation information of maize *PG* genes were obtained from Gramene website (http://ensembl.gramene.org/Zea_mays/Info/Index, accessed on 3 April 2021). The web-based bioinformatic tool GSDS2.0 (http://gsds.cbi.pku.edu.cn/index.php) was used to graphically display the exon/intron genomic structures of maize *PG* genes [48]. An online tool MEME motif analysis (http://meme-suite.org/tools/meme, accessed on 3 April 2021) was carried out to identify the conserved motifs of maize PG proteins [76]. The maximum number of patterns determined in the MEME program was adjusted to 10 and the width of the domain was set from 6 to 100. Default parameters were used for these bioinformatic tools, unless otherwise specified. DNAMAN software was used to display four PG conserved domains.

### 4.4. Collinearity Analysis and Selective Pressure for Duplicated Genes

To explore the evolutionary dynamics of the coding sequences of *ZmPGs*, algorithms for vertical and horizontal comparisons were performed. Two genes located in the same chromosomal fragment within 100 kb and separated by five or fewer genes were identified as tandem-duplicated genes [77]. MCScanX was used to analyze the segmental and tandem duplication events [78]. Circos were used to draw the sequence segmental duplication homology [79].

### 4.5. Cis-Elements Analysis of Maize PG Gene Promoters

*ZmPG* promoter sequences (3 Kb upstream the start codon) were retrieved from the Gramene database (http://bl.gramene.org/zea_mays/info/index, accessed on 23 May 2020). Online software PlantCARE (http://bioinformatics.psb.ugent.be/webtools/plantcare/html/, accessed on 18 April 2021) was used to analyze the *cis*-elements in the isolated promoter sequences. The models of *cis*-elements in the promoters were displayed with software GSDS [48].

### 4.6. Gene Model Analysis and the Functional Connection Network Analysis

The GO (Gene Ontology) analysis was performed by GENE ONTOLOGY (http://geneontology.org/, accessed on 8 February 2021). Protein structure prediction was performed based on Phyre2 (http://www.sbg.bio.ic.ac.uk/phyre2/html/page.cgi?id=index, accessed on 4 July 2020) [80].

### 4.7. Expression Analysis of Maize PG Genes

The transcriptome data of Maize at different developmental stages were obtained from the SRA (Sequence Read Archive) database at NCBI (National Center for Biotechnology Information) under the accession code PRJNA171684 [81]. Transcriptome data of seed, coleoptile, root, stem, intemode, leaf, anthers, silk, cob, tassel, and tips were analyzed. First, the high-throughput sequencing data were converted into fastq files using the fast-dump parameter of the sratoolkit software, and the raw sequencing data were assessed for quality using FastQC software, followed by adaptor removing [82]. Low-quality and the excessive number of unknown bases were filtered using Trimmomatic software to obtain clean reads. Clean reads were aligned to the maize B73 reference genome using Hisat2 software [83]. Maize reference genome sequence and annotation information were downloaded from the Ensembl database (ftp://ftp.ensemblgenomes.orgpub/plants/release-27/GenBank/Zeamays/, accessed on 18 February 2021), and transcripts were assembled using stringtie software, after which the balltown package of R software was used to calculate the transcript expression of genes in each tissue. FPKM (fragments per kilobase of exon per million fragments mapped) values were used to measure the expression levels of genes.

Maize (B73) was grown under natural conditions in Beijing, China (Experimental base of Research Center of Biology and Agriculture, University of Science and Technology Beijing, China, 116″38′ E, 40″06′ N). Total RNA was isolated using TRIzol reagent (Invitrogen, Waltham, MA, USA) from maize anthers. One microgram of RNA was used to synthesize first-strand cDNA. RT-qPCR was performed using SYBR TB Green ^TM^ Premix Ex Taq ^TM^ (TaKaRa, Dalian, China) with a QuantStudio 5 Real-Time PCR System (ABI, Waltham, MA, USA). *ZmActin7* was used as an internal control. Primers were designed by Primer3 (version 4.1.0) and are listed in Table S6.

## 5. Conclusions

In conclusion, we systematically conducted a genome-wide exploration of maize *PG* genes through various bioinformatic analyses, including elucidating the physicochemical properties, phylogeny, chromosomal location, gene structure, selective pressures, collinearity analysis, and expression profiles of the *ZmPG* genes.

A total of 55 *PG* genes were identified from the maize genome, and all the maize *PG* genes were randomly distributed across the maize chromosomes. Phylogenetic analysis revealed that these maize *PG* genes were clustered into six Clades. The gene structures of the *ZmPGs* were highly conserved in each of the Clades, reflecting their functional conservation. Collinearity analysis showed that a high proportion of the *ZmPG* genes might be derived from tandem and segmental duplications with purifying selection, providing insights into possible functional divergence among members of the *ZmPG* gene family. Furthermore, comprehensive analyses of the expression profiles revealed that *ZmPG7*, *ZmPG34*, and *ZmPG52* have an expression peak in anther development. Promoters of the three *ZmPG* genes have MBS *cis*-elements, suggesting that orthologs of MYB84 in maize may regulate these *ZmPGs* and probably be relative to fertility. These data provide valuable information for future functional investigations of this gene family.

## Figures and Tables

**Table 1:** The polygalacturonase (*PG*) gene family in maize.

Gene Name	Gene ID	Chromosome	Length (aa)	MW	pI	Subcellular Localization Prediction	Clade
*ZmPG1*	Zm00001d034727	1	477	51.69	6.08	extracellular space	E
*ZmPG2*	Zm00001d034559	1	475	51.21	8.98	extracellular space	A
*ZmPG3*	Zm00001d034552	1	441	47.30	8.46	extracellular space	A
*ZmPG4*	Zm00001d034551	1	436	46.90	8.83	extracellular space	A
*ZmPG5*	Zm00001d030583	1	344	37.24	9.02	extracellular space	D
*ZmPG6*	Zm00001d027441	1	463	49.74	6.16	extracellular space	E
*ZmPG7*	Zm00001d002342	2	496	51.30	6.09	extracellular space	G
*ZmPG8*	Zm00001d044110	3	542	58.27	8.47	extracellular space	B
*ZmPG9*	Zm00001d042556	3	401	41.93	8.99	extracellular space	C
*ZmPG10*	Zm00001d040965	3	428	44.61	5.78	extracellular space	D
*ZmPG11*	Zm00001d040725	3	502	53.47	5.17	organelle membrane	B
*ZmPG12*	Zm00001d040589	3	499	51.60	6.42	extracellular space	B
*ZmPG13*	Zm00001d039668	3	437	47.02	9.14	chloroplast	A
*ZmPG14*	Zm00001d044177	3	287	29.77	5.97	extracellular space	C
*ZmPG15*	Zm00001d053662	4	418	43.92	8.96	extracellular space	D
*ZmPG16*	Zm00001d053395	4	451	48.17	8.82	extracellular space	E
*ZmPG17*	Zm00001d052103	4	495	54.11	9.05	plasma membrane	E
*ZmPG18*	Zm00001d050149	4	377	40.29	9.12	extracellular space	D
*ZmPG19*	Zm00001d048696	4	494	52.45	5.24	plasma membrane	E
*ZmPG20*	Zm00001d015825	5	468	49.33	8.74	plasma membrane	D
*ZmPG21*	Zm00001d015821	5	435	45.61	8.62	plasma membrane	D
*ZmPG22*	Zm00001d015129	5	516	54.60	5.00	extracellular space	A
*ZmPG23*	Zm00001d013032	5	487	51.32	6.70	plasma membrane	A
*ZmPG24*	Zm00001d018548	5	232	25.61	4.93	nucleus	E
*ZmPG25*	Zm00001d015820	5	148	15.42	9.00	extracellular space	D
*ZmPG26*	Zm00001d038875	6	471	49.99	5.83	extracellular space	A
*ZmPG27*	Zm00001d038874	6	412	43.66	8.06	extracellular space	A
*ZmPG28*	Zm00001d036824	6	391	41.77	6.39	nucleus	D
*ZmPG29*	Zm00001d036823	6	410	43.44	6.95	extracellular space	D
*ZmPG30*	Zm00001d036822	6	410	43.44	6.59	extracellular space	D
*ZmPG31*	Zm00001d036821	6	410	43.47	6.59	extracellular space	D
*ZmPG32*	Zm00001d036820	6	348	37.09	6.92	extracellular space	D
*ZmPG33*	Zm00001d036819	6	328	35.47	5.98	extracellular space	D
*ZmPG34*	Zm00001d036818	6	403	42.51	9.14	extracellular space	D
*ZmPG35*	Zm00001d036816	6	410	43.28	8.44	extracellular space	D
*ZmPG36*	Zm00001d036815	6	410	43.23	8.44	extracellular space	D
*ZmPG37*	Zm00001d035899	6	486	51.74	6.30	extracellular space	E
*ZmPG38*	Zm00001d034990	6	493	53.18	6.01	plasma membrane	E
*ZmPG39*	Zm00001d037013	6	287	30.06	7.41	extracellular space	D
*ZmPG40*	Zm00001d020931	7	457	50.30	8.00	extracellular space	E
*ZmPG41*	Zm00001d020615	7	516	56.10	9.46	mitochondrion	E
*ZmPG42*	Zm00001d019282	7	508	54.55	6.17	extracellular space	E
*ZmPG43*	Zm00001d011444	8	541	58.09	8.03	extracellular space	B
*ZmPG44*	Zm00001d011369	8	348	37.36	5.49	extracellular space	E
*ZmPG45*	Zm00001d011156	8	436	46.16	9.21	extracellular space	B
*ZmPG46*	Zm00001d009717	8	446	46.99	8.12	extracellular space	E
*ZmPG47*	Zm00001d009667	8	516	54.74	6.84	plasma membrane	B
*ZmPG48*	Zm00001d009341	8	416	43.05	5.83	anchored component of plasma membrane	D
*ZmPG49*	Zm00001d009057	8	423	44.15	5.69	extracellular space	B
*ZmPG50*	Zm00001d010681	8	283	31.20	5.99	nucleus	E
*ZmPG51*	Zm00001d009167	8	766	79.73	9.22	nucleus	B
*ZmPG52*	Zm00001d048079	9	462	49.63	6.01	plasma membrane	A
*ZmPG53*	Zm00001d045974	9	419	43.59	6.24	plasma membrane	B
*ZmPG54*	Zm00001d023813	10	461	49.31	5.25	anchored component of plasma membrane	E
*ZmPG55*	Zm00001d000355	Contig B73V4_ctg206	498	53.70	8.66	extracellular space	D

**Table 2:** The duplication events of *ZmPGs* identified in maize.

No.	Sequence	Duplication Type	Ka	Ks	Ka/Ks	Date (Millions of Years Ago)
1	*ZmPG20 & ZmPG21*	Tandem	0.007	0.06	0.109	1.833
2	*ZmPG30 & ZmPG31*	Tandem	0.002	0.017	0.115	0.521
3	*ZmPG30 & ZmPG29*	Tandem	0.005	0.049	0.098	1.488
4	*ZmPG31 & ZmPG29*	Tandem	0.007	0.056	0.122	1.684
5	*ZmPG36 & ZmPG35*	Tandem	0.005	0.078	0.06	2.375
6	*ZmPG32 & ZmPG29*	Tandem	0.018	0.135	0.133	4.089
7	*ZmPG30 & ZmPG32*	Tandem	0.02	0.144	0.14	4.371
8	*ZmPG31 & ZmPG32*	Tandem	0.023	0.135	0.167	4.1
9	*ZmPG3 & ZmPG4*	Tandem	0.025	0.103	0.245	3.122
10	*ZmPG28 & ZmPG29*	Tandem	0.027	0.233	0.117	7.056
11	*ZmPG30 & ZmPG28*	Tandem	0.027	0.25	0.11	7.574
12	*ZmPG31 & ZmPG28*	Tandem	0.03	0.238	0.125	7.222
13	*ZmPG33 & ZmPG32*	Tandem	0.05	0.346	0.143	10.499
14	*ZmPG32 & ZmPG28*	Tandem	0.11	0.608	0.181	18.424
15	*ZmPG35 & ZmPG29*	Tandem	0.03	0.468	0.065	14.188
16	*ZmPG31 & ZmPG35*	Tandem	0.033	0.445	0.073	13.474
17	*ZmPG30 & ZmPG35*	Tandem	0.032	0.463	0.07	14.027
18	*ZmPG36 & ZmPG29*	Tandem	0.035	0.469	0.074	14.2
19	*ZmPG31 & ZmPG36*	Tandem	0.037	0.448	0.083	13.572
20	*ZmPG30 & ZmPG36*	Tandem	0.037	0.466	0.079	14.106
21	*ZmPG32 & ZmPG35*	Tandem	0.043	0.487	0.089	14.766
22	*ZmPG35 & ZmPG28*	Tandem	0.046	0.488	0.095	14.774
23	*ZmPG32 & ZmPG36*	Tandem	0.05	0.536	0.092	16.242
24	*ZmPG36 & ZmPG28*	Tandem	0.051	0.545	0.093	16.523
25	*ZmPG21 & ZmPG15*	Segmental	0.044	0.615	0.071	18.624
26	*ZmPG43 & ZmPG8*	Segmental	0.042	0.137	0.307	4.151
27	*ZmPG47 & ZmPG8*	Segmental	1.001	0.998	1.003	30.237
28	*ZmPG51 & ZmPG11*	Segmental	0.935	1.357	0.689	41.115
29	*ZmPG49 & ZmPG12*	Segmental	0.705	1.872	0.377	56.732
30	*ZmPG42 & ZmPG1*	Segmental	0.165	1.385	0.119	41.982
31	*ZmPG23 & ZmPG4*	Segmental	0.934	1.342	0.696	40.666
32	*ZmPG47 & ZmPG43*	Segmental	0.649	0.246	2.634	7.464
33	*ZmPG46 & ZmPG44*	Segmental	1.078	0.842	1.281	25.505

## Data Availability

Not applicable.

## Supplementary Materials

The following are available online at https://www.mdpi.com/article/10.3390/ijms221910722/s1.
