# Supplementary material for: Genome-Wide Identification and Characterization of Polygalacturonase Gene Family in Maize (Zea mays L.)
Source: Int J Mol Sci. 2021 Oct 3;22(19):10722. doi: 10.3390/ijms221910722 (PMC8509529; doi:10.3390/ijms221910722)
Supplement: Supplementary file 1 [file ijms-22-10722-s001.zip › Figure S1. Conserved motif anyais of ZmPGs.pdf]

Figure S1

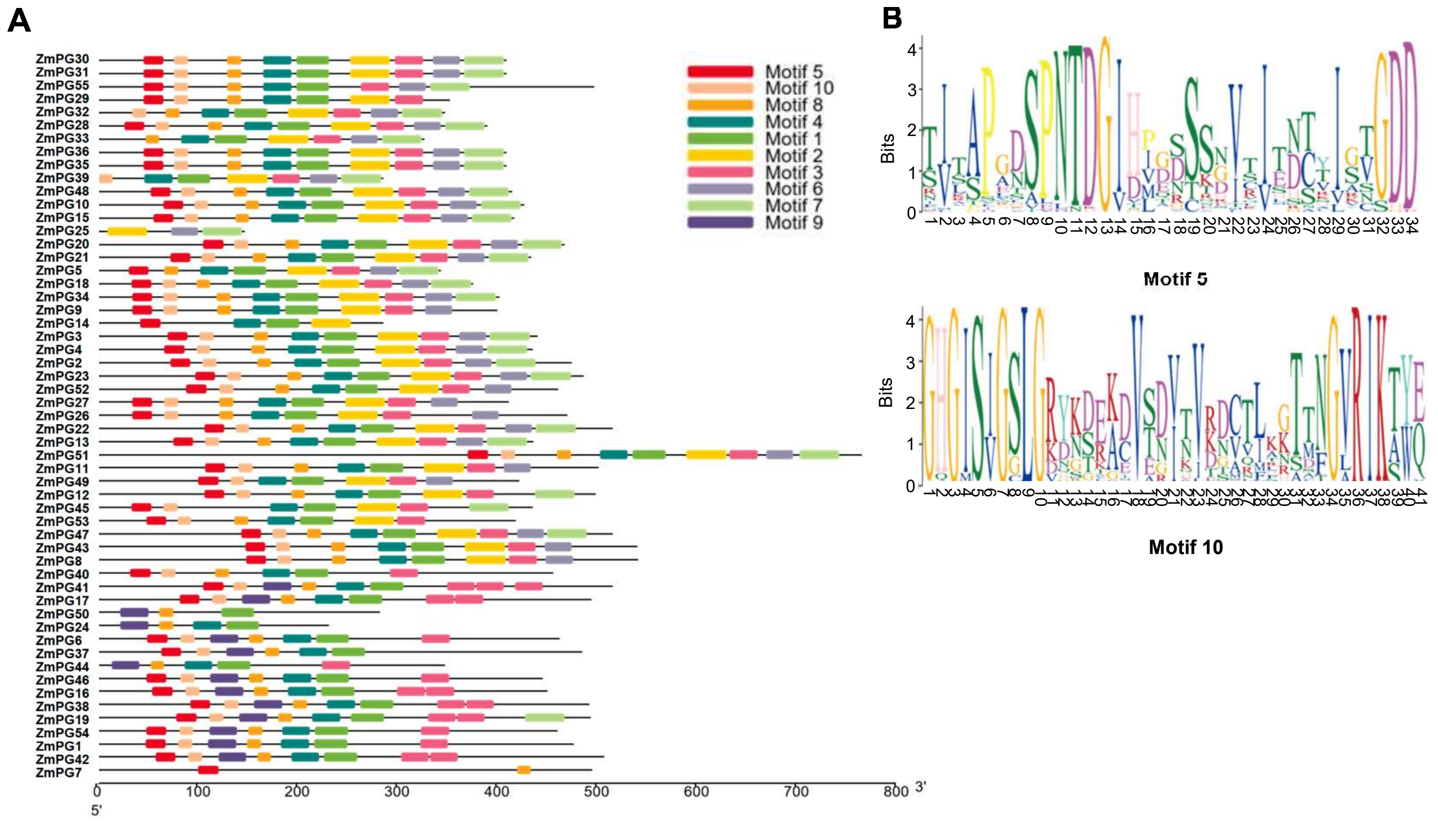

Figure S1. Conserved motif anyais of ZmPGs. **(A)** illustrations of the composition and position of conserved motifs among ZmPGs. **(B)** Two most conservative motifs**,** the heights represent the conservative levels of each amino acid.
