# Supplementary material for: Genome-Wide Identification and Characterization of Polygalacturonase Gene Family in Maize (Zea mays L.)
Source: Int J Mol Sci. 2021 Oct 3;22(19):10722. doi: 10.3390/ijms221910722 (PMC8509529; doi:10.3390/ijms221910722)
Supplement: Supplementary file 1 [file ijms-22-10722-s001.zip › File S1. DNA and protein sequences of ZmPG genes .pdf]

CDS
>ZmPG30
MACTDNAMRALFLLVLFCIVHGEKEESKGIDAKASGPGGSFDITKLGASGNGKTDSTKAV
QEAWASACGGTGKQTILIPKGDFLVGQLNFTGPCKGDVTIQVDGNLLATTDLSQYKEHGN
WIEILRVDNLVITGKGNLDGQGPAVWSKNSCTKKYDCKILPNSLVMDFVNNGEVSGVTLL
NSKFFHMNMYQCKNMLIKDVTVTAPGDSPNTDGIHMGDSSGITITNTVIGVGDDCISIGP
GTSKVNITGVTCGPGHGISIGSLGRYKDEKDVTDINVKDCTLKKTMFGVRIKAYEDAASV
LTVSKIHYENIKMEDSANPIFIDMKYCPNKLCTANGASKVTVKDVTFKNITGTSSTPEAI
SLLCTAKVPCTGVTMDDVNVEYSGTNNKTMAICTNAKGSTKGCLKELACF
>ZmPG31
MACTDNAMRALFLLVLFCIVHGEKEESKGIDAKASGPGGSFDITKLGASGNGKTDSTKAV
QEAWASACGGTGKQTILIPKGDFLVGQLNFTGPCKGDVTIQVDGNLLATTDLSQYKEHGN
WIEILRVDNLVITGKGNLDGQGPAVWSKNSCTKKYDCKILPNSLVMDFVNNGEVSGITLL
NSKFFHMNMYQCKNMLIKDVTVTAPGDSPNTDGIHMGDSSGITITNTVIGVGDDCISIGP
GTSKVNITGVTCGPGHGISIGSLGRYKDEKDVTDINVKDCTLKKTMFGVRIKAYEDAASV
LTVSKIHYENIKMEDSANPIFIDMKYCPNKLCTANGASKVTVKDITFKNITGTSSTPEAI
SLLCTAKVPCTGVTMDDVNVEYSGTNNKTMAICTNAKGSTKGCLKELACF
>ZmPG55
MACTDNAMRDLFLLVLFCIVHGEKEESKGIDAKASRPGGSFDITKLGASGNGKTDNTKDV
QEACASACGGTGKQTILIPKGDFLVGQLNFTCPCKGDVTIQVDGNLLATMDLSQYKEHGK
WIEILRVDNLVITGKGNLDGQGPAVWSKNSCTKKYDCKILPNSLVMYFVNNGEVSGVTLL
NSKFFHMNMYQCKNMLIKDVTVTAPGDSPNTDGIHMGDSSGITITNTVIGVGDDCISIGP
GTSKDCTLKKTMFGVRIKAYEDAASVLTVSKIHYENIKMEDSANPIFIDMKYCPNKLCTA
NGASKVTVKDVTFKNITGTSSTPEAVILLYTAKVPCTGVTMDDVNVEYSGTNNKTMAICT
NAKGSTKGCLKELACFLTLRRLTHLSSYNFSLVLALPICCYPLVGARCRRDSSTSGQLEP
HRAVPSCPKHRIWVSDIPVSFFCANSAIRGGFQPSKLPLAGPQHLHGVRPLQPLPEPELR
PRAPHPLPGAYANRRAPV
>ZmPG29
MACTNNAMRALFLLVLFCIVHGEKEESKGIDAKASGPGGSFDITKLGASGNGKTDSTKAV
QEAWASACGGTGKQTILIPKGDFLVGQLNFTGPCKGDVTIQVDGNLLATTDLSQYKDHGN
WIEILRVDNLVITGKGNLDGQGPAVWSKNSCTKKYDCKILPNSLVMDFVNNGEVSGVTLL
NSKFFHMNMYRCKDMLIKDVTVTAPGDSPNTDGIHMGDSSGITITNTVIGVGDDCISIGP
GTSKVNITGVTCGPGHGISIGSLGRYKDEKDVTDINVKDCTLKKTMFGVRIKAYEDAASV
LTVSKIHYENIKMEDSANPIFIDMKYCPNKLCTANGASKVTVKDVTFKNITGT
>ZmPG32
MAYTDNAMRALFLLALFCVVHGEKEESKGIDAKASGPGGSFEITKLGASGNGPCKGDVTI
QILRVDNLVITGKGNLDGQGPAVWSKNSCTKKYDCKILPNSLVMDFVNNGEVSGVTLLNS
NFFHMNMYRRKDMLIKDVTVMAPGDSPNTDGIHMGDSSGITITNTVIGVGDDCISIGPGT
SKVNITGVTCGPGHGISIGSLGRYKDEKDVTDINVKDSTLKKKIFDVRIKAYEDATSVLT
VSKIHYENIKMEDSANPIFIDMKYCPNKLCTANGASKVTVKDVTFKNITDTSSTPEAVSL
LCTAKIPCTGVTMDDVNVEYSGTNNKTMAICTNAKGSTKGCLKDLACF
>ZmPG28
MHGEKEESKRIDAKASGPGGSFDITKLGASGNGKIDNTKAVQEAWASACGDTGKQTILIP
KGDFLVGQLNFTGPCKGDVTIHVDGNLLSTMDLSQYKEHGNWIEILRVDNLVITGKGNLD

GLGPAVWSKNSCAKKYDCKILPNSLVMDFVNNGEVSGVTLLNSKFFHMKMYQCKDMLIKD
VTVTALGDSPNTDGIHMGDSSGITITNTVISVSDDCISFGPGTSKVNITGVTYGSGHGIS
IGSLGRYKDEKDDTNINVKDCTLKKTTFGVRIKAYEDAASVLTVSKIHYENIKMEDSANP
IFIDMKYCPNKLCTANDASKVTVKDVTFKNITGTSSTPEAVSLLCSAKIPCTGVTMDDIN
VEYSGTNNKTMAICTNAKGSTKGCLKELACF
>ZmPG33
MAYTDNAMRALLRSSTSCMVDGNLLATTYLSQYKEHGNWIEILRVDNLVITGKGNLDGQG
PFVWSKNSCTKKYDCKILPNSLVMDFVNNGEVSGVTLLNSKFFHMNMYQCKDMLIKDVTV
TAPGDSPNTDGIHMGDTSGITITNTVIGVGDDCISIGPGTSKVNITGVTCGPGHGISIGS
LGRYKDEKDVTDINVKDCTLKKTTFGVRIKAYEDAASELTVSKIHYENVKMEDSTNPIFI
DMMYCPNKLCTANGASKVTVKDVTFKNITGTSSTPEAISLLCTAKIQCTGVTMDDVNVEY
SGTNNKTMDICTNTKGCTKGCLKELACF
>ZmPG36
MACIDNAMRALFLLALFCTVHGEKAKSKDNDSKASGPGGSFDITKLGASGNGKTDSTKAV
QEAWASACGGTGKQTILIPKGDFLVGPLNFTGPCKGDVTIQVNGNLLATTDLSQYKDHGN
WIEILRVDNLVITGKGKLDGQGPAVWSKNSCVKKYDCKILPNSLVMDFVNNGEVSGITLL
NSKFFHMNMYKCKDMLIKDVNVTAPGDSPNTDGIHMGDSSGVTITNTVIGVGDDCISIGP
GTSKVNITGVTCGPGHGISIGSLGRYKDEKDVTDINVKDCTLKKTANGVRIKAYEDAASV
LTASKIHYENIKMEDSGYPIIIDMKYCPNKLCTANGASKVTVKDVTFKNITGTSSTPEAV
NLLCSAKIPCTGVTMDDVNINYSGTNNKTMAVCKNAKGSAKGCLKALACF
>ZmPG35
MACIDNAMRALFLLALFCVVHGEKAKSKDNDAKASGPGGSFDITKLGASGNGKTDSTKAV
QEAWASACGGTGKQTILIPKGDFLVGPLNFTGPCKGDVTIQVNGNLLATTDLSQYKDHGN
WIEILRVDNLVITGKGKLDGQGPAVWSKNSCVKKYDCKILPNSLVMDFVNNGEVSGITLL
NSKFFHMNMYKCKDMLIKDVNVTAPGDSPNTDGIHMGDSSGVTITNTVIGVGDDCISIGP
GTSKVNITGVTCGPGHGISIGSLGRYKDEKDVTDINVKDCTLKKTANGVRIKAYEDAASV
LTASKIHYENIKMEDSGYPIIIDMKYCPNKLCTANGASKVTVKDVTFKNITGTSSTPEAV
NLLCSAKIPCTGVTMDDVNIKYSGTNNKTMAVCKNAKGSAKGCLKELACF
>ZmPG39
MIPKGDYLAGPLNFSGPCTLTTSSSPARAPSTARAKKCGTTTNSLVLDFVNNGTVSGITL
LNAKFFHMNVFQCKDMTIKDVTITAPEDSPNTDGIHIGDSSEVTISGTTIGTGDDCISIG
PGSSGINITGVTCGPGVGSLGRYKDEKDVTDVNVKDCTLKKTSNGVRIKAYEDAASVLTA
SKLHYENIAMEDVANPVIIDMKYCPNKICTAKGDSKVTVKDVTFKNITGTSSTPSAVSLL
CSDKIPCSDITMDKVKVEYKGTNNKTMAVCNNAKGSATSCLKELACL
>ZmPG48
MASAHNALRVFFILAVVCAVCTAKRTGANKEESAAAPGGAAGGSGGTFDISKLGATSDGK
TDCTKAVQDAWTSACEATGSATVVIPKGDYLVGPLNFTGPCKGSNIAIQLDGNLLGSNDL
DKYTASWIELSHVNNIGITGSGTLDGQGTAVYSKSKTDNVKAMPNTLVLFHVINATVAGI
KLLNSKFFHINIDNSESITVKDVNITAPADVENTDGVHVGGSSKISILNSTIGTGDDCVS
IGPGCNGVLVDSITCGPGQGISVGCLGRYKDEKDVSDITVRNCVLKNTTNGVRIKSYVDA
ESVLTASHLTFENIRMEEVANPIVIDQYFCPQKVCPGKRSNSSHVSVKDVTFRNITGTSS
TPEAISLLCSETQPCSGVSLIDVNVEYAGKNNKTMAVCSNAKGTAKGSIEALACLV
>ZmPG10
MAASTNNTLRVLFTLMVVCAAVCTAKRTVAKAGDLAPAPAPLGAGGATAAPEGAARASRT

FDISKFGATSDGKTDSTQAVQDTWTSACGAMGDATMLIPKGDYLVGPLYFSGPCMGDITI
QLDGILLGSNDLAKYTASWIQVSHVNNIVINGSGTLDGQGTAVYTKSKTDVVKAMPNTLV
LFYVTNGTVSGIKLLNSKFFHINIDTSKDITVKDVNITAPADVENTDGVHVGGSSNVRIT
NSTIGTGDDCVSIGPGSDGVMVNNITCGPGQGISVGCLGRYKDEKDVSDVTVRDCVLRNT
TNGVRIKSYEDAESVLTASHLTFENIRMEEVANPIIIDQYFCPQKVCPGKRSNSSHVSIK
NVTFRNISGTSSTPEAVSLLCSETQPCSGVSLIDVNVEYAGKNNKTMAVCSNAKGTAKGS
LQALDCLV
>ZmPG15
MAFISSAAMKAAVAALLVFAAVSPAARAVAAEAEAKAKAVGGAPSVPAGSLDIAQLGAKG
DGKSDSTPMVLKAWKHACEATGQQKIVIPKGNYLTGALDLVGPCKSSIIIRLDGNLLGTG
DLNAYKRNWIEIQNVDNLSINGHGTIDGQGALVWNKNECQRSYNCKILPNSLVLDFVTNA
QIRGITLLNSKFFHMNIFGSKNVVIDKVTIKAPGNSPNTDGIHIGDSSNVTISGTTIAVG
DDCVSIGPGSKTIRVKGVKCGPGHGISVGSLGRYKDEKDVEDVKVTGCTLAGTTNGLRIK
SYEDSKSSLKATKFLYQDVTMDNVSYPIIIDQKYCPNNICVKSGASKVAVNDVVFKNIHG
TSNTPEAITLNCANNLPCQGVQLINVDIKYNRSDNKTMSVCKNAIGKSIGMAKELACV
>ZmPG25
MIRIHGVKCGPGHGISVGSLGRYKDEKDVEDVKVTGCTLAGTTNGLRIKSYEDSKSSPKA
SKSTAPNNICVKSGASKVAVNDVVFKNIHGTSNTPEAITLNCANNLPCEGVQLVNIDIKY
NGSGNKTMAVCKNAIGKSIGLAKELACI
>ZmPG20
MEALKKAEDTNVSPRCLAIPNIAFHPSWHRSIYVCSSAPPQSRHRHQSAILSITKMAFIS
NVAVKAAGVAALLVVAVVSPAARAAAVAVAGGAPSVPAGPLDIAQLGAKGDGKSDSTPMI
LKAWKNACEATGVQKIVIPPGNYLTGGLELKGPCKSSIIIRLDGNLLGTGDLSAYQRNWI
EIENVENLSINGHGTIDGQGALVWSKNQCQHSYNCKILPNSLVLDFVTNVQIRGITLLNS
KFFHLNIFECKNVLIDKVTVKAPGDSPNTDGIHIGDSSNVTISSTTIGVGDDCISIGPGS
KMIRIHGVKCGPGHGISVGSLGRYKDEKDVEDVQVTGCTIAGTTNGLRIKSYEDSKSSLK
ASKFLYEGITMDNVSYPIIIDQKYCPNNICVKSGASKVAVNDVVFKNIHGTSNTPEAITL
NCANNLPCQGVQLVNVDIKYNGSGNKTMAVCKNAIGKSIGLAKELACI
>ZmPG21
MCSSAPPQPRHRHESAILSNTKMAFISNIAVKAAAVAALLLVAAVSPAARAAAVAVAGGA
PSVPAGPLDIAQLGAKGDGKSDSTPMILKAWKNACEATGVQKIVIPPGNYLTGGLELKGP
CKSSIIIRLDGNLLGTGDLSAYQRNWIEIENVENLSINGHGTIDGQGALVWSKNQCQHSY
NCKILPNSLVLDFVTNVQIRGITLLNSKFFHLNIFECKNVLIDKVTVKAPGDSPNTDGIH
IGDSSNVTISSTTIGVGDDCISIGPGSKMIRIHGVKCGPGHGISVGSLGRYKDEKDVEDV
QVTGCTIAGTTNGLRIKSYEDSKSSLKASKFLYEGITMDNVSYPIIIDQKYCPNNICVKS
GASKVAVNDVVFKNIHGTSNTPEAITLNCANNLPCQGVQLVNVDIKYNGSGNKTMAVCKN
AIGKSIGLAKELACI
>ZmPG5
MEARPRLLLVVVAIFVSISAVAAAVINVKNYGAHGNGVNDDTKGTLKAATDLKRFGNDWI
EFGWVNGLTVAGGVIDGQGAASWPFNKCPIRKDCKVLPTSVLFVNNQNTVVRDVTSVNPK
FFHMALLSVKNIRMSGLKISAPSNSPNTDGIHIERSSGISIMDTHISTGDDCISIGQGND
NVDVARVQCGPGHGMSVGSLGRYSGEGDVTRVHVRDMTFTGTMNGVRIKTWENSPSKSN
A
AHMVFENMVMRDVQNPIIIDQKYCPYYNCEHKYVSGVTLNDIHFKNIKGTATTPVAVLLR

CGVPCRGLVLQDVDLRYKGQGGTSAKCENAKAKYLGYQFPKPCP
>ZmPG18
MEARPRLLLVAAVAVFVSVSAAAGAAVVINVKNYGAHGNGVNDDTKPLMAAWKAACGSAG
AVTMVVAPGTYYIGPGTLKAATDLKRFGNDWIEFGWVNGLTVAGGVIDGQGAASWPFNKC
PIRKDCKVLPTSVLFVNSQNTVVRDVTSVNPKFFHMALLSVKNVRMSGLRIRAPPNSPNT
DGIHIERSSGVSIVDTHIGTGDDCISVGQGNDNVEVSRVQCGPGHGMSVGSLGRYSGEGD
VTRVHVRDMTFTGTTNGVRIKTWENSPSRSNAAHMVFENMVMKDVQNPIIIDQKYCPYYN
CEHKYVSGVTLKDIHFRNIKGTATTPVAVLLRCGVPCQGLVLQDVDLRYKGQGATSAKCE
NAKAKYVGYQFPKPCSP
>ZmPG34
MPLRLAALLLLATTATWTAGVTAAAAGRVFNVSDFGAVADGRTDDSEAFLRAWTEACATP
GRPAVVVPRGDYLLHPLVFRGPCRGYVEVHVGGVLRAPPGLAAFRGCREWVHFSSIDGLL
VTGGGTFDGRGATAWPLNECPQKRDCRLLPTSIKLGLVRNATITGVTSLDSKFFHVAVVG
SQDVRIHGVSIRAPRSSPNTDGVHIQGSSNVRVTDSAVATGDDCVSVGPGSSDVLVSGVA
CGPGHGISVGSLGRYPGEGDVRRLRVANCTVAGTSNGVRIKTWRGGSWPPTAVAGLVFED
IVMRKVRNPIIIDQEYCPYPSCRESEQRPSAVRISDVKFRNIRGESATKVAVKLSCSEAS
PCRELELRDIDLRYVKRGVATQSRCAHVAGGVVGGTLVPPSCI
>ZmPG9
MAMPKPVLSLLVHLHAAVLFLPAPAGGAVYNVLRYGARPDGVTDAAGPFLRAWADACRSP
LPAAVYVPPGRYLVRSATFTGPCHTRAVTFAVAGTVVAPAAYGARGSSGRWITFENMDGL
VVAGGGTLDGRGRALWACRQRGQRDCPTPTSTLTIANSKDVVVAGLRSVDSELFHVVVLQ
CHGVTVRGVTVEAPADSPNTDGIHLHMSTHVSVYDARISTGDDCISIGPGNSHLWIERVA
CGPGHGISIGSLGKQQGTEVEAVQNVTVKTTWFTGTMNGLRIKTWGGSKRGFVTGVTFAD
STMSGVDNPIIIDQNYCPTSSGCPGAGRSSSIRISDVRYVGIRGSSATPVAVNFDCSRSY
PCSGISLQDVALTYQNRAAAKSYCRNVQGTALGLVLPPSCL
>ZmPG14
MAPFFDAYYSSLVPLLFSCVLSSGAGAAEAAAAAAYKYNVAGFGARPDGRTDSAGAFASA
WPAACRSQEPATVLHQPWRQQQPAGTTAPGSCSTASTASLCGAAPSTAAARRCGLWACKQ
AAEHGGCPSGATSLKVLNSRDVVISGLTSVESELYHVVVEGCEGVTVQDVQIVAPGSSPN
TDGIHVQASSQVTVTRTSIRTGDDDCVSVGTGTTNLRVEHGWSLGKEREESGVENVTVTG
AAFVATDNGLRLRIKTWARARVDGAYVRGVVFEHALMHDVRNPIIID
>ZmPG3
MASSSGMKLVYMAPLLPLLFMSGVLEAAGAGSSADGTNGNLHGSVTSRPAAAPRGRSLPE
SQSVFSLDRYGARGDGRNDDTRALEMAWKAACASPRPAVVLVPAGRRYLLKLLTLRGPCK
SSVTLTVKGTLVASPNRADWSDSDRRHWIVFRSIDELTVNGGGAIDGNGEKWWPHSCKIN
KALPCKEAPTALSFHYCVGLKVEDLKIMNSQQIHMSVEDSANVLLARLSITAPGTSPNTD
GIHITRSKDVRVTDCKIKTGDDCMSIENGTHNLHVSKVTCGPGHGISIGSLGDDNSRAEV
SGITIDSVQLHGTTNGARIKTYQGGSGYAKDITFQNMVMYDVKNPIIIDQNYCDRAKPCG
EQESAVQVSGVVFKNIRGTTSTKDAIKMNCSENVPCQGITLQNIDLEMQDGKGSTRSTCR
NAKWREFGTVHPQPCTATDTE
>ZmPG4
MKLVYMVTLLPLLFMSGVLEAAGAGNDSSADGTNGNLHSSGTSRPAAAPRGRSLQASQSS
VYSVDRYGARGDGRNDDTRALEMAWKAACASPRPAVVLVPDGRRYLLKLLTLRGPCKSSV
TLTVKGTLVASPNRADWSDNDRRHWIVFRSIDKLTVNGGGAIDGNGEKWWPHSCKINKAL

PCKEAPTALSFHYCVDLKVEDLKIVNSQQIHMSVEDSANALLARLSITAPGTSPNTDGIH
ITRSKDVRVTDCKIKTGDDCMSIENGTHNLHVSKVNCGPGHGISIGSLGDDNSRAEVSGI
TIDSVQLHGTTNGARIKTYQGGSGYAKDITFQNMVMYDVKNPIIIDQNYCDKAKPCGEQE
SAVQVSGVVFKNIRGTTSTKDAIKMNCSENVPCQGITLQNIDLKMQDGKGNTRSTCQNAK
WTEFGTVRPQPCTAIK
>ZmPG2
MASSMKPTMLASTAALPLLALLLLLLHLSSGALEAVVVSASNGTAADGASYTCSGSLTWP
SSSHQSVFSLDRYGARGDGRHDDTGALARAWEAACASPRPAVLLVPGGRRYLLKLVVLRG
PCRSRVAVTVKGTLVASPNRADWSDSDRRHWIVFRSVDRLTVHGRGSIDGNGHSWWPHSC
KINKALPCKAAPTALSFHYCTNLRVENLKMMNSQQIHMSIEDCASVQVSRLSITAPGTSP
NTDGIHITRSKDVRVTNCKIKTGDDCMSIEDGTHGLHVSGVVCGPGHGISVGSLGDDDSR
AEVSGITIDSVQLHGTTNGARIKTYQGGSGYARDITFQNMAMHGVRNPIVIDQSYCDRAE
AEPPCREQRSAVQISDVVFRNIRGTTVTRDAIRMSCSRDVPCRGIVLQNIDLKMQGGKGH
AESTCQNAKWRKSGKVVPQPCTSKEEGKVGQLLEFLLDHELASSLRSWSSWSLWN
>ZmPG23
MHSSLLLLLAMPTTMVVAAAAARTDKPRLMWLPPLVVALLLLSGALLEAAAAAAGTSTTS
ADDAASSTSSLAAERAATGRRSRLLVASQSTFFSLDSYGARGDGRHDDTQALARAWEAAC
FSPRPAVLLVPGGKRYLLKLVALSGPCKSSVTVTVEGTLVASRDRSDWIGGGGGTDRRRH
WIVFRQVDGLTVGGGGAVDGSGETWWKHSCKINKDLPCEEAPTALSFHYCTSLRVHDLKI
VDSQQIHVSIEDCTGVQLTGLSITAPGTSPNTDGIHITRSRDVQVTNCKIKTGDDCLSIE
AGTHDLHVSQIVCGPGHGISIGSLGDDNSRAEVSGITIDTVQLYGTTNGARIKTYQGGSG
YARDITFQNMAMHGVENPIVIDQDYCDKRQATSPPCEAQGSSSSSAVEVSDVAFRNIRGT
TVSKDAIKLSCSWNVPCHGITLQNISLEMLGGKGTAESTCRNARWRTSGTVLPQPCTSFI
DLGHGSN
>ZmPG52
MLVPSSPLLSSSPPLHSPEPPPHLRMGGSTTTVVVMLIVVLHYYMLLVDGASWDEVSSEA
DGPAPAEGPDDELWLPRAGPPPRVVNVDDYGAADAGVDAAEAFLAAWSEACNSSAGRSVF
LVPEGKAYLLMPVSFRGPCRAASVAAMIKGTLEAPSDRSVWLERGLQEWITFEGVDRLRV
LGGGTLDGNGMQWWINSCKLNRSARCVTGPTALYLRRCTRLVVEELQVRDSMQMHVAIAY
SRDVAVSRLSITAPAWSPNTDGIHVSNSREVSISRCTIATGDDCVSIVTGSTFVRVTSIF
CGPGHGISIGSLGANNSWAHVSDVLVEKATLLGTTNGVRIKTWQGGYGYAERISFRDISM
RNVTNPIIIDQNYCDSARTTTPSSCHDQGSAVAVRNISYRNIHGTSASRVAISLVCSAAL
RCDGIRMQDVYLVGEGRYATCSYRNATVVQSGYTFPFCSAEM
>ZmPG27
MAKAVPSPLAAVAVLALLLLCRGAEARVLLTLDDFGGVGDGIANDTQALLDAWAAACSST
QEAVLAVPAGKVYQIWPVQLSGPCKKRLKLLISGAIVAPSSPDEWAGRDPMKWLYVYGVD
GLSVSGGGTIDGMGQQWWASTCKRKKTQPCYSGPRPKAVHFEECREVSVQGVTLQNAQQ
F
QLTFTRCSCVKASFLRVVAPADSPNTDGIHLNDTSHVRITDNLISTGDDCVSMVGNCSDV
RVKDISCGPGHGISIGSLGKNRTTDMVEDVKVDTCLLTNTTNGVRIKSWQGGTGFARDLR
FENIVMRNVSNPIIIDQYYCDQPTPCANQARHSSTQAVEVRRVEFAGIRGTSATPQAISI
ACSDAVPCRDLELANVNLTLEGGGAGRATALCYRASGKSAGTVVPPSCLARS
>ZmPG26
MMARGAVLLLLAAAFAAALLPDAAESRILLTLHDFGAVGDGVADDTKALAGAWTAACAAA

DDVILNVPAGGTFRIWPLTLAGPCRSEIKLLISGDIVAPESPQDWGQGQSDQWLHFHKVR
DLTVTGGGIIDGRGQQWWAQSLARAQPAPKAVHFEDCQGISVKGITLQNSQSYHLTFTRS
SDVEANYLRVTSPEHSVDTKGIHLVDSYNVHVMDNLISTGDDCVSIVGNCTDVRLRAISC
GPGHGISIGTLGVNSSVDYVEKIKVDTLFISNAENGVRVRTTENGGGGGFARKVKFESIV
MRNVTNPIIVDQGISSDDPPPSSPEAVALVHHTLIRLPSLSAFTPRDAAAYISVRQAVSL
NSRAGKFCAAQAATASAAVQVEKINYIDITGTSASERAVTFSCSDARPCRRLSLDNVNLT
QVDGSEASSYCRQAFGRSVGTVVPESCLSKEDFVHHVPPQRSEEDGEDSES
>ZmPG22
MGAAPAARRCALLLALVVLVAAAGERGGSGAGGEAGAEQVLRLWSSADSARGGEAVDEED
RFFRWEWEEEEEDDDNNDEEEGEEEDDHAVVVQRKGACRNVVNVDSFGAAGDGVADDTQ
A
FASAWKTACSLDNAVFLVPAGRRYKVGAIQFVGPCKDRRMIIQIQGTVVAPEEPSEWDPR
SPRLWLLFSGLAGARIQGGGVIDGSGSKWWASSCKINRSNPCRPAPTAVTIDSCRGVRVR
GLRVQNAQQMHVTVSRSRGVRVAGLRIDAPEDSPNTDGIHVAESTAVTIQSCRIGTGDDC
ISIVNGSFGVKMRDIDCGPGHGISIGSLGKGGAFAAVADVALDRARISRAQNGVRIKTWQ
GGAGYVRGVRFADVAVDGVDHPIVIDQFYCDVTRRQRDGGGGGACANQTSAVAVSDVSYR
NISGTSRRAEAIRFACSDAVPCTGIVLSNINLRRADDDGEVQTVCNCAVGLDYGRVQPAA
DCLRSSTCGGTPDGHHHHDDDDEEQGKDDAVLHTEL
>ZmPG13
MAIIVSSSARSTLFILSSITNHVYRRQVVSALKTTAAAARRHTAFAMLLAFVTLLLVLAT
RPAQCHQQSYDVVRNFHAAADGKTDDAKAFLAAWKAACSDEARPVVVVPGGRTFLLSQVT
FQGPCKSPITIQLDGRIVAPNRIWTTQPANLLVFHGVDSLTLDGNGEIDGRGAIWWDCYN
HKRCNARPILLAFSFCDHLRVTRIRLTNSADKHMTLFRCSQALVDGVSIAAPPDSPNTDG
ITVASSNHTVISNCSIRSGDDCVSILSQTRNVTVTRSTCGPGHGISVGSLGRSESAVVEQ
IVVTNCSFVGTMNGVRIKSWQGGKGYAKGFLFAGLNMTGVQYPIVIDQFYCPQGNCPVKP
GGVAITDARFIDIQGTSSRQEAIRLLCSQSVHCHGIYLSNVNLSWVNHTAPSNATILNAH
GTTEGVVVPKIQFLQPL
>ZmPG51
MPPATSTRPPFRRPVPLLTVVVSVALLSLVCFLVAADASRSLHYHYHLRKHRRHHGHHRA
ENYSHISLPPAAPPPGADGDSPAGPVGLPPDVGGALPPSSCSDKPCPLSPSPSRAPAGAP
CSTPGSAKPPALTLATPPSPAPATPPSHSPPNPPSSHQPFQRPTLPLAKAPPSSKPPSPD
IAPFHPHAKPPRALPDAKSPSQPPKRAPAPSPLSPSKPRKPPSCTLATPPQAQTSRLAPA
SAKPPMRSPAHPPKLSPASPLAHPPVEPPKSALAKPPGFSFSHTTKPQPPVASMVKPPRL
APAKPPAPSPLPAAQPPRCSTANPPVAPTASAKPPAYPPAAASKPIPPPPLPPAANNSSS
AWGNVFDVRAFGASGSGSGNDTRALRAAWKAACSSNSTTPTLLVPSDGVFTISSTIFAGP
CKSAVTFQIDGVLMPPDGPASWPASDSRKQWIVFYKANGMTLAGEGTIEGNGEEWWDLPC
KPHRGPNGSTLPGPCDSPALVRFFLSNDVTVRGLRIENSPQFHLKFDDCERVRVDGLFVR
SPAFSPNTDGVHVENTTSVQILNSRIYNGDDCVSIGAGCSDVRIENITCGHGHGISIGSL
GVHGTRACVSNITVRNARILDSDNGVRIKTWQGGTGAVTAVEFAGVQMQNVKNCILIDQY
YCLGSGCANLSSAVRVAGVTYRDIRGTYNPQASAPIRLACSDAVACTDITMSGVELLPAS
GGGARALLADPYCWNAYGVMETLTLPPVYCLQEGRPESLQDQLTSC
>ZmPG11
MRPAPETSTRRPQLRRRRPVPLVTVAVSLALISLACLPVFADASRALHRDREEHRRQHHR
TEDKKSRVSLPPDADGDSPPEPPDSGGAPPPADDSDCGNVFDVRAFGASGDGSCVDDDTA

AFRAAWKAACSSDSAAATLLVPSDGVFTIASTIFSGPCKPDLTFQIDGVLMPPDGPASWP
ATDSRKQWIVFYKADRLTLAGEGTIEGNGEEWWDLPCKPHRGPNGSTLRGPCDSPALIRF
FSSNDVTVCGLRIENSPQFHLRFDDCERVRVDGLFVSSPASSPNTDGVHVENTKSVQILN
SRIYNGDDCVSIGAGCSDVHVENITCGHGHGISIGSLGVHNTHACVSNVTVRNVRILDSD
NGVRIKTWQGGAGAVSAVEFTGVQMQNVKNCIVIDQYYCLGHGCANQTSAVRVAGVAYRD
IHGTYNPQAGAPIRLACSDAVACTDITMSGVELLPAGGDDGALLADPYCWNAYGVMETLT
LPPVYCLLEGSPESLQDPLTSC
>ZmPG49
MEFTVVRTAIVLLLSLAVSSTFLCGGVQGGRHYHYHHTTHTKHNTAHPPSAHAPGPASGR
RLPPVSPPPSDSSGYPTPGAAPEPAPAPAAAGTMYDVVKDFGAVGDGVTDDTDALKTAWD
TACQDDGDSVVLAAAGYSFLVHTTVFTGPCQGSVTIQALRFFMTNNVTVQGLKVQNSPEF
HIRFDSCCGVVASGLSISSPALSPNTDGIHVENTEDVLITNTAVSNGDDCVSIGAGTRNM
HVENVTCGPGGHGISIGSLGKQGSRACVANVTVRNAVIRHSDNGVRIKTWQGGSGAVSSV
SFENVRMDAVRNPIIIDQYYCLSKSCDNATSAVFVSGVSYAGIRGTYDPRTTPIHFGCSD
AVPCTNITLSDVELLPASGDSTIDNPFCWNVYGSTATPTVPPVPCLIEGVPRRVDDNSTL
KCY
>ZmPG12
MEFTARTAIALLLSLAVSSSFLCGSVQGGRHHHHGKHAKHSSAHPPSAHAPGPASGRRHA
RPVSPPAPPPSSGSGGYPTPGGAAPEPAPAPAAAAAGGTVYDVVKDFGAVGDGVTDDTGA
IKTAWDTACQDDGASVVLAAAGYSFLVHTTVFTGPCQGSVTIQLDGTIVAPSDPDMWPAN
SKRNWLLFYQAHGMSLRGAGLIDGKGQKWWDLPCKSHKGQGGSSGHGASCDSPVALRFF
M
TNNVTVQGLKVQNSPEFHIRFDSCRGVVASGLSISSPALSPNTDGIHVENTQDVLITNTA
VSNGDDCVSIGAGTLNMHVENVTCGPGGHGISIGSLGKQGSRACVANVTVRNAVIRHSDN
GVRIKTWQGGSGAVSSVSFQNVRMDAVRNPIIIDQYYCLSKSCENATSAVFVSGVSYAGI
RGTYDPRTPPIHFGCSDAVPCTNITLSDVELLPASGETIDDPFCWNVYGSTATPTVPPVP
CLIEGVPRNLDDNSSLKCY
>ZmPG45
MRSSVASAALLVTCLVVAAAARSTGRTTLSVLSFGAAGNGVTDDAQALVAAWQVACRVPR
ATVLVLPSGHRFLLSPVTLQGPCSAKLTLQIDGTVLAPPEMGSWPKPRRPLQWLNFKWLQ
GFSIQGTGAVDGGQQNLDLDMKCCLSITHFYLQTSTGTGHWHSPGVKPTLIRFYNSFNVS
VRNIRISNSPQCHLKFDSSGGIRVKNVTISSPGDSPNTDGIHLQNTKDVEIRSSTIGCGD
DCVSIQTGCSNVHMKNVVCNPGHGISLGGLGKDNSLACVSDVVAENIVVQNALYGVRIKT
WQGGVGSVRNITFSNVRVANVATPIAIDQFYCDRGGARCANRTGAVAIAGVAYRRVVGTY
TFQPLRLACSDARPCTGVTMLDVRLSPAAAGGTVAPLCWNSYGEARGTIIQPLSVGCLQR
SNGYAMPLTQPFNYTC
>ZmPG53
MGQSIQNVCLANNATFLSALLLLAVTTSGGPSLQCCLARSETHRRPPPTPPDGPHHPPAA
PAFADAWSAACAAEASTVLVPASYVFLVGPIAFTGGDSCEPNVVFQVDGTILANTGSTAW
RSGYATQWLEFKSVRGLTIQGCGVIDGQGSHWWSRNPLVDQGQTTTALRVYQGDNVTVTG
ITIRSSPKFHLTLDTCRAVEVHGVTIASPGDSPNTDGIHLASSVGVSIHHTTIACGDDCV
SIQAGCSDVSIRNVYCGPGHGISVGGLGKGGATATVSDVTVQDVTFNHTMTGVRIKTWQG
GSGSVKNVRFSGVRVSAVKTPIVIDQYYCDHTACTNQTSAVAVVGAAYQGVAGTYTQRPV
YLACSDAAPCSGIHLADIQLQPVKDSGYRVQGPFCWKAHGDEMRPVEPPVDCLITAGAP

>ZmPG47
MTQVKLLSSRVSLPLFKHPLSFPPQAQKPCPSLFCSSSFSVMLRLKALTFLLLLLLLALC
SGVHLCDARSGTRWSRTTRGGGSGAAPSASAAAIGRKGKARSSGSSHRQSGEGSRSPTD
G
DPVQGDGSQAPPPQDTTVFSVVEFGAKGDGVTDDTQAFEAAWAAACKVEASTVLVPSGLE
FVVGPISFSGPYCKPNILFQWLEFTKLTGIAVQGSGVINGRGQDWWTYADPNDADDESVE
MLLPRTKPTALRFYGSSNVTVAGITIVNSSQCHLKFDSCEGVMVHDLSVSSPEDSPNTDG
IHLQNSRGVDIHHATLACGDDCISIQTGCSDVNIHSVNCGPGHGISIGGLGRFNTKACVS
NVTVRDVKMFRTTTGVRIKTWQGGSGLVQGVRFSNIQVTEVQTPIMIDQFYCDRATCRNQ
TSAVAVSGVQYEDVRGTFTVRPAHLACSDSSPCSGITLAGIQLQPLPLPQHRRLSYDPFC
WQAFGELYTPTVPPIPCLQIGRPAAGNSVLSDGDLC
>ZmPG43
MKLRAKGIGLLLLLVLLALCSTIEVGEARRGKHWRSSRSSPGSSQLKKGKGKKSSSHRQY
GANRPGPKPPVSSTPSSGAGKGNHQNPYQPSPSPSPNAPDIPPMPSPANGSRHSTPEPPA
PSCGKGQQQPSQPPPATSQGAVFNVVDFGAKGDGVTDDTKAFEGAWAAACKKGACTVLVP
PELEFLVGPISFSGPYCKSNIVFQLEGTILAPTSAKSWGSGLLQWLEFTKLNGMVIQGNG
IINGRGQQWWTYSDTEDEDDDDTYDVEFERMPQIKPTALRFYGSFNVVVAGITIVNSSQC
HLKFDNCQGVMVHDLTISSPENSLNTDGIHLQNSKDVSIHHTNLACGDDCISIQTGCSNI
NIHNVNCGPGHGISIGGLGRDNTKACVSNVTVRDVNMFRTMNGVRIKTWQGGVGLVQDIR
FSNIQVSEVQTPIIIDQFYCDRSTCRNQTSAVAVSGVQYENIRGTFTIKPVHFACSDSLP
CSGISLTGVQLRPVQVPHYHLNNPFCWQAFGELYTPTVPPIACLQIGKPAGNNLQSYDDI
C
>ZmPG8
MKLGAKGLGLLLLLVLLALCSTIEVGEARRDRHWRSSRTSSSQLLKKGKGKKTSSRRQYG
SNRPSPKPPASSTPSSGAGGKGNQTPYQPSPNAPHIPRPSPPANGSAHSTPKPPTPPSCG
KAHQQPSQSQPPPPATSSQHGAVFNVVDFGAKGDGVTDDTKAFQGAWAAACTQGASTLLV
PPELEFLVGPISFSGPYCKPNIVFQLEGTILAPISAKSWGSGLLQWLEFTKLNGIVIQGN
GIINGRGQQWWTYSDPEDEDDDDTYHVEFQRMPQIKPTALRFYGSFNVVVSGITIVNSSQ
CHLKFDNCQGVMVHDVTISSPENSLNTDGIHLQNSKDVSIHHTTLACGDDCISIQTGCSN
INIHNVNCGPGHGISIGGLGRDNTKACVSNVTVRDVNMFRTMNGVRIKTWQGGVGLVQDI
RFSNIQVSEVQTPIIIDQFYCDRSTCRNQTSAVAVSGVQYENIRGTFTIKPVHFACSDSL
PCSGISLTGVQLRPVQVPHYHLNNPFCWQAFGELYTPTVPPIACLQIGKPAGNNLQSYDD
IC
>ZmPG40
MTSPIQLAAAPLLLLVVVVAGTRSSRIFSVADYGAAGDGFRYDTAAIQAAVDACAAAGGG
RVLLPAPGNYLTATVQLRSRVVLEVPPGARLLGGTRQADYPPESRRWYVVLAENTTGAGI
TGGGEINGQGGAFVVRPSEVKNVMVSWNATGDCLGDECRPRLVGFIDSKDVRIHDITLNQ
PAYWCLHLVRCDNTVIHNVSIFGDFKTPNNDGIDIEDSNNTVITDCHIDTGDDAISPKST
TGPVYNLTATNCWMRTKSCAIKFGSASFFDFKRLVFDNITIVDSHRGLGMQIRDGGNVND
VVFSNIKMRTRYYHPLWWGRAEPIYITTCPRHPDSKEGAISDIRFINISSVSENGVFLAG
SKHGLLRNLKFKNIDLTYKRWTNYTGGLYDYRPGCQEMVKHKTGGMMLEHISGLEIDNVR
MRWARGSLKGWNVNPILFRPSTIDNLSFHDWQSVDVQ
>ZmPG41
MLEATAAASSPRAAAAAVAAGAAAAAVVSSPRRGGGSAHNHRRWAPAPFRACLLALWLLG

FALVFLWQSTSVGRVRLYTRPPMPKRAVASLGHWAASPPVYDLREFGAVGDGRTVNTAAF
ESAIAAIAERGGGRLTVPAGRWLTAPFNLTSHMTLFLAAGAEILGIQDERYWPLMSPLPS
YGYGREHKGPRYGSLIHGQDLKHVTITGHNGTINGQGQSWWVKFRRKLLNHTRGPLVQLM
RSSNIIISNITLRDSPFWTLHTYDCKNVTISETTILAPIAGAPNTDGIDPDSCENVVIKN
CYISVGDDGIAIKSGWDQYGIAYGRPSANITIQNVVIRSMVSAGVSIGSEMSGGVSDVLV
ENVHVWDSRRGVRIKTAPGRGAYVTNVIYQNITFENVRVGIVIKTDYNEHPDEGFDPKAV
PTIGNISYTSIHGHRVRVPVRIQGSAQIPVKNVTFHDMSIGIVDKKHHVFQCSFVQGQVI
GYVFPVPCKNLDLYDERRGLVKQSTLQNISDIDYSF
>ZmPG17
MVDTGGGRLYQRRGAVAFVAANKALLAAAWVVGFALVFLWQSASMSFGSAGGGGYLRLLS
VPPPPPRPAPRLRPTAYNITDFGAVGDGRAVNTAAFERAVEAIAALAERGGGQLNVPPGR
WLTAPFNLTSHMTLFLAEGAEILGITDEKYWPLMPALPSYGYGRERKGPRFGSLIHGQNL
RDVVITGHNGSINGQGEVWWMKHRRRILNNTRPPLVQLMWSKDIIVANITLRNSPFWHLH
PYDCTNVTVSNVTIMSPVSGAPNTDGIDPDSCQDVLIENCYISVGDDAIAIKSGWDQYGI
AYGRPSSDILIRNVTARSLVSAGISIGSEMSGGVANVTVENVRIWESRRGVRIKTATGRG
GYIRNISYRNITFDNVRAGIVIKVDYNEHADDGYDRTAFPDITSISFKGIHGQGVRVPVR
AHGSDVIPIKDISFQDMSVGISYKKKHIFQCSYLEGRVIRPVFPKPCENLDVYDEQGQLV
KRAVALNSTELDYDI
>ZmPG50
MAYGDRVITFEDSEKESEYGYVRKDMKHWPLIAPLPSYGRGRDEPGARYSNFIGGSNLTD
VIITGKNGTINGKGQVWWDKFHAKELKFIRGHLLELLYSENIIISNVTFVNAPYWNLHPT
YCTGVTILAPVNSPNTDGIDPKSSSRVKIEDCYIVSGDDCVAVKSGWDEYGIRFNMPSQH
IVIQRLTSVSPTSAMIALSSEMSGGIRDVRAKDSVAINTESAIRVKTLGAIAGVLSRNEL
VAAALFTLAINHKQVDVFSFGIVLWEILTGEEPYANMHCGAII
>ZmPG24
MAYGDRVTTFEDSEKESEYGYVRKDMKHWPLIAPLSSYGRGRDEPGPRYNNFIGGSNLTD
VIITGKNGTINGQGQVWWDKFHAKELKFIRGHLLELLYSDNIIISNVTFVNAPYWNLHPT
YCTNVTISGVTIPAPVNSPNTDGIGPESSSRVKVEDCYIISSDDCVAVKSGWDEYDISEM
SDDICDVRAEDSVAINTESGIRVKTLGAIAGVLLSNELVAAALFTHAINHKQ
>ZmPG6
MAPGRSLRPAGTAAVLWAASLLLLATSCARAGGAYAGPGCRKHVARVTEYGAVGDGRTLN
TAAFARAVADLARRAPDGGAALVVPPGKWLTGPFNLTSCFTLYLDEGAEILASQDMKHWP
LIAPLPSYGRGRDEPGPRYSNFIGGSNLTDVIITGKNGTINGQGQVWWDKFHAKELKFTR
GHLLELLYSDNIIISNVTFVDAPYWNLHPTYCTNVTISGVTILAPVNSPNTDGIDPDSSS
RVKIEDCYIVSGDDCVAVKSGWDEYGIRFNMPSQHIVIRRLTCVSPTSAMIALGSEMSGG
IRDVRAEDSVAINTESAVRVKSGAGRGGFVRDIFVRGLSLHTMKWVFWMTGNYGQHPDNT
SDPNAMPEVTGINYSDVFAENVTMAGRMEGIPKDPYTGICISNVTARLAPDAKELQWNCT
NVKGVTSHVSPKPCPELAAEGKPCAFPEEELVIGPPELPKCSY
>ZmPG37
MAPRTLGLNAAPLLQILRSLCLLALAAAAAVSGRRHGASPARGSGQSMYLAPACRAHTAS
VADFGGVGDGTTSNTAAFRSAVDHLSQYSGEGGGGGMLYVPAGKWLTGPFNLTSHFTLYL
HQDAVILGSQDVGEWPIVDPLPSYGRGRDKVGGRFASLIGGSNLTDVVITGSNGTIDGQG
AMWWSKFHKNQLKYTRGYLIELMHSDTIYISNLTLLNSPAWNIHPVYSSNIVVQGITILA
PTNSPNTDGINPDSCSHVRIEDCYIVSGDDCVAIKSGWDEYGISYGMPSQHIVIRRLTCV

SPTSAVIALGSEMSGGIQDVRAEDITAINTESAVRIKTAVGRGAYVRDVFARRMTLTTMK
RVFWMTGDYKSHPDDKYDPNAVPVVANISYQDVVATGVYKEAARLQGIQGAPFRGICVAN
VTADLSKSRKYPWNCADIEGVSANVSPAPCDPLQGAHDGACPFPTDTLPIDQVTVQQCAY
DIPATN
>ZmPG44
MTLFLARGAVIRATQDTSSWPLIEPLPSYGRGRELPGGRYTSLIHGNGLQDVVITGENGT
IDGQGSAWWDMWKNRTLLYTRPHLLELMSSSDIIVSNVVFQDSPFWNIHPVYCSNVVIRN
VTILAPHDSPNTDGIDPDSSSNICIEDCYISTGDDSIAIKSGWDEYGIAYGRASSGITVR
RITGSSPFAGFAVGSETSGGVENVLAEHLNFFNSGFGIHVKTNTGRGGFIRNITVSDVTL
DNVRYGLRIVGDVGNHPDDSYNRSALPIVDALTVKNVQGQNVREAGLIKGIPNSAFSRIC
LSNVKFTGGAPVRPWKCEAVSGGALDVQPSPCTELTSTSGTSFCTNSL
>ZmPG46
MPPTLGLLLAAAAAVLLLLLPPPPSAGAQETCWSGPAPRRGAWMSVASFGARGDGQTLNT
GAFARAVARIARRRGARGGTLLYVPPGVWLTGPFNLTSHMTLFLARGAIVRATQDTSSWP
LIDPLPSYGRGRELPGGRYMSLIHGHGLQDVFITGENGTIDGQGGVWWDMWKKRTLPFTR
PHLLELMYSTDVVVSNLVFQDSPFWNIHPVYCSNVVIANLTVLAPHDSPNTDGIDLDSSS
NVCVEDCYISAGDDLISIKSGWDEYGVAFGRPSSGITVRRITGSGPFAGFAVGSETSGGV
EDVVAEHLSFSGVGVGVHVKTNSGRGGFIRNVTVSQVTLDGARYGLRIAGDVGGHPGASY
NASLLPVIDGVAVRNVWGRNVRQAGLIRGIRDSVFSNICLSNVKLYGIGSDSIGPWRCRA
VSGSALDVQPSPCAELASTSGTGFCT
>ZmPG16
MAAATSRGPASVAICALAVLHAMAALCDSATAAAATCAGLAPAKHRPEVISIADFGGVGD
GRTLNTWAFRKAVYRIQHQRRRGGTTLHVPAGTWLAGSFNLTSHMTLFLARGAVLKATQD
TRGWPLVEPLPSYGRGRELPGPRYASFIHGDGLRDVVITGDRGVIDGQGEVWWNMWRRRT
LEHTRPNLVEFMHSTGIHISNIVLKNSPFWNIHPVYCDNVVVTNMMILAPRDSPNTDGVD
PDSSSNVCIEDSYISTGDDLVAIKSGWDEYGIAYGRPSAGVTVRRVRGSSPFSGIAIGSE
ASGGVRDVLVEDCAIFDSGYGIHIKTNVGRGGYIRNVTVDGVRLTGVRSGVRIAGDVGDH
PDAHFSQLAVPTVDAVRISNVWGVNVQQPGSLEGIRASPFTRICLSNVKLFGWRSDAAWK
CRDVRGAALGVQPSPCAELATSFASARSSCS
>ZmPG38
MAARAKAAAVDDHDPMPPPLPLPWPRPAPLLVMLSLLATASYLALTRFPAALLPIAPRSR
PHDAPAHAPGDSCAGFYAGAGPAREVWASVEEFGAVGDGATLNTAAFRRAVAELGARAVG
GGGARLDVPPGRWLTGSFNLTSRFTLFLHRGAVILGSQDPEEWPLITPLPSYGRGRERLG
PRHISLIHGEGLNDVVITGSNGTIDGQGHMWWELWRNRTLNHTRGHLIELVNSTNVLISS
VTLSNSPFWTVHPVYCSNVVMKDLTILAPLDAPNTDGIDPDSSSEVCIEDCYIESGDDLV
AVKSGWDQYGISFGKPSTNIVIQRVSGTTPTCSGVGFGSEMSGGISNVLVRDLHVWNSAQ
AVRLKTDVGRGGYITNITIASVAMEKVKVPIRFSRGADDHPDDRYDPAALPRISNVLVSD
VVGVHLQRAPMLEAVPGAVYEGICFRNFSFRGIRRQQDSRWHCESVYGEAHDVFPAPCEE
FRRDGSSSWCGLL
>ZmPG19
MNGGRALIATAGDLVVTDGGATPTPMRLRCGFLVPVVLALVLAAALGPATAPAPARAQLR
RDHQAPPGPRPHSVTITEFGAVGDGRTLNTVPFQNAVFYVRSFADKGGAQLYVPRGRWLT
GSFNLTSHLTLYLEEGAVIVGAKDSSQWLIVEPLPSYGQGLDLPGPRHQSLISGYNLTDV
VITGNNGVIDGQGLVWWQWLRSHELNHSRPNLLEFLYSEDIVISNLTFLNSPAWSIHPVY

CRCSAINLALCVHNVTIKTSLDAPLTDGIVPDSCSNLCIEDSTISVSHEAISLKSGWDRY
GISFGRPTSDIRISRVDLLSSSGAALAFGSEMSGGISDIHVNHLRIHDSSKGISFKTSPG
RGGYIEDVVISEVQMENVHVGIEFTGNCSTHPDDSFDPSDLPAIDHVTMKNMAGTNISVA
GVLSGIEGAPFTAICLSNLNFSMAAGSGPSSWSCSDVSGYSEAVFPEPCTELRDPSSSSS
VCYSLASYSAIETA
>ZmPG54
MAPCWLQVALASMVAVLVLGLACAEAEPQCTRRRPPAPPRPHSVTITEFGAVGDGVTLNT
VSFQNAVFYLRSFADKGGAQLYIPRGRWLTGSFNLTSHLTLFLESGAVIVGTQDVSQWPV
VEPLPSYGRGMDLPGSRHRSLINGQNLVDVVITGNNGTIDGQGSTWWNWFRSNKLNYSRP
HLVEFVDSEQIVISNITFLNSPAWSIHPVYCSNVVVHSVTIQTSLDAPLNHGIVPDSCSN
MCIEDSSISVSHDAISLKSGWDKQGIAFGRPTSDIHISRLDLQSSLGAALAFGSEMSGGI
SDIHADRLLIHSSSRGVFFKTAPGRGGYIRDTVISDVQMEDVDVAIAFTGDWPSHPDDQF
DPAALPVVSHITLKNMTGTRISVAGVLSGIAGDPFTDICLSNINFSLADSASPTSHWSCS
NVSGYSELVFPEPCLDLQTQSSDASVCSTLPSFHAAAIAAA
>ZmPG1
MKRSASLFQVLLVFTAVIEIQWSTVSSIYCKDMPPNVYRPHSVTITEFGAVGDGVTLNTK
AFQNAIFYLNSFADKGGAQLFVPAGRWLTGSFHLISHLTLSLDKDAIIIGSPDSSHWPVI
DPLPSYGRGRDLPGKRHQSLIFGLNLTDVIITGANGSIDGQGAIWWGWFHNHTLNYTRPH
LVELMYSTNVVISNLTFKNSPFWNIHPVYCSQVLVRHVTILAPLNSPNTDGVTPDSSTNV
CINHCYVRNGGDVIVIKSGWDEYGISFAQPSSNISISDITGETRGGSGIAFGSEMSGGIS
EVRAVGLRIVNSLHGIRIKTAPGRGGYVENVYIADVSMDNVSMAIRITGNYGEHPDDKYD
STALPVISNITIKDVVGVNIGVAGILEGIQGDNFSNICLSNVSLSVQSAHPWNCSLIEGY
SNSVIPESCEQLRSNCRQTSICYDGSSSSVMSVQQPRHTSPTSRLLDLFTGVGFILV
>ZmPG42
MLNCSRKMGRSASVLQVLLVSATIVVAQTQWASGVWGMYCEDLTASVERPHRASVTDFGA
VGDGATLNTKAFQNALFHLDSFAKKGGAQLFVPAGRWLTGSFSLISHLTLSLDKDAVILG
SPDSSDWPVIDALPSYGRGRELPGKRHQSLIFGSNLTDVIITGANGTVDGQGAVWWDWFH
NHTLNYTRPPLVELMYSTRVVISNLTFINSPFWNIHPVYCSQVLVQHLTILAPISSPNTD
GIDPDSSTNVCIEDCYVRNGDDIIVIKSGWDEYGISFAHPSSNISIRNITGQTRNSAGLA
FGSEMSGGISDVRAEGVRIVNSVHGIRIKTAPGRGGYVKNVYVADVSFDNVSIAIRITGN
YGEHPDDGYDRNALPTISNITIKDVVGVNIGVAGMLQGIPGDSFSGICLSNVSLSVRSTD
PWNCSLVEGYSSSVSPEVCEQLRATPGPGSGQMCYGGNYPAAAAQPQPPQKSGASRLVLG
WLVLVRYGCRHITWFLLANELPGLFNKG
>ZmPG7
MEPAAGRRARCLLLPLLVGALALALAGAATATAWPHPHGGAAGLGHGAGAAAAGAGRPGG
AERWYRDLALRRMESVRSSFGARRDLATASASARVYHVTDYGADPTGAADATAAISKAIA
DAFRPPTNATMTGGIPDLGGAEVHLDGGTYLIKGPLTLPASGGGNFKIHSGSLRASDDFP
TDRYLIELSAAKSGRSYDYEYATLRDLMLDCSYRGGGVAVVDSLRVAVDNCYVAHFASDG
VAVRGGHETLIRNTYLGQHMTAGGDPGERGFTGTAIRLDGNDNSVSDVVIFSAATGIMVT
APANSISGVHCYNKATGFGGTGIHLKIPGLTQAWISNCYMDYTSIVAEDPVLLHVSGSFF
LGDANVVLKAVSGVARGVQVVGNIFSGRDKGVDIVQLDGAFATVDQVYVQQNSATGMTVR
STSARASLEGNGTSWTLDFSPVLLFPDRIGHVQYSLVAGDEFPGHTLRNVSGNQVVVATD
KAVSATVHVLVDQNSD
>Os07g10740

MACNVLLFSAFVVVMSFFLCSVHAKVNMNASSLTNGDDSLRGKSSLESKKAVFDVRKHGSY
GDGQHDDTKALSKAWAAACSSLQPSIVLVPKGKRYLTKHITLSGPCKSSITFMIEGTLVAPPKR
SDWSKETIRHWIMFNGVIGLTVAGGGTVDGNGKIWWQNSCKTNAKLACTESPTALTFYSCSN
LKVENLKLLNSQQIHMSVEDCTNVRISGLTITAPGTSPNTDGIHITRSKNVQVTGCTIKTGDDC
MSIEDGTENLHVKNMVCGPGHGISIGSLGDHNSEAHVNNVTIGTVRLYGTTNGARIKTWQGG
RGYAKYIVFQNMIMENVWNPVIIDQNYCDSATPCKKQLSICVNNMEAYNLVFLSQTSAVQISN
VVFKNIRGTSASKEAIKLDCSRNVPCQGITLNDVKLTVKGGGGDAKSTCRNAKWKKSGTVVP
QPCASTTTV
>Os07g10730
MTNKENIGKHFILKVENLKVVNSQQIQISVEDCTDVKMSRLSITAPETAPNTDGIHITRSRDVQ
VTDCTIKTGDDCMSIEDGTKNLHVKNMVCGPGHGISIGSLGDHNSEAHVNNVTVDNVRLYGT
TNGARIKTWQGGKGSAKNIVFQNMVMDNVWNPIIIDQNYCDSSTPCKQQKSAVEVSNLLFKN
IRGTSASEEAIVLHCSNSVPCHGITLENVNLTVKGGSSNAKSTCQNAEWKKSGVSVHCPVVS
KIDLELVGTSD
>Os07g10680
MACNVLIFSAYVVVMSFFLCSVHAKVNMNASFHLTSGDDSLRGRSLESKKVVFDVRKHGAY
GDGQHDDTKALAKAWAAACSSSQPSIVLIPKGKRYLTKHITLSGPCKSSITFMIEGTLVAPPKR
SDWSKETIRHWIMFNGVSGLTVAGGGTVDENGKIWWQNSCKTNAKLPCTEAPTALTFYSCS
NLKVENLKLLNSQQIHMSVEDCTDVRISSLTITAPGTSPNTDGIHITRSKNVQVTGCIIKTGDDC
MSIEDGTENLHVKNMVCGPGHGISIGSLGDHNSEAHVNNVTVDTVRLYGTTNGARIKTWQG
GWGYAKNIVFQNMIMENVWNPIIIDQNYCDSATPCKEQTSAVQVSNVVFKNIRGTSASKEAIK
LDCSRNVPCQGITLKDVKLTIKGGGSDAKSTCGNAKWKKSGIALCFQ
>Os07g10700
MASTVLIVSASLLALFFFLHSADADVGSNVFSIQSYGAHGDGRHDDTKALGDTWAAACSSAK
PAVLLIPKGKKYLIKHTTLSGPCKSSISLMVKGSLVASPERSDWSKETIRHWILISGVTGLTVTG
GGTIDGNGKIWWQNSCKTNSKLPCTEAPTALTFYSCKNLKVEYLKVVNSQQIQISVEDCTDV
MVSRLSITAPETAPNTDGIHITRSRDVEVTDCMIKTGDDCMSIEDGTENLHVKNMVCGPGHGI
SIGSLGDHNSEAHVNNVTVDNVRLYGTANGARIKTWQGGKGSAKNIVFQNMVMDNVWNPIII
DQNYCDSSTPCKQQKSAVEVSNVLFKNIRGTSASEEAIMLHCSSSVPCHGITLENVNLTVKG
GIDLKHEHPWTLEDVEK
>Os06g35320
MGFVRALFLLAMVCVAAHAKDYPKEEGAKAEGPAAASGGGGGSTHDVVKLGGKGDGKTDS
TKAVNEAWTAACAGTGKQTIVVPKGDFLTGPLNFTGPCKGDIVIQLDGNLLGSTDLALFKSNW
IEIMRLESLEISGKGKLDGQGAAVWSKNSCAKKYDCKILPNTLVLDFVNNGLISGISLVNPKFF
HMNVFKSKNITIKDVTITAPGDSPNTDGIHMGDSSKISIIDTVIGTGDDCISIGPGTEGVNISGVT
CGPGHGISVGSLGRYKDEKDVTDVTVKNCVLKKSTNGVRIKSYEDAASVLTASKFTYENIKME
DVANPIIIDMKYCPNKICTANGNSKVTIKDITFKNITGTSSTPEAVSLLCSDKLPCTGVTLNDIKV
EYSGTNNKTMAVCKNAKGTATGCLKELSCF
>Os06g35370
MGFVRALFLLAMVCVAAHAKDYPKEEGAKAEGPAAASGGGGGSTHDVVKLGGKGDGKTDS
TKAVNEAWTAACAGTGKQTIVVPKGDFLTGPLNFTGPCKGDIVIQLDGNLLGSTDLALFKSNW
IEIMRLESLEISGKGKLDGQGAAVWSKNSCAKKYDCKILPNTLVLDFVNNGLISGISLVNPKFF
HMNVFKSKNITIKDVTITAPGDSPNTDGIHMGDSSKISIIDTVIGTGDDCISIGPGTEGVNISGVT
CGPGHGISVGSLGRYKDEKDVTDVTVKNCVLKKSTNGVRIKSYEDAASVLTASKFTYENIKME

DVANPIIIDMKYCPNKICTANGNSKVTIKDITFKNITGTSSTPEAVSLLCSDKLPCTGVTLNDIKV
EYSGTNNKTMAVCKNAKGTATGCLKELSCF
>Os03g59330
MASVRALLALTFLLSGGAATAAAAMVRNGGSPSIYGGGGGEGAAVIGRGGRSLLQAAAAAAA
TTQSAVFSLDSYGAHGDGERDDTAALARAWSAACASAAPAVVLVPESRSYLLRQVTLSGPC
ESTIKLMVKGTLVASPDMSNWNESNRRYWIVVRGVDGLAVGGGGTIDGNGEGWWENSCKI
NRALPCKGAPTALSFHTCDNLSVNGLKMVNSQQIHMSVEDCTGVELAHLSISAPGTSPNTDG
IHITHSKNVQVSDCTIKTGDDCVSIEDGTHGLHVTRLVCGPGHGISIGSLGDDNSRAEVSDIFI
DTVHLYGTTNGARIKTWQGGSGYAKDIVFQNMVMNSVKNPIIIDQNYCDSAKKCETQEGSAV
EISNVVFKNIAGTTISKSAITLNCSKNYPCYDISLQDINLEMVDDNGATGSTCQNAKWRKSGTV
VPQPCTSTN
>Os01g33300
MAVTMDNVIRFMFILSVVYGAAYAKKSEAKVASAPGLAAAVNTVFDITELGAVADGKTDSTKAV
QDAWDAACGLAGSQKVVIPKGEFMTGPLNFSGPCKGYVTVQIDGTMFGSNDIPKYNKGNWI
EILHIDNVLVNGSGTLDGQGAAVWKDECKILPNTLVLDYVKNGTVSGLKLVNAKFFHINVYMS
KGVTIKNVTITAVANSPNTDGVHIGDSSEISVSDATIATGDDCISVGPGSSRISIQGITCGPGQGI
SVGCLGRFKDEKDVTDVTVRDCVLRNTSNGVRIKSYEDVLSPITASRLTFENIRMDGVANPVI
VDQKYCPEKDCPEKKGSKTVTIKNVTFRNITGTSNTPEAVSLLCSDQLPCSGMELLDVNLKY
DGKDNKTMAVCTNAKGISKGSLQALACL
>Os06g40890
MAFRNHDAAMVLFFFLLMVTTYANAHGHSKKPEEITQGVYGAAAAVAAGPGGTFDITKLGAV
GNGRADSTGAVMAAWRSACAGAGKQTILIPKGDFMTGAMELRGPCNGAVTIQLDGNLLGSN
DLSKYPGKKMPNWVEVRHVDNFVISGKGKLDGQGPGVWSKNSCAKNYNCKLLPNTLVLNT
VNDGVVSGITLLNAKFFHMNIYRCKDIKISGVTINAPGDSPNTDGIHMGDSSKITIAATTIGTGD
DCISIGPGTDGVNITGVTCGPGHGISIGSLGRYKDERDVRDVSVTRCVLRKTTNGLRIKSYED
SVSPVTVSKVSYDGVVMDHVDNPIIIDQKYCPNSICTSKGDSKVSVRDVTFRNITGSSNTPAV
VQLLCSGKLPCSGVAMQDVRVLYGGSDKKTTAVCDHALGKSTGCLKELACL
>Os11g14410
MSVITVVVVVGVVLALAAAASVMAAEYSVVDYGARAGGRADAAGAFLAAWAAACGDDGER
PVMRVPAGTFLVGRAYFRGPCRSAGGVVLAIDGTVVAPPAVGNASWITFHYAHGLAIRGGTL
DGNGHAFWACKAAAGRRCPPGTTTLDISQSNNVSVKRVTLVDSKNVHVSIFDCAGVTLQGV
RIAAPADSPNTDGIHVALSRDVAVLSATVRTGDDCVSVGPGTSGVAIRNIRCGPGHGISIGSLG
GRAGEGEVRNVTVESASLAGTQNGLRIKTWGKPFAGRVSGVRFANVAMRDVQNPIVVDQN
YCPGNVNCPGQSSGVKISDVEYEGITGTSATAVAVRFDCSGSNPCTGIRLRNINLTYDGGGG
KPARSFCKNAGGSASGVVIPPSCL
>Os02g10300
MVSSTRRTTSSLSATTAAAIAAAAAALMVSVAFATAQYTPATPAAPGAAAAGAAAGATPAAPYT
PATPGAAGAAPSVPAGPLDIAQLGAKGDGTSDSTAFVLQAWKNACNATGTQKIVIPPGNYLTG
ALNLKGPCTSSIILRLDGNLLGTGDLNAYKTNWIEVEHVDNFAINGHGIIDGQGPLVWTHNQC
NKNYNCKILPNSLVIDYSTNVTVRGITLKNSKFFHLNIYESKNVVIDKVTITSPGDSPNTDGIHV
GDSTNITISSTTIAAGDDCISIGPGTKMVRVNGVRCGPGHGISVGSLGRYKDEKDVEDIIVTNC
TIKGTTNGLRIKSYEDSKSQLRATKFLYDGITMDNVSYPIIIDQKYCPNNICSASGTSKVAVTDIV
FKNIVGTSATPEAVTLNCANNLPCQGIQLHNVDLKYAGQGNTTLSVCKNVAGKSSNVAKELAC
V

>Os05g46510
MAILRVSVMRLVVAAMAVAAGVLFFSGAGEARVLLTLDDFGAVGDGITNDTQAFLDAWNAAC
ASTEPAVLAVPAGKTYQIWPVRLAGPCKKKLKLMISGTIAAPASPDEWAGRDPTKWLYVFRVD
DLSVSGGGTIDGMGAEWWARSCKRKKTKPCSTVSAPKALQFEECRRVSVQGITMQNGPQF
HLMFTRCTDVKASFLRVVAPESSPNTDGIHLNDTTHAQIMDNLISTGDDCVSMVGNCSDVRV
KDISCGPGHGISIGSLGKNRTTDRIENVRVDTCLLTNTTNGVRIKSWQGGMGYAHNLRFEGIV
MKNVSNPIIIDQYYCDQPTPCANQTQAVEVRKIEFAGIRGTSATEQAIKLACSDAVPCRDLELR
NVNLTMVGGGAASAFCHRASGKAAGAVVPASCLAKAPHRMLGDATPAARVGS
>Os03g11760
MGPRKLHLASISALSIFLYVFAHTNSAQAFPVADGTYPPEAEGPAAESSDMDEQLELTPGPQP
RVVDVDDYRARADAGDHTEAFLQAWKEACNSSDYPSVLLVPEGKTYLLMPVSFNGPCRATT
ITATIRGTLEAPSNRSVWLDRDLQEWITFDNIDHLRVLGGGTLNGNGHQWWINSCKTNRSMR
CVTGPTALYFRRCNHLVVEGLQIRDSMQMHVVIAYSWRVLVSRLLITAPGWSPNTDGIHVSNS
REVLMSGCIISTGDDCISIVTGSMFIRATGIFCGPGHGISIGSLGANKSWAHVSDVLVEKATLVG
TTNGVRIKTWQGGDGHAERITFQDITMHNVTNPVIIDQNYCDSMTPCHEQGSAVAINNIRYRN
IRGTSSSKVAINFVCSNSVHCDGIVMQDVSLVGEGSYVSCSYMNARVVELGYNFPYCRAEM
>Os01g19170
MELAAAGRTAAIALLLALAFASSFISAADGARSARHHHAKHAKRNAAHPPSQAPGPAARHAP
GPARHHGAPAPHPGRRSPPAPAPANPPSSDPMPGGAPSAAPAAGAATVYDIVKDFGAAGDG
VTDDTDALKTAWDTACADDGAGVVLAAAGRSFLIHTTVFTGPCQGSVTLQVDGTIVAPSEPAT
WPANNKRNWLVFYRADGVSLVGAGLIDGKGQKWWDLPCKPHKGGNTHGPCDSPVAMRFAI
SNNVTVRGLKVQNSPEFHFRFDNCNGVRVDGLSISSPALSPNTDGIHVENTSDVLITNTVVSN
GDDCVSIGAGTLNVHIENVTCGPGHGISIGSLGKAGTKACVANVTVRNAVIRHSDNGVRIKTW
QGGSGSVSAVAFENVRMDAVRNPIIIDQYYCLSKSCENETTAVFVNGVSYSGIRGTYDVRGP
PIHFGCSDAVPCTNITLSDVELLPASGDTVDEPFCWNVYGNAATPTVPPVSCLSSGFPNYTEK
KDLQCY
>Os02g03750
MAKTASGGGGDDGGAGGDHDHDHEQFLKLWNGRGGADAKEDYLNWDDDDDDDEDEDEE
EEEAEQVMAWAAKCRPPAGRNVVNVDSFGAAGDGCSDDTEAFLNAWKKACSLNNAVFLVP
GGRRYKVGAARFIGPCKNRMIIQIQGTIVAPDEPSEWDPASPRLWLLFSGLAGARIQGGGLID
GSGSKWWANSCKIDRSKPCKGAPTALTIDSCRGVSVRNLRLQNAQQMHLTVSRSRDVRLAS
VRVDSPEDSPNTDGIHVADSTAVTIQSCRIATGDDCISISNGSFAVRMRDIDCGPGHGISIGSL
GQGGAFAAVDGVSLDGARVARAQNGVRIKTWQGGAGYVRNVRFAGVRVDGVDHPIVIDQF
YCDATRPCRNRTSNVRVSGVVFRNITGTARRAEAIRLACSDAVPCVGIVLSDIDLRREDGGGE
VQTVCNCAMGFDDGRVSPAADCLRTSPCGGMSPDDYHPDDKDDDDEVRHTEL
>Os08g23790
MEARLRLLVVVVVVAGHCAAVASAAGNSSVVGYHGDPTFNVRNYGAKGNGQTDDSKALMT
AWKAACAATGAVTLVLPPGTYYIGPVQFHGPCSKATTMTFLMQGTLKAATDLKRFGNDWVEF
GWVNHLIVSGQNGAAFDGQGAASWPFNKCPIRKDCKVLPTSVLFVNNKNMVVQNVASVNS
KFFHMALLQCSGAKISGVKISAPESSPNTDGIHIERSNGVSIADTTIATGDDCISIGQGNDNIDV
ARVHCGPGHGMSVGSLGRYVGEGDVTRIHVRDMTFHGTMNGVRIKTWENSPTKSNAAHML
FENLVMNDVQNPIIIDQKYCPYYNCEHKFVSGVTIKDVQFKNIKGTATTQVAVLLKCGVPCQGV
VLQDVDLRYKGNGVSSSKCENVRAKYAGFQNPKPCP
>Os06g31270

MTTILKVFTLHLFIMLHGVHGHIYDVTEYGAEPSNIDNKDAFLAAWRAACGSAAGNATLLIPEG
TFAVSTVEFSGPCKNGRSPLAVVVDGVLHPCAGGCHRKSGDDDVWITFSGVSNLLVTGAGT
LDGRGGEHGHSNGGGKSKTTTTLELDSVANATVRGLRFLNSRGFHLNLHRSSHVAAERLRIE
APAASRNTDGIHVGLSSHVTVADSLVGTGDDCVSIGPGSSGVVIAGVACGPGHGISVGSLGR
EEGEGDVRGLVVRNCTVVGTTNGLRIKTWPGSPPSRAFNITFRDIVMSNVSNPIIIDQHYCPH
AHCSDIAKPSLVQISDVTYERIEGTSSSRVAVQLLCSEDRPCSGVRFDRVNLSCGRERCGSK
FSNVEGTKPTLVAADEAAAFGPGAVPPPDQDADVVESQH
>Os06g40880
MTPLLQITMSSLIITSWWWLQATCTARQLVFDVRDFGAVADGQTDNSKAFERAWAKACAAPG
RAAVVVPAAGGGGGGGGGYLLHPVVFRGPCKGFVEVRVAGVVRAPAGLDAFRGYHEWINF
AGIDGLLVTGGGTFDGRGASSWHLNDCPWKPDCVPPPSSIKLGSVRNATITGVTSLDSKFFH
VTIVGSHDVEVSHVSIRAPRDSPNTDGVHIQGSTGVRITDTAVATGDDCVSVGPGSADVTVS
GVSCGPGHGISVGSLGRSPGEADVRRLRVSNCTIAGTANGVRIKTWRGGQRSSAAAAAAA
VSGLVFEDIVMRRVRNPIIIDQEYCPYLSCHHQSERRPSVVRISDVKFRNIRGVSATQVAVKLS
CSAASPCRGVELRDIDLRYVRRGVATVSRCANVAGGVAGGTLVPPPCI
>Os01g07790
MATIPEGGSLIMEGDIPGTGLEKIPLIPQKRSELLAHSNSTVNLLEMSRNGRIIAINDTGNPVVP
QRIWVEAKYTFVLLLVLTRSSEAQKRPLYNVLDFNATGDGNTDDTKAFLAAWEETCNNEGWP
ILIIPGGRTFLLKQIKFNGSCKSPIKIQMDGNIVAPNYIWASEADNLITFYSVNNLTLDGNGQIDG
KGAIWWTCYTEKKCVYRPVILAFAACNNLSVTNIHLTNSADKHMTVYRCSQVHVHNVTIVAPG
DSPNTDGITMAISDHVYISNCSIQSGDDCVSMLSYTTDVNITDITCGPGHGISVGSLGRFETAL
VERITVSNCNFIGTKNGVRIKSWQGGMGQATGFIFENINMTAVESPIIIDQFYCPQGNCPLKDG
GVAISDARFINIRGTSSEQEAIKILCSQSVHCQGIYLSNINLSWENHTALANATILNANGTVEGS
VVPKVVFS
>Os01g43490
MSVASSDPISDNLVVFSRMARCSVSRVVLLLLCCMHLSAAAAGAAVYNVESYDARPDGRTD
ASRALASAWSAACRSPEPATVYVPDGEFFVSHSAFAGPCSGGRMTVQIDGTLVAPSGYTGS
ASSGGEWIVFDHVDGLTVSGGTLDGRGESLWACKAAGHGGCPDGATSMKVLNSRDVVISG
VKSVNSELYHVVIDGCEGVAVQDARIVAPGSSPNTDGIHVQSSSAVTITGASIQTGDDCISVGP
GTSNLRVEHVSCGPGHGISIGSLGKESEEGGVENVTVSGAAFVGTENGLRIKTWGRAARSG
AYVRGVVFEHALMRDVSNPIIIDQSYCPNDGGQGCPHQSSDVQISGVTYTDIQGSSASQVAV
KFDCSASKPCSGLGLQDIKLTFDGGKPAEATCQHADGTASGVLMPPSCL
>Os05g20020
MGGGPSRLGLPRLLMVVALVLLPLCGFGVHGRNHIHKKPHGGGGGGGRQHRGGGTVVSSP
AVPPADEQTQPPGIVPSDPVIPAQPEQCVFDVRAFGAVGDGTTDDTEAFRAAWRAACAVESA
VISVPSDGTFTITTTTFTGPCKPGLVFQVDGVLMPPDGPDCWPPSDNRRQWLVFSNLDGLTL
RGAGTIEGNGEGWWNLPCKPHRGPNGSTLRGPCDSPTLVRFFMSRNLVVEGLRVENSPEF
HFRFDGCSDVRVDGLSIRSPANSPNTDGIHVENTQRVAIYNSMISNGDDCISIGTGSYDVDIQ
NVSCGPGHGISIGSLGVHNSQACVANVTVRNAVIRNSDNGLRIKTWQGGMGSVSGINFDTVS
MENVRNCIIIDQYYCLDKRCMNQSTAVHVTDVSYANVRGSYDVRAAPIHFACSDTVPCTNITM
SEVELLPFSGELVDDPFCWSAYGLQQTPTIPPIYCLQDGLPDSLLDNPDLRCR
>Os01g44970
MKLRVKGLGLLLLLVLLALCSTIDVCDARRGKHWRPRSSPSSSLLRNKGKGKKGSSNRQHG
SNRPSPKPPLSPPPSPGNGKGYQSPYQPSPSPSPNAPVSPSPVNGSGHASPKSPTPSCGK

GNQPPSRPTPTSPQGAVFNVVDFGAKGDGVSDDTKAFEAAWAAACKQGASTVVVPSELEFL
VGPISFSGPYCKPNILFQLDGTIVAPTSAKAWGSGLLQWIEFTKLNGVSIQGNGIINGRGQQW
WTYSDIDDDEDDDTQYDVEFERMPQVKPTALRFYGSFNVVVAGITIVNSSQCHLKFDSCQGV
MVHDVTISSPENSLNTDGIHLQNSKDVSIHHTNLACGDDCVSIQTGCSNINIHNVNCGPGHGI
SIGGLGRDNTKACVSNVTVRDVNMFRTMTGVRIKTWQGGLGLVQDVRFSNIQVSEVQTPIIID
QFYCDKRTCSNQTSAVAVSGVQYENIRGTFTIKPVHFACSDSSPCSGITLTGVQLRPVQIPHY
RLNDPFCWQAFGELYTPTVPPIACLHLGKPAGNNLQSYHDLC
>Os01g66710
MAKAMLSLLVHLHAALLFVPEPAGAGAVYSVVRYGARGDGASDSTRPFLRAWADACRSPRP
ATVYVPPGRYLLGRATFVGPCSSRAVAFSIAGTVVAPAGYAWDGATAGQWITFESVVGLTVS
GGTLDGRGDALWACKKQQPRGHCPTGASSLTISNARNVVVEGVRSVSSELFHVVVLQSRG
VTVRRVTVEAPADSPNTDGIHIHKSTNVAVYDAAIRTGDDCVSVGPGNSNLWIERVACGPGH
GISIGSLGKQQGMAVEAVQNVTVKTTWFTGTTNGLRIKTWGNSKRGFVRGVTFSDSTMAGV
GNPIIIDQHYCPDGGCGGAARGSSSGIKISEVEYADVRGSSATPVAVSFDCSRSNPCSGIRLR
DVRLTYQGKSGRLQAAGAVSSCRNAQGTASGLVVPPSCL
>Os06g35300
MKLGGKGDGKTDSTKAVDEAWKAACAGTGKQTIVFPKGDFVTGPFNFTGPCKGDIVIQLDG
NLLGSTDLALFKVNWMEIKRVDNLEFSGKGKIDGQGAAVWSKNTCAKKYICKILPNSLVLDFV
NNGLVSGISLVNPKFFHMNMFKCKNITIKDLTITAPEDSPNTDDIHICDSSKISIIDTVIGTGDDCI
SIGPGTEGINISGVTCGPGYGISVGSLGRYKDEKDVTDVTVKNCVLKKSTNGVRIKSYEDAAS
VLTTSKFTYKNIKMEDVANPIIIDMKYCPNKICTANGNSKVTIKDITSRISPAPPPRPRPLASSAQ
TSCRAPASL
>Os05g46520
MPPSSRMAPPPLAGVVVVAAALLLLLPEAAEPRTLLSLDDFGAVGDGVANDTQALVDAWSAA
CATGDHTFLHVPAAKSYLVWPVTLAGPCREEIKLFISGNIVAPESPDEWPEGGGGGGEWLHF
VGVSDLTLSGGGVIDGRGHRWWARSCKAKHNATENCTTQAAPKALHFEDCQGISVMGITLQ
NSQESHLTFTRCSHVKANYLRITSPEDSPDTTGVHVVSSRNVHIMDDSISTGHDCVSIVGNST
DVRLRAISCGPGHGISIGGLGENRSYHRVEKIKMDTLFISNTENGVRVKTFQGGCGTARKMK
FGDILMKNVKNPIVIDQQNSSSNEIPCGSKNGSAVTVGEISYTDITGTSASERAVTFACSEAAP
CSKLSLENVNITMAGGQNASAYCHHAFGKSVGVVVPDSCLGKEDYLRRQVPASAAAAGGGT
QEKGGEDDDR
>Os01g45060
MASISRMETPQLRVLRLLNRADVATTLVKWLGRIRTLYMAIRPEARRPGPQHSILADPTTGPC
LGLPTGMLGRPGMTDPARHDGSLDGTIVAPTSAKAWDSGLLQWIEFTKLNGVSIQGNGIING
RGQQWWTYSDTDDDENDDTQYDVEFERMPQVKPTALRFYGSFNVVVAGITIVNSSQCHLKF
DSCQGVMVHDVTISSPENSLNTDGIHLQNSKDVSIHHTNLACGDDCVSIQTGCSNINIHNVNC
GPGHGISIGGLGRDNTKACVSNVTVRDVNMFRTMTGVRIKTWQGGLGLVQDVRFSNIQVSE
VQTPIIIDQFYCDERTCSNQTSAVAVSGVQYENIRGTFTIKPVHFACSDSSPCSGITLTGVQLR
PVQISHYRLNNPFCWQAFGELYTPTVPPIACLHLGKPAGNNLQSYHDLC
>Os06g28670
MMRRPRSLNASTAGSFPFLLVLVVTSSFLSGRCLATTDSHHRRPPAGRHGPPRPVSPPSPPA
TTFSVLQYGAVGDGDKDDTKASAECRHTHIHSRLILHTLIVMCKFMAFVHAWSAACAVRSST
VVVPAGYRFVVGPVTFTGDSCQPNTVFQLDGTIVANTDSGAWCSGNAVQQWLEFRSCTGLT
IQGSGTVDGQGSHWWSGGAPATDIDADRVGTNNRPTALRVYESTNVAVTGITIQNSARFHLT

FDTCRAVEVRGVAIRSPGDSPNTDGIHLAGSVGVSIQNATVACGDDCVSIQDGCSRVLVRGV
TCGPGHGISIGGLGKGGAMAVVSDVTVQDVSLVGTSAGVRIKTWQGGSGSVRGVLFSGVRV
SAVKTPIVIDQYYCDHATCANQTAAVAVSGVAYSGITGTYTQRPVYLACSDAAPCAGLRLEDIK
LAPVKEGGYGRLYGPFCWKAYGDEVRPVVPPVDCLMAGEP
>Os01g22590
MGPTASAAARRSTRQPSTMTTTMSRRTAAAVAVSVVLLLLSSCLPCCSEARLHYHRRQHRR
APHRGHHRAAAAAAAATNGGSHISQPPAALPPDFDSGESPAETPGLPPAGVEDAPPRRSPR
EKPCPTMQPPVKPPEELSPVGAPRSRVRAMPPSPSLSPPAKAPSHSHAKTPSMPPAERPAL
PPTKAPAAISPATPPQLSPANAHSTHHHAKPPSLPPAEPPVPSPSPEHPPRHSPSKPPAYAPA
KPPTALRPAIPPAAMPKPPSVAPVQPPQRPPAPATKPPPSFPPQLAPTMPPPAHAPAETPAPP
TTPPALPPATTAPSPKNSSSSPPPPCTGGGGGISNVFDVRAFGATGNGSSADGDTRAFRAA
WKAACSAESATVLVPSDGVFTITSTIFAGPCKPGLTFQIDGVLMPPDGPASWPAADGRRQWI
VFYRADGMTLSGKGTIEGNGEEWWNLPCKPHRGPNGSTLPGPCESPALIKFVASSDVSVQG
LRMENSPQFHLKFDGCSRVLVDGLVVSSPASSPNTDGVHVENTSSVRILNSRISNGDDCVSI
GGGCSGVRVENVTCVHGHGISIGGLGARGARACVSNVTVRGARVVDSDNGVRIKTWQGGA
GSVSGVVFDAVQMVNVRGCIVIDQYYCDAHGGAGAGCANQTAAVRVDGVAYRGIRGTYNPR
GGGGAPVRFACSDTVACTGITMTDVELLPAGGGDEGGGASAGAKLADPYCWNAYGVMETL
TQPPVHCLQEGRPESLQDQLASC
>Os11g14400
MPRRAHAHAAVAAHLLVSAFVAAAAAAAATYNVIDYGAVGDDGGVTDSARAFEAAWAAACA
GDAAAAAATVVVPAGGVYLVSRARFAGPCRSGAVAVNMTGATVVAPVPYAGVQLWIVFQDV
DGVSVAGGTLDGRGRALWACRRARRPDCPPATRSLTIYRSRNVAVRGLTSRDSAGIHITVQA
SAGVAIVDTVVSAPGRSPNTDGIHIKQSTGVTVRNAVIGTGDDCVSMVEGSSDVLIEAVTCGP
GHGISIGSLGDTPEQVAVRNITVKGAALAGTTNGLRIKTWAKANAGAVAGVSFSGVVMRNGS
GIEISGVSYTDIEGTSATATAVRFDCSPSRPCAGIAMRDVRLRYQPPAAAAEEEQPAASFCRN
AHGVAFGDVDPPSCLTE
>Os05g50260
MGLGIKGLTFLLLLVLLVLCSNVSLSDARSGKHWRQNRASSSTLLRRKGKGKTNNSHKQYGK
GNQDPYQPSPSTSPNVPVNPSERPVQGKGHPAPTMPPPSSGSGHTLPSPPPPLPPLLPPP
QPPAAQSQNTVFNVVDFGARGDGVTDDTQAFEEAWAAACKVEASTVLVPSELEFVVGPISF
SGPYCKPNILFQLDGTILAQTSTRVWGSGLLQWLEFTKLSGISIQGSGVINGRGQEWWTYSD
PNDDDNDDVDAYNVELEKMPQIKPTALRFYGSSNVTVTGITIVNSSQCHLKFDSCQGVMVHD
LTISSPENSPNTDGIHLQNSKQVSIHHSNLACGNALINSIKAKPTGFRTKGKLKTLVQVSEVIFA
LCDAGDDCVSIQTGCSDINIHNVNCGPGHGISIGGLGRYNTKACVSNVTVRDVNMFKTMTGV
RIKTWQGGSGLVQGIRFSNIQVSEVQTPIIIDQFYCDRTTCRNQTSAVAVLGVQYENIRGTFTIK
PAHFACSDSSPCSEITLTGIQLKPLIVPQYHLYNPFCWQAFGELSTPTIPPISCLQIGKPSGNNV
MSDYDLC
>Os01g36830
MRRRCFSPAGMLIAGAILCLVASTAPAPALSAGRTTFSVSSFGAAGNGIADDSEALVKAWKFA
CRIPRSTVLLPSGHRFLISPVTLQGPCNTRLTLQIDGDVLAPPGMGYWPKARRPLQWLNFKW
LDGFTIQGTGTVDGQSTLLRSVSPANVSQHWYVSGVKPTLIRFYSSFNVSVRNIRITNSPQCH
LKFDSSGGIKVKNITISSPGDSLNTDGIHLQNTRDVDIRSSSIGCGDDCISIQTGCSNVHMKNIN
CNPGHGISLGGLGKDNSLACVSDVFAEHINVENALYGVRIKTWQGGKGTVRNVTFSNVRVAN
VATPIAIDQFYCDAGGGGARCGNRSDAVGITGVAYRRVAGTYTYQPVRLACSDARPCTGVSM

ADVRLSPASATGAGGLRQPLCWKSYGEAMGMIEPTGIACLQRSNGFVMPLTKPFNYTC
>Os09g26800
MGETGVAVASSPRAAASSPRAAASSASVASSPRAGGVGGRHHHRRWGGAAAISPSYRAVLL
ALWLVGFALVFLWQSTSVGRARLYTRPPLLPKRAPSAQGMGQWVAAPPVYDLREFGGVGD
GRTLNTEAFVAAVASIAERGGGRLVVPAGRWLTAPFNLTSRMTLFLAAGAEILGVQDERYWPL
MSPLPSYGYGREHRGPRYGSLIHGQDLKDVTITGQNGTINGQGQSWWSKFRKKVLNHTRG
PLVQLMRSSNITISNITLRDSPFWTLHIYDCKDVTISDTTILAPIVGAPNTDGIDPDSCENVVIKN
CYISVGDDGIAIKSGWDQYGIAYGRPSTNIIIHNVTIRSMVSAGVSIGSEMSGGVSNVLVENVHI
WDSRRGVRIKTAPGRGAYVSNITYRNITLEHIRVGIVIKTDYNEHPDEGFDPKAVPIIENISYSSI
HGHGVRVPVRIQGSAEIPVKNVTFHDMSVGLVDRKNHVFQCSFVQGQVIGYVFPVPCKNLDL
YNERRELVKQSALQNISDIDYSF
>Os03g61800
MRRSASLLRVLLVFITMVGTQWSNVSSTYCKDMASSVYRPHSVTITEFGAVGDGVTLNTKAF
QNAIFYLNSFADKGGAQLFVPAGRWLTGSFSLISHLTLSLDKDAEIIGSPDSSDWPVIDPLPSY
GRGRELPGKRHQSLIFGTNLTDVIITGANGTIDGQGAIWWDWFHSNTLNYTRPHLVELMYST
DVVISNLTFKNSPFWNIHPVYCSQVLVQHVTILAPLNSPNTDGIDPDSSTNVCIDHCYVRNGD
DVIVIKSGWDEYGISFARPSTNISISNITGETRGGAGIAFGSEMSGGISEVRAEGLRIVNSMHGI
RIKTAPGRGGYVKNVYISDVSMDNVSMAIRITGNFGEHPDDKYDRNALPMISNITIENVVGVN
VGVAGILEGIEGDNFSSICLSNVSLSVQSMHPWNCSLIEGYSNSVIPESCEQLRTDCGQTPIC
YDGGSSSAIHAQAARHRLSSASRLLNPLLKLAML
>Os08g01600
MLPHATAHQPTPSPRPRLLPVAAAALLLASSYLALTRLPAAAPLAALVAPATARVDGDRDRDS
CAGFYRGGSSGRRAASASVEEFGAVGDGVTSNTAAFRRAVAALEARAGGGGARLEVPPGR
WVTGSFNLTSRFTLFLHHGAIILGSQDPEEWPLIAPLPSYGRGRERLGPRHISLIHGEGLDDV
VITGNNGTIDGQGRIWWDLWWNRTLNHTRGHLIELVDSTNIMISNITLRNSPFWTVHPVYCRN
VVIRNLTVLAPLNAPNTDGIDPDSSSEVCIEDCYIESGDDLVAVKSGWDQYGISVGKPSSNIIIQ
RVSGTTPTCSGVGFGSEMSGGISNVIIRDLHVWNSAQAVRIKTDVGRGGYITNITIENVRMEK
VKVPIRFSRGADDHSDDKYDRSALPKISDVRIRDVVGVDLQRAPMLEAVHGAVYEGICFRNV
SLTVIKRQDRWHCESVYGEAHDVLPAPCEEFRRNGSSSWCGHS
>Os02g15690
MAALHGHGFAVTVLLVVLLVQCDAAEATTCAGAVPARPRPETVSIAEFGGVGDGRTVNTWAF
HKAVYRIQHQRRRGGTTLLVPAGTWLTGSFNLTSHMTLFLARGAVLKATQETRSWPLAEPLP
SYGRGRELPGARYTSFIHGDGLRDIVITGDKGIIDGQGDVWWNMWRQRTLQHTRPNLLEFM
HSSGIHISNIVLKNSPFWNIHPVYCDNVVITNMMIIAPHDSPNTDGVDPDSSTNVCIEDSYISTG
DDLVAIKSGWDEYGIAYGRPSSGITIRRVRGSSPFSGIAIGSEASGGVSNVLVEDCSIFNSGYG
IHIKTNIGRGGFIRNITVDNVRMNSVRNGLRIAGDVGDHPDEHFSQLALPTVDAVSIKNVWGV
NVQQPGSIEGIRNSPFTRICLANVKLFGWRNNAAWKCRDVHGAALGVQPGPCAELTTSLSS
GFCSY
>Os02g54030
MVETSGGGGRLHPHGQRRSVAAFLAANKTLLAAAWVIGFTLVFLWQSAKVSVGGGGGGGG
FLRLRSAPPPPPSRPAPLLRPKAYELTDFGGVGDGRAVNTEAFERAVKAIAALAERGGGQLN
VPPGRWLTAPFNLTSHMTLFLAEGSEILGIPSFREVQKLVLLSGFDRDERYWPLMPALPSYGY
GRERKGPRFGSLIHGQNLKDVVITGHNGSINGQGEVWWMKHRRRILNNTRPPLLQLMWSK
DIIVANITLKNSPFWHFHPYDCTNITVSNVTILAPISSAPNTDGIDPDSCQDVLIENCYISVGDDA

IAVKSGWDQYGIAYGRPSRNIVIRNVMARSLVSAGISIGSEMSGGIANVTVEDVRIWESRRGL
RIKTAIGRGGYIRDISYRNITFDNVRAGIVIKVDYNEHADDGYDRDAFPDITNISFKEIHGRGVR
VPVRAHGSSDIPIKDISFQDMSIGISYKKKHIFQCSFIEGRVIGSVFPKPCENLDLYNEQGQLVK
RAAMWKLHGYMTRSSGVKAVNKRSIMKQGWTELEEVVNFLF
>Os07g14160
MMSRSASALQVLLVFATVFAATQWATSSAMYCNDLTASVHRPHSVSITEFGAVNDGVTLNTK
AFKNAIFYLSSFADKGGAELFVPAGRWLTGSFNLISHLTVSLDADAVIIGSQDSSDWPVIDPLP
SYGRGRELPGKRHQSLIFGSNLTDVIITGANGTIDGQGELWWNWFHNHTLNYTRPPLLELMY
SDRVVISNLTFMNAPFWNIHPVYCSQVLVQHLTILAPISSPNTDGIDPDSSSNVCIEDCYIRNG
DDIVVIKSGWDEYGISFAHPSSNISIRNITGQTRNSAGIAFGSEMSGGISDVRAEGLRFINSVH
GIRIKTAPGRGGYVKNIYIADVSMDNVSIAIRITGNYGEHPDDNYDKNALPVISNITIKNVVGVNI
GTAGMLLGIQGDIFSNICLSNVSLSSKSADPWNCSLIEGFSNSVAPEICEQLRPSPGPGQVCY
DGNSYPVPAAQQPYKSGATRLQNPFLKFISLYS
>Os05g50960
MVASLRLSLAAAAAAALAVAVALLLPPLAAAQGETTCPADVPPRGAWMSVASFGGVGDGRAL
NTAAFARAVARIERRRARGGALLYVPAGVWLTGPFNLTSHMTLFLARGAVIRATQDTSSWPLI
DPLPSYGRGRELPGGRYMSLIHGDGLQDVFITGENGTIDGQGSVWWDMWRKRTLPFTRPH
LLELISSTDVIISNVVFQDSPFWNIHPVYCSNVVITNVTVLAPHDSPNTDGIDPDSSSNVCIEDS
YISTGDDLISIKSGWDEYGIAFGRPSSGITIRRITGSGPFAGFAVGSETSGGVENVHVEHLNFF
GMGVGIHVKTNSGRGGFIRNITVSEVTLNGARYGLRIAGDVGGHPDASYDPSKLPVVDGVTI
KNVWGQNIRQAGLVRGIRDSVFSRICLSNVKLYGGDSVGPWKCRAVSGGALDVQPSPCAEL
TSTSEMSFCTN
>Os01g43160
MGRQRWWLLLAVAAAAAVVVGAGAQETCSDMVPAAPRRGAWMSLASFGGGGGDGRTLST
AAFQAAVASIERRRAPGGALLYVPPGVWLTGPFNLTSHMTLFLARGAVIRATQDTSSWPLIEP
LPSYGRGRELPGGRYMSLIHGNGLQDVVITGDNGTIDGQGSAWWDMWKKGTLPFTRPHLL
ELMNSSDVVVSNVVFQDSPFWNIHPVYCSNVVIRNVTVLAPHDSPNTDGIDPDSSSNVCIED
CYISTGDDLIAIKSGWDEYGMAYGRPSSHITIRRITGSSPFAGFAVGSETSGGVEHVLAEHLNF
FSSGFGIHIKTNTGRGGFIRNVTVSDVTLDSVRYGLRIAGDVGGHPDDRYDRNALPVVDGLTI
KNVQGQNIREAGSIKGIATSAFSRICLSNVKLNGGAAVRPWKCEAVSGAALDVQPSPCTELTS
TSGMSFCTNSL
>Os03g03350
MAKKTILQVVCAVALVALCACGVAASPSSPAGAGCRKHVARITEYGGVGDGRRSNTAAFAKA
VADLSLRAGDGGAALVVPKGKWLTGPFNLTSHFTLFLDHGAEILASQNLEDWPLIAPLPSYGR
GRDEPGPRYSNFIAGSNLTDVIITGRNGTINGQGQVWWDKFHAKELTYTRGYLLELLYSNNIII
SNVTFVDSPSWNLHPTYCTNVTISGITILAPLNSPNTDGIDPDSSSHVKIEDSYIVSGDDCIAVK
SGWDQYGIKFNMPSQHILIRRLTCISPTSAMIALGSEMSGGIRDVRAVDNVAIDTESAVRIKSG
VGRGGYVKDVFVRGLSLHTMKWVFWMTGNYGQHPDNSSDPNALPEVTGINYSDVFAENVT
MAGRMEGIPNDPYTGICMSNVTAQLAPDAKKLQWNCTDVKGVASDVSPVPCPELGAAGKP
CAFPEEELVIGPPELPTCSY
>Os12g36810
MAGLVVLVVAMAAVAVAVGVASGEEEAAAARCARRPRPRPHSVTISEFGAVGDGVTVNTLPF
QNAIFYLRSFADKGGAQLYVPRGRWLTGSFNLTSHLTIFLEKDAVIIGAKEVSEWPIVEPLPSY
GQGIDLPGARHRSLINGHNVTDVVITGNNGIIDGQGLTWWNWFRSNKLNYSRPHLVEFVDSE

DIVISNLTLLNSPAWGIHPVFCSNVMVHDVTIRTSLDAPLTDGIVPDSCSNMCIEDSSISVAHDA
ISLKSGWDNYGITIGRPASDIHISRVDLQASLGAALAFGSEMSGGISDIHVDHLNIHGSSRGILF
KTAPGRGGYIRDVVISDVQMEDVNVAIKFTGDWSTHPDNHFDPSALPMINRITLKNMVGTNIS
VAGVLSGINGDPFTNICLSNISFSLADSTQSSSWSCSNISGYSELVFPEPCPDLHHSSSNSSIC
FSLLTYHALAAA
>Os11g43750
MKRRLVVVALVLLAVVELVHGDGWRWAPGSRPHSVTITEFGAVGDGKTLNTLPFQNAVFYAR
SFADKGGAQLYVPKGRWLTGSFNLTSHLTLFLEEEAVIIGTKDPSQWPIVEPLPSYGQGLDLP
GPRHRSLINGYNLSDVVITGNNGVIDGQGSVWWDWLHSHELNHSRPHIVEFLHSEEVVISNL
TFLNSPAWSIHPVYCSNVKVHNVTIKTSLDAPLTDGIVPDSCSNVCIEDSSISVGHDAISLKSG
WDNYGISFGRPTSDIHISRVDLQASSGAALAIGSEMSGGISDIHVDHIRIGSSSKGISFRTTPG
RGGYIAEVVVADVVMDSVHLAIEFTGNWSSHPDDHFDPSFLPVIDQITLKNMEGTNISVAGVL
SGIEGDPFSAICLSNLNFSIADLAPSSAWTCSNVHGYSELVFPKPCSELHDTSTNSSICFSLSS
YSALAVL
>Os09g31270
MASPLLLLLPLAVSSLLLPFAAAARVFSVADYGAAGDGARYDTGAIQAAVDACAAAGGGRVLL
PAPGDYLTATVHLRSRVVLDVAPGARLLGGTRQADYPPESRRWYVVLAENTTGAGVTGGGE
INGQGGAFVVTPNPQKNIMVSWNATGDCEGDECRPRLVGFIDSKDVTIHDITLNQPAYWCLHI
VRCDNTMIHNVSIYGDFDTPNNDGIDIEDSNNTAITHCHIDTGDDAICPKSTTGPVYNLTATNC
WIRTKSCAIKFGSASFFDFKKLVFDNITIVDSHRGLGMQIRDGGNVSDVVFSNIKMSTRYYHPL
WWGRAEPIYITTCPRHPDSKEGTISDIQFINISSVSENGVFLAGSKHGLLRNLKFKNVDLTYKR
WTNYSGGLYDYRPGCQKMVKHRTGGMMLEHISGLEIDNVRMRWSRGSLKGWDVDPLLFQ
PSTVDKLSFHDWQSLAVSR
>Os04g52320
MKNMEAARRGRRGRQGGGLLLVVLAALLLLLLALARGASAWAHGGLAGGAGAGAGERRYM
DLAMRRMESVRSSFVARRELATSTAASSSRVYHVTDYGADPTGGADATAAINSAIADAFRRP
SNATMTGGIPDLGGAEIHLDGGSYLLKGPLSLPASGGGNLKIHSGSLRAADDFPTDRYLIELS
AKAAGGGGGSSPAMSYYYEYVTLRDLMLDCNYRGGGVRVVDSLRVGVDNCYVVHFATDGV
AVSGGHETFVRNTFLGQHMTAGGDPGERSFTGTGIRLDGNDNSVSDVVVFSAATGIMVTGG
ANAISGVHCYNKATGFGGAGIYLKVPGLTQTWITNCYMDYTGIVAEDPVLLHVSGSFFLGDAN
VVLKAVNGVARGVQIVGNLFNGRGKGVDIVELDGEFATVEQVYVAQNAATGMTVRSTTARAA
AEGNGSSWTVDFSPVLLFPDRIGHVQYSLAAGDAFPGHALRNVSGNRVVIATDAAVSATVHV
LVDQNSS
>AT1G02460
MLTSTYNRNQVLGFMTLILTLMSLSEARNHHHKEKHKHNNHNHHSSKPKPPSSSISQPPTPP
PGPPDSPAPSLPPSPSDDPADDNNGIYNVRKYGAVGDGETDDTEAFKTAWDSSCNNENNTD
SVLLVPYGYTFMIQSTIFTGPCRSYQFFQVDGTIVTPDGPESWPSNISKRQWLVFYRVNGMA
LKGEGVIDGRGQKWWDLPCKPHRSVNKSAIVTGPCDSPIALRFFMSSNLRVEGLQIKNSPQF
HFRFDGCQGVHVESLHITAPPLSPNTDGIHIENSNSVTIYNSIISNGDDCVSIGSGSYDVDIRNL
TCGPGGHGISIGSLGNHNSRACVSNITVRDSVIKYSDNGVRIKTWQGGSGSVSGVTFNNIHV
DSVRNPIIIDQYYCMTKDCANKTSAVFVSDIAYQGIKGTYDIRSPPMHFGCSDAVPCTNLTLSD
IELLPAKGEIVLDPFCWNAYGIAEELSIPPVWCLMSDPPKGLQGSLVDKCGSS
>AT1G02790
MANARSLVAKANNINVGSLILMALVFGSCVANGEYLGGRRGLAANSGNPTVYDITKFGAVGD

GSTNTFKAFLNTWIQVCDSPVPATLLVPKGTFLAGPVIFAGPCKSKVTVNVIGTIIATTSGYATP
EWFLFERVDNLVLTGTGTFHGKGEAVWKADGCGKKVQCNLPPTSLKFRNMKNVEINGISSV
NAKAFHMFLVKTENVNIQNIKLTAPAESPNTDGIHLSNADNVSILDSTIATGDDCVSVGRGSNN
VTVERVICGPGHGLSVGSLGKYKNEEDVSGIHVNNCTMIETDNGLRIKTWGGSDPSKAVDIK
FENIIMQSVKNPIIIDQNYGSRGGDSQVAISDILFKNIRGTTITKDVVQIMCSKSVPCQGVNVVD
VNLDYVGKTGGEKKSSSGGLVGALCDNANVIFGGKLSFPMCPK
>AT1G05650
MTKSAITFPLIFTLLTFIDVSSSASIVFNVVSFGAKPDGVTDSTAAFLKAWQGACGSAASATVV
VPTGTFLLKVITFGGPCKSKITFQVTGTVVAPEDYRTFGNSGSWILFNKVNRFSLVGGTFDAR
GSGFWSCRKSGQNCPPGVRSISFNSAKDVIISGVKSMNSQVSHMTLNGCTNVAVRNIRLVAP
GDSPNTDGFTVQFSTGVTLTGSTVQTGDDCVAIGQGTRNFLISKLACGPGHGVSIGSLAKQL
NEDGVENVTVSSSVFTGSQNGVRIKSWARPSTGFVRNVFFQNLIMRNVQNPIIIDQNYCPSN
QGCPTEHSGVKITQVTYKNIQGTSATQEAMKLVCSKSNPCTGITLQDIKLTYNKGTPATSLCFN
AVGKNLGVIQPTSCLNR
>AT1G05660
MTKSVIRFSLLFTLLTFIDVSISASNVFNVVSFGAKPDGVTDSTGAFLKAWQGACVSASSATVV
VPKGTFLLKVITFGGPCKSKITFQVAGTVIAPEDYRTFGNSGFWILFNKVNRFSLVGGTFDARA
NGFWSCRKSGQNCPPGVRSISFNSAKDVIISGVKSMNSQVTHMTLNGCTNVVVRNVKLVAP
GNSPNTDGFHVQHSTGVTFTGSTVQTGDDCVAIGPGTRNLLITKLACGPGHGVSIGSLAKEL
KEDGVENVTVSSSVFTGSQNGVRIKSWARPSNGFVRTVFFQDLVMKNVENPIIIDQNYCPTH
EGCPNEYSGVKISQVTYKNIQGTSATQEAMKLVCSKSSPCTGITLQDIKLTYNKGTPATSFCFN
AVGKSLGVIQPTSCLNR
>AT1G10640
MIMRRSLSLRSITMMILMAVLVWSVTLETCIARRGRHWRHSHRSSSDLSDSLSNKKPKSHGN
SHHHSSHNNNHHHKSKPKPKPKLKTPPKAHDNNSPVVSGPPKVQPPPLPPQKGSQVFNVM
DFGAKGDGKCDDTKAFEAAWVAACKIEASVMLVPPEYTYLVGPISFSGPYCQANIVFQLDGTI
IAPTDSKTWGKGLMWWIDFTKLKGIKVQGKGVIDGRGSGWWQQDSPFIDSDTKLIVPLNNSA
NQNPPMPIRSELDERMPSIKPTALRFSGSFGVEVTGITIQNSPQCHLKFDDCVGVVVHDIAVS
SPGDSPNTDGIHLQNTKDVLIHSTTLACGDDCISIQTGCSNVFVHNVNCGPGHGISIGSLGKE
GTKACVSNITVRDVAMHNTMTGVRIKTWQGGVGSVKGIIFSNIQLNQVQIPITINQFYCDHSK
CKNQTSAVAVEGVTYERIKGTYTVKPVHFACSDNFPCVDVQLSSIELKPVQEKYRMYDAYCW
QTFGELNTPTLPPIDCLKIGKPPRNKVQSDHDVC
>AT1G17150
MAPCIDFVFMAFGLLLLLIAVTTSRPITRPKVFDVRSYGAKGDGKMDNTNAFTNAWKDACRW
NGPSKMYIPLGTFYLGGVTFVGPCDGKISFVIDGTLLAPPNNDDIKKEIWINFRYINYLTVFGD
GTLDGQGKKSWSLIDCQKDNNCPKLAINMGFDFVKNSSMNGITSLNSKAGHFNFLSVDHFSI
TRVNIIAPSNSPNTDGIKIALSSNMQISNTHISTGDDCIAMLSGNTNFDIYNVKCGPGHGISIGS
LGKNKDEKNVNGLMVRNSVFTGTTNGIRIKTWESSASTIRIINLVYENLQMINVENPIGIDQKY
CPYPPCSNMGDSHIQIRNVTLKNIWGTSKNKVAVKFQCSKTFPCKDVQLIDINLTHHGVDGPA
SALCENVDGSATGKMVPPHCLS
>AT1G19170
MVENLFPSRFQFHLQRLDPKRRLTSLLASHKTLFTFLWITAFGSVFLWQRTAYIEGGSGTGPV
GGKFTIFGKIKPIQPIPRLRPVVFDLKDFGGVGDGFTLNTEAFERAVISISKLGGSSGGGQLNV
PPGRWLTAPFNLTSHMTLFLAEDSEILGVEDEKYWPLMPPLPSYGYGRERPGPRYGSLIHGQ

NLKDIVITGHNGTINGQGQSWWKKHQRRLLNYTRGPLVQIMWSSDIVIANITMRDSPFWTLH
PYDCKNVTIRNVTILAPVTGAPNTDGIDPDSCEDMVIEDCYISTGDDAIAIKSGWDQFGIAYGR
PSTNILIRNLVVRSVISAGVSIGSEMSGGISNVTIENLLIWNSRRGIRIKTAPGRGGYIRNITYKNL
TLDNVRVGIVIKTDYNEHADDNYDRKAYPILSGFSFAGIHGQGVRVPVRIHGSEQIPVRNVTFR
DMSVGLTYKKKHIFQCSFVKGRVFGSIFPRPCENFDVYDEEGRLVKPAAESNVADIDYDI
>AT1G23460
MMDKLFILSLLGLLLVTAYGAAGKMVYTDLDILDELENFDVLVDDDDDTKLLDWPSFTSRHSG
KNLVNVDTFGAAGDGVSDDTQAFVSAWSKACSTSKSVFLVPEGRRYLVNATKFNGPCEQKLI
IQIDGTIVAPDEPSNWDSKFQRIWLEFSKLKGVVFQGKGVIDGSGSKWWAASCKKNKSNPCK
SAPTALTIESSSGVKVSGLTIQNSQQMNFIIARSDSVRVSKVMVSSPGDSPNTDGIHITGSTNV
ILQDCKIGTGDDCVSIVNASSNIKMKNIYCGPGHGISIGSLGKDNTTGIVTQVVLDTALLRETTN
GLRIKTYQGGSGYVQGIRFTNVEMQDVANPILIDQFYCDSPTTCQNQTSAVKISQIMYRNITGT
TKSAKAIKFACSDTVPCSHIVLNNVNLEGNDGQVEAYCNSAEGFGYGVIHPSADCLYSHDDK
GLDQTHKSEEAETGHDEL
>AT1G43080
MVSIASTFKALCLSFLFIAVASRPTIRPKVFNVRRYGSRPDGKTDNANAFTSVWKRACTRISG
SSKIYVPKGTFYLGGVEFVGPCKNPIEFVIDGTLLAPANPRDIKQDTWINFRYINNLSISGSGTL
DGQGKYSWPLNDCHKNTNCPKLAMTMGFAFVNNSRIKDITSLNSKMGHFNFFSVHRFNITG
VTITAPGDSPNTDGIKMGSCSNIHISNTNIGTGDDCIAILSGTTNLDISNIKCGPGHGISVGSLG
KNKDEKDVKHLTVRDTVFNGTSDGIRIKTWESSASKIVVSNFIYENIQMIDVGKPINIDQKYCP
HPPCEHEKKGESHVQIQDIKLKNIYGTSNNIVAVNLQCSKSFPCKNVELIDINLKHTGLEKGHS
TAMCENVDGSVRSKMVPQHCLD
>AT1G43090
MVSIASTFKALCLSFLFIVVASRPTIRPKVFNVRRYGSRPDGKTDNANAFTSVWKHACTRISG
SSKIYVPKGTFYLGGVEFVGPCKNPIEFVIDGTLLAPANPRDIKQDTWINFRYINNLSISGSGTL
DGQGKYSWPLNDCHKNTNCPKLAMTMGFAFVNNSRIKDITSLNSKMGHFNFFSVHRFNITG
VTITAPGDSPNTDGIKMGSCSNIHISNTNIGTGDDCIAILSGTTNLDISNVKCGPGHGISVGSLG
KNKDEKDVKHLTVRDTVFNGTSDGIRIKTWESSASKLVVSNFIYENIQMIDVGKPINIDQKYCP
HPPCEHEKKGKSHVQIQDIKLKNIYGTSNNIVAVNLQCSKSFPCKNVELIDINLKHTGLEKGHS
TAMCENVDGSVRGKNGSSTLSRLTQWSIEKITFWVIHDIFSYILLPNFCIYFMFYVLVYMIS
>AT1G43100
MVSIASTFKALCLSFLFIAVASRPTIRPKVFNVRRYGSRPDGKTDNANAFTSVWKRACTRISG
SSKIYVPKGKFYLGGVEFVGPYKNPIEFVIDGTLLAPANPRDIKQDTWINFRYINNLSISGSGTL
DGQGKYSWPLNDCHKNTNCPELAMTMGFAFVNNSRIKDITSLNSKMGHFNFFSVHRFNITG
VTITAPGDSPNTDGIKMGSCSNIHISNTNIGTGDDCIAILSGTTNLDISNVKCGPGHGISVGSLG
KNKDEKDVKHLTVRDTVFNGTSDGIRIKTWESSASKLVVSNFIYENIQMIDVGKPINIDQKYCP
HPPCEHEKKGKSHVQIQDIKLKNIYGTSNNIVAVNLQCSKSFPCKNVELIDINLKHTGLEKGHS
TAMCENVDGSVRGKNGSSTLSRLTQWSIEKITFWVIHDIFSYILLPNFCIYFMFYVLVYMIS
>AT1G48100
MRRLSGILVIYIISTVLFGHFTNVARARYHYHKGRHGVTHPLPPPPPPPLETANPPDQVPSDPY
PSPDPAPGDSDSGCVFDVTSFGAVGDGSCDDTAAFQDAWKAACAVESGVVLAPEGGVFKIT
STIFSGPCKPGLVFQLDGVLMPPDGPEEWPEKDNKNQWLVFYRLDGFTFSGKGTVEGNGQ
KWWDLPCKPHRGPDGSSSSGPCASPTMIRFFMSNNIEVKGLRIQNSPQFHMKFDGCQGVLI
NEIQISSPKLSPNTDGIHLGNTRSVGIYNSVVSNGDDCISIGTGCSDVDIQGVTCGPSHGISIG

SLGVHNSQACVSNITVRNTVIRDSDNGLRVKTWQGGTGSVSNLLFENIQMENVLNCIIVDQY
YCQSKDCRNETSAVKVFDVEYRNIKGTYDVRSPPIHFACSDTVACTNITMSEVELLPEEGELV
DDPFCWNAYGKQETLTIPPIDCLLDGSPVVEEAYDSNYGC
>AT1G56710
MSLPNSLFYTFFLILFSTIRIAQSIYLSPSPAPNPAYNDNDNIAPTVFDVTSFGAIGDCSTDDTSA
FKMAWDAACMSTGPKSALLLVPYTFCFLVKPTTFNGPCRTNLVLQIDGFIVSPDGPRSWPSN
YQRQWMMFYRVNGLSIQGSGVINGRGQKWWNLPCKPHKGLNGTTQTGPCDSPVAIRLFQS
SKVRIQGINFMNSAQFHVRFDNCSDVVVDSVIIKAPASSPNTDGIHIENTHNVQIRNSMISNGD
DCISIGAGCFNVDIKNVTCGPSHGISIGSLGVHNSQAYVSNITVTNSTIWNSDNGVRIKTWQG
GSGSVSRIVFSNILMVNVRNPIMIDQYYCQTNNCANQTSAVIISDVLYANIKGTYDLRSPPIHFG
CSDSVPCTNLTLTEVDLFPSKGQHLENPFCWNAYGSMKITVPPVYCLLDAPPDF
>AT1G60590
MITRESLTLRSITMMTLVTILVWSATLETCIARRGRHWRHHHRSSSSLSDSLSSKKPKSHENN
HHHMSHKSKPKPKMKTQPPKSNDGSPVVSQPPQVQQPPPPHVQPPPTPLPLQPVEDSQQF
NVLDFGAKGDGMSDDTQAFEAAWASACKVEASTMIIPPDYIFLVGPISFSGPYCQANIVFQLE
GMIVAPTDTESWGGGLMWWIEFTKLSGITIQGNGVIDGRGTVWWQQDYLSDYPIDDDFKLIV
PLNNSVQERPPMPIRSELNWRMPSIKPTALRFYGSIDVTVTGITIQNSPQCHLKFDNCVKVLV
HDVNVSSPGDSPNTDGIHLQNTRDVMIHTTTLACGDDCISIQTGCSNVYVHNVNCGPGHGISI
GSLGKDSTKACVSNITVRDVVMHNTMTGVRIKTWQGGIGSVKGILFSNIQLTEVQLPIVIDQFY
CDHSKCMNHTSAVSVEGVTYEKIRGTYTVKPVHFACSDSFPCIDVQLSGIELKPVQLQYHMY
DPFCWKTFGELNSATVPPIDCLQIGKPARNGVHSDHDICL
>AT1G65570
MALFLSFVQVFSIVITIIMSHFGQFDARTSLNVLSFGANPNGIVESAKAFSDAWDAACGVEDS
VVIYVPKGRYLVSGEVRFEGESCKSREITLRIDGTLIGPQDYSLLGKKENWFSFSGVHNVTVL
GGSFDAKGSTLWSCKANGYNCPEGATTLRFMDSNNVKIKGVLSLNSQLFHIAINRCRNIKIED
VRIIAPDESPNTDGIHIQLSTDIEVRNASIKTGDDCISIGPGTKNLMVDGITCGPGHGISIGSLAK
SIEEQGVENVTVKNAVFVRTDNGLRIKSWPRHSNGFVERVRFLGAIMVNVSYPILIDQNYCPG
DSSCPSQESGIKINDVIYSGIMGTSATEIAIKMDCSEKVPCTGIRMQAINLTSYGEAAKTSCTNV
SGKQLGLVTPSGCL
>AT1G70500
MIKPFIISLFALLMVASYGATGKMLFKELDILGKLENLDVPEDDIEDDVTFFDFSSFTSQYSGKN
LVNVDSFNASGDGVSDDTQAFIRAWTMACSAPNSVLLVPQGRSYLVNATKFDGPCQEKLIIQI
DGTIIAPDEPSQWDPKFPRNWLQFSKLQGVVFQGNGVIDGSGTKWWAASCKKNKSNPCVG
APTALTIYSSSNVYVRGLTIRNSQQMHLIIQRSTTVRISRVMVTSPGDSPNTDGIHITASTDVVV
QDSKISTGDDCVSIVNGSAKIKMKRIYCGPGHGISIGSLGQGHSKGTVTAVVLETAFLKNTTNG
LRIKTWQGGNGYVKGVRFENVVMQDVANPIIIDQFYCDSPSTCQNQTSAVHISEIMYRNITGT
TKSSKAINFKCSDAVPCSHIVLNNINLEGNDGKVEAYCNSAEGFGYGVVHPSADCLYSHDDK
SLNQTDQYLAETIMVEKETENAHDEL
>AT1G78400
MVSTISGFSLILFIAAVASSISAAPSAALVGRKVFDVRSYGARGDGKTDNTMAFTKAWKDACQ
WKGLPRVYIPFGTFYLGAVAFTGPCKSRISFIIKGTLLAPKDPNAIKQDSWIIFRYVDYLTVSGG
GILDGQGSYSWPLNNCRQTHNCRALPMNMGFQFVRFSRLTRIKSINSKMGHLNFFSVQHFDI
TRVNIKAPGDSPNTDGIKIGSSNHMKIHHVDIATGDDCIAILSGTFNLDINKVNCGPGHGISVGS
LGKFKGEKSVQGLIVRNSIFNGTSNGVRIKTWPSPGEPNLVSNFLFKNLQMIDVQSPINIDQR

YCPNPPCSFQSFSKIQIRDVKFQNIWGTSTAKEAVKLQCSKNVPCKNVQLFNINIVHRGRDGP
ATSVCENVGGWIGGKISPPSCIR
>AT1G80140
MGSVNVPAGKVFLLNSLHFTGPCIPKPLLFIIDGEMIAQSDPQKWGNGENGVIPWLIFDQVDG
LAIVGRGLLDGQGKSWWDIHCRDHPGPMMTFSNCGNVTLKSLRFRNSAQTHVLVMGSQNV
YIDDIKITSPEASPNTDGIHITSSTAVSINHSDIATGDDCVSIGDQVNNLNVTFMNCGPGHGVSI
GSLGRGGTEVTVENIRVSHVNFTGTTNGARIKTWPGGTGYVRGIEFFDIRFSSVQNPIIIDQFY
GCAPTCVETMKGVHIEKVRYMKMSGTSATKVAMKLECSGESVPCSNLFMRDIDLSPADGIDS
VSSLCSFAHGSAQGIIRPLSCF
>AT1G80170
MSYSRGGTLVTLLLLLVVASSLALTANANSFESLLQLPRRQSRSTRPRSERLLHVGNFGAKGN
GVTDDTKAFADAWKTACSSKVKTRILVPENYTCLLRPIDLSGPCKARLTLQISGTIIAPNDPDV
WEGLNRRKWLYFHGLSRLTVEGGGTVNGMGQEWWERSCKHNHSNPCRGAPTALTFHKCK
NMRVENLNVIDSQQMHIALTSCRRVTISGLKVIAPATSPNTDGIHISVSRGIVIDNTTVSTGDDCI
SIVKNSTQISISNIICGPGHGISIGSLGKSKSWEEVRDITVDTAIISDTANGVRIKTWQGGSGLVS
KIIFRNIKMNNVSNPIIIDQYYCDSRKPCANQTSAISIENISFVHVRGTSASKEAIKISCSDSSPC
RNILLQDIDLEPSNGDGFTESFCWEAYGSSSGQVYPPPCLSDDTSFLEQSVQSGITSAYL
>AT2G15450
MTCISFAFNALCFCLFFIVVASRSTVRPKVFNVQRHGAKPDGKTDNANAFTSIWSRACKRISG
SSKIYVPKGTFYLGGVEFVGPCKNPIEFIIDGTLLAPANPSDIKQDTWINFRYINNLSISGSGTL
DGQGKQSWPHNDCHTNPNCPKLAMTMGFAFVNNSNIKDITSLNSKMGHFNFFSVHHFNITG
VTITAPGDSPNTDGIKMGSCSNIQISDTNIGTGDDCIAILSGTTNLNISNVNCGPGHGISVGSLG
KNKDEKDVKDLIVRDVIFNGTSDGIRIKNWESSASKILVSNFVYENIQMIDVGKPINIDQKYCPH
PPCEHERKGESHVQIQNLKLKNIYGTSKNKVAVNLQCSKIFPCKNVELIDINIKQNGVKDGSST
SVCENVDGFARGKMFPPHCLN
>AT2G15460
MACISFAFKALCFCLFFIVVASRSTVRPKVFNVQRHGAKPDGKTDNANAFTSIWSRACKRISG
SSKIYVPKGTFYLGGVEFVGPCKNPIEFIIDGTLLAPANPSDIKQDTWINFRYINNLSISGSGTL
DGQGKQSWPHNDCHTNPNCPKLAMTMGFAFVNNSNIKDITSLNSKMGHFNFFSVHHFNITG
VTITAPGDSPNTDGIKMGSCSNIQISDTNIGTGDDCIAILSGTTNLNISNVNCGPGHGISVGSLG
KSKDEKDVKDLIVRDVIFNGTSDGIRIKTWESSASKILVSNFVYENIQMIDVGKPINIDQKYCPH
PPCEHERKGESHVQIQNLKLKNIYGTSKNKVAVNLQCSKIFPCKNVELIDINIKQNGVKDGSST
SVCENVDGFARGKMFPRIV
>AT2G15470
MACISFAFKALCFCLFFIVVASRSTVRPKVFNVQRHGAKPDGKTDNANAFTSIWSRACKRISG
SSKIYVPKGTFYLGGVEFVGLCKNPIEFIIDGTLLAPANPSDIKQDTWINFRYINNLSISGSGTLD
GQGKQSWPHNDCHTNPNCPKLAMTMGFAFVNNSNIKDITSLNSKMGHFNFFSVHHFNITGV
TITAPGDSPNTDGIKMGSCSNIQISDTNIGTGDDCIAILSGTTNLNISNVNCGPGHGISVGSLGK
NKDEKDAKDLIVRDVIFNGTSDGIRIKTWESSASKILVSNFVYENIQMIDVGKPINIDQKYCPHP
PCEHERKGESHVQIQNLKLKNIYGTSKNKVAMNLQCSKIFPCKNVELIDINIKQNGVKDGSST
SVCENVDGFARGKMFPQHCLN
>AT2G23900
MEFSINPSTLKMIRTSFCILVVLAISSFSMMEARDLASKGKTNIEYMALNCRKHTAVLTEFGAV
GDGKTSNTKAFKEAITKLAPKAADGGVQLIVPPGKWLTGSFNLTSHFTLFIQKGATILASQDES

EYPVVAPLPSYGQGRDAAGPTFASLISGTNLTDVVITGNNGTINGQGKYWWVKYRSGGFKGI
TRPYTIEIIFSQNVQISNITIIDSPAWNIHPVYCNNVIVKGVTILAPIDSPNTDGINPDSCTNTLIED
CYVVSGDDCIAVKSGWDQFGIKVGMPTQQLSIRRLTCISPDSAGIALGSEMSGGIKDVRIEDIT
LLQTQSAIRIKTAVGRGGYVKDIFARRFTMKTMKYVFWMSGAYNQHPASGFDPKAMPVITNIN
YRDMTADNVTQPARLDGFKNDPFTKICMSNIKIDLAAEPKKLLWNCTSISGVSSKVTPKPCSL
LPEKGAPVDCAFPVDKIPIESVVLNKCSA
>AT2G26620
MACIAFVLKALCLSLLLIVVASRSTIRPKVFNVQRHGSKPDGKTDNTNAFTSIWSRACKRISGS
SKIYVPKGTFYLGGVEFVGPCKNPIEFIIDGTLLAPANPNDIKQDTWINFRYINNLSISGSGTLD
GQGKQSWPLNDCHKNPNCPKLAISMGFAFVNNSNIKDITSLNSKMGHFNFFFVHHFNITGVTI
TAPSDSPNTDGIKMGSCSNIQISNTNIGTGDDCIAILSGTTKLNISNINCGPGHGISVGSLGKNK
DEKDVKDLFVRDVIFNGTSDGIRIKTWESSASKILVSNFVYENIQMIDVGKPINIDQKYCPHPP
CEHERKSHVQIQDLKLKNIYGTSKNKVAVNLQCSKSFPCKNVELIDINIKQNGLEDGSSITVCE
NVDGFARGKMFPQHCLN
>AT2G33160
MASRVLLILCVPSLFFFLAASTKEYPGQPKIFDVRNYGARADSQRDNAFAFTKAWNEACQWS
YGRSTVYIPSGIFYLRQVTFSGPCKSSITFFIRGTLLAPRNPYAINQEEWILFKYVDNLTVTGGG
LLDGQGSYSWPLNDCNKNTNCRTLAMNIGFAFVKSSKINGLRSINSKMGHFNLFSVEDFNIT
GVTITAPGDSPNTDGIKIGKSSHMQIYNVTIGTGDDCIAILDGTSNLDISDVRCGPGHGISVGSL
GRYKEEKNVQGLTVRNSIINGTTDGLRIKTWAKSVSQISVSNFLYENIQMINVGNPIVIDQQYC
PHGQCDSPGKYASHVQIKDVKYNNIWGTSTSKEALKMQCSKTFPCQDVELSNINLHYVGRD
GLVTALCENVGGSIRGKIVPANCRINLFLPSNIITDSNSTLEDKMQGIFQLANTTQTQRFHQLPL
WILWRLWNSRNILYFQRRHVPWETTLQLAQMDLQEWNEAVTPSHINSHGTTIRSHIPNWRRP
TNGWVKCNVDGSFINGNIPGKGGWIVRDHNGRYEFAGQAIGNCIDNALECEFQALLMAMQH
CWSKGYRRICFEGDNKEMFDLINGLKMHFGAFNWIRDILWWASKFEQTQFQWTNRQNNKP
ADILARTPLQHQFLFANHFWVPRVISFALHCDYTSSI
>AT2G40310
MASIAFVFKVLCVSFLFIFVASRPTIRPKTFNVQRHGSKPDGKTDNANVFTSIWSRACKRKSG
SSKIYVPKGIFYLGGVEFVGPCKNPIEFVIDGTLLAPANPSDIKQDTWINFRYINNLSISGSGTL
DGQGKQSWPLNDCHKNLNCPKLAMTMGFAFVNNSNIKDITSLNSKMGHFNFFSVHHFNITG
VTITASGDSPNTDGIKMGSCSNMHISNTNIGTGDDCIAILSGTTNLDISNVKCGPGHGISVGSL
GKNKDEKDVKNLTVRDVIFNGTSDGIRIKTWESSASKILVSNFVYENIQMIDVGKPINIDQKYCP
HPPCEHEQKGESHVQIQDLKLKNIYGTSKNKVAMNLQCSKSFPCKNIELIDINIKSNGLENSSS
IAVCENVDGSMSGKMVPQHCLN
>AT2G41850
MARCTNLVTVFLLWALLMFSWCKASRISPNVYDHSYKRFKSDSLIKRREDITGLRSFVRASLR
TPTTVSVSDFGAKGDGKTDDTQAFVNAWKKACSSNGAVNLLVPKGNTYLLKSIQLTGPCNSIL
TVQIFGTLSASQKRSDYKDISKWIMFDGVNNLSVDGGDTGVVDGNGETWWQNSCKRNKAK
PCTKAPTALTFYNSKSLIVKNLKVRNAQQIQISIEKCSNVQVSNVVVTAPADSPNTDGIHITNTQ
NIRVSESIIGTGDDCISIESGSQNVQINDITCGPGHGISIGSLGDDNSKAFVSGVTVDGAKLSG
TDNGVRIKTYQGGSGTASNIIFQNIQMDNVKNPIIIDQDYCDKSKCTTEKSAVQVKNVVYRDIS
GTSASENAITFNCSKNYPCQGIVLDRVNIKGGKATCTNANVVDKGAVLPQCNST
>AT2G43860
MVYNTSCLILPLALIILDFTLISSLAHPIPSTLNVLSYGAKPDGSKDSTKAFLAAWDVACASANP

TTIIVPKGRFLVGNLVFHGNECKQAPISIRIAGSIVAPEDFRIIASSKHWIWFEDVTDVSIYGGILD
AQGTSLWKCKNNGGHNCPTGAKSLVFSGSNNIKISGLTSINSQKFHIVIDNSNNVNIDGVKVS
ADENSPNTDGIHVESSHSVHITNSRIGTGDDCISIGPGSTNVFIQTIRCGPGHGISIGSLGRAE
EEQGVDNVTVSNVDFMGTNNGVRIKTWGKDSNSFARNIVFQHINMKMVKNPIIIDQHYCLHK
PCPKQESGVKVSNVRYEDIHGTSNTEVAVLLDCSKEKPCTGIVMDDVNLVSVHRPAQASCDN
ANGSANDVVPFTPCLKREIIIT
>AT2G43870
MASLLVLFVFFFISSCSAQSYNVLSFGAKPDGKTDATKAFMAVWQTACASSRPVTIVVPKGRF
LLRSVTFDGSKCKPKPVTFRIDGTLVAPADYRVIGNEDYWIFFQHLDGITVYGGVLDARGASL
WDCKKSGKNCPSGATTIGFQSSSNVVVSGLTSLNSQMFHVVINGCNNVKLQGVKVLAAGNS
PNTDGIHVQSSSSVSIFNTKISTGDDCVSIGPGTNGLWIENVACGPGHGISIGSLGKDSVESG
VQNVTVKTVTFTGTDNGVRIKSWARPSSGFAKNIRFQHCVMNNVENPIIIDQNYCPDHDCPR
QVSGIKISDVLFVDIHGTSATEVGVKLDCSSKKPCTGIRLEDVKLTYQNKPAASACTHAGGIEA
GFFQPNCL
>AT2G43880
MANNISFPCAIIFFSINIFFLIKSSHAMPSFNVQRYGARGDGRADATKSFLTAWSLACGSRARA
MVYVPRGTYLVKNLVFWGPCKNIITFKNDGTLVAPANYWDIGNSGYWILFAKVNRISVYGGTID
ARGAGYWSCRKKGSHCPQGARSISFSWCNNVLLSGLSSFNSQNMHVTVHHSSNVRIENVRI
RAPSGSPNTDGIHVQSSSGVTISGGTIATGDDCIALSQGSRNIWIERVNCGPGHGISIGSLGD
YANEEGVQNVTVTSSVFTKTQNGVRIKTWARPSRGFVNNVVFRNLIMNNVENPVIIDQNYCP
NGKGCPRQSSGVKISGVTFANIKGTSTTPIAMKLDCSGSNHCTGLRLQDIKLTYMRRSSASY
CRNAHGRASGVMVPRNCM
>AT2G43890
MDNNKLVAVLLMFFSSFLLMKSSTAASNYNVVSFGAKPDGRTDSTKAFLGAWQAACRSAAA
VTVTVPRGSFLLKPVEFRGPCRSRITFQIYGTIVAPSDYRGLGNSGYWILFVKVNRISIIGGTLD
ARGASFWACRKSGKSCPVGARSMTFNWANDVVVSGLTSINSQTTHLVINSCNNVIVRKVKLV
APDQSPNTDGLHVQGSAGVTVTDGTFHTGDDCISIGPGTRNLYMSKLNCGPGHGISIGSLGR
DANEAGVENITLINSVFSGSDNGVRIKTWARQSTGFVRNVLFQNLIMKNVQNPIIVDQNYCPS
NQGCPKQGSGVKISQVVYRNIQGTSRTQQALTFDCSRSNPCQAIRLHDIKLTFNGRSATSTC
KNIKGVKAGVVMPQGCL
>AT3G06770
MSVVTILILALTSVIPIYANVHGAGKICDELGRRSLSTRPHSVSITDFGAVGDGKTLNTLAFQNA
VFYLMSFADKGGAQLYVPPGHWLTGSFSLTSHLTLFLENGAVIVASQDPSHWEVVDPLPSYG
RGIDLPGKRYKSLINGNKLHDVVVTGDNGTIDGQGLVWWDRFTSHSLKYNRPHLIEFLSSEN
VIVSNLTFLNAPAYSIYSIYSSHVYIHKILAHSSPKSPYTIGIVPDSSDYVCIQNSTINVGYDAISLK
SGWDEYGIAYSRPTENVHIRNVYLRGASGSSISFGSEMSGGISDVVVDNAHIHYSLTGIAFRT
TKGRGGYIKEIDISNIDMLRIGTAIVANGSFGSHPDDKYDVNALPLVSHIRLSNISGENIGIAGKL
FGIKESPFSSVTLSNVSLSMSSGSSVSWQCSYVYGSSESVIPEPCPELKRDADAYGRAAV
>AT3G07820
MGGYFGVSTIFIICLLGISANAEVFTIGSSSGSDITQALLKAFTSACQSSSPSKVVIPKGEFKLG
EIEMRGPCKAPIEVTLQGTVKADGNAIQGKEKWVVFGNIDGFKLNGGGAFDGEGNAAWRVN
NCHKTFECKKLPISIRFDFILNSEIRDISSIDAKNFHINVLGAKNMTMNNIKIVAPEDSPNTDGIH
LGRSDGVKILNSFISTGDDCISVGDGMKNLHVEKVTCGPGHGISVGSLGRYGHEQDVSGIKVI
NCTLQETDNGLRIKTWPSAACSTTASDIHFEDIILKDVSNPILIDQEYCPWNQCNKQKASTIKLV

NISFKNIRGTSGNKDAVKLLCSKGYPCQNVEIGDIDIKYNGADGPATFHCSNVSPKILGSQSPK
ACSAPAA
>AT3G07830
MGSYFGISTIFVICLLGFSANAHEVLRLTSSPGSDITQALLRAFTTACQSPTPRKVVIPKGQFKL
GEIMMSGPCKSPVEITLLGTVLADGNSIHGKEKWVVFQRMDGFRLNGGGTFDGEGNAAWR
VNNCHKTFECKKLPISIRFDFVTNAEIRDISSIDAKNFHINVIGAKNMTFDNVKIMAPAESPNTD
GIHLGRSVGVSIINSRISTGDDCVSVGDGMVNLLVKNVVCGPGHGISVGSLGRYGHEQDVSG
IRVINCTLQETDNGLRIKTWPSAACSTTASNIHFENIILRNVSNPILIDQEYCPWNQCNKQKSSS
IKLANISFRRIRGTSGNKDAVKLLCSKGYPCENVQVGDINIQYTGADGPATFMCSNVRPKLVG
TQFPKACNTPPVLTQPK
>AT3G07840
MGSYLGISAVLFVISLLGFSANAEVFNVGGSPGSDITQALLKAFTSACQAPTASKVVITKGEFK
LGEIEMTGPCKAPVEINLQGTLKADGKAIQGKERWVVFLRINGFKLNGGGIFDGEGNAAWRV
NNCHKTFECKKLPISIRFDFVENAEIRDISSIDAKNFHINVLGAKNMTFDNVKVIAPAESPNTDG
IHLGRSEGVKILNSKIATGDDCISVGDGMKNLHVENVMCGPGHGISVGSLGRYVHEQDVTGIT
VVNCTLQGTDNGLRIKTWPSAACATTASGIHFENIILNNVSNPILIDQEYCPWNQCNKQKPSTI
KLVDISFKNIRGTSGNKDAVKLLCSKAHPCANVEIGNINLEYKGADGPPTFMCSNVSPKLVGT
QNPKACSAGPTPKTAAKGDL
>AT3G07850
MVGSHKASGVLLVLLVVMATTIANGTPVVDKAKNAATAVEDTAKNAATAVGGAAASVGAKVS
GAKPGAAVDVKASGAKGDSKTDDSAAFAAAWKEACAAGSTITVPKGEYMVESLEFKGPCKG
PVTLELNGNFKAPATVKTTKPHAGWIDFENIADFTLNGNKAIFDGQGSLAWKANDCAKTGKC
NSLPINIRFTGLTNSKINSITSTNSKLFHMNILNCKNITLSDIGIDAPPESLNTDGIHIGRSNGVNL
IGAKIKTGDDCVSIGDGTENLIVENVECGPGHGISIGSLGRYPNEQPVKGVTVRKCLIKNTDN
GVRIKTWPGSPPGIASNILFEDITMDNVSLPVLIDQEYCPYGHCKAGVPSQVKLSDVTIKGIKG
TSATKVAVKLMCSKGVPCTNIALSDINLVHNGKEGPAVSACSNIKPILSGKLVPAACTEVAKPG
P
>AT3G07970
MYEKIIILSVFLLTFLPSCFSSYPFNHRDDLFMSSNVYYETNRQHQHGHNTRNSHLKNRHGYA
PRSSPRSFNVNTFGAKANGNDDSKAFMKAWEAACSSTGIVYIVAPKNRDYMLKAVTFSGPC
KSSLIIFKIYGRIEAWENPSDYKERRHWIVFENVNNLRVEGGGRIDGNGHIWWPKSCKINPQL
PCLGAPTAVTFVECNNLRVSNIRLENAQQMHLTFQDCKNVKALNLMVTSPADSPNTDGIHVS
GTQNILIQDSIVRTGDDCISIVSGSENVRATGITCGPGHGISIGSLGEDNSEAYVSNVVVNKATL
IGTTNGVRIKTWQGGHGMAKNIIFQDIIMKNVTNPIIINQDYCDRVEACPEQKSAVQVSNVLYK
NIQGTSSRPIAVKFVCSKNIPCRGISMQNVKLVDQTQQDVSKASCSNVKLDTRGNVSPLCT
>AT3G14040
MVGSHKASGVLLVLLVVMATTIANGTPVVDTAKNAATAVEDTAKNAATAVGGAAASVGAKVTG
AKPGGASLDVKASGAKGDGKTDDSAAFAAAWKEACAAGSTITVPKGEYLVESLEFKGPCKG
PVTLELNGNFKAPATVKTTKPHAGWIDFENLADFTLNGNKAIFDGQGSLAWKANDCAKTGKC
NSLPINIRFTGLTNSKINSITSTNSKLFHMNILNCKNITLTDIGIDAPPESLNTDGIHIGRSNGVNLI
GAKIKTGDDCVSIGDGTENLIVENVECGPGHGISIGSLGRYPNEQPVKGVTVRKCLIKNTDNG
VRIKTWPGSPPGIASNILFEDITMDNVSLPVLIDQEYCPYGHCKAGVPSKVKLSDVTIKNIKGT
SATKVAVKLMCSKGVPCTNIALSDINLVHNGKEGPAVSACSNIKPILSGKLVPAACTEVAKPGP
>AT3G15720

MKKKTWFLNFSLFFLQIFTSSNALDVTQFGAVGDGVTDDSQAFLKAWEAVCSGTGDGQFVV
PAGMTFMLQPLKFQGSCKSTPVFVQMLGKLVAPSKGNWKGDKDQWILFTDIEGLVIEGDGEI
NGQGSSWWEHKGSRPTALKFRSCNNLRLSGLTHLDSPMAHIHISECNYVTISSLRINAPESS
PNTDGIDVGASSNVVIQDCIIATGDDCIAINSGTSNIHISGIDCGPGHGISIGSLGKDGETATVEN
VCVQNCNFRGTMNGARIKTWQGGSGYARMITFNGITLDNVENPIIIDQFYNGGDSDNAKDRK
SSAVEVSKVVFSNFIGTSKSEYGVDFRCSERVPCTEIFLRDMKIETASSGSGQVAQGQCLNV
RGASTIAVPGLECLELSTDMFSSAQLLEQTCMSAQSVQPRTTTQPMQDPIWVFQSRGKQLR
VYNIAILVSFISLVTYILAR
>AT3G16850
MKMPVALVWLLAFTILLISGEGNNAICKENFKLDPRPHSVSILEFGAVGDGKTLNTIAFQNAVF
YLKSFADKGGAQLYVPPGKWLTGSFNLTSHLTLFLEKGATILASPDPSHWDVVSPLPSYGRGI
ELPGKRYRSLINGDNLIDVVITGENGTFDGQGAAWWEWLESGSLNYSRPHIIEFVSSKHILISN
LTFLNAPAINIHPVYCSQIHIRKVLIETSVDSPHVLGVAPDSSDNVCIEDSTINVGHDAVSLKSG
WDQYGIHYGRPTTAVHIRNLRLKSPTGAGISFGSEMSGGVSDVTVERLNIHSSLIGVAFRTTR
GRGGYIRNITISDVDLTSVDTAIVANGHTGSHPDDKFDRDALPVVTHIVMRNFTGVDIGVAGNL
TGIGESPFTSICLADIHLQTRSEESWICSNVSGFSDDVSPEPCQELMSSPSSCFAGGSIYEGD
ATAQSYYSW
>AT3G26610
MKTVKSLPILAMLLGVIIVAAAISTVSVEGRKHHVKKIKPKHRRHSKNTPTGSPAPAPYPSTNE
GVFNIFSYGAKGDGVSDDSKALVGAWKAACKVVGGKVEIPAGTQFLVKAVTLQGPCKEETVV
QIEGILVAPEKIGSWPNSSLFQWLNFKWVSHLTIQGSGTLNGRGYNWWNLDTYQTQTRNKYI
PPMKPTALRFYSSNNVTVRDISIVNSPLCHLKFDDSDGVKVNNITISSPENSPNTDGIHLQNTR
NVEIQHSNIACGDDCVSIQTGSSNVHIHHINCGPGHGISIGGLGKDKSVACVSDIIVEDISIQNT
LAGVRIKTWQGGLGVVKNLTFSNIQVKDVKVPIVIDQYYCDKSKCKNQTRAVSISGVKYNNIV
GSFTVQPVRIACSNNVPCMDVDLMDIRLRPSGGIRGLQTHQQQALCWNSYGKTQGPLVPS
SIGYCLRKSNIGGYYSQKVSRSYDKICPS
>AT3G42950
MVRRFLSSHKSLITVFWIATFASLFIWQFGGVSTNLYSGFSLFWSSSSTTTTVFSGGFPKLRP
VVFNLTDFGAVGDGVTINTEAFEKAIYKISKLAKKGGGQLNVPPGRWLTAPFNLTSYMTLFLSE
NAEILALQDEKYWSLLPPLPSYGYGREHHGPRYGSFIHGQNLRDVVVTGNNGSINGQGQTW
WKKYRQKLLNHTRGPLVQIMWSSDIVFANITLRDSPFWTLHPYDCKNVTITNMTILAPVFEAP
NTDGIDPDSCEDMLIENSYISVGDDGIAIKSGWDQYGTTYGKPSKNILIRNLIIRSMVSAGISIG
SEMSGGVSNITVENILIWSSRRGVRIKTAPGRGGYVRDITFRNVTLDELRVGIVIKTDYNEHPD
GGFNPQAFPILENINYTGIYGQGVRVPVRIQGSKEIPVKNVTFRDMSVGITYKKKHIFQCAYVE
GRVIGTIFPAPCDNLDRYDEQERLVKQSDSQNATDIDYEI
>AT3G48950
MIRPTFGLLVFLAIFLFPAIESRSHRNSVTSKIEFSALNCRKHSAILTDFGAVGDGKTSNTKAFR
NAISKLSQMATDGGAQLVVPPGKWLTGSFNLTSHFTLFIQRGATILASQDESEWPVIAPLPSY
GKGRDGTGTGRFNSLISGTNLTDVVITGNNGTINGQGQYWWDKFKKKQFKITRPYLIEILFSK
NIQISNITLIDSPSWNIHPVYCNSVIVKSVTVLAPVTVPNTDGINPDSCTNTLIEDCYIVSGDDCI
AVKSGWDQYGIKFGMPTQQLSIRRLTCISPKSAGVALGSEMSGGIKDVRIEDVTLTNTESAIRI
KTAVGRGAYVKDIYARRITMKTMKYVFWMSGNYGSHPDEGFDPKALPEITNINYRDMTAENV
TMSASLDGIDKDPFTGICISNVTIALAAKAKKMQWNCTDVAGVTSRVTPEPCSLLPEKKAQAK
NVDCAFPSDLIPIESVVLKKCFL

>AT3G57510
MARCCRHLAVFLCVLLMLSLCKALSSNVDDGYGHEDGSFESDSLLKLNNDDVLSLISSDETT
LEASTVSVSNFGAKGDGKTDDTQAFKKAWKKACSTNGVTTFLVPKGKTYLLKSTRFRGPCK
SLRNFQILGTLSASTKRSDYKDKNHWLILEDVNNLSIDGGSTGIINGNGKTWWQNSCKIDKSK
PCTKAPTALTLYNLKNLNVKNLRVKNAQQIQISIEKCNKVEVSNVEITAPGDSPNTDGIHITNTQ
NIRVSNSDIGTGDDCISIEDGTQNLQIFDLTCGPGHGISIGSLGDDNSKAYVSGINVDGAKFSE
SDNGVRIKTYQGGSGTAKNIKFQNIRMENVKNPIIIDQDYCDKDKCEDQESAVQVKNVVYKNI
SGTSATDVAITLNCSEKYPCQGIVLENVKIKGGTASCKNANVKNQGTVSPKCS
>AT3G57790
MMGSILLLLLFFSLVQSRSDTSYSKIQLPGDSLTLSVTDFGATGDGINYDTSAIQSTIDACNRH
YTSFSSICRVVFPSGNYLTAKLHLRSGVILDVTENAVLLGGPRIEDYYPAETSSDWYVVVANNA
TDVGITGGGAIDGQGSKFVVRFDEKKNVMVSWNQTGACLGDECRPRLVGFVDSINVEIWNIT
LREPAYWCLHIVRCENTSVHDVSILGDFNTPNNDGIDIEDSNNTVITRCHIDTGDDAICPKTYT
GPLYNLTATDCWIRTKSSAIKLGSASWFDFKGLVFDNITIFESHRGLGMQIRDGGNVSDVTFS
NINISTRYYDPSWWGRAEPIYITTCPRDSSAKEGSISNLLFVNITIDSENGVFLSGSPNGLLSDI
KFKNMNLTFRRWSNYSAGLVDYRPGCQGLVNHRATSGIIMEHVNGFRVENVDLKWSDDDDV
NAAWNVPLEFRPSTVNNVSFVGFTSGLYTKLFESDYVMVGENNIAFA
>AT3G59850
MHSSLLIVLLFLLSVSSSSAQTYNILSYGAKPDGKTDSTKAFTVLWAKACASVKPVTILVPKGR
FLLRSIIFDGSKCKRKSVTFRIQGTLVAPSDYRVIGKENYWILFQHLDGISVYGGVLDAQGASL
WSCKKSGKNCPSGATSIGFQSSRNVVISGLTSLNSQMFHVAINGCSNVKLDGVKVSADGNS
PNTDGIHVQSSSTVSILNSKISTGDDCVSIGPGTNGLWIENVACGPGHGISIGSLGKESVEVGV
QNITVKTATFTGTENGVRIKSWARPSNGFAKNIRFQHCVMNNVQNPIVIDQNYCPGNENCPN
QVSGIKISDVMFFDIHGTSATEVGVKLDCSSKKPCTGIRIQDVKLTYRNKPATTDCSHAGGSE
AGFQRPNSCL
>AT3G61490
MKNILACIVTIITLSNLITISQGRRVSQSFETFEYTAIICRSHSASITEYGGVGDGKTLNTKAFQS
AVDHLSQYSSEGGAQLFVPAGKWLTGSFNLTSHFTLFLHKDAILLAAQDLNEYPILKALPSYG
RGRDAAGGRFASLIFGTNLSDVIITGNNGTIDGQGSFWWQKFHGGKLKYTRPYLIELMFSDTI
QISNLTFLDSPSWNIHPVYSSNIIVKGVTIIAPVKSPNTDGINPDSCTNTRIEDCYIISGDDCIAVK
SGWDEYGISFGMPTKHLVIRRLTCISPYSAAIALGSEMSGGIEDVRAEDITAYQTESGVRIKTAV
GRGAFVKNIYVKGMNLHTMKWVFWMTGNYKAHADSHYDPHALPEITGINYRDIVAENVSMA
GRLEGISGDPFTGICISNATISMAAKHKKAIWMCSDVEGVTSGVDPKPCDLLDGQESETTKKK
MIDGGCDFPTDVLEIDNVELKTCSYQMS
>AT3G62110
MKRSFLLLYVLLVQAFYGAWCSVGESLHCEYSNLASLHRPHSVSITEFGAVGDGVTLNTKAF
QNALFYLNSFSDKGGAKLFVPAGQWLTGSFDLISHLTLWLDKGATILGSTSSENWPVVDPLPS
YGRGRELPGRRHRSLIYGQNLTDVVITGENGTIDGQGTVWWDWFRNGELNYTRPHLVELMN
STGLIISNLTFLNSPFWNIHPVYCRDVVVKNLTILAPLESPNTDGVDPDSSTNVCIEDCYIVTGD
DLVSIKSGWDEYGISYARPSSKIKINRLTGQTTSSSGIAIGSEMSGGVSEIYIKDLHLFNSNTGI
RIKTSAGRGGYVRNVHILNVKLDNVKKAIRFTGKYGEHPDEKYDPKALPAIEKITFENVNGDGI
GVAGLLEGIEGDVFKNICFLNVTLRVKKNSKKSPWECSNVRGYSQWVSPEITCDSLKESIFPE
HGSDCFGLSENNMEISSGLSRSPWLLSW
>AT4G01890

MLKLSRDPILCITTLILIITFSLLSYGTEARLHHQASQPPSPSPNPNDPSKSPSRSQDLDHEVVY
DVRKYGAVGNGVADDTVSFKTAWDSACSNNKNNTASVLHVPYGFTFMIRSTIFTGPCRSYQY
FQVDGTIVPRDGPKSWPSSLNKRQWLAFYRINGMALQGAGVIDGRGQNWWDLPCKPHQQ
NVNKTKLAGPCESPAALRFFMSSNVIVKGLSIKNSPQVHLKLDGCHVVHINSLRIISPPASPNT
DGIHIENSNSVEIYNSVISNGDDCVSIGPGAYDIDIRNITCGPGGHGISIGSLGEKNSHACVSNV
TVRDSFIKFSENGVRIKTWQGGSGSVSGVTFDNIHVDTVRNPIIIDQYYCTTKSCANKTSAVFV
NDIVYQSIKGTYDIRSPPMHFGCSNNVPCTNLTLSNIELLPSKEDIVVGPFCWNAYGITDEFSV
PLISCLKSNPSTSLLSGLSGRCGSP
>AT4G13760
MTSVVFAFKVLCVSFLFIVVASRPTIRPKMFNVQRHGSKSDGKTDNTNVFTSVWNRACRRKS
GSSKIYVPKGTFYLGGVEFVGPCKNPIEFLIDGTLLAPANPNNIKQDTWIKFKYINDLSISGSGT
LDGQGKQSWPLNDCHKNPNCPKLAMTMGFAFVNNSNIKDITSLNSKMGHFNFFSVHHFNIT
GVTITAPGNSPNTDGIKMGSCSNIHISNTNIGTGDDCIAILSGTTNLDISNVNCGPGHGISVGSL
GKNKDEKDVKDLTIRDVIFNGTSDGIRIKTWESSASKILVSNFLYENIQMIDVGKPINIDQKYCP
HPPCEHEQKGESHVQIQNLKLKNIYGTSKNKVAVNLQCSKRFPCKNIELIDINITNNGLVDSFS
TLVCENVDGSVSGKMVPQHCIN
>AT4G18180
MENFRYLKFFFFFIFVILSPSEAGNIVDKSSVFLVDVRSFGARANDHRDHTKAFVAAWDKACK
SSSSSVNLIIPRGEFSVGSLRFSGPCTNVSNLTVRVKASTDLSKYRSGGGWIQFGWINGLTLT
GGGTFDGQGALAWPFNNCTSDSNCKLLPTSLKFVGMNRTVVRRISSVNSKFFHIALVECRDF
KGTRLNITAPSDSPNTDGIHIERSSNVYFSRSHIATGDDCVSIGQGNSQITITSIKCGPGHGISV
GSLGRYPNEKDVNGLVVKDCKISGTTNGIRIKTWANSPGLSAATNMTFENIIMNNVTNPIIIDQS
YCPFSSCISNVPSKVELSEIYFKNIRGTSSSLVAVQLHCSRGMPCKKVYLENVHLDLSSSDGG
RKQSSNRGNEAVSSSCRNVRANYIGTQIPPPCH
>AT4G20050
MELRKSQVAMPVFLAIMSLMVSQVVFAEKDSGSMSPHDRALAEMQALKASLVRRNLPALVSP
PPTPPQAVPGPRVYQVISYGADPTGKLDSTDAILKAMEEAFDGPNHGVLMQGINDLGGARID
LQGGSYLISRPLRFPSAGAGNLLISGGTLRASNDFPVDRYLIELKDESSKLQYIFEYITLRDLLI
DCNYRGGAIAVINSLRTSIDNCYITRFGDTNGILVKSGHETYIRNSFLGQHITAGGDRGERSFS
GTAINLMGNDNAVTDTVIFSARIGVMVSGQANLLSGVHCYNKATGFGGTGIYLRLPGLTQNRI
VNSYLDYTGIVAEDPVQLQISGTFFLGDAFILLKSIAGYIRGVSIVDNMFSGSGHGVQIVQLDQ
RNTAFDDVGQVVVDRNSVNGMVEKSTVARGSVDGNGTSWTVDFNPVLLFPDLINHVQYTLV
ASEAGVFPLHALRNVSDNRVVVETNAPVTGTVYVTVNQGV
>AT4G23500
MTSLSTAGYLLLLLLLPISYDGGFIGAADAEHDLRATDLHNHRHRRHRNHHRGEEFEYSAISC
RAYSASLDEFGAVGDGVTSNTAAFRDAVSQLSRFADYGGSLLFVPAGRWLTGNFNLTSHFTL
FLHRDAVILASQEESDYEVIEPLPSYGRGRDTDGGRFISLLFGSNLTDVVITGENGTIDGQGEP
WWGKFKRGELKYTRPYLIEIMHSDGIQISNLTFLNSPSWHIHPVYSSNIYIQGLTILAPVTVPNT
DGINPDSCTNTRIEDCYIVSGDDCIAVKSGWDQYGINYGMPTKQLLIRRLTCISPDSAVIALGS
EMSGGIEDVRAEDIVAINSESGIRIKTAIGRGGYVKDVYVRGMTMMTMKYVFWMTGSYGSHP
DDHYDPKALPVIQNINYQDMVAENVTMPAQLAGISGDQFTGICISNVTITLSKKPKKVLWNCTD
VSGYTSGVTPQPCQLLPEKQPGTVVPCNFPEDPIPIDEVKLQRCYSRRRNM
>AT4G23820
MWRLSVSIFLFSCLFVSSSSLGDSEATCSGIVPLRYRYDKISITDFGGVGDGRTVNTKAFRAAI

YRIQHLKRRGGTLLYIPPGVYLTESFNLTSHMTLYLAKGAVIRAVQDTWNWPLIDPLPSYGRG
RELPGGRYMSFIHGDGLRDVVITGQNGTIDGQGEVWWNMWRSRTLKYTRPNLIEFKDSKEIII
SNVIFQNSPFWNIHPVYCSNVVIHHVTILAPQDSPNTDGIDPDSSYNVCIEDSYISTGDDLVAIK
SGWDQYGIAYGRPSSNITIRRITGSSPFAGIAIGSETSGGIKNIIAEHITLSNMGVGVNIKTNIGR
GGYIKNIKISDVYVDTAKYGIKIAGDTGDHPDENYNPNALPVVKGIHIKNVWGVNVRNAGSIQG
LKGSPFTGICLSEINLHGSLNSYKTWKCSDVSGTSLKVSPWPCSELRTTGGSNLCSSTF
>AT4G32370
MFNFFFTMKDFKTLFSLIIVISYYFEYGEGQRILSIKDFIIDTNHTIIDYSQAFQEAWKGLCEDET
PNGSALVIRKNETYTLQPSTFRGPCVSSNIHIQIDGKLEGPRKPIYWKNKENRSWLGFKDVEG
LVINGSGVLNPHGEAWWKSVSLSKRPTTISFASCMDIVYNGLHHINSPRNHISIYGCTNATLSN
LDISAPEDSPNTDGINICLSHRIQILDSSIQTGDDCVAITGGRGGSSDINITGVACGPGHGISIGS
LGKDNERDDIVENVNVRSCSFTGTQNGARIKTWNGGRGLAKNILYENITLIDAGYPIIINQHYF
DNKKKHYFDKSFLKVIFKL
>AT4G32375
MVQMAFKEAWNALCEANVNGTTTSLVINANETYIVQPQLFQGPCASRNIHIQIDGKLEAPKMV
REWGNYESKCWLCFTNVKGLVLNGSGILHPHGEAWWSSIEHSHRPRTIGFSGSSNILYNGLT
QMNSPKNHISILDCTNVTLSNFHLIAPKDSPNTDGIDIAHSNNIRIFNSSIQTGDDCIAINGGSYD
INITHVACGPGHGISIGSLGRYSVNDTVQNVKIRHCSFNGTENGARIKTWTVRGGLGVAKNILY
ENITLTDTKYPIIIDQHYCNGGHNCTKEAMTAVKVSNVTFRYFTGTCANDIAIKLDCDEVTGCK
DIVMEHINITSSSTKRPLTAYCQFADIISHFVSMKIKCDYYEEPLVPVEPPQQVEPPTPTKPLAP
AKPPRHVGPLMPTKPPTMFPKPLAPAKSPRHVELPMPTKPPTMFPKPLAPAKPPRHVEPPM
PTKPPTMFPKPLAPAKPPVYYAKPPAPNAQPSMLSFLSCLI
>AT4G32380
MANDWGRNKLDCWLCFEKVTGLVLTGSGVLNTHGESWWSSVALQSRPVAVRFFGCQNILY
NGLTQINSPRNHITILDSNNATLSNLHLIAPASSPNTDGIDISHSQNINIMSSTIKTGDDCVAIKRN
SYNINVTYVTCGPGHGISIGSLGEGGASEVVQNVNVRHCTFTGTQNGARIKTWPGGQGFVK
NILYEDITLINANFPIIIDQQYRDNAGQYKQSAGATAVKVSDVTFRSFTGTCAAPIAIKLDCDPNT
GCDNIVMEQINIASSSPKTPLTSYCKFAHVVSRFVSIPITCSFHTEDSQPASLNPQPSAPYAISP
TTPHTQPHAPTTQPPLFFRFYTNFKAFLGRLGRNC
>AT4G33440
MEPEKNPTTVLSAITRPSWAFLLLVFTVLAILSLQISSNSFLPLWIPTSQYDDPVTCSGFFNHD
PFPNRIVMSITDFGGVGDGKTSNTAAFRRAVRHLEGFAAEGGAQLNVPEGTWLSGSFNLTSN
FTLFLERGALILGSKDLDEWPIIEPLPSYGRGRERPGGRHISLIHGDNLTNVVITGENGTIDGQ
GKMWWELWWNRTLVHTRGHLIELKNSHNILISNLTLLNSPFWTIHPVYCSNVVIRNMTILAPM
NAPNTDGIDPDSSTNVCIEDCYIESGDDLVAVKSGWDQYGMAVARPSSNIVIRRISGTTRTCS
GVGIGSEMSGGIFNITVEDIHVWDSAAGLRIKTDKGRGGYISNITFNNVLLEKVKVPIRFSSGS
NDHSDDKWDPKALPRVKGIYISNVVSLNSRKAPMLLGVEGTSFQDVCLRNVTLLGLPKTEKW
KCKDVSGYASDVFPLSCPQLLQKKGSIAQCSYR
>AT4G35670
MKTILDISILINFLCFGIVYGKNYNVLNFDAKGDGQTDDSEAFLQAWTAACGGDGDIKTLLIPS
GKTFLLQPTVFQGPCKSSSIKVQLDGTIVAPSDKFAWSDPISRMWIKFSTVSGLIIVGSGTIDS
RGSSFWELNLKASQRPTALHISKCDNLRINGITSIDSPKNHISIKTCNTVAISNINLFAPETSPNT
DGIDISDSTNINIFDSTIQTGDDCIAINSGSSNINITGINCGPGHGISVGSLGAGGAEAKVSDVQ
VTHCTFNQTTNGARIKTWLGGQGYARNISFTDITLVNTKNPIIIDQHYIDKGRLTEESAVAISNV

KFVDFRGTSSNKNAITLKCSETTHCVDVVMDGIDITMANGGKPKVNCQYVDGESSDTDLMR
DCFKNNTSS
>AT5G14650
MCRLTLKCLSLNFLLLISLFSSRFGTCDARYSVYWKGNRRSIAEGGSSGTINVLDHGAKGDGT
SDDTKAFEDAWQVACKVAASTLLVPSGSTFLVGPVSFLGKECKEKIVFQLEGKIIAPTSASAW
GSGLLQWIEFKALQGITIKGKGIIDGRGSVWWNDMMGTKMPRTKPTALRFYGSNGVTVSGITI
QNSPQTHLKFDNCISIQVSDFTTSSPGDSPNTDGIHLQNSQDAVIYRSTLACGDDCISIQTGC
SNINIHDVDCGPGHGISIGGLGKDNTKACVSNITVRDVTMHETTNGVRIKSWQGGSGSVKQV
MFSNIQVSNVANPIIIDQYYCDGGGCHNETSAVAVSNINYINIKGTYTKEPVRFACSDSLPCTGI
SLSTIELKPATGKASSLDPFCWKAHGELKTKTLPPIQCLKTEKSPEAASRSNNDAC
>AT5G17200
MKSSLFFVLLCLFQINKLCFCLEEESLKVKNGFGSNGFISVTSFGAIGDGKTDDTKAFLKAWE
AVCKGGHNRKILVPQGKTFMLKPLTFIGPCKSSTISLSIRGNLVAPGYTWYAGRYTTWISFDSI
NGLVVTGGGTIDGRGSLWWGNVNNRPCAMHFNNCNGLRISNLRHLNSPRNHVGLSCSQNI
EVRGLRMTAPGDSPNTDGIDISNCIGVHIHDSVIATGDDCIAINSGSSHINITGIFCGPGHGISV
GSLGVTGDFETVEEVRVKNCTFTKTQNGVRIKTYQNGSGYARKISFEDINMVASENPIIIDQTY
HNGGTNGGISKSSSSYQNCHLTAKQRTPSGNGKGVKVTDVRYARIRGSSASDQDITLNCDA
DLGCSDIVMDNVNMVSATFGHKLFSSCKNAHGSLFASKVDCLKH
>AT5G27530
MEDMVTIQKNGDIPAFVRVTTQWHGLMSFPETILRISILINFICFGLVNGQIYNVLKFGAEGDG
QTDDSNAFLQAWNATCGGEENINTLFIPSGKTYLLQPIEFKGPCKSTSIKLQLDGIIVAPSNITS
WSNPKSQTWISFSGVPGLMIDGSGTINGRGSSFWEALHICNCNNLTINGITSIDSPKSHISIKN
CHYVAISKINILAPENSPNTDGIDISYSTNVNIFDSTIQTGDDCIAINTGSSSINITQVNCGPGHGI
SVGSLGADGENAAVSDVYVTQCTFNKTTNGARIKTWQGGQGYARNISFENITLINVQNPIIIDQ
QYTDKVLLDATKDSAVAISSVKYVGFQGTTLNEDAIMLKCSAITYCKDMVIDDIEVTMENGEKP
KVECENVEGESSDNDLMRECFNSNSTFSGGSVGGFGWVYECPCVRMGGPNWRFSSRISR
TFLDPSSFHVH
>AT5G39910
MANFVMFLNFLLSLTVFHTFQSVQSHFSLYNDNNIFNVLNYGAIGDGFSDDSKAFKDAWEDT
CNYIGSESIMEIPEGNTFLLQPIEFHGPCKSKKIILSISGNLIAPESPYEWKCNKDDCHQWIEFA
HINGLYIDGHGLMACLKRPRGVVISHSSNVHISNIMVKDSPNFQMSLEDSKWVIVKQLTITADG
DSPNTDGIHIQRSQNVIVYDSNIRTGDDCISIGDGSKYINISRISCGPGHGISIGSLGRYGTKET
VENVVVRDCTFRETTNGVRIKTWQTKAVEIKNVMFNHIHGTSIKKPFVQLLCSKSVPCRDIFM
NDINIHDENEEEEKKYHKSLSRHDDHPSAECINVKGESNGVMKPKLACLESKRH
>AT5G41870
MKISQAPISVFVLFLLSTVLPHHLSLGAPITCSGIVPMKHRNEMLSISDFGAVGDGKTLNTKAF
NSAIDRIRNSNNSNEGTLLYVPRGVYLTQSFNLTSHMTLYLADGAVIKAVQDTEKWPLTDPLPS
YGRGREHPGRRYISFIHGDGLNDVVITGRNGTIDGQGEPWWNMWRHGTLKFTRPGLIEFNN
STNILVSHVVLQNSPFWTLHPVYCSNVVVHHVTILAPTDSYNTDGIDPDSSSNVCIEDSYISTG
DDLVAVKSGWDEYGIAYNRPSRDITIRRITGSSPFAGIAIGSETSGGIQNVTVENITLYNSGIGIHI
KTNIGRGGSIQGITISGVYLEKVRTGIKISGDTGDHPDDKFNTSALPIVRGITIKNVWGIKVERA
GMVQGLKDSPFTNLCFSNVTLTGTKRSPIWKCSDVVGAADKVNPTPCPELSATTQQGGSCE
NQS
>AT5G44830

MCGSGGNSKTFLIPSNQTFLLQPLTFQGPCKSPSVQVKFDGKIVAPINKAAWSESKLFRWVS
FKEIIGLTVNGSGTIHGRGSSFWKQLHFQRCNDLKIIGITSFNSPRNHISISECKRVQLTKIKLVA
PEDSPNTDGINISGSSDVDVYDTFIGTGDDCVAINNGSVNINITRMNCGPGHGISVGSLGRDG
EESIVENVQVTNCTFFRTDNGVRIKTWPNGKGYARNILFKDLTFRESKNPIIIDQNYVDKGRLD
VEESAVAISNVTFTDIRGTSQRNEIIKIDCSEVTYCKDIVLDKIDIATVDGNKPVVECSNVYGKSI
NTNDANGCFED
>AT5G44840
MEIILRGVFLLTLLHSILVNGQIYDVLEFGADGNGITDDSKAFVKAWSAMCGSGGNSKTFIIPSN
KTFLLQPLTFQGPCKSPSVQVKFDGKIVAPINKAAWSDYKLFRWVSFKEIIGLTVNGSGTIHGR
GSSFWKQLHFQRCNDLMITGITSFNSPKNHISISECKRVKITKIKLVAPHDSPNTDGINISESSD
VDIYDTVIGTGDDCVAINSGSMNINIARMNCGPGHGISVGSVGRDGEESIVENVQVTNCTFIR
TDNGARIKTWPNGKGYAKNILFKSLTFRETKNPIIIDQNYVDKGRLDVEESAVAISNVTFTDLR
GTSKLDEIIKIDCSKVTYCKDIVLDKIDIATVDGNKPIVECSNVYGKSINANEANGCFKT
>AT5G48140
MGRVHFGVSAFFVFCLLGLSANAKIFNINSPPGSDITNALLKAFNEACQFPTKSTVMIPKGEY
KLGEIVMMGPCKAPIRIALLGTVKADGNANGKEKWVAFRNINGFKLNGGGVFDGEGNAAWR
VNNCHKTFNCKKLPISIRFDFVTDAKIRGITSLDAKHFHINVIGAKNVTFEDVKIIAPAESPNTDG
IHVGRSDGIKIINSFISTGDDCVSVGDGMKNLLVERVTCGPGHGISIGSLGRYSHEENVSGIKII
NCTLQETDNGLRIKTWPSAACTTTASDIHFENILLKNVSNPILIDQEYCPWNQCNKQKPSTIKL
ANISFKKIRGTSGNKDAVKLLCSKGYPCQNVEVGDVNIQYTGADGPATFQCSNVSPKLVGTQI
PKACSSPVTKPPK
>AT5G49215
MPVSVALVLLLTLSSVILINGRSYGVGNICDRGRRPSERPHSVKITDFGAVGDGKTLNTLAFQN
AVFYLKSFADKGGAQLYVPPGRWLTGSFNLTSHLTLFLEKDAVILASQDPSHWQVTDALPSY
GRGIDLPGKRYMSLINGDMLHDVVVTGDNGTIDGQGLVWWDRFNSHSLEYSRPHLVEFVSA
ENVIVSNLTFLNAPAYTIHSVYCRNLYIHRVTANTCPESPYTIGIVPDSSENVCIQESSINMGYD
AISLKSGCDEYGLSYARPTANVQIRNVYLRAASGSSISFGSEMSGGISDVEVSDAHIHNSLSGI
AFRTTNGRGGYIKEIDISNIHMVNVGTAFLANGSFGTHPDSGFDENAYPLVSHIRLHDIVGENI
STAGYFFGTKESPFTSILLSNISLSIKNSASPADSWQCSYVDGSSEFVVPEPCLELKSFDSYY
GRAEAL

DNA

>ZmPG30

ATGGCGTGCACAGACAATGCGATGAGAGCCTTGTTCCTCCTGGTCCTCTTCTGCATCGTG
CATGGTGAGAAGGAAGAGTCAAAGGGCATCGATGCGAAAGCGTCCGGGCCTGGTGGGTCC
TTCGACATCACCAAGTTGGGCGCCTCCGGCAATGGCAAGACAGACAGCACGAAGGCTGTG
CAGGAGGCATGGGCATCGGCGTGCGGCGGCACTGGGAAGCAGACAATCCTCATACCCAAG
GGCGACTTCCTTGTCGGACAACTCAACTTCACAGGCCCTTGCAAGGGCGACGTGACCATC
CAGGTGGATGGCAATCTGCTGGCGACCACGGACCTAAGCCAGTACAAGGAACATGGTAAT
TGGATCGAGATTCTACGCGTGGATAACCTGGTCATCACCGGCAAGGGAAACCTTGACGGG
CAGGGCCCAGCCGTGTGGAGCAAGAACTCCTGCACCAAGAAGTATGACTGCAAGATCCTT
CCCAACTCGCTGGTGATGGACTTCGTGAACAACGGGGAGGTGTCCGGGGTCACGCTGCTC
AACTCCAAGTTCTTCCACATGAACATGTACCAGTGCAAGAACATGCTGATCAAGGACGTG
ACCGTGACGGCGCCCGGGGACAGCCCCAACACGGATGGCATCCACATGGGCGACTCATCC
GGGATCACCATCACCAACACCGTCATTGGCGTCGGCGACGACTGCATCTCCATCGGCCCT
GGGACCTCCAAGGTGAACATCACTGGCGTGACCTGCGGCCCCGGCCACGGCATCAGCATC
GGCAGCCTAGGGCGGTACAAGGACGAGAAGGACGTCACGGACATCAACGTCAAGGATTGC
ACTCTTAAGAAGACAATGTTCGGCGTCCGCATCAAGGCGTACGAGGACGCCGCCTCCGTG
CTCACCGTCTCCAAGATCCACTACGAGAATATCAAGATGGAGGACTCAGCCAACCCCATC
TTCATCGACATGAAGTACTGCCCCAACAAGTTGTGTACCGCCAACGGCGCCTCCAAGGTC
ACCGTCAAGGACGTCACCTTCAAGAACATCACTGGCACCTCCTCCACCCCGGAGGCCATT
AGCCTGCTCTGCACTGCCAAGGTCCCATGCACCGGCGTCACCATGGATGACGTCAACGTC
GAGTATAGCGGCACCAACAACAAGACCATGGCTATATGCACGAACGCCAAGGGCAGCACC
AAGGGTTGCCTCAAGGAGCTTGCATGCTTCTAG

>ZmPG31

ATGGCGTGCACAGACAATGCGATGAGAGCCTTGTTCCTCCTGGTCCTCTTCTGCATCGTG
CATGGTGAGAAGGAAGAGTCAAAGGGCATCGATGCGAAAGCGTCCGGGCCTGGTGGGTCC
TTCGACATCACCAAGTTGGGCGCCTCCGGCAATGGCAAGACAGACAGCACGAAGGCTGTG
CAGGAGGCATGGGCATCGGCGTGCGGCGGCACTGGGAAGCAGACAATCCTCATACCCAAG
GGCGACTTCCTTGTCGGACAACTCAACTTCACAGGCCCTTGCAAGGGCGACGTGACCATC
CAGGTGGATGGCAATCTGCTGGCGACCACGGACCTAAGCCAGTACAAGGAACATGGTAAT
TGGATCGAGATTCTACGCGTGGATAACCTGGTCATCACCGGCAAGGGAAACCTTGACGGG
CAGGGCCCAGCCGTGTGGAGCAAGAACTCCTGCACCAAGAAGTACGACTGCAAGATCCTT
CCCAACTCGCTGGTGATGGACTTCGTGAACAACGGGGAGGTGTCCGGGATCACGCTGCTC
AACTCCAAGTTCTTCCACATGAACATGTACCAGTGCAAGAACATGCTGATCAAGGACGTG
ACCGTGACGGCGCCCGGGGACAGCCCCAACACGGATGGCATCCACATGGGCGACTCATCC
GGGATCACCATCACCAACACCGTCATTGGCGTCGGCGACGACTGCATCTCCATCGGCCCT
GGGACCTCCAAGGTGAACATCACTGGCGTGACCTGCGGCCCCGGCCACGGCATCAGCATC
GGCAGCCTAGGGCGGTACAAGGACGAGAAGGACGTCACGGACATCAACGTCAAGGATTGC
ACTCTTAAGAAGACAATGTTCGGCGTCCGCATCAAGGCGTACGAGGACGCCGCCTCCGTG
CTCACCGTCTCCAAGATCCACTACGAGAATATCAAGATGGAGGACTCAGCCAACCCCATC
TTCATCGACATGAAGTACTGCCCCAACAAGTTGTGTACTGCCAACGGCGCCTCCAAGGTC
ACCGTCAAGGACATCACCTTCAAGAACATCACTGGCACCTCCTCCACCCCGGAGGCCATT
AGCCTGCTCTGCACTGCCAAGGTCCCATGCACCGGCGTCACCATGGATGACGTCAACGTC
GAGTATAGTGGCACCAACAACAAGACCATGGCTATATGCACGAACGCCAAGGGCAGCACC

AAGGGTTGCCTCAAGGAGCTTGCATGCTTCTAG
>ZmPG55
ACGAATAAAGCATCGATCACGACAAGATGGCATGCACAGACAATGCGATGAGAGACTTGT
TCCTCCTGGTCCTCTTCTGCATCGTGCATGGTGAGAAGGAAGAGTCAAAGGGCATCGATG
CGAAAGCGTCCAGGCCTGGTGGGTCCTTCGACATCACCAAGTTGGGCGCCTCCGGCAATG
GCAAGACAGACAACACGAAGGATGTGCAGGAGGCATGTGCATCGGCGTGCGGCGGCACTG
GGAAGCAGACAATCCTCATACCCAAGGGCGACTTCCTTGTCGGACAACTCAACTTCACAT
GCCCTTGCAAGGGCGACGTGACCATCCAGGTGGATGGCAATCTGCTGGCGACCATGGACC
TAAGCCAGTACAAGGAACATGGTAAATGGATCGAGATTCTACGCGTGGATAACCTGGTCA
TCACCGGCAAGGGAAACCTTGACGGGCAGGGCCCAGCCGTGTGGAGCAAGAACTCCTGCA
CCAAGAAGTACGACTGCAAGATCCTTCCCAACTCGCTGGTGATGTACTTCGTGAACAACG
GGGAGGTGTCCGGGGTCACGCTGCTCAACTCCAAGTTCTTCCACATGAACATGTACCAGT
GCAAGAACATGCTGATCAAGGACGTGACCGTGACGGCGCCCGGGGACAGCCCCAACACGG
ATGGCATCCACATGGGCGACTCATCCGGGATCACCATCACCAACACCGTCATTGGCGTCG
GCGACGACTGCATCTCCATCGGCCCTGGGACCTCCAAGGTGAACATCACTGGCGTGACAA
GGACGAGAAGGACGTCACGGACATCAACGTCAAGGATTGCACTCTTAAGAAGACGATGTT
CGGCGTCCGCATCAAGGCGTACGAGGACGCCGCCTCCGTGCTCACCGTCTCCAAGATCCA
CTACGAGAATATCAAGATGGAGGACTCAGCCAACCCCATCTTCATCGACATGAAGTACTG
CCCCAACAAGTTGTGTACCGCCAACGGCGCCTCCAAGGTCACCGTCAAGGACGTCACCTT
CAAGAACATCACCGGCACCTCCTCCACCCCGGAGGCCGTTATCCTGCTCTACACAGCCAA
GGTCCCATGCACCGGCGTCACCATGGATGACGTCAACGTCGAGTATAGCGGCACCAACAA
CAAGACCATGGCTATATGCACGAATGCCAAGGGCAGCACCAAGGGTTGCCTCAAGGAGCT
TGCATGCTTCTTGACCCTCCGTCGACTGACCCATCTCTCTAGTTATAATTTTTCTCTCGT
CCTTGCATTGCCCATTTGTTGCTATCCATTGGTAACGGACAACAGTAAAACGACAGACAA
CCAACAGCAATATTATGTTCGACGGTGTAACACCCTGAATTTGAGGGTATAAAATTTCTT
CTCTAAATGCCAACCAAATTCAGGTGTTACCTCTTGTCTCTCTCTCTCTCTCTCTTTTCC
TTTTGATTAAAAGTAAGTGAATTAGGCGAGGGTTTAATTATTTATTTTGTCAAAGCTTAT
GTGAGTCATGAAATGTTGCATCATGCTGAGCCTAAATTATTCTTTTGTTTGATGCACATG
TTTGAATTGGTTCGAATTTGAAATTTGGTTTGAGTTTGATTTGAAAACCATAAAGAAATT
AAAATAGAAAAGACATTAGAAATTTCAAAAAAAGAAAAGGAAAGCAGCTCGAACCTCCTC
CCTCCTTGGCCTTTCGGCCCATCCAGCCCAATTAACCTCCCGCATCCGCTGACAAGCAGG
CCCCACCTATCGGCGCCACGCCCCGCTCGCCCTCTCTCTCTCTTCTTAGTGGGACCGCCC
CATCAGCACCGTGCCCTGCTCGCCCGCGCGTTCCCTCGCTGACTCGCGGCCCCTATTGTC
AGACCCATCCCCTCCCCTGCAACTGTCGCGCCCGCGCCCATGGCGTGCGCCCATGCGGTC
AAAGCGTGCCCACATCCCCACGACCTGCCCATGTCACCCGAGTGCTAGGTGAAGACCCCG
AACACTCCCTCAGCTTGCCCCTGCCTCACATTCGCTCTTTCCCTGCCCTCGCACTCACCC
AGCCGCAACGCCGCCCCCCGCTGTTGCTTCACCTTTCTGTTGTCGCCGCTGGACTTCTGC
CAGCGCTTTGGCCATGGTGAGCTTTGCCTGAGCTTGGCGCACCGGGAACCCACGATGGTT
TCCCCTTTCCTCAAATCCTCTACCCGATTTGCGCTCCACCTCTCCCCTGCGCAGGTCGGA
GCTCGCTGCCGTCGTGATTCTTCAACGTCCGGTCAACTAGAGCCCCATCGCGCCGTGCCA
AGCTGCCCGAAGCATCGTATCTGGGTAAGTGACATTCCTGTGTCCTTCTTTTGCGCCAAT
TCTGCTATACGTGGGGGATTTCAGCCATCGAAGTTGCCCCTCGCCGGGCCACAGCATCTT
CATGGCGTTCGACCACTGCAACCCCTCCCTGAGCCCGAACTTAGGCCTAGAGCACCCCAC
CCTCTCCCTGGAGCCTATGCCAACCGCAGAGCGCCGGTTTAA

>ZmPG29
ATGGCGTGCACAAACAATGCGATGAGAGCCTTGTTCCTCCTGGTCCTCTTCTGCATCGTG
CATGGTGAGAAGGAAGAGTCAAAGGGCATCGATGCGAAAGCGTCCGGGCCTGGTGGGTCC
TTCGACATCACCAAGTTGGGCGCCTCCGGCAATGGCAAGACAGACAGCACGAAGGCTGTG
CAGGAGGCATGGGCATCGGCGTGCGGCGGCACTGGGAAGCAGACAATCCTCATACCCAAG
GGCGACTTCCTTGTCGGACAACTCAACTTTACAGGCCCTTGCAAGGGCGACGTGACCATC
CAGGTGGATGGCAATCTGCTGGCGACCACGGACCTAAGCCAGTACAAGGACCATGGTAAT
TGGATCGAGATTCTACGCGTGGATAACCTGGTCATCACCGGCAAGGGAAACCTTGACGGG
CAGGGCCCAGCCGTGTGGAGCAAGAACTCCTGCACCAAGAAGTACGACTGCAAGATCCTT
CCCAACTCGCTGGTGATGGACTTCGTGAACAACGGGGAGGTGTCCGGGGTCACGCTGCTC
AACTCCAAGTTCTTCCACATGAACATGTACCGGTGCAAGGACATGCTGATCAAGGACGTG
ACCGTGACGGCGCCCGGGGACAGCCCCAACACGGATGGCATCCACATGGGCGACTCATCC
GGGATCACCATCACCAACACCGTCATTGGCGTCGGCGACGACTGCATCTCCATCGGCCCC
GGGACCTCCAAGGTGAACATCACCGGCGTGACCTGTGGCCCTGGCCACGGCATCAGCATC
GGCAGCCTAGGGCGGTACAAGGACGAGAAGGACGTCACGGACATCAACGTCAAGGATTGC
ACTCTTAAGAAGACGATGTTCGGCGTCCGCATCAAGGCGTACGAGGACGCCGCCTCCGTG
CTCACCGTCTCCAAGATCCACTACGAGAATATCAAGATGGAGGACTCAGCCAACCCCATC
TTCATCGACATGAAGTACTGCCCCAACAAGTTGTGTACCGCCAACGGCGCCTCCAAGGTC
ACCGTCAAGGACGTCACCTTCAAGAACATCACCGGCACCTCCTCCACCCCGGAGGCCGTT
AGCCTGCTCTGCACTGCCAAGGTCCCATGCACCGGCGTCACCATGGATGACGTCAACGTC
GAGTATAGCGGCACCAACAACAAGACCATGGCTATATGCACGAACGCCAAGGGCAGCACC
AAGGGTTGCCTCAAGGAGCTTGCATGCTTCTAG
>ZmPG32
AAGAGACACGAATAAAGCATCGATCACGACAAGATGGCGTACACAGACAATGCGATGAGA
GCCTTGTTCCTCCTAGCGCTCTTCTGCGTCGTGCATGGTGAGAAGGAAGAGTCAAAGGGC
ATCGATGCGAAAGCGTCCGGGCCCGGTGGGTCCTTCGAAATCACCAAGTTGGGCGCCTCC
GGCAACGGTAAGACAGACAGCACAAAGGCTGTGCAGGAGGCATGGGCATCGGCATGTGGC
GGCACCGGGAAGTAGACAATCCTCATATCCAAGGGCGACTTCCTTGTCGGACAACTCAAC
TTCACAGGCCCATGCAAGGGCGACGTGACCATCCAGGTGGATGGCAATCTGCTGGCGACC
ACGGACCTAAGCTAGTACAAGGAACATGGTAATTGGATCGAGATTCTACGCGTGGATAAC
CTGGTCATCACCGGCAAGGGAAACCTTGACGGGCAGGGCCCAGCCGTGTGGAGCAAGAAC
TCCTGCACCAAGAAGTACGACTGCAAGATCCTTCCCAACTCGCTGGTGATGGACTTCGTG
AACAATGGGGAGGTGTCCGGGGTCACGCTGCTCAACTCCAATTTCTTCCACATGAACATG
TACCGGCGCAAGGACATGCTGATCAAGGACGTGACCGTGATGGCGCCCGGGGATAGCCCC
AACACGGATGGCATCCACATGGGTGACTCATCCGGGATCACCATCACCAACACCGTCATT
GGCGTCGGCGACGACTGCATCTCCATCGGCCCTGGGACCTCCAAGGTGAACATCACCGGC
GTGACCTGCGGCCCCGGCCACGGCATCAGCATCGGCAGCCTAGGGCGGTACAAGGACGAG
AAGGACGTCACGGACATCAACGTCAAGGATTCCACTCTTAAGAAGAAGATATTCGACGTC
CGCATCAAGGCGTACGAGGACGCCACCTCCGTTCTCACCGTCTCCAAGATCCACTACGAG
AATATCAAGATGGAGGACTCAGCCAACCCCATCTTCATCGACATGAAGTACTGCCCCAAC
AAGTTGTGTACTGCCAACGGCGCCTCTAAGGTCACCGTCAAGGACGTCACCTTCAAGAAC
ATCACCGACACCTCCTCCACCCCGGAGGCCGTTAGCCTGCTCTGCACTGCCAAGATCCCA
TGCACCGGCGTCACCATGGACGACGTCAACGTCGAGTATAGCGGCACCAACAACAAGACC
ATGGCTATATGCACGAACGCCAAGGGCAGCACCAAGGGTTGCCTCAAGGATCTTGCATGC

TTCTAGACCCTTCGTCGACTGACCCATCTCTCTAGTTATAATTTTTCTCTCGTCCTTGCA
TTGCCCATTAGATGCTATCCATTGGTAATGCACAAAAGTAAAACGACAGACATCCGACAG
CTATATTATGTTCGACGGTGTAACACCCTGAATTTGAGGGTATAAAATTTCTTCTCTAAA
TGCCAACCAAATTCAGGTGTT
>ZmPG28
AAGAGACATGAATAAAGCATCGATCGCAACAAGATGGCGTGCACAGACAATGTGATGAGA
GCCTTGTTCCTCCTGGCGCTCTTCTGCGTCATGCATGGTGAGAAGGAAGAGTCAAAGCGC
ATCGATGCGAAAGCGTCTGGGCCTGGTGGGTCCTTTGACATCACCAAGTTGGGCGCCTCC
GGCAACGGCAAGATAGACAACACGAAGGCTGTGCAGGAGGCATGGGCATCAGCATGCGGC
GACACCGGGAAGCAAACAATCCTCATACCCAAGGGCGACTTCCTTGTCGGACAACTCAAC
TTCACAGGCCCGTGCAAGGGCGACGTGACCATCCATGTGGATGGCAATCTACTGTCGACC
ATGGACCTAAGCCAGTACAAGGAACATGGTAATTGGATCGAGATTCTACGCGTGGATAAC
CTGGTCATCACCGGCAAGGGAAACCTTGACGGATTGGGCCCAGCCGTGTGGAGCAAGAAC
TCCTGCGCCAAGAAGTACGACTGCAAGATCCTTCCCAACTCACTGGTGATGGACTTCGTG
AACAACGGGGAGGTGTCCGGGGTCACTCTGCTCAACTCCAAGTTCTTCCACATGAAAGCA
CTTACTTCTAGGGGAAGGGTCAAGGCATAGGCAGAAGGTCTAATTCCAGACTAAACTAAT
AATAGTTTGCTCGAGAAATGTACCAGTGCAAGGACATGCTGATCAAGGACGTGACCGTGA
CGGCGCTTGGGGACAGCCCCAACACGGATGGCATCCACATGGGCGACTCATCCGGGATCA
CCATCACCAACACCGTCATCAGCGTCAGCGACGATTGCATCTCCTTCGGCCCCGGGACCT
CCAAGGTGAACATCACCGGCGTGACCTACGGCTCCGGCCACGGCATCAGCATCGGCAGCC
TAGGGCGGTACAAGGATGAGAAGGACGACACAAACATCAACGTCAAGGATTGCACTCTTA
AGAAGACGACCTTCGGCGTCCGCATCAAGGCGTACGAGGACGCCGCTTCCGTGCTCACCG
TCTCCAAGATCCATTATGAGAATATCAAGATGGAGGACTCAGCCAACCCCATCTTCATCG
ACATGAAGTACTGCCCCAACAAGTTATGTACTGCCAACGACGCCTCCAAGGTCACCGTCA
AGGACGTCACCTTCAAGAACATCACCGGCACCTCCTCCACCCCGGAGGCCGTCAGCCTGC
TCTGCTCTGCCAAGATCCCATGCACCGGCGTAACCATGGACGACATCAACGTCGAGTATA
GCGGCACCAACAACAAGACCATGGCTATATGCACGAACGCCAAGGGCAGCACCAAGGGTT
GCCTCAAGGAGCTTGCATGCTTCTAGACCCTCCGTCGACTGACCCATCTCTCTAGTTATA
ATTTTTCTCTCATCCTTGCATTGCCCATTAGTTGCTATCCATTGGTAACGCACAACAGTA
AAACGATAGACATCCGACAGCTATATTATGTTCGACGGTGTAACACCCTGAATTTGAGGG
TATAAAATTTCTTCTCTAAATGCCAACCAAATTCAGGTGTTAC
>ZmPG33
AAGAGACACGAATAAAGCATCGATCACGACAAGATGGCGTACACAGACAATGCGATGAGA
GCCTTGTTGCGCTCTTCTACGTCGTGCATGGTGAGAAGGAAGAGTCCAAGGGCATCGATG
CGAAAGCGTCCGGGCCCGGTGGGTCCTTCGACATCACCAAGTTGGGCGCCTCCGGCAACG
GCAAGACAGACAGCACAAAGGCAGTGCAGGAGGCATGGGCATCAGCGTGTGGCGGCACTG
GGAAGCAGACAATCCTTATACCCAAGGGCGACTTCCTTGTCGGACAACTCAACTTCACAG
GCCCGTGCAAGGGCGACGTGACCATCTAGGTGGATGGCAATCTGCTAGCGACCACATACC
TAAGCCAGTACAAGGAACATGGTAATTGGATTGAGATTCTACGCGTGGATAACCTGGTCA
TCACCGGCAAGGGAAACCTTGACGGGCAGGGCCCATTCGTGTGGAGCAAGAACTCCTGCA
CCAAGAAGTACGACTGCAAGATCCTTCCCAACTCATTGGTGATGGACTTCGTGAACAACG
GGGAGGTGTCTGGGGTCACGCTGCTCAACTCCAAGTTCTTCCACATGAACATGTACCAGT
GCAAGGACATGCTGATCAAGGACGTGACCGTGACGGCGCCCGGGGACAGCCCCAACACGG
ATGGCATCCACATGGGCGACACATCCGGGATCACCATCACCAACACCGTCATCGGCGTCG

GCGACGACTGCATCTCCATCGGCCCCGGGACCTCCAAGGTGAACATCACTGGCGTGACCT
GCGGCCCTGGCCATGGCATTAGCATTGGCAGCCTAGGGCGGTACAAGGACGAGAAGGATG
TCACGGACATCAACGTCAAGGATTGCACTCTTAAGAAGACGACCTTCGGCGTCCGCATCA
AGGCGTACGAGGACGCCGCCTCCGAGCTCACCGTCTCCAAGATCCACTACGAGAATGTCA
AGATGGAGGACTCGACCAACCCCATCTTCATCGACATGATGTACTGCCCCAACAAGTTGT
GTACTGCCAACGGCGCCTCCAAGGTCACCGTCAAGGACGTCACCTTCAAGAACATCACCG
GCACCTCCTCCACCCCGGAGGCCATCAGCCTGCTCTGCACTGCCAAGATCCAATGCACCG
GCGTCACCATGGACGACGTCAACGTCGAGTATAGTGGCACCAACAACAAGACCATGGATA
TATGCACGAACACCAAGGGCTGCACCAAGGGTTGCCTCAAGGAGCTTGCATGCTTCTAGA
CCCTCCGTCGACTGACCAATCTCTCTAGTTATAATTTTTCTCTCGTCCTTGCATTGCCCA
TTAGTTGCTATCCATTGGTAACACACAACAGTAAAACGACAGACATCCGACAGCTATATT
ATGTTCGACGGTGTAACACCCTGAATTTGAGGGTATAAAATTTCTTCTCTAAATGCCAAC
CAAATTCAGGTGTT
>ZmPG36
ATGGCATGTATAGACAATGCAATGAGAGCCTTGTTCCTTCTAGCGCTCTTCTGCACCGTG
CATGGTGAGAAGGCAAAGTCAAAGGACAACGATTCAAAAGCGTCCGGGCCCGGTGGGTCC
TTCGACATCACCAAGTTGGGCGCCTCTGGCAATGGCAAGACGGATAGCACGAAGGCTGTG
CAGGAGGCGTGGGCATCGGCGTGCGGCGGCACCGGGAAGCAGACGATCCTCATCCCCAAG
GGCGACTTCCTCGTCGGACCACTCAACTTCACAGGCCCATGCAAGGGCGACGTGACCATC
CAGGTGAATGGCAATCTGCTGGCGACCACGGACCTAAGCCAGTACAAGGATCATGGTAAT
TGGATCGAGATTCTACGCGTGGACAACCTTGTCATCACCGGCAAGGGAAAGCTCGACGGG
CAGGGGCCAGCCGTGTGGAGCAAGAACTCCTGCGTCAAGAAGTACGACTGCAAGATCCTT
CCCAACTCGCTGGTGATGGACTTCGTGAACAACGGGGAGGTGTCCGGGATCACGCTGCTC
AACTCCAAGTTCTTCCACATGAACATGTACAAGTGCAAGGACATGCTGATCAAGGACGTC
AATGTGACGGCGCCCGGGGACAGCCCCAACACGGACGGCATCCACATGGGCGACTCGTCC
GGGGTCACCATCACCAACACCGTCATCGGCGTCGGCGACGACTGCATCTCCATCGGCCCC
GGGACCTCCAAGGTGAACATCACCGGCGTGACCTGCGGCCCCGGCCACGGCATCAGCATC
GGCAGCCTAGGGCGGTACAAGGACGAGAAGGACGTCACGGATATCAACGTCAAGGACTGC
ACGCTTAAGAAGACGGCCAATGGCGTCCGCATCAAGGCATATGAGGACGCTGCCTCCGTG
CTCACCGCCTCCAAGATCCACTATGAGAATATCAAGATGGAGGACTCGGGCTACCCCATC
ATCATCGACATGAAGTACTGCCCCAACAAGTTATGCACCGCCAATGGTGCCTCCAAGGTC
ACCGTCAAGGACGTCACCTTCAAGAACATCACCGGCACCTCCTCCACCCCGGAGGCCGTC
AACCTGCTCTGCTCCGCCAAGATCCCATGCACCGGTGTCACCATGGACGACGTCAACATC
AACTACAGTGGCACCAACAACAAGACCATGGCTGTATGCAAGAACGCTAAGGGCAGCGCC
AAGGGTTGCCTGAAGGCGCTCGCATGCTTCTAG
>ZmPG35
ATGGCGTGTATAGACAATGCAATGAGAGCCTTGTTCCTTTTAGCGCTCTTCTGTGTCGTG
CATGGTGAGAAGGCAAAGTCAAAGGACAACGATGCAAAAGCGTCCGGGCCCGGTGGGTCC
TTCGACATCACCAAGTTGGGCGCCTCCGGCAATGGCAAGACGGATAGCACGAAGGCTGTG
CAGGAGGCGTGGGCATCAGCGTGCGGCGGCACCGGGAAGCAGACGATCCTCATCCCCAAG
GGCGACTTCCTCGTCGGACCACTCAACTTCACAGGCCCATGCAAGGGCGACGTGACCATC
CAGGTGAATGGCAATCTGCTGGCGACCACGGACCTAAGCCAGTACAAGGATCATGGTAAT
TGGATCGAGATTCTACGCGTGGACAACCTTGTCATCACCGGCAAGGGAAAGCTCGACGGG
CAGGGGCCAGCCGTGTGGAGCAAGAACTCCTGCGTCAAGAAGTACGACTGCAAGATCCTT

CCCAACTCGCTGGTGATGGACTTCGTGAACAACGGGGAGGTGTCCGGGATCACGCTGCTC
AACTCCAAGTTCTTCCACATGAACATGTACAAGTGCAAGGACATGCTGATCAAGGACGTC
AATGTGACGGCGCCCGGGGACAGCCCCAACACGGACGGCATCCACATGGGCGACTCGTCC
GGGGTCACCATCACCAACACCGTCATCGGCGTCGGCGACGACTGCATCTCCATCGGCCCC
GGGACCTCCAAGGTGAACATCACCGGCGTGACCTGCGGCCCCGGCCACGGCATCAGCATC
GGCAGCCTAGGGCGGTACAAGGACGAGAAGGACGTCACGGATATCAACGTCAAGGACTGC
ACGCTTAAGAAGACGGCCAACGGCGTCCGCATCAAGGCGTATGAGGACGCCGCCTCCGTG
CTCACCGCCTCCAAGATCCACTATGAGAATATCAAGATGGAGGACTCGGGCTACCCCATC
ATCATCGACATGAAGTACTGCCCCAACAAGTTATGCACCGCCAATGGTGCCTCCAAGGTC
ACCGTCAAGGACGTCACCTTCAAGAACATCACCGGCACCTCCTCCACCCCGGAGGCCGTC
AACCTGCTCTGCTCTGCCAAGATCCCATGCACCGGCGTCACCATGGACGACGTCAACATC
AAGTACAGTGGCACCAACAACAAGACCATGGCCGTATGCAAGAACGCTAAGGGCAGCGCC
AAGGGTTGCCTCAAGGAGCTCGCATGCTTCTAG
>ZmPG39
ATGATCCCCAAGGGCGACTACCTTGCTGGTCCCCTCAACTTCTCCGGGCCGTGCACGTTG
ACAACCTCGTCATCACCGGCAAGGGCACCCTCGACGGCCAGGGCAAAGAAGTGTGGGACA
ACAACAAATGTGCCAAAAAATACGACTGCAAGATCCTGCCCAACGTGAGTACTCACTCTA
ATGGCTGGCCATCGTTGCCTCAGTCTCGATCCACCCGGCACTAAACGTACGGTGCATGCC
ACATTATTAACTACTGCAGTCGCTGGTGCTGGACTTCGTGAACAACGGCACCGTCTCCGG
GATCACCCTGCTGAACGCCAAGTTCTTCCACATGAACGTGTTCCAGTGCAAAGACATGAC
GATCAAGGACGTGACCATCACCGCGCCGGAGGACAGCCCCAACACCGACGGCATCCACAT
CGGCGACTCCTCCGAGGTCACAATCTCCGGCACCACCATCGGCACGGGTGACGACTGCAT
CTCCATCGGCCCCGGCAGCAGCGGGATCAACATCACCGGCGTCACCTGCGGCCCCGGCGT
CGGCAGCCTGGGCAGGTACAAGGACGAGAAGGACGTGACGGACGTCAACGTCAAGGACTG
CACGCTCAAGAAGACCAGCAACGGCGTCCGGATCAAGGCCTACGAGGACGCCGCCAGCGT
GCTCACCGCCTCCAAGCTCCACTACGAGAACATAGCCATGGAGGACGTGGCCAACCCCGT
CATCATCGACATGAAGTACTGCCCCAACAAGATCTGCACCGCCAAGGGCGATTCCAAGGT
CACCGTCAAGGACGTCACCTTCAAAAACATCACCGGCACCTCGTCCACCCCCTCCGCTGT
CAGCCTGCTCTGCTCCGACAAGATCCCCTGCAGCGACATCACCATGGACAAAGTCAAGGT
CGAGTACAAGGGAACCAACAACAAGACTATGGCGGTCTGTAACAACGCCAAGGGAAGCGC
CACCAGTTGCCTCAAGGAACTGGCATGCCTCTGA
>ZmPG48
GCAATCTTCCGGAACATCCATCGATCTCCCCCAGCGGCGAGGAGAGCCGGCGGCCACAG
GAAGGAGAAATGGCTTCCGCACACAACGCTCTCCGGGTGTTTTTCATCCTAGCCGTGGTA
TGTGCCGTATGCACAGCGAAAAGGACAGGAGCCAACAAGGAAGAATCGGCGGCAGCCCCC
GGTGGCGCTGCTGGAGGCAGCGGCGGGACGTTCGACATCTCCAAGCTCGGCGCGACCAGC
GACGGCAAGACGGACTGCACAAAGGTACGTAAATAGTACTAAGTGCCAATGTACGCCATG
ATGCGTTGCAGATAGATATCATGTCGTTCTCAGATCGGTGACGGTGCAGGCAGTCCAGGA
CGCGTGGACGTCAGCGTGCGAAGCGACCGGAAGCGCCACGGTGGTGATCCCCAAGGGCGA
CTACCTGGTCGGCCCTCTCAACTTCACTGGGCCATGCAAGGGGAGCAACATCGCCATCCA
GCTGGATGGCAACCTGCTGGGATCAAACGACCTGGACAAGTACACGGCGAGCTGGATCGA
ATTGTCTCACGTTAACAACATCGGGATCACCGGCTCGGGCACGCTGGACGGCCAGGGGAC
CGCCGTTTATAGCAAGAGCAAGACCGACAACGTGAAGGCGATGCCCAACGTACGTACTTT
CGTCATGCATGCACATTATTAATTAACTGTTGTTATTAAAAACGACAGCTAGCGTCCTCG

ATCGATCATGATGTGATTGCAGACACTGGTGCTGTTTCACGTGATCAACGCCACTGTCGC
CGGAATCAAACTACTCAACTCCAAGTTCTTCCACATCAACATCGACAACTCAGAGAGCAT
CACCGTGAAGGACGTGAACATCACCGCGCCCGCCGACGTTGAGAACACGGACGGCGTCCA
CGTCGGAGGCTCCTCCAAGATCAGCATCCTCAACTCGACCATCGGCACCGGCGACGACTG
CGTCTCGATTGGGCCCGGGTGCAACGGCGTCTTGGTGGACAGCATCACCTGCGGCCCCGG
GCAGGGCATCAGCGTCGGCTGCCTAGGCCGCTACAAGGACGAGAAGGACGTGAGCGACAT
CACGGTGCGGAACTGCGTGCTCAAGAACACCACCAACGGCGTGCGCATCAAGTCGTACGT
GGACGCCGAGTCCGTGCTGACGGCCTCCCATCTCACCTTCGAGAACATCAGGATGGAGGA
GGTGGCCAACCCCATCGTCATCGACCAGTACTTCTGCCCGCAGAAGGTATGCCCTGGCAA
GCGGAGCAACTCCTCGCATGTCTCCGTCAAGGACGTCACGTTCCGCAACATCACCGGCAC
GTCGTCCACGCCCGAGGCCATCAGCCTGCTCTGCTCGGAGACGCAGCCATGCAGCGGCGT
CTCCCTCATCGATGTCAACGTGGAGTACGCCGGCAAGAACAACAAAACCATGGCCGTCTG
CAGCAACGCCAAGGGCACCGCCAAGGGAAGCATCGAGGCACTGGCTTGCCTGGTCTGATG
ATGACCTTCCTTTTGCATGCATGCATGGTATCTCATCCTTGATGATGATCTAGCTCACTA
GTTCTTTTTAATTTCCGCTTCATTCGCTTTTCCAAATTCGATTGTGTTTCAGCCAAGTTG
TTTAGCGGGACATCTCTTGTCTGATCTTCCTGCTAAATAGAGTTGGACTCCTATATATAG
AGGCCTTCCGGCACATATAATATACGTCGGACCAATATTATTGTGATCACTAGTTATGTC
TCTATATGTTGCGACGGGGATAAAAAATTTGAATGAAACATAGATACTGAACGACAAACA
TCACCATGATATATAAAATTTACGTGGCAAACATTATCAATATCGTGTTGAGTAAGCAGC
ATCCAGTCTGTAATCGTGTCAATGTTTGTCAGCATATAGGTGTGCATTAGTTAGCTTGTT
TATGGCTTAATCATATTTGTAATAGTGCGTAGAATCATGGGCATGCAGGAGATTGCACTC
TGCGTGCCATGACCACGTCAAGGGCCACTTCCCCGTCATGCAGTGCCGACCGTGGATGTC
GCGCGACGAGTCTAGAAAAGCTTGATCGTTCAGCTGCTCAGCATGGCGGGGGAAGCGGAT
CTCATGCCATTTATATTTACAAGTAATGCACCGAAAAGGTTGCGCGATTTGCAAC
>ZmPG10
AGGATATCTTCGGGAACATCCATCCATCCCCAATCCCCAAAGGCGAGGAGAGAACTAGCA
AAGGGGAAATGGCTGCTTCCACAAACAACACTCTCAGGGTGCTGTTCACCCTAATGGTTG
TATGCGCCGCAGTATGCACAGCGAAAAGGACTGTAGCAAAGGCAGGAGACTTGGCGCCAG
CCCCTGCTCCGTTAGGAGCAGGAGGCGCCACCGCAGCCCCAGAAGGTGCGGCTAGAGCCA
GCAGGACGTTCGACATATCGAAGTTCGGCGCGACCAGCGACGGCAAGACGGACTCGACAC
AGGTTGCATTGCATTGCATTGCATTGCATTGCATGTTGGCACGGTGGTGTGACTGATGCA
CTGGTTCAATGATCTATCAGGCAGTCCAGGACACGTGGACGTCAGCGTGCGGAGCGATGG
GAGACGCAACGATGCTCATCCCCAAGGGCGACTACCTGGTGGGCCCTCTCTACTTCAGTG
GCCCGTGCATGGGGGACATCACCATCCAGCTCGATGGCATCCTGCTGGGATCCAACGACC
TGGCCAAGTACACGGCGAGTTGGATCCAGGTGTCGCACGTTAACAACATCGTCATCAACG
GCTCCGGCACGCTCGATGGCCAGGGCACCGCAGTATATACCAAGAGCAAGACCGACGTCG
TCAAGGCGATGCCCAACGTACGTATGCGTGCATGCATGCATGTGCATTAATCTATTATAT
AACAATTAATGCCACGTACGGACTCATGCATGATGCAGACACTAGTGTTGTTCTACGTGA
CCAACGGCACTGTTTCCGGAATTAAACTACTCAACTCCAAATTCTTCCACATCAACATCG
ACACTTCTAAGGACATCACCGTGAAGGATGTGAACATCACGGCGCCCGCGGACGTCGAGA
ACACGGACGGCGTCCATGTCGGCGGGTCCTCCAATGTACGCATCACCAACTCGACCATTG
GCACCGGCGACGACTGCGTCTCCATCGGCCCCGGGAGCGACGGTGTCATGGTGAACAACA
TCACCTGTGGCCCCGGGCAGGGCATCAGTGTGGGCTGCCTAGGCCGCTACAAGGATGAGA
AGGACGTGAGCGACGTGACGGTGCGGGATTGCGTGCTTAGGAACACCACCAACGGCGTGC

GCATCAAGTCGTACGAGGATGCCGAGTCTGTGCTCACGGCGTCCCATCTCACCTTCGAGA
ACATCAGGATGGAGGAGGTGGCCAACCCCATCATCATCGACCAGTACTTCTGCCCCCAGA
AGGTCTGCCCCGGCAAGCGAAGCAACTCCTCGCATGTCTCCATCAAGAACGTCACCTTCC
GCAACATCTCCGGCACGTCGTCCACGCCCGAAGCCGTCAGCCTGCTCTGCTCCGAGACGC
AGCCGTGCAGCGGCGTCTCGCTCATCGATGTCAACGTCGAGTACGCCGGCAAGAACAACA
AGACCATGGCTGTGTGCAGCAACGCCAAGGGAACCGCCAAGGGAAGCCTCCAGGCCCTGG
ATTGCCTCGTCTGATCGATGACCTTGCATTTGCATGCATTCTTCTTCCGTTTCACTTTTC
CATATTCTATTGCGTTCCAGCCAAGCATCTTGTTTCTTTTATATATATATATATATATAT
ATATATATATATATATATATATATATATATATATATATATATATATATATATGCAGGCAA
TTATATTAATGGTGGAGACGACACATATATCTGGGCGTTTGTCTGCCTTCCCAGCTGGTT
TAGAGCACTGTAATATTATTAGATATAACAGGGCGTTATGTTGTATTTTTACATCACTAT
ACCTTACCATGAGGTCCTTTCTCCTCTGTGAGGGGTCAGGCATTGTATTGGAACCTTGTA
CGGCGTTTGCCTTTTTAATGCAATGACATACACTACTCGCACC
>ZmPG15
ACGGCTCCGGCGCGGCACGCACCCTACAGCACGAGACATATCGATGGCGTTCATCAGCAG
TGCGGCGATGAAGGCAGCCGTGGCCGCTCTGCTGGTGTTCGCCGCGGTGTCGCCTGCCGC
GCGCGCGGTGGCGGCGGAGGCGGAGGCGAAGGCGAAGGCTGTGGGAGGCGCGCCGTCGGT
GCCCGCTGGCTCGCTGGACATCGCGCAGCTGGGCGCCAAGGGCGACGGCAAGTCGGACAG
CACCCCGATGGTGCTCAAGGCGTGGAAGCACGCGTGCGAGGCGACGGGGCAGCAGAAGAT
CGTCATCCCCAAGGGCAACTACCTGACGGGCGCGCTGGACCTGGTGGGCCCCTGCAAGTC
CTCCATCATCATCCGCCTCGACGGCAACCTGCTCGGCACCGGCGACCTCAACGCGTACAA
GAGGAACTGGATCGAGATCCAGAACGTCGACAACCTGTCCATCAACGGCCACGGCACCAT
CGACGGGCAGGGAGCCCTGGTGTGGAACAAGAACGAGTGCCAGCGTTCCTACAACTGCAA
AATCCTCCCCAACGTACGTGCTTCTTCTTTCTCTGTCTGTCCCCTGCTTCGGAAGGTGAT
GTGATGTAAATCGCAACGGCGAACGCGTGCAGAGCCTGGTGCTGGACTTCGTGACGAACG
CCCAGATCCGCGGCATCACGCTGCTGAACAGCAAGTTCTTCCACATGAACATCTTCGGGA
GCAAGAACGTGGTGATCGACAAGGTGACGATCAAGGCCCCCGGCAACAGCCCCAACACGG
ACGGCATCCACATCGGCGACTCGAGCAACGTGACCATCAGCGGCACCACCATCGCCGTCG
GCGACGACTGCGTCTCCATCGGCCCCGGGAGCAAGACCATCCGCGTGAAGGGCGTCAAGT
GCGGCCCGGGCCACGGCATCAGCGTCGGCAGCCTGGGGCGGTACAAGGACGAGAAGGACG
TGGAGGACGTGAAGGTGACGGGGTGCACGCTCGCCGGCACCACCAACGGCCTGCGCATCA
AGTCGTACGAGGACTCCAAGTCGTCGCTCAAGGCCACCAAGTTCCTGTACCAGGACGTCA
CCATGGACAACGTCTCCTACCCCATCATCATAGACCAGAAGTACTGCCCCAACAACATCT
GCGTCAAGTCCGGCGCCTCCAAGGTGGCCGTCAACGACGTCGTCTTCAAGAACATCCACG
GCACCTCCAACACGCCGGAGGCCATCACGCTCAACTGCGCCAACAACCTGCCCTGCCAGG
GCGTGCAGCTCATCAACGTCGACATCAAGTACAACAGGTCCGACAACAAGACCATGTCCG
TCTGCAAGAACGCCATCGGCAAGTCCATTGGCATGGCGAAGGAGCTCGCCTGCGTCTGAA
CCTACTTGCATCCATCACTCACTCTTCGTCACCTCTCTCTTTCTCACTCTCGCCAGTCTT
TTTTTAGGCCTCTGGCAATCTGCGAACTTTCTTATTCATTCTACTAGTGTGGATCTATAA
TTCCATTCAAAATATATACATGATCTAAATTTCATGCCACAGAGGACATTGCAATGTACA
AGTTTAAGAACACAACCCAACTCTTAAATAGAGGGAGACATCGTATAAAATGGTGAAGCG
GTTTTATTGCACAACAGAGCATCAGTCTCCTCATCTTCAACAGCCCAGGAAAACAACCCA
TATTCATATTGTTCAGTTACATAGCAGCCTTGCTAGATAGCGGCATGAACTCTTTGATAG
TTGAGACCGTTGAGACAACTCTCTGATAGCTGAGATCGTTGAGACTCCGGAGAACTGATG

CCAAGACGTGTTGGCTCAGGGAAGTGCCGGCAGAACCTTCCCGGTGCAGGTTCCAAAACC
AATGTTGTAGCCTTCACCCTGAAGAACCAGCGGATGGTGGAATCAAAATACAGCCTAGCG
TCACTGTAGAGGCCTAGATCTGAATTGATCAGGAAAGACGAACTCACAGTACCTTGCAAC
ATCCTGTCAAGATGACTTCATCTCCATCTTCTAGGAACTTGCGGGTCAAATTCCCCACCG
GTATCTCCTTCTGCCCGTTCCATGTCAGCTCCAGCAGACACCCGAGGGAGTCCGGTTCCT
GAAATGGCACATGTCAAAGTAAGATTCATACTATGGGTATAGAAGTTGCAATGTTTACTC
CATACAGGTCCACTCAGTGTGCCAGTCGCAAATATATCCCCCGGCCTCATGTTGCATCCG
TTGATGGTGTGGTGTGCTAGCTGCTGTGTCAGCGTCCAATACCTGTGCAAAAATTGGTTA
CAAGAAATGTGCCAGCACTGTTATGTGCTATGGCAAAACATTATATGGTTGAATAGTTGG
GATCCTATTAACACACATTAGTATTTCTTTTTCTCGAACGGTGCAGGAGCACACGTTAGT
ATTTAAAGTGAAAAAAAAACGAATATGCAGTGTGCTTACAGATGCTTGAAATTAGTTTTT
GTGACAATTGATGCGTCATTTTGATCTTTCGGCTTAATCCAGGCCTTTCAAAGGAGAAAA
AAAGTGTCCCAGTTAGATCTCTTTTTATGACATAAATAATAGCGAAGAAGAAGCTGCAAG
GTAGCAATGTCATACTTCAAGAGGAATGTCATAATTTATGTGATTCTTTTCGGCTAAGTA
CGGTAAAGGCTCGGGTTCCTGTAATGCAAGGAGAATTAGGTCACCATGGATCGAAGTTCT
ATAAAATGCTGATTAGAAAAACTAACCAAACTGACCGGTGTTAACTAAGCGCTAGTGATT
AACTGATTATTCCAACGATAAATCTTAAAGAATGTTTCCTCACCTGCTTAGGAGCATCAC
ACATGAAAGGCTTCAAAGCATCTAGGGTAACAATCCATGGTGATACCGTGGTACCTAGAG
AAATTGTAGAAGCACAGTACATTTTCAGGTCACAACTGAAATATGGAGCCCAAAAGGGAA
AATGACGGAAGTGCATATGTTAAGCTTGCAGGACTTCTAACTTTTTTTGTTTATAAAGCA
TGGCCCTTAAGATTTGTTTAGAAAACCTAAAGTTTCATTAAGGGCATCAAGTGTCTTACT
GAAGCTTTTCCCAAGGAAAGGTCCAAGAGGTATAGTCTCCCAAGCCTGGATATCTCTGGC
TGGCATATTACATGCAACTCAGTATTAAACACATCATGGATGTTAATCTTAAACAGCTGA
AAATTGAAAAGAACAGAATACCACTCCAATCATTCATCAAAGTTAGGCCAAAAATATGTT
CTTCGGCGTCATTAATATCAATAGGTTTGCCTAATTCATTCCCTGGACCAACAATGGCAG
CCTGAAAGCAACAAGCCAGCAATTATTATGATATAATACAGTGAATAAAGAAAATTGTCA
CTGAAATAAACCACAGTTACCTATACTAGTCCCATAATTGATAATCATATACCAATGATA
ACTGAAAGTGTGTGGGGAAAATAATAATAGCTGAAAGTGACCTTACCGCTTAATGAGAAA
AAAACAATTACTGAAAGTGACTCGACAGCTTAATTAGGTAGGTAGGCCAATTACAACCAC
AAAAGAGAGTAAGTACATGGGGTCTAGGGTTAGTAAGAAGCTAACCATCTCAAGCTCAAA
ATCAAGCTTCTGCGAGGGACCAAAATAAGGAGCGGAGTTTCCTGTTGGATGTCCTTGTCC
CCTAAAAATAGAGAAATAATTAAACAAAAGAGCAACAGGCAGCACACAAAATAAAGGTTT
CAAGCTTGAAACAACAGTTAAGACTCGTTTTAACAAGTATTTTGGGATGCACACCTGGGC
CTAATGACATTGGTTCCAGACACAACTATAGATGATGCTCGCCCATTGTATCCTACTGGA
AGATAAAACCTGAATTGACAAAATGTACATTGTAAGTTAGTTACAACTGGGTCACATCAA
TGAAGTAACGAGAACAAAAGTAGAGTAAACAATGGCATGCAGCAAAGCATTTGAGATATT
TAATCATTACCATGAAGATTGAGCATAGTAACATAACAACCAGCTCATGACTACAGTAAC
TTAGCAAGAATATTCAGACAACAGGATCTGATCTCAACTGATTAACATACTGCTTTTGCT
CCCCTAAATGCCAACAAAGCACATTATCCGAGAAAACACATACCAATTTGGATTGACTGG
AGTCTGCGGCCCTCGGAAGATGAATCCACAATTCATTGCATGGTGCACAGAACAAAAGAA
GTCCGTGTAACCTCCAACCGTGATTGGTAGAACCATCTCTATATCACTCTGCATTGCCAA
AGCAGAGCATTGGCGATGATTTAGTCAATGATCCTTTGTTATTACCCTCAGAAGAGACGG
GAAGACAGACTAAAAAAAACTCACCATTGGTACCAGACACTTGTTCCTCAGTGCTTCGTT
GTCACGCAAGACGGGCTCGTCGGCTGCAAAGCAACAAAGAAAAAGAATGAAAAAAAACAA

GCACATTGTTCTTACTTCTTCTTTATATTGATACTTTTCCTAATTGTGTGGCTAAACAGA
AGAAGACTCGGAACCTGATAGGATTTTCTGTAGCGTGGCGCGCGCCTCCCTCCACGCCGG
CCGGCCCATCCCCAAGAACATGTTGAGCGTCTCCTGCATGCAGCACCAAGGACACCAGTT
ATTTTCTACTCTATAGTCACTACTCACTACCAGACCAGTTTCCTTAGATCCTCAATTAAC
TATTTCCACGCTGCAACCGATACCAACACCCATTCACGAGTCGGAGAAGCTAAAATCAAG
CTCATACCATCCGTCCGTCCGTCACTGCAGAGCAATCAAAACAAATCAAAGGGTTGAGGA
GCAGAGCAGGCACCTGGTGGAAGCACGGGGAGCCGGAGAGCGCGGGCCCGTCGAAGAGCC
CCGCGTCGGCGACGGCGGCGAGGTCGAGCGCGAAGTCCCCGATGGCCACCGCGGGGCGCG
GCGCCTGCGCCTGCACCCCCCGCCGGCGGAAGACCCCGAAGGGCAGGTTCTGGATGGGGA
AGTGGGAGCCCGCCGGCACCTCCACGAACGACCGCAGCTGCTGGCTCGGCTCCGCCATTG
CTCCCTCCTCCGCTTTCTCGTGGGTGGTGTTTGGTTTGCACTTTGCAGTTTACTGGTGGT
CACCACTCTCGATCGCCTTGTGCTGGTGCTGGCGGCTGGCCACAAGCGACAACAACCCCG
CCGTGAAGACTAGCACTCCTGTATAAACAGACACCCTGTCTATTTATAAGCTGATAGAGC
ATCTCCAACAAACATTATTAATATGCCATCACCATCCGTATATCCCAAATCTACAATTTC
TCACTTCTTCACAAATCTATTTGCATGCGTGCTCTCCTAAAAAACGCTCTCTCGTTTACA
CGCTCCCATTGATACTGTTCAAAATACATATCTGCATACGGTGACTATTAGAGATAGTCC
GTAGTATTGATGTATCCTCTTAGATCTCATCATAGCCCATCCGACCCGACTCGATCCGAT
TAATGCAACGGATATTTTGACCCGCAATAATTTACAGGCTGGTATAGGCCGTGGTTTTTT
TTGACTCAAACCCCAACCCAATTCAAAAAAATCCGACCAAAAGCCAAAAAGCAGACCTGA
AAAAACCCTGGCTTGAAACGTCAAACAGAAAGACACGAGCTAACCCGATCTGACCCAAGA
CTAGCTAGTCATAGGTCGAGTACGGGCCGTAGTAAATGGCCCACAATCCAACCTTACCCG
ACCTGAACTCAATCCGACGTTTGAACGGGTCTGATACACAATAATGTGGATAGATATTAA
AGTTTAGAAATATTGGGTATCACGAGTCGAGGGCAACAAGTTACACATATGATATTCGTA
TTTGTCCTAATCTATATGGTTTGGGATAGATTGACATGAAAATTAATCAATTTCTACAAT
AACCCGTTCCAACATGGAGATTAGTTTGAATGCAATCTTAGTTTTTTTGTTTTACGTTGT
CGTTAGGGATAAAAGTGGATATCCAAATTGTTCAAATAAATTTAATATTTTAAGTAGATA
TACATAAAATTCGATGTTGGTATTTTCTTATGTTATCCAGCATATTATTATAAATAAGAA
TAACATTTTGTATAAATTGTCTTATGCATTGTTTGTTCTCTACAACAACAAAAAATGTGA
ATAAATATCGAAATTTGTATCCGAATTTTATATCTATCATTTGAGAAAATATATGATAAA
TTTGAGGTTTATCTTT
>ZmPG25
CTCCAGCAACGTGACCATCAGCAGCACCACCATCGGCGTCGGCGACGACTGCATCTCCAT
CGGCCCCGGGAGCAAGATGATCCGCATCCATGGCGTCAAGTGCGGCCCAGGCCACGGCAT
CAGTGTCGGCAGCCTGGGGCGCTACAAGGACGAGAAGGACGTGGAAGACGTGAAGGTGAC
GGGGTGCACGCTCGCCGGCACCACCAACGGCCTGCGCATCAAGTCGTACGAGGACTCCAA
GTCGTCGCCCAAGGCCAGCAAGTTCCTATACGAGGGCATCACCATGGACAATGTCTCCTA
CCCCATCATCATCGACCAGAAGTACTGCCCCCAACAACATCTGCGTCAAGTCCGGCGCCT
CCAAGGTGGCCGTCAACGACGTCGTCTTCAAGAACATCCACGGCACCTCCAACACGCCGG
AGGCCATCACGCTCAACTGCGCCAACAACCTGCCATGCGAGGGCGTGCAGCTCGTCAACA
TCGACATCAAGTACAATGGATCCGGCAACAAGACCATGGCCGTCTGCAAGAACGCCATCG
GCAAGTCCATCGGCTTGGCAAAGGAGCTCGCCTGCATCTGAACCAATTAACTAACATTTT
CATATATTATATATATAATCACTCTTCGTAACCTCTCTCTTTCTCACGTACTCTCGAGTC
T
>ZmPG20

ATGGAAGCCCTAAAGAAAGCAGAGGACACCAACGTCTCGCCGCGATGCTTGGCCATACCG
AACATAGCATTCCACCCATCTTGGCATCGCAGTATATATGTGTGTTCGAGCGCACCACCA
CAATCACGACACCGGCACCAATCTGCCATCCTCTCCATCACGAAGATGGCGTTCATCAGC
AATGTCGCAGTGAAGGCGGCGGGCGTGGCCGCGCTGCTTGTGGTCGCAGTGGTGTCGCCT
GCCGCGCGCGCGGCGGCGGTGGCGGTGGCGGGAGGGGCGCCGTCGGTTCCGGCGGGTCCG
CTGGACATCGCGCAGCTGGGCGCCAAGGGCGACGGCAAGTCGGACAGCACCCCGATGATC
CTCAAGGCGTGGAAGAACGCGTGCGAGGCGACGGGGGTACAGAAGATCGTCATCCCGCCG
GGCAACTACCTGACGGGCGGGCTGGAGCTGAAGGGCCCCTGCAAGTCCTCCATCATCATC
CGTCTCGACGGCAACCTGCTCGGCACCGGCGACCTCAGCGCGTACCAAAGGAACTGGATC
GAGATCGAGAACGTCGAGAACCTGTCCATCAACGGCCACGGCACCATCGACGGGCAGGGA
GCCCTGGTGTGGAGCAAGAACCAGTGCCAGCATTCTTACAATTGCAAGATCCTCCCGAAT
GTACGTATAGACACTTGTTTTCTTTGTCTCTGTCTCCTCTTCTCGATAATATTGATAATT
GATTGAAATTGCATGCATGCAATGCCATTGCGATGGATATACAGAGCTTGGTGCTGGATT
TTGTGACGAACGTCCAGATCCGCGGCATCACGCTGCTCAACAGCAAGTTCTTCCACCTCA
ACATCTTCGAGTGCAAGAACGTGCTGATCGACAAAGTGACGGTCAAGGCCCCCGGCGACA
GCCCCAACACGGACGGCATCCACATCGGCGACTCCAGCAACGTGACCATCAGCAGCACCA
CCATCGGCGTCGGCGACGACTGCATCTCCATCGGCCCCGGGAGCAAGATGATCCGCATCC
ATGGCGTCAAGTGCGGCCCAGGCCACGGCATCAGCGTCGGCAGCCTGGGGCGCTACAAGG
ACGAGAAGGACGTGGAAGACGTGCAGGTGACGGGGTGCACGATCGCCGGCACCACGAACG
GCCTGCGCATCAAGTCGTACGAGGACTCCAAGTCGTCGCTCAAGGCCAGCAAGTTCCTGT
ACGAGGGCATCACCATGGACAATGTCTCCTACCCCATCATCATCGACCAGAAGTACTGCC
CCAACAACATCTGCGTCAAGTCCGGCGCCTCCAAGGTGGCCGTCAACGACGTCGTCTTCA
AGAACATCCACGGCACCTCCAACACGCCGGAGGCCATCACGCTCAACTGCGCCAACAACC
TGCCATGCCAGGGCGTGCAGCTCGTCAACGTCGACATCAAGTACAATGGATCCGGCAACA
AGACCATGGCCGTCTGCAAGAACGCCATCGGCAAGTCCATCGGCTTGGCAAAGGAGCTCG
CCTGCATCTGAACCAATTGACTAACATGCATATATTATATACTAGGTATATGCCCGTGCA
TTGCAACGGAAAACACTTGTGTATACACAGGTCATTTTTTAGTAATGCTATCAAGAATCA
ACTCATAAATCATAACAAATGTCGAAGTCTATCATTTGAAACAACCAAAATTTGACTCAA
TGCCATTATTAATCGAAATAGACAAATCCTAGGTTGATAATATAGGATATGATGATGTAC
CATGGCCTTCATAGAACAATTTTGTATTGTAGGGTGAAAAATTATAAAAAGCTATATTCT
AAGCATATTGAAAGGGATGGTGAGTTGTGATTATCAAAATTCATGTTCAACTCAAGTTCA
CACAATTGCCAGGCCTCTATTGTACATACTTGTTCTCAAGCAACAAAATATATATATATA
CTCAGGTTAGACAGGGATCAACTCAAGTTCACACAATTGACAAGCTTCTAGTGCACATAC
ATGTTCTCAAGCAATAAAAAGTATATATACTCCAAATACATGAAAAACACATACTTAACC
CATGAAGTCTTTTATACATCCTTGGATTGGAATTGCAAGACACTTCCAACAAGAGTGGAT
ACATACTCAACACATACAATCTTTAACTTTAATAAATTAATAAAGCAATCTGTAAATACT
CAAAGTTCTCATGGGACTGCATAAATAAAACATATAGTAATTAGAGCCAAATCCTCTCTT
AGTCTATTATCATGGTCTTATACCGTGTCTTGGCAACTCTTCTAGGATAAAAGGGGTAAA
AATTGGTTATAACTGCATATTCAACTCCAGAGGGGCCACTCCTGCAAAGCATTATAAAAG
TGAAAGATTCTAATCAGTAATATTCTTAGAAATGATGTCAGTTCACAAATATGGTGTGGA
TAATTTCAGCTACAAATTTCTACAATCTTGAAAACACATTTGTAACTAAAATACAGTTGG
TCTTGAAGTAGTAACTAAATACAGTGGGCATCAACATATGTTCTTAAAAGTTCTTGTGTC
ACAATATAAAACATGTAGCTAGAATCAGGTTTAAGAAACTACCTGCAGGTTTAAGATGCT
ATATTTCCAATTTACTGATTTAGATGTCTCATTATTTTTTCACCCATATGGCAGCAACAA

AATGAACTAAGCTAATTTTCTCCCCTGGTAATTAGACATATGAGATGCGATTGAACTAAG
CTATTTTTCTCCCCTAGTAGTATAAAAGATTTACAAAAACAAACACAATTGATATGTTTC
AGGATCACAGTGAGTTTATTTCATAGTGCCGAAATAAAATATCCATTTCAGTTTCACAAA
TCAATCAGACCGGAAGCATATATTTGACTTTAGCAAATCATTTGAATAAGAACCTGGTAT
GCACCATAGCACCACCACTGCCTACTTATGAATTATTTTCAGCATATATTTCTGTTTTTA
TCGGTTTCTGCAATCAAACAGATTCAGCTTTTGTTTGTGCTCTTAAACAGCTTGTATTTC
CCCAATTTTCTGATTCTGCCAGATTAAGTGCTAGATGTAGCATTTCAGTATCTGTTTCTA
CACTCAAACATAATAGATTCAATTCTATTTGTGGAGTTCACTAAAACCAGAACAAAACAA
TTTCTGTTTGTGCACTCAAACATATTCAGATCTAGTTCCGTTGAGTTCTGTTTGTGCACT
CAAACAAAATAGAACAATTTCTATTTTTGCAGTTCAGTTCAACAGATTCATTGAAAACAG
AAATGGTGGTTTCATCAGTGAAAACAGAAATGCACTGACAACAAAAAACAGAAACACACA
TTCACTTAAACAAGTAGTTCACAACCAGAATTCAGTTTACTTTTCACAAGTAGTACTCAT
ACCAGAATTCCACTGATAACATGAGTTGAATACAAATCATAAGTTCACAACCACAACTCA
CTACAAATGTCACATAGTACAATCCCACCACTCTTCGAAGCGAATGAGGTGATGGAACAA
TGGAAATGACGATCGAGTTGTGTGTGCAAAATGCAAAGCAGAGCCAAAAAACAAACCTTC
TAGAGCGAGGCGAGTCGATCCATAGAAAGCATTTATAGTTGTTTTTTGCTCATAAAGCAA
CAACAGTTTCATGAAATGTTTATCATCACGTAGCTAGTAGATACGGAGAAAGCATTCCTT
GTATTGTTGCATCTAGAATGGGTGGTGCACAATTTGGTACAAGATGTGGCCAAGGAGCTA
ATCTTCAAATTTTATAGGCTCCCTTTTCTTCCCATAAATAAGGCCAAATTAGCACACATA
AATAACTTATACAACAACCTATTTGTCAAACAAATCGTCATATGGCATCTAACATATAGT
TGTCAGTGTAGGTACCTATGGATCAAGAATTGGTTTTCTCTTCTCAACATAACGATGAGG
ATATGAGGTAGCAGCCAAAGCGGCTCGCTCCTGGGGGAAAAAGGAATATAATATGTTTAG
TGAATGGAATGTCAAAATAGAGGATTGTTTGAAACACATAAATCTATAGCCCATGAATTG
GAAGAATCAAGTAGTAAAAATCATCATTAATGTACGACAATAGATCGATTAATAAACATA
TCATTAAAAGATAATTATATATACATGGTGACATACTGATGCTACACAGAATCAGTTCAA
ACAAAAAAAAGACTTTGTTAAACTTTATTAGGATTCTTTTGTTGTATTATACATATAGGA
TTCTTTTGTTGTATTATACATATTTGTTCAAATAAAAAGTATTGCATCGTACAATGCCCA
TGCATTACGATGACACACAAATCTCAAAATCAACTCTAACAAACCCAAATGTGCTAGTTG
CAGATGATGTAAGAGAAGATTATAGTAGCAACTAATGTAAGAGAAGAGTAATGAAGGCGA
AACCAAACAAACACTGAAAGATATGCTTTGTCAAGGTCTTGTGCGACATGAACTCTGCGA
TGAAAAGCACTAAATGACATGAACTCTGCGATGAAAAGACCTTCTACAGCTGCTGCAGCC
ATTGACCTGGCCTGACTCTTGACAGATGGATTGGCAGGGTCCAGCACGATTGATCTACAA
CCGGAAAAGTTAAGAGGATATATAAGCGGTTGGGAAAAAACTACAATATCATAAAACATG
AAAATAGAGATTCTGCATATCAGGATTATCATAAATCAATAGAAACCAAGAGGATTATAG
AACAGGGAAAGCAGACAAACTTTCGAGATTTTGAGAGACGTTCAGCCTCCTTTGAGCGCA
TTTTCACAAGGTTCTTTGTTGATTCATCCAAAATGCGTTGCACATTCACCTTGCGCCAAG
CAGAGCCACACTCCAGAAGCAACCCATTTCCATCCTCTCCACTCCGACGTTCTTCATAAA
TGTCACACAATTCGCCTCGTTCAGATGCCCAGGTGAATTTGAATTCTTTACCACTAACAT
CTATAACCTGGCACAAGAAAAGAGATTTGAGAGCTCCAATTGAAACACTAAATGTACAAG
TGCAATCATCATGTTGAAATAAGTTCTCATGTCAAACAAATCCAGTTTGGATCTAGTAAG
GCACTTTAAGTAAGCATTGTAACCTTGAAATTCTTTAAAGCATCCTTCACAGTTCACTCA
GCCTCCACGATTTCAGTTTGGTTGGACGTTCAAGACAAAGTGAATAGGCATATTCTTTCT
TGTTTCAACAATTCTGGTCAGTGATCATACTTGTCGGCGATTCCATATATATGGGGAAAA
AAGGTGACTCATAAAATGCAAGAAAAAAATCTGCTAGGAGGGGGTATCCAATCTATGCAG

GGCAGATGGCTCTACTAATACTTCAGAGCTGAGATTTGAACTCCTGATCCACCGTATAAA
GCTAACTGAAATATGACATTATTATGCTAGATATGTCTGAATCACCATTAAGGGAAGTCA
TCCAAAAGTAACAACACCAAATCAAATACAATGTAAGAACTAATTGTACAACAATAAATA
GTGAAAATGTGGACGCGGAT
>ZmPG21
CGCAGTATATATGTGTTCGAGCGCACCACCACAACCACGGCACCGACACGAATCTGCCAT
CCTCTCCAACACGAAGATGGCGTTCATCAGCAATATCGCAGTGAAGGCGGCGGCCGTGGC
CGCGCTGCTGCTGGTCGCAGCGGTGTCGCCTGCCGCGCGCGGCGGCGGTGGCGGTGGC
GGGAGGGGCGCCGTCGGTGCCGGCGGGTCCGCTGGACATCGCGCAGCTGGGCGCCAAGGG
CGACGGCAAGTCGGACAGCACCCCGATGATCCTCAAGGCGTGGAAGAACGCGTGCGAGGC
GACGGGGGTACAGAAGATCGTCATCCCGCCGGGCAACTACCTGACGGGCGGGCTGGAGCT
GAAGGGCCCCTGCAAGTCCTCCATCATCATCCGTCTCGACGGCAACCTGCTCGGCACCGG
CGACCTCAGCGCGTACCAAAGGAACTGGATCGAGATCGAGAACGTCGAGAACCTGTCCAT
CAACGGCCACGGCACCATCGACGGGCAGGGAGCCCTGGTGTGGAGCAAGAACCAGTGCCA
GCATTCTTACAATTGCAAGATCCTCCCGAATGTACGTATAGACACTTGTTTTCTTTGTCT
CTGTCTCCTCTTCTCGATAATATTGATAATTGATTGAAATTGCATGCATGCAATGCCATT
GCGATGGATATACAGAGCTTGGTGCTGGATTTTGTGACGAACGTCCAGATCCGCGGCATC
ACGCTGCTCAACAGCAAGTTCTTCCACCTCAACATCTTCGAGTGCAAGAACGTGCTGATC
GACAAAGTGACGGTCAAGGCCCCCGGCGACAGCCCCAACACGGACGGCATCCACATCGGC
GACTCCAGCAACGTGACCATCAGCAGCACCACCATCGGCGTCGGCGACGACTGCATCTCC
ATCGGCCCCGGGAGCAAGATGATCCGCATCCATGGCGTCAAGTGCGGCCCAGGCCACGGC
ATCAGCGTCGGCAGCCTGGGGCGCTACAAGGACGAGAAGGACGTGGAAGACGTGCAGGTG
ACGGGGTGCACGATCGCCGGCACCACGAACGGCCTGCGCATCAAGTCGTACGAGGACTCC
AAGTCGTCGCTCAAGGCCAGCAAGTTCCTGTACGAGGGCATCACCATGGACAATGTCTCC
TACCCCATCATCATCGACCAGAAGTACTGCCCCAACAACATCTGCGTCAAGTCCGGCGCC
TCCAAGGTGGCCGTCAACGACGTCGTCTTCAAGAACATCCACGGCACCTCCAACACGCCG
GAGGCCATCACGCTCAACTGCGCCAACAACCTGCCATGCCAGGGCGTGCAGCTCGTCAAC
GTCGACATCAAGTACAATGGATCCGGCAACAAGACCATGGCCGTCTGCAAGAATGCCATC
GGCAAGTCCATCGGCTTGGCAAAGGAGCTGGCGTGCATTTGAACCAATTGACTAACATGC
ATATATTATGTACTAGGTTTGTGCCCGTGCGTTGACACGGAAGTTAAAAATTAGTATAAA
ACAAAGATACATAACGATAAATATCACTATGACATTCAAAATCCATGTGGCAAAATATCA
CTGTAACCATCTATGATTGTGCATTGCGACGCCACACAAATTATTCGATAAAATATTAGC
AATTGGGAATAAACCTAGTGCCAAATAACCCTTTATGCAATAACTTCCGTTAATAGCAAT
TGAGAACAAACCTGGTGGCATAACTAACAGCAATGACAGGAAAGTTCAGGAGGATACACC
AACAGGCCGAACTAACTATCCAGTAACATTCCATAACGACCATAAGAAAGCAAAAAGACA
AATAAAATCCTACTGTCCAAAGAAAACATTGCCAGTTGCAGTTAAACGTCTAACCAGGGA
GATCATCAAAGAACTTCATGGACTCATCAAGCTTCTCTTCATGACTGATCTTAACGATCG
AACCAATTGACTAACATTGCATATATTATATACTAGGTTTGTGCACGTGCGTTGCCACGG
AAGTTAAAAATTAGTATAAAACAAAGATACAGAACGACAAATATCTAAGAAAACATTGGT
TGGCAATAGGCATAAGGGGTATTTGAAACACATAAAATGTGCATGTTGTCAAGCTCTTTT
ACATGATCCCCTTCGCCTTCTTGACTTCCATGTTCTACGACCTCAAGCCTTGCCAGGCTT
GGTTTCTTTAAGCTCCTCTAAGCCGCCAACAGTAGGCTCTTCAAAAAAACACCCTCTCCT
AATTTGTCTTGCTTGGCCATCCTTCTCAGCAACAATGATTAATACATGGCTTCATAAGAA
AAGAACCAAGTTCCTGCCATGGACAAGTGTTCTGCAACATTGTGCGGGATACAAAGGGGA

CACCGCACAAGTACGCACATCGATAGGCATCTAGGTAAAGGCCAGGAGCGACAGGGTGCA
AGCTGTGCAGCATGATGTGGAATAAAGGAGAGATGGTGTGCAGGGGATTTCAACCCAAGT
TTGAGCATCTCTAGCTCAAACTCTATATTTGAGGCCTCAGGCAAAAACAAAAGGTAGCTT
CAACAGGCCCCGATGAAGCCATCAAACTGGAGAGGGACCAACAAGCAATTGCCCTCAGCC
CCATTTCCCACAGGTCAAGTAGGGGTAGAGCGATAGCATGTAAGGAAGCATGGTAACAGT
GTAGGGTCATGACTCAGGAGTAGGGGTGGTAATGGATCGTGATCCAAATAGTTCTTCACA
AAATGTCAAAGTCCTAAATAAATTATAGTTCAAAAATGACTATAAATAGAGCCCGATCCT
AACCCGCTTCGATCCTTAAATCTTATAGTGCAAAATTTATAGCCCATTGTCAGCCCTACT
CAGGAGCAATAGGGATTGCAGCCCTGATGGACTTCATTTCTTAGAATCACCCCCCAAAAC
TACTATAGTTGCTCTAATGAAGCAATATCCAGGTAAATATATGAAATTCTCTAGTTTGTA
GTGGTTTTTTCCATTTTCCTATAAAAATCACGAGAACAAGAGAAAGTAGGCGACAGTATG
CCCCCCACCCCACCCACACACAGTGGGAAGCTAGCACCCTCTTTCGCTAAAAAAATCATG
TTGCAATGTTTACAAATGATTTATATAGTACCTAAAGAAAGTTAAGTGTAGCTGAAACAA
TTCAGTTTGAAATTCATGTTTGATGTTCAGATTCAAAAAAAAGTGTCACTCTCTGCACAT
GAGGAACAGTGCCAGGTTGGCTGCTTAATTATTTAGCCATGTTAGGTTAGCTTCTTTACA
GTGCTGCTGCTAAATTCTATTGTTCCTACAGTGTTTTTGTGTGTTCGGTTGGTAGCAGCT
GCAGACTAAACTATTGGCTTTAATCGCAAACAAATAGTTCGTTTCTTGACATCTAGGATG
AATATCAAACTAAATTTTTGCCACTATACTTTGTGTGTACTACATACATGATTTATGTGA
TGATCTTTTGAAATATTGCAACATGATATTTTAGTAAATGAGGCACCAGTCCCACTGGAC
ACCAAATATGAATTGTAGTTTGCACCCAGTACTGTGGTGCCCTCAACTTCAGGTTGTAGG
TGGATACCATGCTCATGCAGTAGACAGTACACTTGCAACATGAACATCCAGTCCAGCTCC
CTACAAAGCAATATGTCAGAAGAAATCAAAGGAGACGTTGATCATCAAATAAAATATAAT
GGATTTGAGAAAAAACCTCATCATGTGTTGGAACTTGGAAACTCCCTATCTTTTCCAAGG
AATCTCCATCCTTGTCTAATAGTTCTGTGACCTGTTATAATATGCTAGCAAACACAATGA
GATTAGGAGTACTATCACAAAAAATGCTCGTTTGGTAAAGTTGAGGAATGACATTAGAGA
AAAGTAAAAAGGAAGATATCTAGTTGCCATCAAAAGCGAATTATTTTAAACAAAGACATA
ATTAAGTAATACCATAGTAAAATGTAGTGATCGGTCATATCTGAAACTAACAATGACCAC
CAGAAAAGACAAACTCTATATAAATGCTAACTGGGTTTAGTACTTTAGCTATGAGTCCAT
TCTGACACTCATTTTCACTAGGTTTGCTCCGTACATTATTCTGCTTTGGTTGACAAGTTT
TAGAAGCTATGCATAATTTCTAGTATGTTTAATAGGTTCCCAAATATGCAAATAAATGCA
TAAATCTTCTTTAAACGTTGTGCACCAAGTTGCAGTTTTGGATATCTTGATAACTACCCA
TACGTGCAAACTGGATGGATGGCTAAACTAACATTTTTTATAGCCAAGTATGGTATGCAG
TGGTAGAGATAGGCACACATGTATTTTGTTTTGGAACTATTAATGCTTTTCTAGTTGTGC
TGTATAAATTCTAATGGGAAAATAGAAACAAGATTTGCAATTTCACGGTGTATTAGTGCT
ATTGTAGTCTTTAGAACATGAACAAGCTCACCAGTTCATGGCACAACGATGCAGATCCAT
ACTAATTGATGGAATAAATCCGATCGTAAATCTAAGCACACATATACCTTGTAGGGACGG
TTCTTGATGATGATGAAGTCGTTCTTGCAGACGTTGCTGGCCTGCTACGGGTAGGTCTTG
CACATCGTGCATGGTGATGCCTCGCTCGTTCGGGCTCTAACGACGGTAGGGCGGAGGTCG
CGGTGGGGCGGAGTGGCCGTGCAACGAGGAAGCAGTCGTCCACGTCCGCGGCGGGCAAGG
AGCAGAGCACTAGGCGAGCGGCGCGGGACGCATAGGCCAGCGAGATGCAGAGGTCACGAG
GGCAGGTTGGTCGCGAGCGGGGAGGGACACCCACGACGCACGAAGGCGGCTACGCACGAG
GCTACAGGTTGGACGATCGGGAAGGGGAGGTTGCGATGGACGATGGCAGCCACAAGCGGG
GGATGCGATGGCTCGAGAGGGCGAGGATCGATCAAGGGATACACTGACGACATAGGCAGA
ACAGGGAGTCAGCTTACCCCGTGCACACAATTTACGGCGTGGGGGGCGCCATCCACGGCA

TAGGATCTCAAACTGCCAAATCGGTGGGGAACGGGGGCATCATTCCCAAATCAGGCCTCA
GCGACCCATGGATGGCGGCGACCTGTGCCGGGTGCATATGCATGGTGCACGCAGATGGCA
GCGTGGGGGCGCCAATTTCCAAATCAGTGGGGAACGGGGGCATCATTTCCAAATCAAGGA
ACGGGGATGGAGCAGCGTTGAGGCAGGGGAGTAGCGCCGATGGGGGAGCGTTACCGAGGC
GGGGGGAGTAGCGTCGAGGGGGAGCGGTGCCGAGGCGAAGCTAGGGTTGAGAACGCATGC
ACGGGAACTAGGTTGGGGTTATGAGCTGGAGGCCGAGAGACATGCCCTGGGAGGGGGGCA
TAGGAGGCAGAGAGGTAGACATGAGATGCGCGTGGGGAGCTGGTGGGGGGATGGCCTAGG
CAGAACCGTGCACCATGGTCTACGTTAGGTTCTTATATAGTAGTAGAGATATAATCACTC
TTCGTAACCTCTCTCTTTCTCACGTACTCTTGAGTCTCCACCATATATATAATCCGCTGG
TTTGTATTTTCTTTCTTCTTTGGATCATCACAAAACCAACCAATGTAATTATTATTAGAC
CGACGCCACACAATCTTGGAACTTATTCATTATGCTAGTGTATATCTGTAATCCCATTCC
ATAATGTTAATGTTGATTTGTGGTCACGTTTCAATGAGAGCTAGCATGCCCACTGTTGCA
AACAACTTCCATTGAAGTAAGAACATAACAAATCG
>ZmPG5
ATGGAAGCGCGGCCGCGGCTGCTCTTAGTCGTCGTCGCGATCTTCGTGTCGATATCTGCC
GTCGCGGCAGCCGTCATAAACGTCAAGAACTACGGGGCCCATGGCAATGGTGTCAATGAC
GACACCAAGGTTCTTTCTTGGTCTTGGATCTGATTCGCCCTCCTGCACTCTGATGATTCA
TCAATATATATATATAATACTGCCCGCAGCCACTGATGGCGGCGTGGAAGGCAGCGTGCG
GATCAGCCGGCGCGGCGACGATGGTGGTGCCGCCGGGGACGTACTACATCGGCCCAGTGC
AGTTCCACGGCCCCTGCAAGGCCTCCACCTTGACCTTCCAGCTGCAGGCAAGTCATTCCC
ATTAGTAGTGGCCGGACGTGTGGGCCTCCCTCCACCTTGACCATGATGATGGATCGATCG
ATCGTCGTCCGTGCAGGGGACGCTCAAGGCCGCCACGGACCTGAAACGGTTCGGCAACGA
CTGGATTGAGTTCGGGTGGGTGAACGGGCTCACCGTCGCCGGTGGCGTCATTGACGGCCA
GGGCGCCGCCTCGTGGCCTTTCAACAAGTGTCCCATTCGCAAGGACTGCAAAGTGCTGCC
AACAGTACGTTCGCTCTCTTCTCCAATCATACATTAATTAATGATCTAGTGATCTAACAT
CATCTTGTGTTGCATGCACATGCAGAGCGTGTTGTTCGTGAACAACCAGAACACGGTGGT
GCGCGACGTCACGTCCGTGAACCCCAAGTTCTTCCACATGGCGCTGCTGTCGGTGAAGAA
CATCCGGATGAGCGGGCTCAAGATCAGCGCGCCCTCCAACAGCCCCAACACGGATGGCAT
CCACATCGAGCGCAGCAGCGGGATATCCATCATGGACACGCACATCAGCACGGGCGACGA
CTGCATCTCCATCGGCCAGGGCAACGACAACGTGGACGTGGCCCGCGTGCAGTGCGGCCC
AGGCCACGGCATGAGCGTCGGCAGCTTGGGTCGCTACTCCGGCGAGGGAGACGTCACACG
GGTGCACGTCCGCGACATGACCTTCACGGGCACCATGAACGGCGTTCGCATCAAGACATG
GGAGAACTCCCCTTCCAAGAGCAACGCCGCGCACATGGTCTTCGAGAACATGGTCATGAG
GGACGTGCAGAACCCCATTATCATCGATCAGAAGTACTGCCCCTACTACAACTGCGAGCA
CAAGGTATGTCGTCGTGCAGTGCTTCATCATTGTAAAATGTACTCTTCTCTTCATTCATG
CATGTATACACGTACGTACAGTACGTGTCCGGGGTGACTCTCAATGACATTCACTTCAAG
AACATCAAGGGCACGGCGACGACGCCGGTGGCGGTGTTGCTCCGGTGCGGTGTGCCGTGC
AGGGGTCTGGTGCTGCAGGACGTGGACCTTAGGTACAAGGGGCAGGGTGGTACATCGGCC
AAGTGCGAGAACGCCAAGGCCAAGTACCTTGGCTACCAGTTTCCCAAACCCTGCCCCTAG
GTGCCGGTGCTTGTCCGGCCGTATTTTAGCTCATCTCTGTCCACTCTCTGGTGTCCGGTG
AAGTGGGCTTTGATTCTTCGGTCGTGTTCTCTAGACGTTTTTTCTTCTTCTCTTCTGGGT
TTTCCTGTTTGCTTTCTTGATTAGCTGAATTTCGCGGTATAATGTCAGTTGCTTGTATCA
GTGAATTTGTGAATACCAGTCATTGTTGAATATATATATGGGGAATAATGCCTTGATAAA
TCCTTCCTGACTCATGATCTTCTAAAAAAAT

>ZmPG18
GGCGGCCGACCCATGTGCTTATGCCCCGTTACGACGGCAGCAGCATCGTGCGCGCGTGAC
TGATCCGCCGAAGCAGACACCGCGTACCATGGAAGCGCGGCCGCGGCTGCTCCTAGTCGC
CGCCGTGGCGGTCTTCGTGTCAGTGTCCGCCGCCGCCGGGGCGGCCGTCGTCATAAACGT
GAAGAACTACGGGGCCCATGGGAATGGCGTCAACGATGATACCAAGGTTGGGCCGGGCCG
GGGCTTATTCATTTCTTCCTTCGTCCTCGATGTGTGTCTGCCTGCCCTCTGATTCACCCT
CCGGCCGGCCGGCCGGCACTCTGATTCATGATGCTGCAGCCGCTGATGGCAGCGTGGAAG
GCAGCGTGCGGATCAGCTGGCGCGGTGACGATGGTCGTCGCGCCGGGGACGTACTACATC
GGGCCGGTGCAGTTCCACGGCCCCTGCAAGGCGTCCACCTTGACCTTCCAGCTGCAGGCA
CGTCGTCATCAGTTGCGCATTCATTCCCTTGGTAGTTTGCCGATCACACATTCACACGGA
CGTGTGTGTGGGCATGCGGTTACGTACAGTCTTGCATCCATGATTTGATGGTGGTGGTGG
TGGTGCAGGGGACGCTCAAGGCCGCCACGGACCTGAAGCGGTTCGGCAACGACTGGATCG
AGTTCGGGTGGGTGAACGGGCTGACCGTCGCCGGCGGCGTCATCGACGGCCAGGGCGCCG
CCTCGTGGCCTTTCAACAAGTGCCCCATCCGCAAGGACTGCAAAGTGCTGCCCACGGTCC
GTAAGGACGGAGAGCATCGAGCACTCAATGAACTAACCGGTGATCGACGATCGATCACTC
TAACATGTGCATCTGCATTACCACGTCGTACATATATATATGCAGAGCGTGCTGTTCGTG
AACAGCCAGAACACGGTGGTGCGCGACGTGACGTCGGTGAACCCCAAGTTCTTCCACATG
GCGCTGCTGTCGGTGAAGAACGTCCGGATGAGCGGGCTGAGGATCCGAGCGCCGCCCAAC
AGCCCCAACACGGACGGCATCCACATCGAGCGCAGCAGCGGGGTGTCCATCGTGGACACG
CACATCGGCACGGGCGACGACTGCATCTCCGTCGGCCAGGGAAACGACAACGTGGAGGTC
TCCCGCGTGCAGTGCGGCCCGGGCCACGGCATGAGCGTCGGCAGCCTGGGCCGATACTCC
GGCGAGGGCGACGTCACGCGGGTGCACGTCCGCGACATGACCTTCACGGGCACCACCAAC
GGCGTCCGCATCAAGACCTGGGAGAACTCGCCGTCCAGGAGCAACGCCGCGCACATGGTC
TTCGAGAACATGGTCATGAAGGACGTCCAGAACCCTATCATCATCGACCAGAAGTACTGC
CCCTACTACAACTGCGAGCACAAGGTACGGTATCGTCTTCGTCTCGATCGATCGTCGTTG
CCATTGATGTCGACATCATAACAACATATATATGTGATTAATGCTCTTTCATTTCATTTA
CATGCAGTACGTGTCCGGGGTCACTCTCAAGGACATTCACTTCAGGAACATCAAGGGCAC
GGCGACTACGCCGGTGGCGGTGTTGCTTCGCTGCGGCGTGCCGTGCCAGGGTCTGGTGCT
GCAGGACGTGGACCTTAGGTACAAGGGGCAGGGTGCCACATCGGCCAAGTGCGAGAACGC
CAAGGCCAAGTACGTTGGCTACCAGTTCCCCAAGCCCTGCAGCCCCTAGCTTAGATAGGT
GTCGTCGTGTGGCGCGAGGAGATCCTCTCCGTGGCCGGCCGGCCGGCCCCGCTTACCACT
CTGGTGTACCTGGCTCTAGCTAGCTAGCTCCAGTGTGCGGTGAAGTGGGCTTTGATTCTT
CCATCGTGTTGTCTAGACGTACGTTTTTCTTTTGGCTTTTCATGTTTGCTTTCTTGATTA
GCTGATTTTTGTGTTATGTGTAATCGCAATTTCTGTCATCTGCTAGTTGCCTGCATCAGT
GAATTGTGACTTCAGTTGGTAAAACCTAACTCATGGTTCAGTTGTTAACTTGCTTGTGGC
TT
>ZmPG34
GTAAAACCATGCATGCTCGGAGCAATCCGTGATGCCAACTGCATGTTAGGCATGTAGCTT
AATTTTATTCGATCTGTTCTCTTCCAGATCGAGCTGAGGTAAGGTACCGACGCAGCCATG
CCACTGCGTTTAGCCGCTCTGCTTCTCCTGGCTACTACGGCGACGTGGACCGCAGGGGTC
ACCGCCGCTGCCGCCGGCCGCGTCTTCAATGTGTCGGACTTCGGCGCGGTCGCGGACGGG
CGCACGGACGACTCCGAGGCCTTCCTGAGGGCGTGGACGGAGGCGTGCGCGACGCCGGGG
AGGCCGGCGGTCGTGGTCCCCAGGGGCGACTACCTGCTCCACCCGCTGGTGTTCCGGGGC
CCGTGCAGGGGCTACGTGGAGGTCCACGTCGGTGGCGTCCTCCGCGCGCCGCCGGGGCTC

GCCGCCTTCCGTGGCTGCCGCGAGTGGGTCCACTTCTCCAGCATCGACGGGCTGCTGGTC
ACCGGCGGCGGCACGTTCGATGGCCGCGGCGCCACCGCGTGGCCGCTCAATGAGTGCCCG
CAGAAGCGCGACTGCAGGCTTCTCCCAACCGTAAGTTCTGCGACTTGTGCGACGATTTTG
TTGCATTCTGATATAATTGTGGTTCAACGTCGTCATCAGTCCATCAAGCTCGGGCTGGTG
AGGAACGCGACCATCACGGGCGTGACGTCGCTGGACAGCAAGTTCTTCCACGTGGCCGTG
GTGGGCAGCCAGGACGTGCGCATCCACGGCGTCAGCATCCGCGCGCCGCGCAGCAGCCCG
AACACGGACGGCGTGCACATCCAGGGCTCGTCGAACGTGCGCGTGACGGACTCGGCCGTG
GCCACGGGCGACGATTGCGTGTCCGTGGGGCCGGGCAGCTCGGACGTGCTGGTGTCCGGA
GTGGCGTGCGGCCCGGGCCACGGCATCAGCGTGGGCAGCCTGGGCCGGTACCCCGGCGAG
GGGGACGTGCGGCGGCTGCGCGTGGCCAACTGCACCGTCGCCGGCACGTCCAACGGCGTG
CGCATCAAGACGTGGCGCGGCGGGTCGTGGCCGCCCACGGCCGTGGCCGGGCTCGTCTTC
GAGGACATCGTCATGAGGAAGGTCCGCAACCCCATCATCATCGACCAGGAGTACTGCCCC
TACCCGTCCTGCCGCGAGTCGGTACGTACGCTACGCTCCAGAAGCAAACTAATTAACTAA
TCGTCGCTAGCCCGTCGGCGCGCGCGCGCGCGCGCACATATATATATATATATATATA
TATATATATATATATATATATATATATATATATATATATATATATATATATATATATA
TATATCTGTCACGTTCACAATCGTGCAGGAGCAGCGGCCGTCGGCGGTGAGGATAAGCGA
CGTCAAGTTCAGGAACATCCGGGGCGAGTCGGCGACCAAGGTGGCCGTGAAGCTGTCGTG
CAGCGAGGCCAGCCCGTGCCGGGAGCTGGAGCTTAGGGACATCGACCTGCGCTACGTCAA
GCGCGGGGTCGCCACGCAGTCGCGCTGCGCGCACGTCGCCGGCGGTGTGGTCGGCGGCAC
GCTGGTCCCTCCCTCTTGCATATGAACGCCGGCCGCTCGCTCGCGCTACGTCAAGAGATG
GCTTGCTGCTGACTAGCTATGTGGACTGAAATACGGGTTAGCTAGTGAAAGAACGATGTA
CGGGCACAACAGTGCCGTATTGTAGGTTGTAGTCGAGTTTTTTTTTTCTTGAATTTTGTG
GCTAGTAGTGGTTCTCACCTCCGCTCCAACGCGACCTGGATTTGCTGACGACTCAGCTGT
TGACTGCATCTGGATCTCGTCACCCTCCAGGATGATAGGATATATGCATGTGCTCAATGT
AGAAATGGAGCTCAAATCCTGAAATTTGCGAAGCAGTAGAACTTCTTCAGTC
>ZmPG9
GTCGAACACATACCTAATTATCACCTGTAAGCCAGCTGAACCACATCTCGTCGTGCACAT
CCGGAGCCCGGGAACACACACACACATTGTCATGGCCATGCCCAAGCCCGTCCTGAGCCT
GCTCGTGCACCTACACGCCGCCGTCCTGTTCCTTCCCGCCCCGGCCGGCGGCGCGGTCTA
CAACGTGCTGCGCTACGGCGCGCGCCCCGACGGCGTGACGGACGCGGCGGGCCCTTCCT
CCGCGCGTGGGCGGACGCCTGCCGCTCGCCCCTCCCGGCCGCCGTGTACGTGCCGCCAGG
GAGGTACCTGGTGCGGAGCGCCACGTTCACCGGCCCGTGCCACACCCGCGCCGTGACGTT
CGCCGTCGCCGGCACGGTCGTCGCCCCCGCGGCCTACGGCGCGCGCGGCTCGTCGGGGAG
GTGGATCACGTTCGAGAACATGGACGGCCTCGTCGTCGCCGGCGGCGGCACGCTGGACGG
CCGCGGCAGGGCACTCTGGGCCTGCAGACAGCGCGGGCAGCGCGACTGTCCGACCCCCAC
GTCCGTACGTCGCCGCCGTCGAAACCTGACGATAACGCAAACAAGCTGATAATGCATGCG
CCTAACTTGGCTCGCCGCACTTTCTTGCTTGGTTGAGCGCGTGCAGACTCTGACGATCGC
CAACTCCAAGGACGTGGTGGTGGCCGGGCTCCGGTCGGTGGACAGCGAGCTGTTCCACGT
GGTGGTGCTGCAGTGCCACGGCGTGACGGTGCGCGGGGTGACGGTGGAGGCGCCGGCGGA
CAGCCCCAACACCGACGGCATCCACCTGCACATGTCCACCCACGTGTCGGTGTACGACGC
CAGGATCAGCACCGGCGACGACTGCATCTCCATCGGCCCCGGCAACTCTCACCTCTGGAT
CGAGCGCGTCGCCTGCGGCCCCGGCCACGGCATCAGGTACGTGCGCCGTCGTCTCGGTGG
TGGATCAGTGCGTGATGCGCGGTGATTTTGTCTGCATGAATGAGGGCGGAAATGCCCCCA
CGTTAGGAATTAAATCGAGGAAAATCAAAGCGTAGGAATGGAAGTTCGAGCTGGTTTTAT

GGAAATCAATCCATAAACTGGTTTGACTGAAACAGCCTCGAAAGTCGAAAGTTGAAAGAT
CTTCAAGTCAGATCAGACGAGTGGAAACTAGAGATGGCCAAACGGGCCGGCCCGGCCCGG
CCCGGCCCGGGCCCGGTGAAGCCCGGCCAAAACCGGGCCGGGCCTGCTGAGCCAGCGGGC
TTAAGTTTCTGTCCAAGCCCGGCCCGCAGCGGGCCTAAACGGGCCGGGCCGGCCCGTTTA
GCACGAAAAACGGGCCAAAAAGCGGGCTAAACGGGCCGGTAAGCACGTTTTAGTGTAAAA
AAAACGGGCTTAACGGGCTTAGAGGTAAACGGGCCGTGCCGGGCTAGCCCGCCGTGCCTA
GTTTCCTGTCCAAGCCCGCCCGCTTATTCTACCGTGCCGGGCTCGGACCGGGCCCAAAAA
GCGGGCTTCGTGCCGGGCTCACGGGCCTCGTGCTTTTTGGCCATCTATAGTGGAAACGGA
CCTGACCTGGGCCCGCTTTCCATACTCTAGTTCGCATAGTATCCACTAACGAAATCGATC
TTTCAACTAGCATCGGGAGCCTGGGCAAGCAGCAGGGCACGGAGGTGGAGGCGGTGCAGA
ACGTGACGGTGAAGACGACGTGGTTCACCGGCACCATGAACGGGCTGCGGATCAAGACGT
GGGGCGGCTCCAAGCGCGGCTTCGTCACGGGCGTCACCTTCGCCGACTCCACCATGTCCG
GCGTGGACAACCCCATCATCATCGACCAGAACTACTGCCCCACCAGCAGCGGCTGCCCCG
GCGCCGGCCGGAGCTCCAGCATCAGGATCAGCGACGTGCGGTACGTGGGCATCCGGGGCT
CGTCGGCCACGCCGGTGGCCGTCAACTTCGACTGCAGCCGGAGCTACCCCTGCAGTGGCA
TCAGCCTGCAGGACGTGGCGCTGACGTACCAGAACCGGGCCGCCGCCAAGTCCTACTGCC
GGAACGTGCAGGGGACCGCGCTCGGCCTCGTGCTGCCGCCGAGCTGCCTCTGATCAGACG
ATCGACCTTGCAACTTACATTGCCGGCCGGACGCAGCAGTTAGCTGCATGCGTTGTCACG
TGTATATAGATGCATATGTCGAGTGTATTATGTACGGAGTATGTGGGGAGTGCTTACTGC
TTAGACTAGGAGAACATTACGAGCTTCTTCTGACAGTAGATGGTTATATAGTCGATATCT
GTTGGTTCTTTTCTTTAACCCTTCGCCTTTTTCGCAGCACGATTATTGTATGAGCAAGTT
TTTTAAATAA
>ZmPG14
ATGGCTCCCTTCTTCGACGCTTATTATTCTTCCCTAGTGCCGCTACTCTTTTCCTGCGTG
CTGTCGTCAGGTGCAGGTGCAGCTGAAGCGGCGGCGGCGGCGGCGTACAAGTACAACGTC
GCCGGTTTCGGAGCGAGGCCCGACGGCCGGACGGACTCGGCCGGCGCGTTCGCCAGTGCC
TGGCCCGCCGCCTGCCGGTCGCAGGAGCCGGCGACCGTGTACGTGCCGAGCGGGCGCTTC
CTGCTCAGCCGTGCGGCCTTCACCGGGCCGTGCTCGAGCAGGGTGATGACGCTTCGGGTC
GATGACACGCTTGTTGCGCCGTCAGGCTACACCAGCCGTGGCGGCAGCAGCAGCCAGCAG
GGACGACGGCGCCTGGATCGTGTTCGACCGCGTCGACGGCCTCACTGTGTGGGGCGGCAC
CGTCGACGGCCGCGGCGAGGCGCTGTGGCCTGTGGGCGTGCAAGCAGGCTGCTGAGCACG
GCGGCTGCCCCAGCGGAGCAACAGTGAGTTGTTGGAGTTGCCATAGCACGTCACGTGGCA
CTGCACCGCAAACTGTAGCACCATATGTTGTTGTTTACATGACACCTCTTGACTTGGCCG
TGCGTCGGCAGTCGCTGAAGGTGCTGAACTCGAGGGACGTGGTGATCAGCGGCCTGACCT
CGGTCGAGAGCGAGCTGTACCACGTCGTGGTCGAGGGCTGCGAAGGCGTCACGGTGCAGG
ACGTGCAGATCGTCGCGCCGGGGAGCAGCCCCAACACGGACGGGATCCACGTGCAGGCCT
CGTCCCAGGTCACGGTCACCCGGACCAGCATCCGGACCGGGGACGACGACTGCGTCTCCG
TTGGCACCGGCACCACCAACCTGCGCGTGGAGCACGTCAGCTGCGGCCCGGGCCATGGCA
TAAGGTGCCTACTAGCTAGTGGGCTGATGATGAGCACACTCTTCGCGCACGCACTGACTG
CACGCCCCCTCTTGTCGTCGTCGCGACTCACTTGTGCTGTGCCGCTGTGCGTGATGCTTC
GGGTCGGGTGGCGTCCAGCATAGGGATGGAGCCTGGGGAAGGAGAGAGAGGAGAGTGGCG
TGGAGAACGTGACGGTGACGGGGGCCGCCTTCGTCGCCACCGACAACGGGCTGCGCCTGC
GGATCAAGACGTGGGCGCGGGCCAGGGTGGACGGCGCGTACGTGCGCGGCGTCGTCTTCG
AGCACGCGCTCATGCACGACGTGCGCAACCCGATCATCATCGACTAG

>ZmPG3
CTTACTCACCATCACCAGGTCAGCATCCAATCCAAGCATCCAGCAACCAGCACCAACCTG
AGCAGCTTGCAACAAGAGCTGCAGTATATCTATAAATTCTGTTCTGATCTTGTTCGCTAG
CTTGACTGTTCGAGCCCGGGTTGCAAATAACAACCTGCACTGCAGCGTGATTAGCTCTGC
CAATGGCATCGTCCAGCGGGATGAAGCTTGTGTACATGGCGCCTCTCCTCCCGTTGCTCT
TCATGTCCGGCGTTCTTGAGGCTGCAGGAGCGGGCAGCTCGGCTGACGGCACGAACGGCA
ACTTGCACGGGTCTGTCACCAGCAGACCTGCCGCCGCGCCCAGGGGAAGATCGCTGCCGG
AATCGCAGAGCGTGTTCAGCCTCGACCGCTACGGTGCCCGTGGCGACGGGAGGAACGATG
ACACGCGGGCGCTGGAGATGGCGTGGAAGGCGGCGTGCGCCTCGCCACGGCCGGCCGTCG
TGCTCGTCCCTGCCGGCAGGCGCTACCTGCTGAAGCTCCTCACCCTCCGTGGCCCGTGCA
AGTCCAGTGTCACGCTCACGGTGAAGGGCACCCTGGTTGCATCGCCAAACCGGGCGGATT
GGAGCGACAGTGACAGGAGGCACTGGATCGTGTTCCGAAGCATCGACGAGCTCACTGTCA
ACGGCGGTGGCGCCATCGATGGCAACGGCGAGAAATGGTGGCCTCACTCGTGCAAGATCA
ACAAGGCTCTCGTAAGTGTGGCGCTGGCCCGTTGGTCTATCTAAATATCTATAAGCATCT
ACTTCACAGGCAAGTGTAATTGAATTGGCCTTTTCTTTTTTTTTCTTTTCTTTAGCCTTG
CAAGGAGGCTCCGACGGCTCTGTCATTCCACTACTGCGTCGGCCTGAAAGTGGAGGATCT
GAAAATTATGAACAGCCAACAGATCCACATGTCAGTCGAGGATTCTGCAAATGTGCTGCT
GGCGCGGTTGTCCATCACAGCGCCTGGCACCAGCCCTAACACTGACGGCATCCACATCAC
TCGCAGCAAAGATGTACGCGTCACAGACTGCAAGATCAAGACAGGTAAATAAATAGAATG
TTCAGGATTTGGATCATATACAACATATGCCATTGTTGTGTCTTCAAGCTTCACTGAACT
TAGTTCCCTTTTTTTTACTGAATTTTGCAGGGGACGACTGCATGTCAATCGAGAACGGGA
CTCACAACCTCCATGTCTCCAAAGTTACCTGTGGTCCAGGGCATGGGATCAGCATCGGAA
GCCTAGGAGACGACAACTCAAGAGCAGAAGTCTCCGGCATCACCATAGACTCAGTGCAAC
TACACGGCACAACCAACGGAGCACGCATCAAGACGTACCAGGGAGGCAGCGGATACGCCA
AGGACATCACGTTCCAAAACATGGTTATGTACGACGTCAAGAACCCGATAATCATCGACC
AGAACTACTGCGACAGGGCTAAGCCATGCGGAGAACAAGAGTCAGCGGTGCAGGTCAGCG
GCGTCGTCTTCAAGAACATTAGAGGGACGACCAGTACCAAGGACGCCATCAAGATGAACT
GCAGCGAGAACGTCCCATGCCAGGGCATTACCTTGCAGAACATCGACCTGGAAATGCAGG
ACGGCAAGGGCAGCACGAGAAGCACATGTAGGAATGCGAAATGGAGAGAATTTGGGACCG
TTCACCCGCAGCCTTGCACTGCCACAGACACAGAGTAGGGGGCAATGGAACTCAGTCGCT
TGTGCTTTGTAGCTAGTCTGTACAGAACTACAGATGGAGTAAAGGCATACTCATGTATAT
TTGTTAATTGGTTCATTCATTCATGTATATAGACTGTATAGTATTTTGTAGAACGAAATA
AAGAAG
>ZmPG4
CTGCCTCTCCCTCCTCCGCGCCCGGCACCGCCATGACCAAGCAGCAGCTAGGTCTGCGTG
CGTGTGCAAATGGAGAGAAGGGATGCGTTGGAGAAGAAGATGGAAAGAGCATGGTCGTGC
CGCCCAGTGCTTGCCGGGGCGCGCTAGCGACTTGTGCCCATGGCCTGGCCGGCGGCCGGT
GTCCTCTGTTGCCAAGCGTTCTCTGTTGCCGGAGAAGAAATGTGCGTGCGTTCTGAAGGA
AGAAGAAGACCCCTTTGGGTCTATGACGTGTGGGCCCAAGGCAGGGTAAGGGTGTTTCAA
ATTCCAGATTTTTGCTATTTCTTCACAAATTCATATCTCGAGTTTTAGAGCTCCTAAAAT
CGTAAAACCTGTTTTATTGGCTTCCTTATGAAATGCTCTAACTCTTGGATCTATATGTTG
GTATGTTTTAGTGGAAAAGTTTTTCTGTAGAAATTCATATTTAAAAACTGGTTTAGAAAG
AAAATGAACTTGCTTCTCTCCATAATTTTATTTATAAAAATCTAAAAATGATGAAACCTG
TTTTGTTAGTTAGATATTGTTATACTCCATCAAGGAAAAATATAAAACTTATATGTTGTT

CACTGTTTTCTTGGTGTGTTAATTAAAACATGTTAAATAGGAAAAAGATGATTGTTGTGA
TTGTTATTTTTGGATCTGTAGCTCTGGTGCTCCAAATAGGTTGAAAGTTTTACAGTAGAC
TCTTGGTGTGATGTTTGGTGACTGGTAAAAATTTCATGAATTGTTGTGCATGTATGATTA
AGTTAGTGATTTAACTTGTTTAATGCACCTTAAGTAGTTTTAAATGATTTAAAAATAATG
TATAGATGCAAAAATGTAAAATGGTGTTCTACTGTCCTTGCATGGTATTATAGTAGCCTA
AGAAAACTCAATATGGTAATGCATCTACAGAAAATAATTAGTTTTAGATTTAACTTGCTC
TCACATGCTTTATTGCATTGACAATTTATTATTTTTGTATTAAATAAAATATAAAACCTG
GGCATGTGAAATTTATACATTAGTCATAAATTGTTATTACCTTTGCCTCATAAAAATTCC
AGCCCCAAATTAGGTACTTTCGTATGGTGCTAAAATAACACCCATTTTATAATTATAAAG
AAGAGTTAGTGAAAATTAGAAGTAAACAAAAGCTTGCATGTCTCTTGGAATGTTGAAATA
TACCTTAGTACCAATTAGAGATTAAATGGTTATGCGCATGATGAGTTTAGTAAAAATTCA
TCGGTGTCACTGGTGTTAAGTTGAGGTTTATTTGCGCGTGTAAAGTAATAATGTGATTAA
TACTTAGAACTCTTAGTCGATAGTAGAAATTGTTTTGGCTTATATAGAGAGGTGGTGACA
TGTCAAAGTAGTCATCGCTTCCTCTATATCTTGAGTAGGGTCAATACGCGTTATGTGTTC
ATGAATTGGGAAGTAAGCACATGATGGTTTCGAGTTAGAGAATAGTGGTTTACGGGTCAT
ATGATTTAGTGGATGTGTGAAAATACATGTGAATATGTACGTTTATATAAATGTTGTGGC
ATATGTTGTGTCTCAGATTTCGATAATAATAGGCTAGCCGACGAGTATGTTGTATAGCGG
CCTAGGTTGTTGCTCAGGTTAATCAACTTATTAGATGACTTTAAGTTCATGGTCACGATG
AATTGCGTGTTGTAGCCCAATTGCGCATGGTTATGACCGCGGGTAAAAAAACAGTAGTGT
TCATATGATGTCTAAGCTAAAATGCAAATTAGTGTGTGAAGGGCGCATCGATACATGTAT
ATGTACCGGGTCCGATTATTGTGGTGGATGTGTTTGTTAATTGTGTGTGGTGATTCTAGC
GTACAAAATGATACGGATAGAAATTTAGTAAGTCTACTTGTTAGTTCTCCTTTGATTATG
TTAACTCTAACAAATGATAAAGTGTTAGAATAATTCAAGTCTCTAGATCAAGGAATTTAT
TGATTGCTGTTTTGGACTGCATGCACTGTTTTAGTTGTGCTGTATATTTGATGAACCAAA
TTGTGTTTTCTGTAGATATGTGCTATAGAAAAGTGGTAAATAACTTTATTATCTTGCTGG
TGTTAAAATTTGACAGCCATAGGTCTGACAGTTAAGGAGTTATGCTTTTTACAAATTCAG
TAACTGAATCTGTCCAATTTCTGTACAGATTTCAGAAACTGCATTGTTTGCTCAATTTAA
TGTTGCAATCTGTTTATGATCGTTATAAGAAAGTTGTAGATGCTTTTCCGATCTTGGTTG
TGTTAAAATTTCATAACTAAAGGCCTGACGGTTTAGGAGTTATGAAATTTGCAAACTAGT
TGTTGTGTTCTGTCCACCGTCAGAACAGATTTCGAAAACTATGTTGTTTGATTTAATTAA
ACATTGAATCACTTCTTAGTGATTATAAAAGTTATGTAGTGCTTTTGCTGAGATTTCCAA
AAAGTCTTGGATCACTATTTTTGGTGGTCTAAAGAATAAGTTATGAACGTTTACAGTCTG
AAGACTGAATCTGTCCAATTTGGGACAGCAAGCCTTCTAGTGTCTTTTATCCTTAATTAA
ATATTGAATCACCTTGAGATGTTTATAAATAATTTGTAGAACATTTAATTAGCTTTCCAG
AAAGTCTAAGATCACTTTGTTTGGATGTCTGAATCTTCAGTTATGGATTTTTAAAGTCAC
AGGTCTGAATCTGTCCAAATCGGGACAGAGCTGCTGTGATTGCACTTTTGACCTTGCTAA
GTGTTTAATCGTGCGGTGATGACAATACCAAAGTTGTAGAGCACCTTCTAAGATTTCCAG
AAAATCTTAGTTTGCTAGTTTTGGATTAATATTTAAAGAGTTATGGTTAAAACAAGCCAC
TGCTGTGCTGCTGTCTTAATTTTGTTGTATTTTGTGGATTGCTCTACTCTGTGGCGTATG
TCACCGTTTCCTCGTTTTGATTTTGGTTGAACACGCAGTAACGATAATATAACTAATATA
AGTGAGCATAAGTATGTAGGTAACTAGTACACGCCTCGCTAAATTAGGGAACTAAATTGG
TTAAGGTCTAATACGTGTTCGCCTAATAAATAATTGTAAAGCCCAATTAAGTGTCTAGTT
CCTTTTCAACTAAATCTGTCTACATATCAGTCATAGGAAACTTAAGTACTTAACTAACCT
AATGTTAAGTAACCAATGAATGATTAAAGTGAATGTGATAAATGCTTGCACGTGTGATGT

CGTATTGCGCTGGTTAAGTCTAGAAGGTATCGGTCGTCTTTCTAATGGTAGTAACTACGT
ATGCCCGATGACGTGTAGATAACTAAATCTAACATGTGGTTGTTTGCGGTGTCTTACAAT
TAAATATTTAGTTCACTACCGTGTATTGTATATCTTGTGATAACTTTTCATTATATTCAT
ACATATGCATCTTGCACCTCATATAGGACCGAGAGATGATGATCGAGCCAGTGATGTGGT
GCCAACCACAAGATGCAGTTGGTGGACGACCTGAAGACTGATGGACTTACCCAGTTGATG
CTCGCCAAGCGAGTACCCCCCCAGCAAACACTACCTAAGTGTTAAATTAAAGGCAAGCCC
CGGTTTTATGCATAACCGTTATTGTATGTTATTTTACTACACTTAATGTTTGTAGGCTTG
TACTGTGCACTTAAGTGTAGGAGTTGACTGAAACCCTAGTTGCATGAACTCAGGTTCCCT
TTTGAGATGGATACTAGTATGCTAGGTCGAGTAGCTGCTTTACTAATTAGGGATCTTGGT
AGAAGTCGAGTGATTTTTCTAGCACTCGCGCGAGGTCATGAATTGGTTGTATCCACTTTG
ATAACAGAATGGTGATGGTCTGTGGACACGAGTCCATGGGGACGCGTGGTCTACGAGATG
AAAATTGGAATAAGGATTAACGTGCGGATACCTGTGTCAAGCGTTTGAACGTACTAAACA
CATACCGAGAAATATGGTAAATCGGTAAGCCTAGTACCTGAGTGAACCTGCCCGCAGACA
TATCCCCTCACGCGACCTGAGACGTGGTCTCCCATTCCGGTTATGGTGGGTACAAGTGCG
GTCACTGCACGACGGCAGTCGGGGTCAGTGAGGCATTGTACGCCAAGGCGGTGAGCCCTG
ATCTGTTGCCAGGGAATCGACGGGGACGGTTGATGTGTGTGGGGACGGAGTGCCTTGCCA
CGTCGTGTGTTTAGGTTTACCTTGCAAGGATAAAAACTCGATTCGAATCGTCTGCTTCTC
GCAGCTAATGAGACTGCTTGATCCATTGTACTGCATTGAGTAATAAGTGGAAATGAGGTG
ACTGGCAAAAGATGTTGATTGATAAAATGTTTGATACCATGTATGAATAGCTAGGTACAC
ATCTAGTATAAAGGATTCTACTAAAACTTGAAAAGCTAAAACTTGATTTTAGACTCAGCT
AGTGCTTTTGGCAAACCAAACCCCTCAGCCAAACAGCTGCATGTCTAGAGGTAGAGGAGT
AGACTCCTCACACCGGGTAAGTCTAGCTGAGTATTAGTATACTCAGCCTTGCTTGTGGCA
TAATTTTTGCAGGTTCCCTTAGGGTGATTGGTTGCTGGTGTGACTTGGCCTCCATCCCTG
CCACCGGGATAGACGGTCGAGTGGGTTATTGCTTCCGCAGGAGAGGACCAGGAGGAGTAG
TGTGGCCAGACTTCGCCATGTTACTCGGTTCTTCTCCGTTAGTTATTTCCGCTGCATTAA
AATTTATGGTTATTATTTTTGAAACTCTGATAATGTAATCACTAATGATACTTATTAAAT
TTGTGGTATTATGTTTTATTGTATTTCTCTGTGCCTCACCTTCGTGTGAGCTAGTGGTAT
TCGATCCTGGTAAGTGGCTTTATCGGACTAGATCCGAGGGACTGACAGATTATTCCTGTT
TAAGTGTGTTGCTGCCCTTAAGGGTGCGACTTGGGCACTTAAGCTGGAATAATTCGGGCG
GTTCCGCCACAGTTTTGCGCCTTATGCAATTGGGAACCCAACATACACCAATGTTGAGGG
ACCAAATATGCCTATCGAGGGATGTAATATGCATGTAGAGGGATCAAATATGGGTATTGA
GGGGAAAATGGTATGTGAGACACAACACCACTACCAAGACTGATCACGTTATGCCAGGTG
TTACTAATGATTTCTTTGGGCACAAAGACGTGGGTCAAGACGACGTCGAAGTTTAGGTTG
GTAAAAGAAAACAAGTGAACTTTTTTCGTAGCAAAATTGCATTATTATTCTGAGTCATCA
ACATTATTTATGAAAGTATGGAGGCTTTAGAGGAGACCGCAACATAACATTTTTACGAAG
AGTCTAAGGGTTGTACCCAAAAATCACAATATTGTGATCTGTTATTTAGTTGTTGATACT
TTAAGGTCAATTCAGTTAGTCTGATGTTAGCTTCAACGAGTGTTATTAGTTCTAGCAAAA
CTCCTTCTAGAGGAGAACAAAGTGTATGCTAACATGTAATGTACAAAGAAGCTAGCGAGT
TCGTTGATTATGGGTATCGAAAAGATCCACGTGTGTAGGAACTATTGTATCCTATATTGA
GGTGAGGATTATGAAGGCTTACAGAGATGTCTAAATTATAGTGCTAATAGATACAAGACG
AACAAAGATTATCTCGGGAAAGAGAATGGAGCCTCTGATGAAGACCTCCAGTTAGTAGTA
TTATAATGTTCTAAAACCAGTAGATTTTTTTGTAAAAATCCTAGAAACTTCGCCCAAGTG
TATTTGATGCCCAGGATAATGAACAAAGATCTTTTGCCTTTTTTCTTGTCACATTTTTAT
TAGAGTTAAATGCATTGTAGCCCATGTACTTGTCAAGAGATGTCATTTAGGTATACGAAC

TTTGGAAATACATTTATGGGTCCTTAAATTTATTAAGTGGCATACCGCAGTGGTCGGTAT
GATCCAAAGATGAACTTATCATTTACCATTTTTACAAGTTCTTAGCATGTTTAAGGGATC
CAGAAATGCATTTTCAAAGTTTGTGGATCTAAGTGACATACTTAACATTTAACTCCTTTT
ATTATATCAAAAAAGTTTAAATGCATCAATAGCAAGGAAATGCATGAACTATGTTTAGGA
TCATTTTACATACATGATGACTCTCTTGTACATGTGATTTTATTTTAGAATGTGAACATC
ATTACTCCGTTAGTATTACCTGTTCTCTAAGTATCATACAGTGACGTTCAGGTTGGCTAT
AGCAAATAAACATATACTCCCTCCGTTCTTTTTTATTTGTCTCATTTTAGTTCAAAAATA
AACTAGTTGGTGACAAATATTCGAAAACCGAGGTAATATGTGATTGGTTAGCTGTACCGT
CTCGTCCCGCGTCACCACGTGCGTTGACTTTATCTGAGTTTGTGTTTGGTTGCCTGCACG
TGTGAAGTGCAGGCTGTATAGACAGACATAAAATAAGTTATATTGGTTGCATCCACACGT
ACGCCTAAAACAGTCGTACGTGGTTGGCCAGGCTCACGCGAGATTATACGCGTGGGTACG
GTCATCCAATCAGGCGTATATGTACCAAGTACCGGATTGCATCATCATCGACACGTAGAT
ATATGTTGCTACGGGGCAATTATATCGGTTCCACGGATGCCTAACTGAATTTTGTTGTCA
CTGCCGTGGGGGCTCTCATGCTCCAATCTTCATGGAGTCCACTCCACAGAATCAGATGGA
CATGGATTTGCTTCCCATGCCAATTGCAACGAGAAAGGAAACATGTTTGAGAATTTTCGT
CATGAAAAACCTGCAGGTGGACTCGATTCAGCAGTTAATCAGTTCGGGCACATTTATCTC
CTTGCGATCATGGCTGAAGAATCTGAGGGCCGGATCCATCTATGTCTGCACGTATGGGTC
ATGGCGTGTCTGCAAGCATGCACACTCTACTGCACATATCTGCTGCCAACCAAACTCTCC
CGCGTTGTTCTTCTTCACAAGCAACGCATGCAACTCTCTTCAGCGCGCCTATATAAACGG
CCGTGGGCATGCAGCTTACTCACTCACCACCAGGTCACCATCCAATCCAGCAACCAGCAC
CAGCACCAACCTGAGCAAGCCTTGCAAAAAGAGCTGCACTATATCCATCGATTCTGTTCT
GATCTTGTTCGCTAGCTTGAGTGTTCAGCCCGGGCTGCAAGCCTGCAACTAACCACCTGC
ACTGCAGCGTGATTAGCTCTACAAATCGCATCCAGCGGGAAGCTGATGAAGCTTGTGTAC
ATGGTGACCCTCCTACCGTTGCTCTTCATGTCCGGCGTTCTTGAGGCTGCAGGAGCGGGC
AATGACAGCTCGGCTGACGGCACGAACGGCAACTTGCACAGCTCTGGCACCAGCAGACCC
GCCGCCGCGCCCAGGGGAAGGTCGCTGCAAGCATCGCAGAGCAGCGTGTACAGCGTCGAC
CGCTACGGTGCCCGTGGCGACGGGAGGAACGATGACACGCGGGCGCTGGAAATGGCGTGG
AAGGCGGCGTGCGCCTCGCCACGGCCGGCCGTCGTGCTCGTCCCTGACGGCAGGCGTTAC
CTGCTGAAGCTCCTCACCCTCCGCGGCCCGTGCAAGTCCAGCGTCACGCTCACGGTGAAG
GGCACTCTGGTTGCATCGCCAAACCGGGCGGATTGGAGCGACAATGACAGGAGGCACTGG
ATCGTGTTCCGGAGCATCGACAAGCTCACCGTCAACGGCGGTGGCGCCATCGATGGCAAC
GGCGAGAAATGGTGGCCTCACTCGTGCAAGATCAACAAGGCTCTCGTAAGTGTGGCGCTG
GCCCGTTGGTCGATCTAAATATCTATAAGCTTCTACTTCACAGATACAGGCAAATGTAAT
TGAATTGACCTTTTTTTTTGTTTTCTTTTCTTTAGCCTTGCAAGGAGGCTCCGACGGCTC
TGTCATTCCACTACTGCGTCGACCTGAAAGTGGAGGATCTGAAAATCGTGAACAGCCAAC
AGATACACATGTCAGTCGAGGATTCTGCAAATGCGCTGCTGGCGCGGTTGTCCATCACAG
CGCCTGGCACCAGCCCTAACACTGACGGCATCCACATCACTCGCAGCAAAGATGTACGCG
TCACAGACTGCAAGATCAAGACAGGTAAATAGCATGTTCAGGATTTGGATCATATACAAC
ATATGCCATTGTTGTGTCTTCAAGCTTCACTGAACTTAGTTCCCTTGTTTTACTGAATTT
TGCAGGGGACGACTGCATGTCAATCGAGAACGGGACTCACAACCTCCATGTCTCCAAAGT
TAACTGTGGTCCAGGGCATGGGATCAGCATCGGAAGCCTAGGAGACGACAACTCAAGAGC
AGAAGTCTCCGGCATCACCATAGATTCAGTGCAACTACACGGCACAACCAACGGAGCACG
CATCAAGACGTACCAGGGAGGCAGCGGATACGCCAAGGACATCACGTTCCAAAATATGGT
TATGTACGACGTCAAGAACCCGATAATCATCGACCAGAACTACTGCGACAAGGCTAAGCC

ATGCGGAGAACAAGAGTCAGCAGTGCAGGTCAGCGGTGTCGTCTTCAAGAACATTAGAGG
GACGACCAGTACCAAGGACGCCATCAAGATGAACTGCAGTGAGAACGTCCCATGCCAAGG
CATTACCTTGCAGAACATCGACCTGAAAATGCAGGACGGCAAGGGCAACACAAGAAGCAC
TTGTCAGAATGCAAAATGGACAGAATTTGGGACAGTTCGCCCGCAGCCTTGCACTGCCAT
AAAGTAGGGGGCAATTGAAGTCTGTTGCTCGTGCTTTGTAGTCTGTACAGATGGAGTAGT
CATACTCATGTATATATATACCGCCAACTCTAGTAACACCGTACATGCGGTCATGTATAA
TATTGTTTTGCACTGTAGATGCATTGTTTGTAGATATAA
>ZmPG2
CGAGCACGATCGCCTCCGACTTCAGTTCCGTGCTGACGACGCGATTCACTTCAGAGTTGA
GGCCTGTCCATCGGTGCTTGGCGCTGCCCATGGCGTCCAGCATGAAGCCGACAATGCTCG
CGTCCACGGCGGCCCTGCCCCTCCTCGCGCTCCTCCTCCTCCTCCACCTCTCGTCCG
GCGCTCTCGAGGCCGTAGTAGTAAGCGCGAGCAACGGCACCGCAGCCGACGGTGCGAGCT
ACACGTGCAGCGGGTCCCTGACGTGGCCGTCGTCGTCTCACCAGAGCGTGTTCAGCCTCG
ACCGCTACGGCGCCCGCGGCGACGGCAGGCACGACGACACGGGCGCGCTCGCCAGGGCGT
GGGAGGCGGCGTGCGCCTCGCCGCGGCCGGCCGTCCTGCTCGTCCCCGGCGGCAGGCGCT
ACCTGCTCAAGCTCGTCGTCCTGCGTGGCCCGTGCAGGTCCCGCGTCGCGGTCACCGTGA
AGGGCACCCTGGTGGCGTCGCCCAACAGGGCGGACTGGAGCGACAGCGACCGGAGGCACT
GGATCGTGTTCCGGAGCGTCGACAGGCTCACTGTCCACGGCCGTGGCTCCATCGACGGCA
ACGGACACAGCTGGTGGCCGCACTCGTGCAAGATCAACAAGGCTCTGGTAGTATAAAGCT
GTGGCGCCGACCGGTTCTAAAAACGAAGTGTGTGTGTCATGTGTGACCTCCTAACTGAAC
TGGCTTCTTCTCTCTTTCTCTATAGCCCTGCAAGGCGGCTCCAACGGCTCTGTCGTTCCA
CTACTGCACCAATCTAAGGGTGGAGAATCTCAAGATGATGAACAGCCAGCAGATCCACAT
GTCGATCGAGGACTGCGCCAGCGTGCAGGTGTCAAGGCTGTCCATCACAGCACCCGGCAC
GAGCCCTAACACCGACGGCATCCACATCACGCGCAGCAAAGATGTGCGCGTCACGAACTG
CAAGATCAAGACAGGTACGTAGTAGTAGTAGCAGCCAACACGTACGCCAAGCCATGGACA
CCACTACTATGCCTTCAATTCAAACTTTTCCCTGCTCAATTTCGCAGGAGACGACTGCAT
GTCGATCGAGGACGGGACGCACGGCCTCCACGTCTCCGGCGTCGTCTGTGGCCCCGGGCA
CGGGATCAGCGTCGGGAGCCTAGGAGACGACGACTCGCGAGCGGAGGTCTCCGGCATCAC
CATAGACTCAGTGCAGCTACACGGCACCACCAACGGGGCCCGGATCAAGACGTACCAGGG
AGGCAGCGGGTACGCCAGGGACATCACGTTCCAGAACATGGCCATGCACGGCGTCAGGAA
CCCGATCGTCATCGACCAGAGCTACTGCGACAGGGCCGAGGCTGAGCCGCCGTGCCGGGA
GCAGAGGTCGGCGGTGCAGATCAGCGACGTCGTCTTCAGGAACATCCGGGGGACGACCGT
CACCAGGGACGCCATCAGGATGAGCTGCAGCAGGGACGTCCCGTGCCGGGGCATCGTCCT
GCAGAACATTGACCTCAAAATGCAGGGCGGGAAGGGACACGCGGAGAGCACCTGCCAGAA
TGCCAAGTGGAGAAAGTCCGGAAAGGTCGTTCCGCAGCCGTGCACCTCGAAGGAGGAGGG
CAAGGTTGGGCAGCTGTTGGAGTTCCTCTTAGATCACGAGCTCGCGTCGTCTTTGCGCTC
GTGGAGTTCCTGGTCACTGTGGAACTAGAGACCTGCATGCAGTTCGTCGTTTTGCTGCTG
TGCTCAGTGAGTACGTCAGTGTATTTGTCATTTCCTTCACGTAAGTAGTACGTAACACCC
ATGCATGTATAATGTACTGTTATATATATTAATATATAGCAACCACAACCACGCATCCAC
ATCAG
>ZmPG23
CATTCTTCTTCACACACGCAACAGCCAGCGTGCACAACCCCTCTCGAACGATCCGTTCCC
CCTCTCGTCGCGCCTCCAGCTCCAAGAATCCAGGCTCCAGCAGCGAGTAGCAGCCCGTGA
GCTGAGGCTGATCCATGCTCCACATCCAATCACACACACACACACACACACATCAAGCCC

GGCAAGCAAAGCAATTCATTCGGGCTGGCATTTTTCATCAAGTCTCCTGTCGTCGTCGAC
GACCTTGCTACTAGCTCATATATAACCAGGCCGATACTAAGCTGCTAAGCGTATAGAGTT
AACCTGTCGCCGATCCTGCATGCATTCGTCCTTATTATTACTATTAGCTATGCCGACAAC
AATGGTGGTGGCGGCGGCGGCGGCCCGTACAGATAAGCCGCGGCTTATGTGGTTGCCGCC
TCTCGTCGTCGCGCTGCTCTTGCTGTCCGGCGCCCTACTAGAGGCTGCCGCCGCCGCTGC
GGGCACGAGCACCACCTCGGCCGATGACGCCGCGAGCTCGACCAGCTCCTTGGCCGCCGA
ACGAGCCGCCACGGGCCGCCGCAGCAGGTTGCTGGTGGCGTCGCAGAGCACGTTCTTCAG
CCTGGACAGCTACGGCGCCCGCGGCGACGGGAGGCACGACGACACCCAGGCGCTGGCCAG
GGCGTGGGAGGCGGCCTGCTTCTCGCCGCGGCCGGCCGTCCTGCTCGTCCCCGGCGGCAA
GCGCTACCTGCTGAAGCTCGTCGCGCTCTCCGGCCCGTGCAAGTCCAGCGTCACGGTGAC
GGTGGAGGGCACCCTGGTGGCGTCGCGGGACCGGTCCGACTGGATTGGCGGCGGCGGCGG
CACTGACCGGCGGCGCCACTGGATCGTGTTCCGTCAGGTCGACGGTCTCACCGTCGGCGG
CGGGGGCGCCGTCGACGGCAGCGGCGAGACGTGGTGGAAGCACTCGTGCAAGATCAATAA
GGATCTCGTACGTGCATGTGCATGTGCATGCATATATGGATGCATTGTTATACTGCTTTA
TTTACCGTACGTAAAGCTCAGAGAGTGCTTTTGCATTCGCGTACGTGCAGCCATGCGAGG
AGGCGCCGACGGCGCTGTCGTTCCACTACTGCACGAGCTTGAGGGTGCATGACCTGAAGA
TCGTCGACAGCCAGCAGATCCACGTGTCCATCGAGGATTGCACCGGCGTGCAGTTGACAG
GCCTGTCGATCACGGCGCCCGGCACCAGTCCCAACACCGACGGCATCCACATCACACGCA
GCAGGGATGTGCAGGTCACCAACTGCAAGATCAAGACTGGTAATTAAGCAGCATTTAGAT
TTAGCAGACTCTATTAGAGTTTGGCTTGTTGCTAAATTCTGATGATGGACTTCAATTGCA
TTGCATTGCAGGTGACGATTGCTTGTCCATCGAGGCAGGGACGCACGACCTCCACGTGTC
GCAAATCGTGTGCGGGCCAGGTCACGGGATCAGCATCGGGAGCCTAGGAGACGACAACTC
GAGAGCCGAAGTCTCCGGCATCACCATAGACACGGTGCAGCTGTACGGCACGACCAACGG
AGCGCGCATCAAGACGTACCAGGGCGGAAGCGGGTACGCCAGGGACATCACGTTCCAGAA
CATGGCCATGCACGGCGTCGAGAACCCGATCGTCATCGACCAGGACTACTGCGACAAGCG
CCAGGCTACGTCACCGCCGTGCGAGGCGCAGGGGTCGTCGTCGTCGTCAGCGGTCGAGGT
CAGCGACGTCGCGTTCAGGAACATCCGGGGCACCACCGTCAGCAAGGACGCCATCAAGCT
GAGCTGCAGCTGGAACGTCCCCTGCCATGGCATTACCCTGCAGAACATTAGCTTGGAGAT
GCTGGGCGGCAAGGGTACGGCGGAGAGCACCTGCCGGAACGCGAGGTGGAGAACGTCCGG
GACGGTTCTTCCGCAGCCGTGCACCTCATTCATAGACCTGGGGCACGGTTCCAACTGAGC
AGATTTGTTCGGAGAAGCAGCATGCGCGACATAGTGTGTGTGTATATTACACAGTATAGG
GTATCTCAATGAATCGATTTCTGGTTACAGGAGATAAATTAGGGTGTGTTTGGTTGTAAT
GACAGAACAAGATAGGATATGATGATCGATTAAATAAGGTTCTTTGGGTTAAGTACGTCA
G
>ZmPG52
CCAAAGAAGTTGGGTGCTAATGCAGTAATGCTCGTCCCTTCCTCTCCTCTCCTCTCCTCG
TCTCCTCCCCTCCATTCGCCTGAGCCTCCTCCCCATCTCCGGATGGGTGGCTCCACCACC
ACCGTCGTCGTCATGCTGATCGTCGTTCTTCACTACTACATGCTACTAGTAGACGGCGCC
AGTTGGGACGAAGTTTCCTCGGAGGCTGACGGGCCGGCGCCGGCAGAGGGTCCAGACGAC
GAGCTGTGGCTGCCCAGGGCCGGACCGCCACCGAGGGTCGTCAACGTCGACGACTACGGA
GCCGCGGATGCCGGCGTCGACGCCGCCGAGGTGAACCCAGCTTTATTTAACTCGCTCCCT
GGCCCGTTTGTGGATCCCGATCGACCGCGGTTCTGATGATCTATCTCACACTCCACACGC
GTTTGGAAACAGGCTTTCCTCGCGGCGTGGAGCGAGGCCTGCAACTCCTCCGCCGGGCGG
TCCGTGTTCCTCGTGCCCGAGGGCAAGGCGTACCTCCTGATGCCCGTGAGCTTCCGCGGG

CCCTGCAGAGCTGCCTCGGTCGCTGCAATGGTGAGAGATGAGTGAATACTATGCTATCCC
GCGCGCTAGCTGCTGCTCTCTCGTTAGCTAGTGTTACAAGCTAATTAAGCAGATATGGTC
AGATAAAGGGGACGCTGGAGGCGCCGTCGGACCGGTCGGTGTGGCTGGAGCGCGGCCTGC
AGGAGTGGATCACGTTCGAGGGCGTCGACCGTCTCCGCGTCCTCGGCGGCGGGACGCTCG
ACGGCAACGGGATGCAGTGGTGGATCAACTCGTGCAAGCTCAACAGATCAGCGGTACGCT
TGCGGCTTGCTTAATGATCAGTCGTCGTCGGTGTTTGCTTAACACTAACATTAACATGCC
ATGCGTATTTTTTTTTATTTTTGTTTTTTTGTTTGTGTATCCACCACCACCACCAGCGCT
GCGTCACCGGCCCGACGGTACCTTCCCTGACCCGGCAACCCCGATGTATGTATGTATGTA
TGTGCGTCTGAGTTGGCGAGCTTGGTCAGGCGCTGTACTTGAGGAGGTGCACCCGGCTGG
TGGTGGAGGAGCTGCAGGTGAGGGACAGCATGCAGATGCACGTGGCGATCGCCTACTCGC
GGGACGTGGCCGTGTCGAGGCTGTCCATCACGGCGCCGGCGTGGAGCCCCAACACGGACG
GCATCCACGTGTCCAACAGCAGGGAGGTGTCCATAAGCAGGTGCACCATCGCCACAGGTA
CGTGTATGTGTATGTTGTATGTATGAGGTGCCCCTTCTTCCTTCCTTTTCATGTGCGTGT
GTGTGTGTCCAACACCATGCAACAACAATGCAGGTGATGACTGCGTATCCATCGTGACTG
GCTCAACGTTCGTACGGGTGACCAGCATATTCTGCGGACCGGGCCATGGAATAAGGTATA
CGGCGTTTAATTTCACGTTTTCAAAATTTGGCATCATCTGTAAAAAGACATGATGGCAAC
ACACACAACACAAGCTCTACGACTGTGCCTGTGCCTGTGCCTGTGCCGCAGCATTGGTAG
TCTAGGAGCAAACAACTCATGGGCCCACGTCTCCGACGTCCTCGTGGAGAAGGCCACGCT
GCTGGGCACGACCAACGGCGTCAGGATCAAGACCTGGCAGGTAGGTAATGCACGATCTCC
TCGCTTGTGTTGTTGTAATAACTTAATTGGTTAACACACGTACTGCACTGCAGGGAGGGT
ATGGCTACGCCGAGAGAATTAGCTTCCGAGACATATCGATGCGCAACGTCACTAACCCGA
TAATTATAGACCAGAACTACTGCGACTCCGCCAGGACGACGACACCGTCTTCTTGTCACG
ACCAGGTATATATATATATATCTATACCGTCGTCCTACATCTACAGTACGTTGGTCCAAG
TTTTCTCTGCTGCTTCTTCGTGTCGTCGTCAGATCTTCCAGAGTAACTCTCGTGCGCATG
CATGCATGCACGCACACTTTGCACACACAGGGATCGGCGGTGGCCGTGCGTAATATAAGC
TACAGAAACATACATGGAACCAGCGCTTCCAGAGTCGCCATCAGTCTCGTCTGCAGCGCC
GCTCTGCGTTGCGACGGTATACGGATGCAGGATGTCTACCTGGTCGGAGAAGGAAGATAC
GCTACGTGCTCTTACAGGAACGCCACCGTCGTACAGTCTGGGTATACCTTCCCCTTTTGC
AGTGCGGAAATGTAGTACTAGTAGCACCTACACATGAACTTAGGGCTCATTTGGTTTAGC
TTTTGAACCTGTTTTTGTTTTTGTTTTTACAAAAAGCCAACCAATATAATGAGATGAAAA
ATCATAGTTGTGTGCGCAGTACAAACACA
>ZmPG27
CTCCCGTCCTCCCCTCTCCTCTCATCAACCTCTGCTGACCTCCCCGACCCCCGAGGCCGT
TCGTTCGGTGCCTGCACTGCGCAGCAGCGCCGGCTGCTGGAGCTGGTCGGCGCGCGCAAC
AATGGCCAAGGCCGTGCCCTCGCCGCTCGCGGCCGTGGCCGTGTTGGCGCTGCTCCTCCT
GTGCCGCGGCGCCGAGGCCCGCGTCCTCCTCACGCTCGACGACTTCGGCGGCGTCGGCGA
CGGCATTGCCAACGACACCCAGGTACGTACGTCGGTACGTGACCTGCTTGCTTGGCAACG
GTGGACCGACGGCCGGCCGCTCTGTTCCGGAATCCGCAGGCTCTCCTGGACGCGTGGGCC
GCCGCGTGCAGCTCCACCCAGGAGGCCGTCCTCGCCGTGCCGGCCGGGAAGGTCTACCAG
ATCTGGCCGGTGCAGCTCTCCGGGCCCTGCAAGAAGAGGCTCAAGCTGCTGGTACGTCAC
GTGCCCTGCCCCTGTCCATCCTGGCGTACGTTATACGTACTCGTTGCTCGCTCGATCGGT
CGCTCATCAACGCTGGTCGGCTGGTGTGGATGTGGTGGTGGCGTGGCGTGGCGTCTCGGC
CGCTGTGCGCGCTAGATTTCCGGCGCGATCGTGGCGCCGTCGAGCCCCGACGAGTGGGCC
GGGCGGGACCCCATGAAGTGGCTCTACGTCTACGGCGTCGACGGCCTGTCCGTCAGCGGC

GGCGGCACCATCGACGGCATGGGGCAGCAGTGGTGGGCCAGCACCTGCAAGCGCAAGAAG
ACCCAGGTAAGTAACAACCAACCACCTAACCCGTCTGAGCCTGCACCTTTTCGCAATGAA
AGCCGCCTCCCTGAAGTCTCCTACTCTTGCAGCCGTGCTACTCGGGGCCTCGTCCAAAGG
TGAACGAACGCTCTGCTCCTCTTTGTCTCAGCTGGTTGATCGCCGACGGTGGATGCGATC
CATCCTTATTCCGTCTTTTGCGTTGCATTGCATGGCATGCAGGCGGTGCACTTCGAGGAG
TGCCGGGAGGTGAGCGTGCAGGGCGTGACGCTGCAGAACGCGCAGCAGTTCCAGCTGACG
TTCACGCGCTGCTCCTGCGTGAAGGCCAGCTTCCTCCGGGTGGTGGCGCCGGCGGACAGC
CCCAACACCGACGGCATCCACCTCAACGACACCTCCCACGTCCGCATCACGGACAACCTC
ATCTCCACAGGTCCGTTGTCCGTCCACGCTTGCCCGATTAAATTATATATATTCGCAGGC
ACTCATCAGTAAGACCGTCAAAGCCGCCGCCGGTGACTAGTTGACCGGACAGATTGAAGT
CCAAAATCGCTGCTGGCCTGCTTATGCTGATGCGTGTTGCACATTGGTTCCTTATTGCAT
CATACAAATTTCCAAATCTGTAATTACTAGTATCGTTATTGTTGAAGAAATATTCGTGGC
TGGCTGCTGCTAGTATTTGACTAAAGCGGTTCTTCACGCTTCCGTGCAGGGGACGACTGC
GTCTCCATGGTCGGCAATTGCTCTGACGTCCGTGTGAAAGACATCTCATGTGGGCCTGGC
CATGGCATCAGGTGACTCCCTGCTCTTCTACTCCTCCATTCCAGGATCTGATCTCTGACA
CAGGGACTAACAACGTGCGCGTCAGCATCGGAAGCCTTGGAAAGAACCGGACCACCGACA
TGGTGGAGGACGTGAAGGTCGACACCTGCTTGCTCACCAACACGACCAACGGCGTCCGTA
TCAAGAGCTGGCAGGTAAAACACCTGACGCTGTGTGACAGGAGCAGAGAGCCACCACACC
CTCCGGCTCCAACACGTGCGATTTCTTTCAGGGAGGCACGGGCTTCGCGCGGGACCTGCG
GTTCGAGAACATCGTGATGAGGAACGTCTCCAACCCCATCATCATCGACCAGTACTACTG
CGACCAGCCCACGCCCTGCGCCAACCAGGCACGCCATTCCTCAGTTCATCTGAATATCCA
GAGCCAGAGCACAGCACACTGCTTGCATTGATGTCTCCGGTGGTATCTGAGGTCTGAACA
GCTCTGCATGTGGCGGTGCAGACGCAGGCGGTGGAGGTGCGCAGGGTGGAGTTCGCGGGC
ATCCGGGGCACGTCGGCGACGCCGCAGGCGATCAGCATCGCGTGCAGCGACGCCGTGCCG
TGCCGGGACCTGGAGCTGGCGAACGTCAACCTGACGCTGGAGGGCGGTGGTGCCGGCCGA
GCCACCGCCCTCTGCTACCGGGCGTCGGGGAAGAGCGCCGGCACCGTGGTCCCGCCGTCC
TGCCTCGCCAGGTCCTGACTGAGACTCACCTAGCGAAGCAGTGGCCGAAGCGCGTATACA
CCCACATGCTGCATGGCTGTCGATTGGAGCTGTTGCCATACCCAAACAAAACCAACCTGT
TGATTGTACACGCCGTTGTTGACGACAAGGAAACACAGAGAAGAAATATCGGAGCTTCTT
ACCTGCAAAATGTCGTTCGCACTTAAAAAAGGTTCGGATTAAAAAAACACAA
>ZmPG26
ACGGCCTGTGCACGCGCTGACGCGCGCGCGCCACCCATCCTTGTGACCGTGTCCGTCGGG
CGAGCCTGCGGTCTGCGCGCTTCAAGTAGCACCCTACCGGTGCGCGCGCACCTCAAAACG
TAGTCAAGTGAGCGGTCTCATTCTGCTCTGCTGCTGCAACTGCAAGCGCGGAATCTGTTC
GATGATGGCGCGGGGCGCCGTGCTGCTCCTGCTCGCGGCCGCCTTCGCCGCGGCGCTCCT
GCCGGACGCCGCGGAATCCCGCATCCTCCTCACGCTCCACGACTTCGGCGCCGTCGGCGA
CGGCGTCGCGGACGACACCAAGGTACCCCCGCAGCGCCCGTACGCACGCTTCAGCGCGGC
CCCAATGCGACTGAGACTGACCGCCAACGCCTCCTCGTCTCTCTCCCCAAGGCCTTGGCC
GGCGCGTGGACGGCCGCGTGCGCCGCCGCGGACGACGTCATCCTCAACGTGCCCGCCGGC
GGGACATTCCGGATCTGGCCGCTCACGCTCGCCGGACCCTGCAGGAGCGAGATCAAGCTG
CTCGTAAGCACGCTCAGCAACCGTATAATCCGTTACCGTCTCTCTGCTCACGCGCACTGG
CTCTGTTCCGTTCTTGCCTCACCCACCCTCCCGTCGCCGGGCGCGTTCTTGCCTCGCGGT
CTCTCAGATTTCCGGGGACATCGTCGCGCCGGAAAGCCCCCAGGACTGGGGGCAAGGCCA
GAGCGACCAGTGGCTCCACTTCCACAAGGTTCGGGACCTCACGGTCACCGGCGGGGGCAT

CATCGACGGCAGAGGGCAGCAGTGGTGGGCGCAGTCGCTTGCGCGCGCGCAGCCCGCTCC
CAAGGTGAGCTTACGCGCGCGCTGTTCGATTGGAGGTTGGCTCCAATGGAACTGGAGCCG
GCCACCGAGGTTAGGGACGGAGTTCCGAGCAGCAAGCCAGGAACTCACCGCGTGTTGTCT
TCTGCTGCTGCTGCGTCTTTCTTTCATCCGCAGGCTGTTCACTTCGAGGACTGCCAGGGG
ATCAGCGTGAAGGGCATCACCTTGCAGAACAGCCAGTCGTACCACCTGACGTTCACCCGG
AGCTCCGACGTCGAGGCCAATTACCTCAGGGTGACCTCGCCGGAGCACAGCGTCGACACC
AAGGGCATTCACCTCGTTGACTCGTACAATGTTCACGTCATGGATAACCTCATCTCCACA
GGTATCTGTAATCTTCCGACCCTCCATTCCCCTGCTTCATACAGGTTCAGCAGACGAGTT
TGGTTTTCAGAAAACAATCTCGCAACAATGTATGCATGTGCCAGGGAAAAAAAAGTACGC
TTCACTGAAGTTGGCTAGAGATTCAGTAGAACGAACACTGGAAACACTTACTCATTGCAC
AAACTTTGGGTACTGTTAAATCGTTGAGCATGTCATGTGATTTGCCCCGGTTTCCCTTCG
TTTCGTGCAGGTGATGACTGCGTCTCCATAGTGGGCAACTGCACGGATGTCCGTCTAAGA
GCCATCTCATGCGGACCTGGCCATGGTATCAGGTACCTTGCTAGCTGACTGTCGTGGTGT
TGCCTCTTCAGATTTGTATCTCCCCACAATTTCCTGGTTTGCTAGCACAGCTAAGCTAAT
AGTACTCGGCTGCGATTAGCATCGGAACCTTAGGAGTAAACAGCTCCGTCGATTACGTGG
AGAAGATCAAAGTCGACACCTTGTTCATCTCAAACGCCGAGAACGGCGTGCGCGTGAGGA
CCACCGAGGTAACAGCAGCCGACAGTTTCGCAATTTCTCCACCTGAATCAGAGTAGCCTG
CTCATATGCGCGAGGAAGCTCAGGTGAAAACACCGTAAAAGTAAAACCTTTGTGGTCTGT
GTCTTCTCCAGAACGGCGGCGGCGGCGGCTTCGCTCGCAAGGTGAAGTTCGAGAGCATCG
TCATGAGGAACGTCACCAACCCCATCATCGTCGACCAGGGGATCTCCTCTGACGATCCGC
CACCTTCATCGCCTGAAGCAGTAGCACTGGTACACCACACACTGATCAGACTTCCATCAC
TGTCTGCCTTTACGCCGCGGGACGCCGCAGCATACATTAGTGTCCGGCAAGCAGTGTCTC
TGAACTCACGCGCCGGAAAATTCTGCGCCGCCCAGGCCGCGACGGCGTCGGCGGCAGTGC
AGGTGGAGAAGATCAACTACATCGACATCACGGGCACGTCGGCGTCGGAGCGCGCGGTCA
CGTTCTCGTGCAGCGACGCCCGGCCGTGCAGGCGCCTGTCGCTCGACAACGTGAACCTGA
CCCAGGTGGACGGGAGCGAGGCGTCGTCCTACTGCCGCCAGGCGTTCGGGAGGAGCGTCG
GCACCGTTGTCCCGGAGTCCTGCCTCTCCAAGGAGGACTTCGTCCACCACGTCCCGCCGC
AGCGTTCTGAGGAAGACGGAGAAGACTCAGAATCGTGA
>ZmPG22
CACCTCCCGCTGTCGCACGCTCGTTTTTTTTATCATTCACTTGCACTTCAAGTCCTCCTC
CTCCCCTGGCTTCTTCTTCTTCCTCCTCCTGCTGGCTGCTGCTGCTCTTCTTTCTTCCTC
CTACTCGATCCGTCTCCACCACCTCACACGTACGGTACTCATCATCTCCCATCCGAAACC
CGCTCTCCCCTGCCAGAGCTAGCTAGATCTATATAGCCTGCAGTCCTGGAGCTTGCGAGC
AAAGGCATACGTACATACATACATACGCGTTCGCCGTGTCGGGCCGTACGTACGTTGTCG
TGCGATGGGTGCGGCGCCGGCGGCGAGGAGGTGCGCGCTGCTCTTGGCGCTGGTGGTGCT
GGTCGCGGCCGCGGGCGAGCGCGGTGGCAGTGGTGCCGGCGGTGAGGCGGGGGCGGAGCA
GGTTCTGAGGCTGTGGTCGTCGGCGGATTCGGCTAGGGGTGGTGAAGCCGTGGACGAAGA
AGACCGCTTCTTCAGGTGGGAGTGGGAGGAGGAGGAGGAGGACGACGACAACAACGACGA
GGAGGAGGGGGAGGAGGAGGACGACCACGCCGTCGTGGTGCAGCGGAAGGGCGCGTGCAG
GAACGTGGTGAACGTCGACAGCTTCGGCGCGGCGGGCGACGGCGTCGCCGACGACACCCA
GGTGCGTGCGCGTTGCCGGTCGGCCATCGACACTCACAGATACGAAATGATGGGTGTATA
TATATGCGTGGGTGGCTCGCATTGTCTGTCTGGATGGGTTGACAGACTGCCTCAATGTGG
TTGGTTGATGCCGATGACGCCGGCAACGCAACGCAATTACGCAATGTATGGCCATGGCGG
CGACTGCTTGACTGGCTCTCATCGTCAGTCGTCACTTGATTATCAGGCGTTCGCGAGCGC

GTGGAAGACGGCGTGCTCGCTGGACAACGCGGTGTTCCTGGTGCCCGCCGGCCGTCGCTA
CAAGGTCGGCGCCATCCAGTTCGTGGGTCCCTGCAAGGACAGGAGGATGATCATCCAGGT
GACTCGTGTTGCTTCAGTTCAACTCAATGCCTGAGCCGTAGCCGTAGCACATGCATATGC
ATTGTAAAGAACGGCGGTGGTTGGATGCATGGTGCAGATCCAGGGCACGGTCGTGGCGCC
CGAGGAGCCGTCGGAGTGGGACCCGCGGAGCCCCCGGCTGTGGCTGCTCTTCTCGGGCCT
CGCCGGCGCGCGCATCCAGGGCGGCGGCGTCATCGACGGCTCCGGCTCCAAGTGGTGGGC
CAGCTCCTGCAAGATCAACAGGTCCAACGTACGTACGCCGCCGGTGCATTTGCCTTCTCT
GCCTCGATCACACCCACACTACTGCTCTGCTCCTTAATTGGTACCGCTCCGTTGACGACT
GACTGACACACACGCCGCTCTCTCGCCCGGCGCCGGTCGCGTGCGTGCAGCCGTGCAGGC
CGGCCCCGACGGCCGTGACGATCGACTCGTGCCGGGGCGTGCGCGTGCGGGGCCTCCGCG
TGCAGAACGCGCAGCAGATGCACGTGACGGTGTCACGCTCACGCGGCGTGCGCGTCGCCG
GGCTGCGCATCGACGCGCCCGAGGACAGCCCCAACACGGACGGCATCCACGTGGCCGAGT
CCACGGCGGTCACCATCCAGAGCTGCCGCATCGGCACCGGCGACGACTGCATCTCCATCG
TCAACGGCAGCTTCGGCGTCAAGATGCGGGACATCGACTGCGGGCCCGGGCACGGCATCA
GGTGAGCTCAGGCGCTCAGCTAGCTCTTCCCCTTCCATTCCTCGACCCTGTTCACGCGAC
GCGGTCGCAACGCGAGCTGTGCGCTTCACTTATGGCCATGGCGACGGCGTACGACGCTTG
CAGCATCGGGAGCCTCGGCAAGGGCGGCGCGTTCGCGGCTGTGGCGGACGTGGCGCTGGA
CAGGGCGCGGATCAGCCGCGCGCAGAACGGGGTGCGGATCAAGACGTGGCAGGGCGGGGC
CGGGTACGTCCGCGGCGTGCGCTTCGCCGACGTGGCCGTGGACGGCGTCGACCACCCCAT
CGTCATCGACCAGTTCTACTGCGACGTGACCCGCCGGCAGAGGGACGGCGGCGGAGGCGG
CGCCTGCGCCAACCAGACCTCCGCCGTGGCCGTGTCCGACGTGTCCTACCGCAACATCAG
CGGCACGTCGCGCCGCGCCGAGGCCATCCGGTTCGCCTGCAGCGACGCCGTGCCGTGCAC
CGGCATCGTTCTCAGCAACATCAACCTGCGCCGCGCCGACGATGACGGCGAGGTGCAGAC
GGTGTGCAACTGCGCCGTCGGCCTCGACTACGGCCGCGTCCAGCCCGCCGCCGACTGCCT
CCGTAGCAGCACCTGCGGCGGCACGCCTGATGGCCACCACCACCACGACGACGACGACGA
GGAGCAGGGGAAGGACGACGCCGTCCTGCACACCGAGCTCTGATAAAAGCAAGCAGCTGC
ACGCCTGCAAGTGAAACCAATGGCCGCCGCGCTCGTTCGTCCGACATTTAGGAGATTGAT
TGGTCTAAAATCTCTTATTATATATTTTTAAATAGTAAGAAATTATAACCCTCGAATCTT
CTCCCTTCCATTTGTTCACAAACAGACCTTTACTGGGGTAACACTGAATGCTAATGCTTA
GCGAGAGGCTCTCTCCAACAGAGCGTGTAAAGGCTCCTCTACCATAAATATGATAATCGA
ACGGTCATCTACTCTCTCGAGCAAAGTTCTCTAAATTTAGAGAACTT
>ZmPG13
ATGGCGATCATCGTCAGCTCCTCAGCTCGCTCGACGCTTTTTATTCTATCGTCCATTACC
AACCATGTCTACCGCCGACAGGTCGTCTCCGCCCTGAAGACGACGGCCGCCGCAGCGAGG
CGGCATACGGCCTTCGCCATGCTGCTCGCCTTCGTCACGCTGCTGCTTGTGCTGGCCACT
CGACCGGCGCAGTGCCACCAGCAGAGCTACGACGTCGTCAGGAACTTCCACGCCGCCGCG
GATGGCAAGACGGACGATGCCAAGGTAAACGCCAAAAAAAAAAAGCGTTATTGCTTGCTTC
TACTTCTACCGCATCGATCCCGGCCTTTCTTCTGGGACTTGTGACCATCGTCGCTGCCAA
AACCAGGCGTTCCTGGCGGCATGGAAGGCGGCGTGCAGCGACGAGGCCAGGCCAGTCGTG
GTCGTCCCGGGAGGACGGACGTTCCTGCTGAGCCAGGTCACGTTTCAGGGCCCCTGCAAG
TCACCCATCACCATACAGGTAAACAAACACCCCCGCGTCTTGTATAGTAAACAGCAAAAC
AACAAGAAGCGCGGGCGTCTGAGTTCTGAGCTCGGTACGTGCAGCTGGATGGCAGGATCG
TAGCGCCGAACCGCATCTGGACGACTCAGCCAGCCAACCTCCTGGTCTTCCACGGCGTCG
ACAGCTTGACTCTGGACGGGAACGGCGAGATCGACGGCCGAGGGGCCATCTGGTGGGACT

GCTATAACCACAAGGTAGGCAGGCTGCCTGCACCGTTTTCTCCCTCCCTCCCTCTCCCTC
TCTCTCTCTCTCTGAACCTTGGGAATGATCTGACATGCTGTTTCTCGTCTTTCCTTGACA
GAGATGCAACGCCCGGCCAATTGTAAGTATTCGACACATATATACAGCTCACAACTCACT
CTCGAACCAGCTGTTCATCAGGTAGACATATACCATATGTGAGTGAGGCACCGGCGCCGC
TGACGACGACGCTGCTTGTGTTGGTGTGCGAGATCAGCTGCTGGCCTTCTCGTTCTGCGA
CCACCTACGGGTCACGAGGATACGGCTGACCAACAGCGCCGACAAGCACATGACCCTGTT
CCGGTGCAGCCAGGCGCTCGTGGACGGCGTCTCCATCGCCGCGCCGCCCGACAGCCCCAA
CACGGACGGCATAACCGTGGCGTCGTCCAACCACACCGTCATCTCCAACTGCTCCATCCG
AAGCGGTATATATAGAGGATCGGAACGCCCTTCCACTTCGTCGTCACAAATTAAGCGCGC
CATTGCAGCACAATACGTAGCTGAACTGACCATGTCGTTCACGGATGCTGACTGACTGTC
ATTTGGGGAAAAAAATGCAGGAGATGACTGCGTATCGATCCTGTCGCAAACGAGAAACGT
TACCGTCACTCGCAGCACCTGCGGGCCTGGCCATGGTATCAGGTAACACAACACGTGTAC
ACTAATTAACAGAGGGCATCTACTAAGCACCGAACATCTTTTCTGATCCTTGTGTAGTGT
GGGAAGCCTGGGAAGATCCGAGAGCGCGGTGGTGGAACAGATCGTAGTGACTAACTGCAG
CTTTGTTGGGACCATGAATGGTGTCAGGATAAAGTCATGGCAGGTAGCTAAGTCTCAGGG
CTTAACGGTTTTGTAACGAGGCTGCATTGCATACGCTCTGTACATATGCTTCAAGCCTCA
AATCGAACAAGGCTAATGCATCAAGCCATGAATGCAGGGAGGGAAGGGCTACGCAAAAGG
GTTCCTCTTTGCGGGCCTCAACATGACCGGAGTGCAGTATCCCATCGTCATCGACCAGTT
CTACTGCCCGCAAGGAAACTGTCCTGTGAAGGTGAGCCACACTAGAAACCACTGCCGTGC
ATGGCTACGGCCTACGGGGTACCTGCACTTCGACTGTCTCTGTCTCTGTCTTTGCAGCCT
GGTGGCGTGGCGATAACCGACGCAAGGTTCATCGACATCCAAGGGACGTCATCCAGGCAG
GAAGCCATCAGGCTGCTGTGCAGCCAGAGTGTCCACTGCCATGGGATCTACCTCAGCAAC
GTCAACCTTTCCTGGGTCAACCACACCGCCCCGTCCAACGCTACTATTCTGAACGCCCAC
GGGACAACCGAGGGCGTCGTCGTGCCAAAGATACAGTTTTTACAGCCTTTGTAG
>ZmPG51
ATACGCAATGCCGCCGGCGACAAGCACGCGCCCGCCGTTTCGTCGCCCGGTACCATTGCT
GACGGTGGTCGTTTCCGTGGCGCTCCTGTCACTCGTGTGCTTTCTTGTAGCTGCTGATGC
AAGCAGATCGCTGCACTACCACTACCACCTCCGGAAGCACCGGAGGCACCACGGGCACCA
CCGCGCGGAGAACTACAGCCACATCTCCCTGCCCCCTGCCGCGCCGCCTCCTGGCGCTGA
TGGCGATTCTCCGGCCGGACCGGTGGGTCTGCCTCCGGACGTTGGCGGCGCGCTGCCGCC
GAGCTCCTGCAGTGACAAGCCGTGCCCGCTTTCGCCTAGTCCTTCCAGGGCACCGGCAGG
CGCGCCATGTTCGACGCCCGGTTCGGCCAAGCCTCCGGCTTTAACCTTGGCCACTCCGCC
GTCGCCCGCGCCAGCCACTCCACCATCTCATTCTCCTCCGAATCCACCGTCGTCGCACCA
ACCGTTTCAGCGGCCCACACTTCCTCTTGCCAAGGCGCCGCCGTCGTCCAAGCCACCGTC
GCCGGACATCGCACCATTTCATCCTCACGCCAAGCCGCCGCGCGCGCTTCCAGACGCCAA
GTCGCCGTCACAGCCACCAAAAAGGGCGCCAGCACCATCGCCGTTGTCTCCTTCGAAGCC
GCGCAAACCGCCATCGTGCACTTTGGCTACGCCACCACAGGCCCAGACATCGAGACTCGC
CCCTGCCAGTGCCAAGCCACCAATGCGATCACCAGCCCATCCACCGAAGCTCTCCCCGGC
AAGCCCACTGGCGCACCCACCGGTCGAGCCGCCAAAGTCAGCTCTTGCCAAGCCGCCTGG
TTTCTCTTTCTCCCATACTACCAAGCCACAGCCACCAGTGGCTTCGATGGTGAAGCCACC
ACGACTCGCCCCGGCCAAGCCTCCAGCGCCGTCGCCGTTGCCTGCAGCCCAGCCACCGCG
GTGCTCCACGGCAAATCCGCCAGTAGCTCCTACGGCGTCAGCGAAGCCACCGGCGTACCC
TCCTGCTGCTGCGTCAAAGCCAATTCCGCCGCCGCCGCTCCCACCGGCAGCCAACAACAG
CTCATCAGCCTGGGGCAACGTGTTCGACGTGAGGGCGTTCGGGGCGTCGGGCAGCGGCTC

AGGCAACGACACGCGCGCGTTGCGCGCTGCGTGGAAGGCGGCGTGCTCGTCCAACTCCAC
CACGCCCACGCTGCTGGTGCCGTCGGACGGCGTGTTCACCATCAGCTCCACCATCTTCGC
CGGGCCGTGCAAGTCCGCTGTGACCTTCCAAGTAAGTTCGATGCTGAGCTGCCTCTTCCA
AAGCATTAACCCAAGCTAGCCAAGGCTGAATCTTCACGCTGCCGTGACGACTGTGAACCG
CGTCGCTGCAGATCGACGGCGTGCTGATGCCGCCAGACGGGCCGGCGAGCTGGCCGGCGT
CCGACAGCCGGAAACAGTGGATAGTCTTCTACAAGGCCAACGGCATGACGCTGGCAGGGG
AAGGCACCATCGAAGGCAACGGGGAGGAGTGGTGGGACCTCCCGTGCAAGCCTCACAGGG
TACGTACGTATGTTTGTCATGGCGTCTACCTGGACCAAACATGTGTGACGTCCGTGTCTC
TATGTTCAGGGCCCCAACGGATCAACGCTGCCCGGGCCTTGCGACAGCCCTGCAGTAAGC
TCGCCGACTCACATGGATTTCGCTCTGCTCTTTGTCCGTGTCCCTGGCTTCACCCTGTTC
GTGGTATCTGCAGCTGGTACGATTCTTCTTGAGCAACGACGTGACGGTGCGCGGCCTGCG
GATCGAGAACAGCCCGCAGTTCCACCTCAAGTTCGACGACTGCGAGAGGGTGCGCGTGGA
TGGCCTCTTCGTCCGCTCGCCGGCGTTCAGCCCCAACACGGACGGCGTCCACGTCGAGAA
CACCACGTCCGTCCAGATCCTCAACTCCAGGATCTACAACGGTACGCTGGCTGAAGCCGG
AACTGGCAAGTCTCGATTCATGGTCATGGACCACTGAAAAATCGATCGTGTGTGTGACTG
CACTGCATGCAGGCGACGACTGTGTCTCCATCGGCGCCGGCTGCTCCGACGTCCGCATCG
AGAACATAACATGCGGCCACGGCCATGGCATAAGGTAGGTAACCTGTACGTCAGCGTGCA
TCTGTGTCTGGTGGTGGTGCAGTGCTGCTTTGAATGTGTCAGCTGACGCGGCAAGCTCGT
GGCGACCTCGCAGCATCGGCAGCCTGGGCGTGCACGGCACGCGCGCCTGCGTGTCCAACA
TCACGGTCCGGAACGCGCGGATCCTCGACTCCGACAACGGCGTCCGGATCAAGACCTGGC
AGGGAGGCACAGGCGCCGTGACGGCGGTCGAGTTCGCCGGCGTGCAGATGCAGAACGTGA
AGAACTGCATCCTCATCGACCAGTACTACTGCCTCGGGAGCGGGTGCGCCAACCTGTCCT
CCGCGGTGCGCGTCGCCGGGGTCACGTATCGGGACATCCGCGGCACGTACAATCCGCAGG
CCAGCGCGCCCATCCGCCTCGCGTGCAGCGACGCCGTCGCCTGCACGGACATCACCATGT
CCGGCGTCGAGCTGTTGCCGGCCAGCGGAGGAGGCGCACGAGCGCTGCTTGCGGACCCCT
ACTGCTGGAACGCGTACGGAGTCATGGAGACGCTCACGCTGCCTCCTGTGTACTGCTTGC
AGGAAGGCCGACCGGAGTCGCTGCAAGATCAGCTCACCAGTTGCTGAGATGACACTGTTT
TCTTTTATCTAACCAAAAACAAATATATTGCCGATGACAGTACAACGGTAAAAATTGTTA
CAAAAGTTTCACATGTGATCAATCCCAACCGGTGTGACAGTTATTGCCTATGGGTCGCAC
CCTTTGGGGACCCTGTGTACTGTACTATATATATAACTATCTCTGCAATAAAGAAACTGT
CTCAGTCATTTGTC
>ZmPG11
AGGGCGGGAGGAGAAGCTGTGGGTCGATCACTTGGGTCTTGGGTGGCACGCGCTACAAGC
TAGGGCTTCTGCTCCGTCCTCCGCGAATCCGCGGCGAGCCTGCTAGCTGCGTGGCTTCCC
TCTTCCCGGGAGCGGCGCGGCGGTAGTGCTGGTGCTGCCCGGACCAGACCAGACCCGAAA
GCGGAGAGGCCCCCGTGCAACCGGAGCCGTGCGTTGGAGGTGGACAGGTGGTGGCACCTG
TGGGCAACGGCCAACGGCACGGCAGGTACACCGTACAGCTCTTGTGTCTTGTTCACTTGG
TCCCTAGTCGTTCGTGGTGTAGGATGCCGTCGTCGGCGTGTCGCTGACAATTTAGCGATC
AAGATCGATTCGGTATTTGCGTATCTGACGGTTAAAAGGCTAGGCTGGAGCAATGCACGT
ACGTACGTACTTAGCCTTTGGTTGTTCCATTGCAGTACCATCACTTTGGCTCGTTGTCGT
ATTCAAAACCAGACTTGAGCTACTTAGCCCCCCAGCGATTCATGTAGAGGCCGGGGGCGA
ATCCACACTAACAACATACTTCTAGATTCTGGTCGATCGTCGATGAGGCCGGCGCCGGAG
ACAAGCACGCGGCGGCCGCAGCTGCGTCGCCGTCGTCCAGTGCCATTGGTGACGGTGGCC
GTGTCTTTGGCGCTCATCTCGCTGGCGTGCCTTCCTGTCTTCGCGGATGCAAGCAGAGCG

CTGCACCGCGACCGGGAGGAGCACCGGAGGCAGCACCACCGCACGGAGGACAAGAAGAGC
CGCGTCTCGCTCCCTCCCGACGCTGACGGCGACTCTCCGCCCGAGCCTCCGGACTCCGGC
GGCGCGCCGCCGCCTGCCGACGACAGCGACTGCGGCAACGTGTTCGACGTGAGGGCGTTC
GGCGCGTCGGGCGACGGCTCGTGCGTCGACGACGACACGGCCGCGTTCCGCGCGGCGTGG
AAGGCGGCGTGCTCCTCCGACTCCGCCGCGGCCACGCTGCTGGTGCCGTCGGACGGCGTG
TTCACCATCGCATCCACCATCTTCTCCGGGCCGTGCAAGCCCGACCTCACCTTCCAAGTA
AGCTAAGCTCGAGCTGTGCGTTTGCATTGCATTGCATTGCATCCTCAAAATCATCGAACC
AAGCCAGCAATGCCCCCGGCCAATTTTCACACTGCTGCAGATCGATGGCGTCCTCATGCC
GCCGGACGGCCCGGCGAGCTGGCCGGCGACGGACAGCCGGAAACAGTGGATAGTCTTCTA
CAAGGCCGATCGCTTGACGCTGGCGGGGGAAGGCACCATCGAAGGCAACGGTGAGGAGTG
GTGGGATCTGCCGTGCAAGCCTCACAGGGTACTCTTCTCTTCCGCGGTGCATTAGTCACG
GAGTTTACCCCGAAGAAACATGCGTGACGTTTGGGTCTGTGTCCGTGCATGTCTAGGGTC
CAAACGGATCGACGCTGCGCGGACCATGTGACAGCCCCGCGGTAAAGCTCGCCGGCAAAC
TTGGATTTCTTTTCTCCCCATCATCATGTTGTGTGTGTTTCTTTTGTCGAAATCCTTGAC
GGTCTCACTCTGCTCGTGGAATCTGTGTGCTGTCTGCAGCTCATACGATTCTTCTCGAGC
AATGACGTGACGGTATGCGGCCTGCGGATCGAGAACAGCCCGCAGTTCCACCTCAGGTTC
GACGACTGCGAGCGCGTGCGCGTCGACGGCCTCTTCGTCAGCTCGCCGGCGTCCAGCCCC
AACACGGACGGCGTCCACGTCGAGAACACCAAGTCCGTCCAGATCCTCAACTCTAGGATC
TACAACGGTGCGCTAAAACATAAAGAATGTATCAGCTCTGTATCAACTTCCCATCAAACT
CTGAGCTCTATCTGTCACGCTGCAGGCGACGACTGCGTCTCCATCGGCGCCGGATGCTCC
GATGTCCACGTCGAGAACATAACATGCGGCCACGGCCATGGAATTAGGTACCTGTCAGTC
TGTCACTCACTCACTCACCAACGTGCTTCCATGGCGCGCGGTACCGTTTGTAGTGAACCT
GACGCGGCGAGCTGCTGCTGTCACCCTCGCAGCATCGGCAGCCTGGGTGTCCACAACACC
CACGCCTGCGTGTCCAACGTCACGGTCCGGAACGTGCGGATACTCGACTCCGACAACGGC
GTCCGGATCAAGACGTGGCAGGGCGGCGCCGGCGCGGTGTCGGCGGTCGAGTTCACCGGC
GTGCAGATGCAGAACGTGAAGAACTGCATCGTCATCGACCAGTACTACTGCCTCGGCCAC
GGGTGCGCCAACCAGACCTCCGCGGTGCGCGTGGCCGGCGTCGCGTACAGGGACATCCAC
GGCACGTACAACCCGCAGGCCGGCGCGCCCATCCGCCTCGCGTGCAGCGACGCCGTCGCC
TGCACGGACATCACCATGTCTGGCGTCGAGCTGCTGCCGGCCGGCGGGGACGACGGAGCA
CTGCTCGCCGACCCCTACTGCTGGAACGCGTACGGGGTCATGGAGACGCTCACGCTGCCA
CCGGTGTACTGCTTGCTGGAGGGCAGCCCGGAGTCTCTCCAGGATCCGCTCACCAGTTGC
TGAGATGAGATCGTTTTCTTTACTAACAAAGATTTCTCTCCTGAGACAGTCAACGCTAAT
ACACTGGACATTCTACATGTGACAAATTACAAATTACGCAGACAATGTGAATTTCTGAAA
TGGCATGCTCCCAGCAACTGAAAGTTGAGCCGCAATGTATTCAAGAGAGTGAAAT
>ZmPG49
CAACCCAACCACTGCATCTCCGGCACCAGCAGTCTAGCTAGCTAGCTAGCTCGTTTGCTA
TAGCTTGCCAGATCCCCGTGACCAGCCGTAGCACACTCGCTGCCCTGTTAATATAATGGA
GTTCACCGTCGTCAGGACCGCCATTGTTCTGCTTCTTTCGCTCGCCGTCTCCTCGACCTT
CCTCTGCGGAGGCGTCCAGGGCGGCCGCCACTACCACTACCACCACACCACGCACACAAA
GCACAACACCGCGCACCCACCGTCCGCCCACGCTCCGGGCCCAGCGAGCGGGAGGCGGCT
ACCCCCCGTCTCCCCGCCGCCGTCGGACTCGAGCGGCTACCCGACGCCTGGCGCCGCGCC
GGAGCCAGCGCCGGCCCCCGCGGCAGCCGGCACCATGTACGACGTCGTCAAGGACTTCGG
CGCCGTCGGGGACGGCGTGACGGACGACACCGACGCCCTCAAGACCGCGTGGGACACCGC
GTGCCAGGACGACGGGGACAGCGTCGTGCTCGCCGCCGCCGGGTACTCGTTTCTCGTGCA

CACCACCGTCTTCACCGGCCCGTGCCAGGGGAGCGTCACGATTCAGGTTGGTAGCAGTAG
CGCCCGCCGCCGTGGAGAAATTAAAAATGTGGACTTGATGGCCTTGACTTGGAGAGGCAC
GTAGTACGTGCATGTATGTACGGCATCGCTCAGACTTGCTGATCCCAATGGCGTCGTCCT
ATCGTGTCTATGTGCAGCTCGACGGGGCGATCGTGGCGCCGAGCGACCCCGACACGTGGC
CGGCCAACAGCAAGCGCAACTGGCTGCTGTTCTACCAGGCCCACGGCATGTCGCTGCAGG
GCGCCGGCCTCATCGACGGCAGGGGCCAGAAGTGGTGGGAGCTCCCCTGCAAGTCTCACA
AGGGCCAGGGTGGATCAAGCGGCCACGGTGCATCCTGCGACAGCCCAGTGGTAAGTGGCT
CTCAACTAATTAACGACTTTTAGGCCTTGTTCGTTTCCGTTGGATTGCACCCGGAATCGT
TCCAGCTAATCAAAGTTTATATAAATTAGAGAAACAATCCGGTCAGGAATCGTTCCAACT
CATCAATCCGGCAGAAACGAACAAGGCCTTAACCGCACTATAATTAACCGCCACTTGGTG
TACTACTGGTCCCATGCTCATGGCAACATCCATCATCGTCAGGCGCTGAGGTTTTTCATG
ACCAACAACGTGACGGTACAAGGCCTCAAGGTGCAGAACAGCCCCGAGTTCCACATCCGG
TTCGACAGCTGCTGCGGCGTGGTCGCCAGCGGCCTGTCCATCAGCTCCCCGGCGCTGAGC
CCCAACACGGACGGCATCCACGTCGAGAACACCGAGGACGTCCTCATCACCAACACCGCC
GTCTCCAACGGCGACGACTGCGTCTCCATCGGCGCCGGCACCCGCAACATGCACGTCGAG
AACGTCACCTGCGGGCCCGGCGGCCACGGCATCAGCATCGGGAGCCTGGGCAAGCAGGGC
TCGCGGGCGTGCGTGGCAAACGTCACGGTGCGCAACGCGGTCATCCGCCACTCGGACAAC
GGCGTCCGGATCAAGACGTGGCAGGGCGGCTCCGGCGCCGTCTCCTCCGTCTCCTTCGAG
AACGTGCGCATGGACGCCGTGCGCAACCCCATCATCATCGACCAGTACTACTGCCTCTCC
AAGAGCTGCGACAACGCCACCTCCGCGGTCTTCGTCTCCGGCGTCTCCTACGCCGGCATC
CGGGGCACCTACGACCCGCGCACCACGCCCATCCACTTCGGATGCAGCGACGCCGTGCCG
TGCACCAACATCACGCTCTCCGACGTCGAGCTTCTCCCCGCGTCCGGCGACAGTACCATC
GACAACCCCTTCTGCTGGAACGTGTACGGCAGCACCGCCACGCCCACGGTGCCGCCGGTG
CCATGCCTCATAGAAGGCGTGCCCAGGAGAGTCGACGATAACAGCACCCTCAAATGCTAC
TAGATAGCAGCTAGTGATCGATGAACGTCCTATACATATGCATCCGGCCTGCTACTACTT
GTTTATTAGAAGGAGATGAGGCAGACGAACAGACAAAAGTAATTAAGATTAGTGCAGATA
AAAGGAGTTCAACGCGTAATTGCTTATGTATGTAGAGAAAAAGATGGAAATGTAATAGTA
GTACTAACAAGTCATTTGTGTAAGAACTATACATATAGTGTTACCGGAATATATACAA
>ZmPG12
CGACCCAAGCAAGATTCACCATTCCGGGGCCTCCCATCATTTTGCCCGCAATCCAGAGTT
TAGCTCCTCCTCAGCAGCTTAATTGAACGGCCTCTAGTAATACATACAATGGAGTTCACC
GCCAGGACCGCCATTGCTCTGCTTCTCTCGCTCGCCGTCTCCTCCAGCTTCCTCTGTGGC
AGCGTCCAGGGCGGCCGCCACCACCACCACGGGAAGCACGCAAAGCATAGCTCCGCGCAC
CCGCCGTCCGCCCACGCTCCGGGCCCGGCGAGCGGGAGGCGGCACGCGCGGCCCGTCTCT
CCCCCGGCCCCGCCGCCGTCGTCTGGGTCGGGCGGCTACCCGACGCCCGGCGGCGCGGCG
CCGGAGCCAGCACCAGCCCCCGCGGCTGCAGCGGCGGGCGGCACCGTGTACGACGTCGTC
AAGGACTTCGGCGCCGTCGGGGACGGCGTGACGGACGACACCGGCGCCATCAAGACCGCG
TGGGACACGGCGTGCCAGGACGACGGGGCCAGCGTCGTGCTGGCCGCCGCCGGGTACTCG
TTCCTCGTGCACACCACCGTCTTCACCGGCCCGTGCCAGGGGAGCGTCACGATTCAGGTC
GGTAGCCGCCCGCCCGCCACCCGGCCCCGCTCCGCCGTGTGGAGATATTGAAAATGCTGA
CTTGATGATCCTCCTGACTTGCTGATCCCAATGGCGTTGTGTTGTGTCGTGTGTACGTGC
AGCTCGACGGGACGATCGTGGCGCCGAGCGACCCCGACATGTGGCCGGCCAACAGCAAGC
GCAACTGGCTGCTGTTCTATCAGGCCCACGGCATGTCGCTGCGAGGTGCCGGCCTCATCG
ACGGCAAGGGCCAGAAGTGGTGGGATCTCCCCTGCAAGTCTCACAAGGGCCAGGGAGGAT

CAAGCGGCCACGGTGCATCCTGTGACAGCCCAGTGGTAAGTGGCCTAACTAACGACTTTA
ACCGCATCATAACGGCCACTTGGGCCTGTCGTCAGCCAATGAAAGTTGTGGTTTAATCCA
CTGCCGTATCCATCACCAGGCGCTGAGGTTTTTCATGACCAACAACGTGACGGTGCAAGG
GCTCAAGGTGCAGAACAGCCCCGAGTTCCACATCCGGTTCGACAGCTGCCGCGGCGTGGT
GGCCAGCGGCCTCTCCATCAGCTCCCCGGCGCTGAGCCCCAACACGGACGGCATCCACGT
CGAGAACACCCAGGACGTCCTCATCACCAACACCGCCGTCTCCAACGGCGACGACTGCGT
CTCCATCGGCGCAGGCACCCTCAACATGCACGTCGAGAACGTCACCTGCGGGCCCGGCGG
CCACGGCATCAGCATTGGGAGCCTGGGCAAGCAGGGCTCGCGGGCGTGCGTGGCCAACGT
CACGGTGCGCAACGCCGTGATCCGCCACTCCGACAACGGCGTCCGGATCAAGACGTGGCA
GGGCGGCTCCGGCGCCGTCTCCTCCGTGTCCTTCCAGAACGTGCGCATGGACGCGGTGCG
GAACCCCATCATCATCGACCAGTACTACTGCCTCTCCAAGAGCTGCGAGAACGCCACCTC
GGCCGTCTTCGTCTCCGGCGTCTCCTACGCCGGCATCCGGGGCACCTACGACCCGCGCAC
CCCGCCCATCCACTTCGGGTGCAGCGACGCCGTGCCGTGCACCAACATCACGCTCTCCGA
CGTCGAGCTGCTCCCCGCGTCCGGCGAGACCATCGACGACCCCTTCTGCTGGAACGTGTA
CGGCAGCACCGCCACGCCTACGGTGCCGCCGGTGCCGTGCCTCATAGAAGGCGTGCCCAG
GAACCTCGACGATAACAGCAGCCTCAAATGCTACTAGCAGCCTCAAATGCTACCTTCCTG
CTACTTGTTCATTATATTAGAAGAAGGAAGAAGGCAAACCAACAGAGAAAAGTAATCAAG
ATTAGTGCAGATAAAACGGAGTTTAACGCGTGATTACTTATGTATATATAGAAAAAGATG
ATAGTACTAGTAACAAGTGATTTGTGTGACAATTATACATATAGTATTACCAGAATATAT
ACATGGTTTTCAACTTTAAT
>ZmPG45
ACAGTATATGTATGTGCCATACGCTGACTTAATCTGCTCCAAGCACCTAGCGCACACGTA
CGCTAGCTCCCCAGTCGATCAGCGGAGATGCGGAGCTCCGTCGCCTCGGCTGCGCTGCTG
GTGACCTGCCTGGTGGTGGCGGCGGCGGCGAGGTCGACGGGAAGGACAACGCTCAGCGTC
CTGTCGTTCGGAGCCGCGGGGAACGGGGTCACAGATGACGCGCAGGTACGTACTGCTATA
GCTAGAATTATATTGATGCATGTAAACTTGTGAACTGAACTGAACTGCCTTCATCCAGGC
ACTTGTAGCAGCATGGCAAGTGGCATGCCGAGTCCCCCGCGCCACCGTGCTAGTGCTCCC
ATCAGGGCACCGGTTCCTCCTCTCGCCGGTCACGCTGCAAGGCCCCTGCAGCGCAAAACT
CACCCTTCAGGTCCTTCTTCCTTCCTGTTCTGTTCCCCGTTCCATTTCGTCATAAAATTG
GCAAGTGCTAATTAGTAGCGGAAGGTTTTTTCCAGATCGACGGCACCGTGTTAGCGCCAC
CGGAAATGGGCTCCTGGCCGAAGCCCAGGAGGCCTCTGCAGTGGCTCAACTTCAAGTGGC
TGCAAGGCTTCAGCATTCAGGGAACCGGCGCGGTGGACGGTGGCCAGCAGAGTGCGTCAC
CAGCCAATGCTTCTCAGGTTCCGTTCCGTCGTCCACATGACATGCAGTTGTCTCCATTTC
TTCTATCTCTGCAGACCTAGACCTTGACATGAAGTGTTGTCTCTCCATCACTCACTTCTA
TCTGCAGACATCTACGGGTACCGGGCACTGGCATTCGCCAGGAGTCAAGCCTACGGTACG
TATGGCCACACGAACCAGCTTCATCGCTCGTTCGTACAGGAGTTTTACCTAGCTACTACT
AGATGGTGATCTGGATCCTAGCTAGGATTGTTAAACTAATAAAGCTACTACATTGTATGT
TTTTCTTCTGCCCTCTCCAGCTGATAAGGTTCTACAACAGCTTCAATGTCAGCGTGCGCA
ACATCCGGATCAGCAACAGCCCGCAGTGCCACCTCAAGTTCGACAGCTCCGGAGGCATCA
GGGTGAAGAACGTCACCATCTCCTCCCCCGGAGACAGCCCCAACACCGACGGCATCCATC
TTCAGAACACCAAGGACGTCGAGATCAGGAGCTCGACCATCGGCTGCGGTAATTAAAACT
CGACATTGCCCTGCATTTTGTTGTTGTTTTGGTTTGGTTTTAATCAGTCTTCCACATATA
TATTCAGGCGATGACTGCGTGTCGATACAGACCGGGTGCTCAAATGTCCACATGAAGAAC
GTGGTCTGCAATCCCGGCCACGGAATCAGGTGTCTGTGCAACTTAACGCAAGAGTTCAGT

TTAATGTTCCTTCCTTGGAATAATGTCTGTCCTGCAGTCTAGGAGGACTCGGGAAGGACA
ACAGCCTGGCCTGCGTCTCTGACGTTGTTGCAGAGAACATCGTCGTCCAGAACGCACTGT
ACGGGGTCAGGATCAAGACGTGGCAGGTAACGATGATATCCATATTATATATAAACACCA
CCTTGAGCTTGAGCAGCATGACCGGTTCATCCCATCCGCAGGGTGGCGTGGGCTCGGTGC
GGAACATCACCTTCTCCAACGTCAGGGTGGCGAACGTGGCCACGCCCATCGCCATCGACC
AGTTCTACTGCGACAGGGGCGGCGCGCGGTGCGCCAACCGCACCGGCGCGGTGGCCATCG
CCGGCGTGGCGTACCGCCGCGTGGTCGGCACCTACACGTTCCAGCCGCTGCGCCTGGCGT
GCAGCGACGCCCGGCCCTGCACCGGCGTCACCATGCTGGACGTGCGCCTGTCCCCGGCGG
CGGCCGGGGGCACCGTGGCGCCGCTGTGCTGGAACTCCTACGGCGAGGCGCGGGGCACAA
TCATCCAGCCGCTCAGCGTCGGCTGCCTGCAGAGGAGCAACGGATACGCGATGCCTCTCA
CCCAGCCTTTTAACTACACATGTTGAGTAAACTACAACAACCAAGGACAAGGATTGTTAT
AAGTTCGAAAGCTTACTCTGTCTAGCTCTATTACTCTTTTTTTTTCTTCTTCTTCTGCGT
ACCTCAATTAATTCTGGACTAATTAGACTTCATCAATGTTCTGAATGACTTTTAAATCCA
TCTAGCTTCCTTGAGCCTTTCTTGCAACTTCTTGGGTTCCAATTCCATAGACTGAGATCA
GGCCTTGATTATATATTATTTTCATGCAGAGGTATGTTGGGTATAATAATGCTTTCAAAA
GCATTCAAAGTTCCTCTACCAATGATTACATTGTAAGGCTACTCCATATTCACAGTATCA
AATGTGATTTCTTCGGTTTTAGTGTTATGAACATAATCGAAGGTTACTGGCATTACTATT
TTTCCTAGTGTTACTATTTGTTTTCCTTCGAAGCCAC
>ZmPG53
ATGGGGCAGAGCATCCAAAACGTCTGCTTAGCTAATAACGCCACCTTCCTCAGCGCCCTG
CTTCTACTCGCCGTTACCACTTCCGGCGGCCCGTCCTTGCAATGTTGCCTCGCGAGAAGT
GAAACCCACCGGCGTCCACCACCGACGCCACCGGACGGCCCACACCATCCTCCGGCAGCT
CCGGTCATGACGCCGCCGCCGTCCCCGTCAGCGACCTTCAGCGTGGTGGACTACGGTGCC
GTGGGGGACGGCGTCACGGACGACACCAAGGCGAGTGCTGATATAATACGTTAGTAGTGG
TGGACTATCGAAAGTTATTATTATTTTGATCGAGTTCGTATGCATGATATATATATATAT
ATATGTAACATATATGCCGTGTGTCTCCTTTTCCTTCGAAAATGCAGGCCTTCGCCGACG
CCTGGTCGGCGGCGTGCGCAGCCGAGGCGTCGACGGTTCTGGTGCCAGCGAGCTACGTGT
TTCTAGTCGGACCCATCGCATTCACCGGCGGTGATTCTTGCGAGCCCAACGTCGTCTTTC
AGGTGGGTCCCCGTCCGGATGACTAGGAGTGCAAAAAAATGAAGCAATCACACATGCCAT
GCCGTGGTTATCGACTCTGCTACATGTTTATTTTTTTTTCCCTTAAGGTAGACGGCACGA
TCCTGGCGAACACAGGCTCCACGGCATGGCGCTCCGGCTACGCGACGCAGTGGCTTGAGT
TCAAGAGCGTCAGGGGCCTCACCATCCAAGGCTGCGGCGTCATCGACGGGCAAGGAAGCC
ACTGGTGGAGCCGCAATCCTCTCGTCGACCAAGGCCAGACGACGACGGTACGGTAACCTC
TTGTCGTTATTACTATACATTTGCTGGATTTTCAGCTTATTGTTGTTGGTTTATGCAGGA
GCTGGACCCTGATCGTGTGGGAACAATCGACCGACCTACAGTTAAGCACGACAATAAACA
TGATCTGATTTCTCTTTCTGGAGCAGCATTAATTATATCGATCTTAAAACCCTAAACCCT
CAGGCGTTGAGGGTGTACCAGGGCGACAACGTGACCGTGACGGGTATAACCATCAGGAGC
AGCCCAAAGTTCCACCTCACTCTGGACACCTGCCGCGCGGTGGAGGTGCACGGCGTCACC
ATCGCGTCGCCCGGCGACAGCCCCAACACCGACGGCATCCACCTCGCCTCCTCCGTCGGC
GTCTCCATCCACCACACCACCATCGCCTGCGGTAATTAATAAGGGGATAGATAGATAGCA
GTGGAAAAACCAATCCATTCACAAATGAAACCAACTGGTCTTGCGATTGATTCGCAGGTG
ACGACTGCGTGTCGATACAGGCCGGCTGCTCCGACGTGTCCATCCGCAATGTGTACTGCG
GGCCGGGTCACGGGATCAGCGTCGGCGGGCTCGGCAAGGGCGGCGCCACGGCCACCGTCT
CCGACGTCACCGTACAGGACGTCACGTTCAACCACACGATGACCGGCGTCAGAATAAAGA

CCTGGCAGGTAAGCCAAGCCAAAGGCATGCATGCGCCACGCCACCCTAGCTAGCTTGCGG
AGGAGGCCCAACCGGAGCGGAGCCTAACACAATCAAAGATACATACGTACATACAGGGCG
GGTCTGGGTCGGTGAAGAACGTGCGCTTCTCCGGCGTGCGGGTGTCGGCGGTGAAGACGC
CCATCGTCATCGACCAGTACTACTGCGACCACACGGCCTGCACCAACCAGACGTCGGCGG
TGGCCGTGGTGGGCGCGGCATACCAGGGCGTCGCCGGAACGTACACCCAGCGCCCGGTGT
ACCTGGCGTGCAGCGACGCCGCGCCCTGCTCGGGGATCCACCTGGCCGACATCCAGCTCC
AGCCCGTCAAGGACAGCGGCTACCGCGTCCAGGGCCCCTTTTGCTGGAAGGCGCACGGCG
ACGAGATGCGCCCCGTCGAGCCACCCGTGGACTGCCTAATAACCGCCGGAGCTCCATGA
>ZmPG47
AGTGGGGAGCGACGAAAGCCATGACACAAGTGAAGCTACTCTCGTCTCGTGTCTCGTTGC
CGTTATTTAAACACCCCTTATCTTTCCCTCCTCAGGCTCAAAAGCCTTGCCCTTCCCTGT
TCTGCTCCTCCTCGTTCTCTGTGATGCTGAGGCTAAAAGCTCTCACCTTCCTGCTCCTGC
TCCTCCTCCTCGCTCTCTGCTCCGGCGTTCATCTGTGCGACGCGAGGAGCGGCACGCGCT
GGAGTCGGACGACGCGAGGCGGTGGTAGCGGGGCGGCACCGTCCGCCTCCGCTGCTGCGA
TCGGGAGGAAAGGGAAAGCGAGAAGCAGCGGCTCCTCCCACAGGCAAAGCGGCGAAGGGA
GTCGGAGCCCGACCGACGGCGACCCCGTGCAGGGCGACGGAAGCCAGGCTCCACCACCAC
AGGATACTACGGTCTTCAGCGTGGTGGAGTTCGGAGCCAAGGGTGACGGCGTTACAGATG
ACACTCAGGTCATCTGCTTGCCTTTCCATCCTTGTGATTTGCAAAGACATGTCATGCCTC
AAACTCCGTTTATAGTCTAGATACATTGATTCTATGTTTTCCTTGCATTCTCAAGGATAT
AGATATTTAGACCAAGGAATTACAAGCGTCTTGTGCAGATCAGATGCGCTTATACACGGG
CTCAAAATGTCTGTTAAACTGAATAACCGGCAGGCCTTTGAAGCAGCGTGGGCAGCAGCG
TGCAAGGTGGAGGCGTCTACGGTTCTTGTACCATCTGGGCTCGAGTTTGTCGTGGGCCCG
ATCTCGTTCTCTGGGCCTTACTGTAAACCGAACATCCTGTTCCAGGTAAAAAAAATAATA
TCTACTTCTTCCAACTCTATTGTAATGCGTGTCGTTACATGGGCAGAGCACAAGGCATGC
TGGCTAGATCCTCGTTAGGTCTGTTTGGTTTATAGAGACTAATTTTTAGTTCTTTTATTT
TATAGTCTCTAAATTGTCAAATATAAAAATTAAAATTAACTTTTTTGTCAACTACGGCAT
CGGTAGCAATCTGTTTTTAGTGTGTGTTCTATTAAACACCATGATACCAACTGCAGAAAA
ATGGATGAATATTTGTTTGACACACACATACACGAAAAAATAATAATAATTCCTGGTACT
GTTGTGGCCTGCCAGCAAACAATGCAACAAAAGATATAGTGCATGTTTGGTTTGTGGCTA
ACACTTTGCCTAACATTAGTCCTTCGAATTGAAAAACTAACTTTAGATAGAAAAGTTAGA
CAAAGTATGGTAAATTAGACAGCGAACCAAACAAAGCCTATAATCAAACTGGAACTGTTC
TGCAGCTAGATGGAACTATTTTGGCCCAAACCAGCGCAAGAGCATGGGGCTCTGCTGGTC
TGTTGCAGTGGCTGGAGTTCACGAAGCTAACCGGAATCGCGGTCCAAGGCAGCGGTGTGA
TAAACGGCAGGGGCCAGGACTGGTGGACGTATGCAGACCCAAACGATGCCGACGACGAGT
CTGTGGAGATGCTGCTGCCACGGACTAAGCCCACGGTAAGCATGCATTGCAGCCTACACA
TCACTGCAGCACTGCTTTGTTGACACATGCATGCATCTCCGTGATTGCTTGTTTTTAAAA
AAAACATGGCTGCAGGCGCTGAGGTTCTATGGCAGCTCCAATGTCACGGTAGCTGGGATC
ACGATCGTGAACAGCTCCCAGTGCCACCTCAAGTTCGACAGCTGCGAGGGCGTCATGGTC
CACGACCTGAGCGTCTCGTCCCCCGAGGACAGTCCCAACACCGACGGGATACACCTGCAG
AACTCCAGGGGAGTCGACATCCACCACGCGACACTGGCCTGCGGTAAAATGCTACTGTAG
CTGGTTGTAGTAGTAAAACGGACCCTGCTGGCTGCTGCTACTGAGACACCTGCTTCATAC
GTGCAGGCGATGACTGCATCTCCATACAGACGGGGTGCAGCGACGTGAACATACACAGCG
TGAACTGTGGGCCAGGGCACGGGATCAGCATCGGTGGTCTAGGGAGGTTCAACACGAAGG
CGTGCGTGTCCAACGTCACCGTACGAGATGTCAAGATGTTCAGGACAACGACGGGTGTCA

GAATCAAGACATGGCAGGTACGCCGCCCGTACGTCAGAATAATGCAGTAGCAGGCAGCAG
CAGCAGGCCTAATAATTACTAATAAACCTTTGCAGGGCGGGTCGGGGCTGGTGCAGGGCG
TGAGGTTCTCGAACATCCAGGTCACGGAGGTGCAGACGCCCATCATGATAGACCAGTTCT
ACTGCGACAGGGCGACGTGCAGGAACCAGACGTCGGCGGTGGCCGTCTCGGGCGTCCAGT
ACGAGGACGTCAGGGGCACCTTCACCGTCAGGCCCGCGCACCTGGCGTGCAGCGACAGCT
CGCCCTGCTCGGGGATCACCCTCGCCGGGATACAGCTCCAGCCCCTGCCGCTGCCGCAGC
ACCGCCGCCTGTCGTACGACCCCTTCTGCTGGCAGGCGTTCGGGGAGCTGTACACCCCCA
CCGTGCCCCCCATACCTTGCTTGCAGATCGGCAGGCCGGCAGCAGGGAACAGCGTGCTGT
CCGACGGCGACCTTTGCTGAAACGCCGATCGGTTCATCAGTTAAGCTAAGGCGGTCGTCG
TGATGCTTCCGACCCCTGCTTTGCACGATGTGGATTGTGGAGTGTCAGTTGATGATGCAT
CATGCATGCATGCGTACGTGGGATACTTGAGGCCGATTTATTATATTGATGCGAGCCGAT
CACCACTATTGCTATGTACACGAGGTCACTTGTCACGCACGCTCCAGTCGTTGTCCAGCA
TCA
>ZmPG43
ACAATCACAGGCCTCCCGGCCTCCCGTGCGCTATTCTCTATCAGCTTACCCAGCACACAG
TACACACTCTAATCTCCACACGACCCAATAGATATACGGGGCTGAAGGATGAAGCTTAGA
GCAAAAGGGATCGGCCTCCTGCTTCTCCTTGTCTTGCTTGCTCTTTGCTCCACCATTGAA
GTCGGCGAGGCAAGAAGAGGCAAGCATTGGAGGTCGTCGAGGAGCTCACCGGGCTCATCC
CAGCTCAAGAAAGGCAAGGGAAAGAAGAGCAGCTCCCACCGGCAGTACGGCGCCAACCGG
CCAGGTCCGAAGCCACCGGTCAGCTCGACACCAAGCTCTGGCGCTGGCAAGGGAAATCAT
CAGAATCCGTACCAACCAAGCCCAAGTCCAAGCCCGAATGCCCCTGACATCCCTCCTATG
CCAAGCCCTGCCAATGGCAGCAGGCATTCCACTCCCGAGCCGCCGGCTCCAAGCTGTGGG
AAGGGTCAGCAGCAGCCATCACAGCCGCCGCCAGCGACCTCGCAGGGTGCAGTATTCAAT
GTGGTTGATTTTGGAGCCAAGGGTGACGGAGTTACAGACGATACTAAGGTACATAGCTTC
GCCCTGCAAATGCTCTGCTTCAAACAAAACTCCATTATAAAATCGTCAAGCATCATGCAT
TCTTTGAGCCAGAAATCTCAAATTGCAATCCCTTTTGTGGGTTTGGGCTTCACATGCTCT
GTTTCAAACTGATCTTTACATGACTTGCATGAGAACTCAGTGATGTCAATCACATCCATC
CAGGGGACCTGTCGTCCCAATGACATCTTGAGATTCCAATTGGCCATGCGTATTTTTCTC
TCTCTCTCTCTCTCCAGAATTCTTAGAAACTGGAATGCACCATGAATTCATTTGCCAGAA
CCACCACAGCTTATGATAGTGAGCGTTTGAAGGTGCCTATGACCATACCAGTTTTACTCT
CACTTTCTCACATGATTTGCAGTAGAAAGCCCGAAAAGTTGTCACGGGCACCATACGTTT
CTGCAGTCTTATTTGCATTTGTTTCGATCTGTTCCCTGCTTTGTCTTGTTCTTTCCGTCT
CACATGATTGTGGCATATAGCGGCATAGACACACCAGCCTGGCCAAGTTTATTTTTTATT
TTTTAAGCCATGGAAGAGAACAAAGTACACCAAGCAAGTCAGAATACACATAATGCAGAA
CCCTTTCCACAGCTCTTACAAGACGTTTCTGTGTACAGAGAAAACAAACAAACCCAATTG
TAATATTGTGATTCTCAGGCTTTACAGATAAGAGAATAGCGAACTCCCTCTCTATGACAT
TAACAGAGCACAACTTTCTTTCTTTTGCAACAGCACACTGCATGCACCAATAACACCATA
TTTTTTTGTTCTTACAGCAAAGCGAAATAGAAAGTTCATGGAAGTTTTGTACCAGGTTAA
CAAGAAATAAGCCATACTTAACGATATTTTCCAGTATGCGTTGTCTCATTATCTGAAAGC
TCTATCAATCCTATAGATTTTTTAATATCTCAAGTAAATTAACTTCGTTTTTTTCAAATC
CTTCGTGTGTGGTGGGGGTGGGGTGGGGGGTCACAAACTGATTCTTGTTTTGGCACAGCG
ACCAAAAAGATATAAGACAAGAGTGCAACAGCTGATTACCCTGCTTAGGGGTCTGGACTC
TCAGTTATTTTTTGTGATGCTAGATTCCAGGGATTGTCTTTTCAGAAATCTAGAGCTAAT
AATTGTACGTGCATTTGCATACAGGCTTTTGAAGGAGCGTGGGCTGCTGCCTGCAAAAAG

GGGGCATGTACAGTTCTCGTACCACCAGAACTAGAGTTCCTAGTTGGGCCAATCTCGTTC
TCTGGGCCTTACTGCAAATCGAATATTGTCTTTCAGGTAAGCATGTTCTTTATCCCAAAT
GATAAGATACTTAAAAGGGTTTTCTCAACATAGCTTGAATTGAGCAACAAAAGGCAGGAT
GGGATTGTATTTTATCTAATTTGAGATGCACACCAATGGAAGAATAAGTTCGTTGAGTAA
TGCTGTTTAATGTTGTACTCACTTTGCAGCTGGAAGGAACAATCCTAGCTCCAACCAGTG
CTAAATCCTGGGGTTCAGGCTTGCTCCAGTGGCTCGAGTTCACCAAACTAAATGGAATGG
TTATTCAAGGGAACGGCATTATAAATGGCAGAGGGCAACAATGGTGGACCTACTCAGACA
CAGAAGACGAGGATGATGATGACACGGTGAGAGCTGGCCAGCAAGTGTATAATTTTTTAT
TAAGTACTCAAAATAAAAGATAGTCATGTTAAGTGCTTACTTTGGATGCTTTCCTGCAGT
ACGATGTGGAGTTTGAAAGAATGCCACAGATTAAACCTACAGTAAGAATTACTATTTTTA
TGCACACTATTATCATACCAAACTGAAATGAAGTAATGACAACACTGATTTCATACCTGA
TGCAGGCATTGAGGTTTTATGGTAGTTTCAACGTTGTAGTGGCTGGTATCACTATTGTCA
ACAGCTCACAGTGTCATCTTAAGTTTGACAACTGTCAAGGAGTGATGGTCCATGACTTGA
CTATATCCTCCCCTGAGAACAGTCTCAACACTGACGGAATACACCTGCAGAACTCCAAAG
ATGTCAGCATTCATCATACAAACCTGGCTTGTGGTAATTCCTTAATCCTTACTGTATGTC
TAAGGGGGTGTTTGAATGCACTAGAGCTAATATTTAGTGGCTAAAATTAGTTGAGACATC
CAAACATCATAGCTAATAATTCAACTATTAGCTATTTTTGGTAAATTGGTTAATAGTTGG
GTAGTTAATTGTTAGCTAGATAATTCCACTAACAATTTTTAACCAACTAACTATTATTTC
TAGTGCATTCAAACACTAACTATTAGCTCAAAAGTTTGTTTCGCAAAGCTTTACAACAAA
GACCCATTTCCATAAAGAACACCAAATAAATAAGACATTATCCACACAAAAAATTACAAA
TAAATATGTAGGGTCAACCAAAAGCCTGCATCTCAGTTCTGTTACGCATGTATACTCTGC
ACTTCAAAGGACATGTTTCAGAGTTAAGACTACACTGAAAAACTGCTGTGGAGTATCATA
ACATCGTTTTGTCCTATTCGATGCAGGTGACGATTGCATCTCCATCCAGACAGGATGCAG
CAACATAAATATACATAACGTGAATTGTGGACCAGGCCATGGAATCAGCATAGGTGGACT
AGGCCGAGACAACACAAAAGCATGCGTATCAAATGTTACAGTAAGAGATGTCAACATGTT
CAGAACCATGAATGGTGTCAGAATCAAGACCTGGCAGGTAAGCTTGAGAACATAAGTACA
AATAAAATTCTAATTCTAATTCAACATCCGGCATTAGTACATAATAGTTAATAATGTCAC
AAAAGAAAATCTGAAAAAGGAAAGAAAACGTACATGTCATCGTTTGCTATCTATACAAGC
AATAACCTTGAAAAGTTCAAAGAAGAAAAATGATGTCACCCAAAACAATTCCTAGTGTCT
AAACAAAGTGAACTCTCATTTCTCGCAGGGTGGTGTAGGATTGGTTCAAGACATAAGGTT
CTCAAATATACAAGTCTCAGAGGTTCAAACACCCATCATCATAGATCAGTTTTATTGTGA
CAGAAGCACTTGCAGAAATCAAACATCAGCAGTGGCAGTATCAGGGGTTCAATATGAGAA
TATCAGAGGAACATTTACAATCAAACCTGTCCATTTTGCATGCAGTGACAGCTTACCTTG
TTCAGGAATCTCTCTTACCGGTGTGCAACTCAGACCAGTGCAAGTACCCCACTACCACCT
GAATAACCCATTCTGCTGGCAAGCTTTTGGGGAGCTCTACACTCCAACTGTCCCTCCTAT
AGCTTGCTTGCAGATTGGAAAACCTGCTGGCAATAACTTACAGTCATATGATGACATATG
CTGAAACACTCAAAGTGCCTTTGTTTGTTGATTGGTGCTTATCCATGAGTGGTCGTTGTG
ATAGATGCGACCCCATTTGCACCATTGCTTGCTGATGAGCACATATACAGTTATTTTGAG
GTCATCTTATTACAACATTGTAGCTTTTTTCATCAAAGTAAACATAAATATATTCATTTG
AAATGGTCAT
>ZmPG8
CCCGCAACCACAGGCCTCCCGCGCTCTATTCTCTCTATCTACCACTACTACTACCCAGCA
GCAGCAGGCCAGCACACAATACACACACTCTAATCTCTCCACACAGCCAACACAGATACG
GACAGGACAGGAGGATGAAGCTGGGAGCAAAAGGCCTCGGCCTCCTGCTCCTCCTTGTCC

TGCTTGCGCTTTGCTCCACCATTGAAGTCGGCGAGGCAAGAAGAGACCGGCATTGGAGGT
CGTCGAGGACCTCCTCCTCTCAGCTGCTGAAGAAAGGCAAAGGAAAGAAGACCAGCTCCC
GCCGCCAATACGGCAGCAACCGGCCAAGTCCGAAGCCACCGGCCAGCTCGACACCGAGCT
CTGGTGCCGGCGGCAAAGGAAACCAGACTCCGTACCAACCAAGCCCGAATGCCCCTCACA
TCCCGAGGCCAAGCCCCCCTGCCAATGGCAGCGCCCATTCCACTCCCAAGCCGCCGACTC
CTCCAAGCTGCGGGAAGGCTCATCAGCAGCCATCACAGTCACAGCCGCCACCACCAGCAA
CCTCCTCGCAGCATGGTGCAGTATTCAATGTGGTTGATTTTGGAGCCAAGGGTGACGGAG
TTACAGACGATACTAAGGTCCGATAGCTTCGCTGCTCTTTTTTTCAAACAAAATTCCATT
ATAAATTTGCCAAGCGTCATGCGTTGTCTGATCCAGAAATCTCGAATTGCAATTCCTTCC
GTGGGTTTGCGCTTCAGATGCTCTGTTTTTTCGCCCTGATCTTTACATGACTTGCATGAG
GACTCAGTGATGTCGATCACCGTGGCATCTTGAGATTCGAATTTGTCGTGCATATTTTCT
TTTTGGAATGCTTAGAAATCGGAAATTAGCAATAAATGCTAACCTGAATTCATTTGCCAG
AACCACCACAGCTTATGATGGTGAGCATTTCAATGTGCCTATGCCCATACCTGTTTTACT
CTCTCACTGTCTCACATGATTTGCAGTACAGAGCCTGAAAAGTTGTCACCAGCGCCATAT
GTTTGCATGTGTTTCCATCTGCTCCCTGCTTTGGCTTCTTCTTTCCTTCTCACATGATTG
TTGCACATAGCGGCATAAACAGATCCATTTACATATCTAGCTGGCCAGGTTTATTTTATT
TATTTATTTTGTAAATCAGGAAGAGAACAAAGTACACCAACCAAGTCAGGATACACAAAC
ACAAATGCATAATCTTTTCCATAGCTCTTGCAAGATGTTTCTTTCAGAAATCTAGAGCCA
ATAATTGTACGTCCATTTTGCATACAGGCTTTTCAAGGAGCGTGGGCTGCTGCCTGCACC
CAGGGGGCATCTACACTTCTCGTACCACCAGAACTAGAGTTCCTTGTTGGGCCAATCTCG
TTCTCTGGGCCTTACTGCAAACCGAATATTGTCTTTCAGGTAATCCTACTCGTAATCCCA
AATGCTAAGATACACAAAAGGGCCTTTTCAACGTAGCTTGAATTGACCAACAAAGTAGGA
CAATGGAGTAGTAATGTTGTACTCATTTTGCAGTTGGAAGGAACAATCCTAGCTCCAATC
AGTGCTAAATCCTGGGGTTCTGGCTTGCTCCAGTGGCTCGAGTTCACCAAACTAAATGGG
ATAGTTATTCAAGGGAACGGCATTATAAATGGCAGAGGGCAACAATGGTGGACCTACTCA
GACCCAGAGGATGAGGATGATGATGACACGGTGAGAGCTGGCCACCAGATGTATAATTTT
TTTATTAAGTACTCAAATGAAAAAGTTATTCATGTTAAGTGCTTACTTTGGATGCTTTCC
TGCAGTACCATGTAGAGTTTCAAAGAATGCCACAGATTAAACCTACAGTAAGGATCACTA
TCTTTATGCACACTATTATCATACATACCATACTGAAATGAAGTAATGGCAACCCTGATT
TCATACCTGATGCAGGCGTTGAGGTTTTATGGTAGTTTCAACGTTGTAGTGTCTGGTATC
ACTATTGTCAACAGCTCACAGTGCCATCTTAAGTTCGACAACTGTCAAGGAGTGATGGTC
CATGATGTGACCATATCCTCCCCTGAGAACAGTCTCAACACTGACGGAATACACCTGCAG
AACTCCAAAGATGTCAGCATTCATCATACAACCCTGGCTTGTGGTAATTCCTCAATCCTT
ACTGTATATCTCAATTCAAGAGGGATCTAGGCTGTATTCATGAAACTCAAAAGTTTTGTT
TTGCAAAGCTTTACAACAAAGACACATTTCCATCAAGAACACTAAACAGATAAGACATTA
TCTACAAAAAAAATATTTACAAGGAATTATCTAGGGTCAACCAGAAGTCTGCATGTCAGT
TCTGTTACACCTGTAAACCCTGCATGTCAAAGGACATGTTTCAGAGTTAAGAGTACACTG
AAAAACTGCTGTAAACTAGCATAACATTCCTTTGTCCTATTCGATGCAGGTGATGATTGC
ATCTCCATCCAGACAGGATGCAGCAACATAAATATACACAACGTGAATTGTGGACCAGGC
CATGGAATCAGCATAGGTGGACTAGGCCGAGACAACACAAAAGCATGTGTATCAAATGTT
ACAGTAAGAGATGTCAACATGTTCAGAACCATGAATGGTGTCAGAATCAAGACCTGGCAG
GTAAGCTTGAGAACATATGTACAAACAAAATTCTAATTCAACATCAGACATTAGTACAGA
ATAATGTCACAAAAGAAAAAAAAAACTAAAAAAAAGGAATGGATATGTATATGTTATCAA
TTTGATATCTATACAACCAATAACCTTGGAAAGTCCCAAGAAGGAAAGTGATGATGGCAG

CCAAAACAACAATTCCTACTGAGCTAAACAAAGTAAACTCTCATTTCTCGCAGGGTGGCG
TAGGATTGGTTCAAGACATAAGGTTCTCAAATATACAAGTCTCAGAGGTTCAAACACCCA
TTATCATAGATCAGTTTTATTGTGACAGAAGCACTTGCAGGAATCAAACATCAGCAGTGG
CAGTATCAGGGGTTCAATACGAGAATATCAGAGGAACATTTACAATCAAGCCTGTCCATT
TTGCATGCAGTGACAGCTTACCTTGTTCAGGAATCTCTCTTACTGGTGTGCAACTCAGGC
CAGTGCAAGTACCCCACTACCACCTGAATAACCCATTCTGCTGGCAAGCTTTTGGGGAGC
TCTACACTCCAACCGTCCCTCCCATAGCTTGCTTGCAGATTGGAAAACCTGCTGGCAATA
ACTTACAGTCATATGACGACATATGTTGAGAAACTGAAAGTGCCTTTGTCTGTTCATTGG
TGCTTATCCATGAGTGGTCGTTGTGATACATGCGACCCCATTTGCACCATTGCTTGTTGA
TGTGCACATATACAGTTATTTTGAGGTCATCATATCACAACATTTTAATCTTTTCATCAA
AGTAAACATAAATATATTCATATGAAATGGTCATACAGCAAGAGTATATGTACTTGAGTT
CTCCGAAAATGAGTAAACATAATGACGAAGGTTATTTGCTTAAATAAGTGTAAACAGCGC
TACAGCTAAGGGCCCAAAGATAATTGATGGACAACAGTAGTAACCCGTGGCCGTACACCT
ACAACTATATTTATCTATATTGATACCTAATCCTGAAGAGATTAATAATTAGAAGTCGAT
GCCTACGGATATTTAGAAAGCAATAGTTTACCGACCAGAAAAATCTCTTGGTTCAAATAC
GAAAAGGAAAGGATTGCTAAAAAAGACTAGGCAAAAAAAATTAGTAACCGCCCTCCCAAT
ATTATATTAAGAAGAGACCGAAATAATGATCTCGACCGAGAAACTTCCTGAACCTTGGCC
CCATCACTAACGGGCCGGCGTCAACTATCCAGTCTGCAATGGTCCAGCCGGAGGGTGGTG
TTGGAGACCCTCGTACTGTTCACGCTGGATTGTAACCGCATTCACGTCTTTACCTTCAAG
CAAACCGAAAATATCAATGTAATGTGTCTTGTTATTATGTATACATTCAATACGAAATGA
ATAAATGATATGATATTACATTTGTAATTATGTTTTTATATTTGTGAATAATGAAGACTT
ATTCTT
>ZmPG40
ATCCGTGTCCGTGCGATTTTTCTACACAATCGTCTCGCAAAGATTCTCGTTGGTCAGTTT
GAGACTTAGCCTTGGAGGAGAACAGAACAGCCACGATGACTTCGCCGATTCAGCTCGCGG
CCGCGCCTTTGCTGCTGCTAGTAGTAGTGGTCGCGGCACACGGTCGAGCCGCATCTTTT
CGGTGGCCGACTATGGTGCGGCGGGCGATGGGTTCCGCTACGACACGGCGGCGATTCAGG
CCGCTGTAGACGCCTGCGCGGCGGCGGGCGGGGGCCGCGTTCTTCTCCCGGCGCCGGGCA
ACTACCTGACAGCGACCGTCCAACTCCGCTCGCGGGTGGTGCTGGAGGTGCCCCCCGGAG
CGCGGCTTCTGGGCGGGACCAGGCAGGCGGACTACCCGCCCGAGTCGCGCCGGTGGTACG
TCGTTCTGGCCGAGAACACCACGGGCGCCGGCATCACGGGGGGAGGCGAGATCAACGGCC
AGGGCGGGGCATTTGTTGTCAGGCCCAGCGAGGTGAAGAACGTCATGGTGAGCTGGAATG
CCACCGGCGATTGCCTGGGCGATGAGTGCCGGCCGCGGCTTGTTGGCTTCATTGACTCTA
AGGATGTTAGGATCCACGACATCACACTCAACCAGCCCGCCTACTGGTGGTAAGCATTCC
TTGCTTCATCAACTTCATCGATCGATTCAGGGAAAAAAAATTGATGGCTGCTATTTTCAC
TACTAAAACCATAGTTGTAGACAGGAACAAACAAACCTGCCTGATTATTCCATTCCAACG
ATGCTAGAATCAACAACCTTTTCTCTCTACCATTTTTGTTCTTGCGGTTGTGTGAAATAA
CTTGTCCTGATATGTTTTCCTACTTATTAGGACATATGATGATCAGAAGTAGACAGTGTT
TATTTGTCAGTGTACTTTGGCAAAATTAACAGGCTCTACACTTGCATTGCTCGCAGTCTT
CACCTTGTCAGATGTGACAACACAGTCATCCACAATGTGTCGATATTTGGAGATTTTAAA
ACTCCCAACAATGATGGCATTGACATTGAGGATTCTAACAACACTGTCATAACTGATTGC
CACATAGACACCGGAGACGATGCTATAAGCCCAAAGTCGACAACTGGACCAGTTTATAAC
TTGACGGCAACAAACTGTTGGATGCGGACTAAATCTTGTGCTATCAAGTTTGGAAGTGCA
AGCTTCTTCGACTTCAAAAGACTGGTTTTTGACAACATTACAATAGTTGATTCACATCGA

GGACTTGGGATGCAGATCCGGGATGGAGGTACTAACCTGCAACCTACGATTGTCAGTAAA
GCAAATATCTGTTGAATGTGAATGACACACTAACAGTTTACCTTTTACAGGGAATGTGAA
TGATGTGGTGTTTTCAAACATTAAAATGAGGACCAGGTACTACCATCCTTTATGGTGGGG
AAGAGCCGAACCAATCTATATCACAACTTGCCCAAGGCACCCAGATTCAAAAGAAGGCGC
CATTTCAGACATAAGATTCATCAACATCTCATCGGTATCAGAAAATGGAGTTTTTCTGGC
TGGATCTAAGCATGGGCTGCTCCGCAACCTGAAATTCAAGAACATAGACCTTACCTACAA
GCGTTGGACAAACTACACGGGAGGGCTCTATGATTACAGGCCTGGGTGCCAGGAGATGGT
GAAGCACAAGACTGGTGGCATGATGCTGGAGCATATCTCCGGTCTGGAGATTGACAACGT
GAGGATGAGATGGGCGAGAGGAAGCCTGAAGGGCTGGAATGTTAACCCTATCCTCTTCCG
GCCATCAACCATCGATAATCTGTCTTTCCATGACTGGCAGTCAGTGGATGTTCAGTAGAT
TTAAGTGGCACAGTTGTATTACGTGTGTATCTATCAGAATAAAGGGGTGATTCTTTTGTG
AAATATTGAAATGTGTTAATAATAAGCAATCCCAACAGCATTTAAGCTTCTGC
>ZmPG41
GCGGAGCCCACAGACCACAGTCACCGAGCGCCAAGAAGAACGCTCTGCCCGGCCCGCCGT
GGCGAGTCCATCCAACGGCCCCGAGAAGCTGAACTGACCGGCCACCGCCCAGGGTCAGCT
CGGTCATTCCGCCGCCGCTGTCGTGGTCGTGGTTCCGCGCCAACTCAACTCCACGGCCGC
GGCGCTAGCATCCACTCACCATCGTCACTCGCCGCCGCTGGCACCACCCTCCACGCCCCG
TCCGCACTTTTTAAAACTACGACCCAGCTGCCGAATCAACCGCCTCCCCCGGCGAGCGCC
TCCTTCCTCTCTCCCATGGGCCCCCGCGCCCGAGCCGGGACCGCTTCGCCCACCGGCCGG
TGCGCGCGCCATAGATAGATTCCACAAGCTTCGCGACCAGATCCGATGCTGGAGGCCACC
GCCGCCGCCTCCTCCCCCCGCGCCGCGGCCGCGGCAGTCGCCGCCGGGGCGGCCGCCGCC
GCGGTCGTTTCCTCCCCGCGGCGGGGTGGAGGCTCCGCGCACAACCACCGGCGGTGGGCC
CCGGCCCCGTTCAGGGCTTGCCTGCTCGCGCTCTGGTTGCTGGGCTTCGCGCTCGTCTTC
CTGTGGCAGAGCACCTCCGTGGGCCGCGTGCGGCTCTACACGCGCCCGCCGATGCCGAAG
CGCGCCGTGGCGTCGTTGGGGCACTGGGCGGCCTCCCCGCCCGTCTACGATCTGAGGGAG
TTCGGGGCGGTCGGGGACGGGCGGACGGTTAACACGGCCGCGTTCGAGTCTGCCATCGCG
GCGATCGCCGAGAGGGGCGGGGGCAGGCTCACCGTGCCAGCCGGGCGGTGGCTCACCGCG
CCGTTCAACCTCACCAGCCACATGACTCTCTTCCTCGCTGCTGGCGCTGAGATCCTAGGG
ATTCAGGTGAGACATTAGCTGCTATTTGCATCAGCTTTGTTTGGCTAAAGACGCTGCTCT
CGGGTCTTTAGTCAATCTGGATTCGTTTGTGCAAGTATCTACTCTGGGCAAGTGAGAAGC
TGAAAAAATTCCTGCTATTGTAGTATTCAATCATATTAAGTCATGAACATTCATAAGTTT
TGCATGAAACGATGAGTTCATCATCAATCCAGGGAGTAATGCAAATACTAGGCAGTTTAA
TAATATGTATCAATAATTACCAGTATTGCTGAGCAGGACAGGCAAATGCCCTTTACACAA
ATAGTTTTTTTTTAAAATTGTATCAGAAATTCCTGCTAAGTTTATCACAAAAAAAAACTGT
CTAGGAGTACCATTTTCAACTACACTGCAAGATTTGTGGAAGAATGAAATTTGATTGAGC
AAGGACTTACTTGAACACTGAAGTCATCAGCATGTCAAAGTTAGTTTGATAGGTTCCAAG
GATCGTAGTAGTTAGTCCCAACATAATGTTTTGTTGTTTTAGGTCAGTTCAGAATTGGTT
TCAAATAAATTTGGTCACTGCCTTATGCAATAGTCTGGTTCAAGTTAAGCTGTTTTCATG
TTTTATGTACCAACAAGTTGTTTTGTACACTTCATTTCATTTGTTTTTGCTTTATTTGTC
ATGGACCAAATGAAGAAGAAAAAATCCGCATGTACATGACGTCATTCAATTAATAAAATA
AGTATAGAGTCGACCTATTTACTTACAAGTGGTGATTGGCCTGGCCAGTAAACTATATAC
TTTTTTTTTTGTTTTTCTTACTGTATTTGGTTACCTTTTACTCCAACAGAGAAAGAGAAA
TTACACCGATTCTTCTTTTTTGTTTTTATAAATTTCTCTCAACCTTAGGCAATGCACTTC
AAGAGTTTCTTACATGTTATTTTACTGGTACCTTTTTCAGTTCGGTGTACTTATTTACAA

TTGAGTTAACATTGGCGACTTCTTTATGTTACTTATATTCAAGGATGAAAGATATTGGCC
CTTGATGTCTCCATTACCATCATACGGCTATGGAAGAGAGCACAAGGGACCTCGATATGG
GAGCCTTATACACGGCCAAGACCTGAAGCATGTGACTATAACAGGTAGTTTGTGTCTTTT
TGTTCTTATTCATATGGAATATGCCAAATTGATTCCATCTTGTATACATTTTGGACCGTA
CAAAGGCATAGGAAGCGCTGAAAGCTTTTGAAATTAAATTTGATACTTATTTCATGGGAT
CACTTAATCTCTAGTTGTACTCGAATTAAGTGGATTAATTTGACCTTGAGCAATACATTC
ATGGTAGTGTGGATTTATTCCACTGAGGATATGCTAAATATAACTAAAATATCATCATTT
GTAGGTAACGAATGTTGGCGTTATTGCTTGTTTATCTTCTGCAGTCTCATTTTCATGAAA
TTGTAATAATATTTCAGTGGGCTCCTCTGCAAAATAGGTCATAATGGTACTATAAATGGA
CAAGGTCAAAGTTGGTGGGTAAAATTTCGGAGAAAGCTCCTTAATCACACAAGGGGTCCT
CTGGTGCAGCTCATGCGGTCAAGTAACATTATCATTTCTAATATCACTCTTCGTGATTCT
CCCTTCTGGACTCTTCACACGTACGACTGCAAGAATGTTACTATCTCAGAAACCACCATC
TTGGCTCCAATTGCTGGAGCTCCAAATACTGATGGGATAGATCCAGGTTACTCCTAATGC
TTGCAAAGTTTTCTGATTATTGAACTTTATTCATTTCACTTATAGCTGCATCCTGTAGAT
TGTATTGCAATCTAGACTCCACTAAAAATTTCAGCAACAATAGGGGCCCAAGAAACCTGC
ACTGCATGTCCATGTTCTCTTGAAGCCTCTTTTTTTTAAAAGTTTATGCTTCATTAATCA
ATCTGTTTGTTATTCTTGATGTATATGCAGATTCTTGTGAAAATGTGGTGATAAAAAACT
GCTACATTTCTGTTGGTGATGATGGGATAGCCATAAAGAGTGGTTGGGACCAATATGGAA
TTGCTTATGGCCGTCCATCAGCTAATATTACTATCCAGAATGTCGTAATCCGCTCTATGG
TCAGGTAAGATATTAGGTCGCTTGCTTATTCTGTATCACTTTAATACTGTATAACATATG
TATGCATGACGTGTTTTGAAATACCATTGGATAGTGAATGACGCTTCTTGTCATGAAGAT
AATATTTAAGAGGTGTTGCGAGTTTGTTAGATATTTTGTTTGTATACTTCAGATACATAC
CACTTGCTGGTTATTTGTTCTATGTTACAGATATACCATACGTACTAGGATCATTTATTT
GTCTAGACTTGAACTAGCCACATAAGACATTTGTCATTGTTGGTTTATCTTTCATGATCT
GGAAATCAATCAACACCACTATTCAAACCTTAATAAACCAATGTGACTGTATGTTTGATC
TTTTTTGTGTTCTGTGGGCAGTGCTGGAGTATCGATTGGAAGCGAGATGTCTGGTGGTGT
CTCGGATGTTTTGGTAGAGAATGTTCATGTCTGGGATTCACGGCGAGGCGTGAGGATAAA
GACTGCACCTGGAAGAGGGGCCTATGTAACCAATGTCATTTACCAGAACATAACCTTTGA
AAACGTACGTGTCGGTATAGTGATAAAGACAGACTACAACGAGCACCCAGACGAAGGCTT
CGACCCAAAGGCCGTCCCCACCATCGGGAATATCTCTTACACCTCAATCCATGGGCATCG
TGTGCGAGTACCGGTCAGGATACAGGGAAGCGCGCAGATCCCTGTGAAGAATGTTACCTT
CCATGACATGTCAATCGGTATAGTAGACAAGAAGCACCATGTTTTCCAGTGCTCCTTCGT
CCAGGGGCAGGTCATTGGCTACGTTTTCCCTGTGCCTTGCAAGAACCTGGACCTGTACGA
CGAGCGGCGTGGGCTGGTTAAACAATCAACGTTACAGAATATCTCTGATATCGACTACAG
TTTCTGACAATGGCATTTGATACCACTGTACTATTGCTGTAGGTGTATTCAGTATTCACA
GCCGTCACAGTCCTTTCCACACATTTAGAAGCAAAATTTTGGGTAGCTTTTACACTCTTC
ACCTCACAGTCAGGCATGTATCTAAGGAAGATCGACTTGACCATACTGTCTCGTAGAGGG
ACGCCAGGAATGTTGCCTTTTCACTTTTTTGATCACTGTATGATTACACTTACTTGGTTA
CACAGTCGACAGTACACTTTTGATAGTGCTTACTGTTCATGGCAGACTACATTTACCTTG
CCCTTTTTAAAACAGATGTGGATGTATATGTATTTGGTAGTTTGAACCTGGAATGGTTGG
TTCTTCTTCCAAGTTGTCTTCATATGAAGCTGGGTTTGTCGAGACAACTTCAAAAGAAGT
GGGTATTCATGATGTGAAGTGATTTAGCAGAAACAAACAGAGATATTTTAATTTGGCCAA
GACAGTCTTTTACACGCGCAAAGTTTCCACGCATTCGTGCGATGGCAGGAGATGGGTGCG
>ZmPG17

GGCGCACACAAGTACACAACCACCCAAGATGGGGAGGAGCCAGGAGCACAGGCCCAATGC
GGCAGCCATCCCACCACCTATCGCGTCGGCTTCACTCAAACGGCTTCTGCCGCATCCTAT
AAAACCTCCTCACCAACCTCCGATGGCGGAAATTCTTGCCCTCCCACACCGACCCCCTCC
CCCACGGCCCGCCTTCCCCCGGGAGCACGAGACCCTCGTTCCCTGCGCCACCGACTGAGC
CAAGGCCGCGCTTTGCCGTTTCCGACCTAGGTCGCGAGATCTCGGAGGAGCGGAGCCCCG
CCGTGCCGCCGCCTGCTACAGGGGGGCACGCAGGGACCGCACGGCGCGATGGTGGACACG
GGCGGGGGGAGGCTCTACCAGCGCCGTGGCGCGGTCGCTTTCGTCGCGGCCAACAAGGCG
CTGCTCGCCGCTGCGTGGGTCGTCGGGTTCGCGCTCGTGTTCCTATGGCAGAGCGCCTCC
ATGTCCTTCGGCAGCGCCGGCGGCGGAGGGTACCTCAGGCTCCTGTCGGTGCCGCCTCCG
CCGCCGCGCCCCGCGCCGCGGCTCCGCCCCACGGCGTACAACATCACGGACTTCGGCGCC
GTCGGGGACGGCCGGGCGGTCAACACCGCGGCGTTCGAGCGTGCTGTCGAGGCCATCGCG
GCGCTCGCGGAGCGAGGCGGCGGGCAGCTCAATGTGCCACCCGGGCGCTGGCTCACGGCA
CCCTTCAACCTCACCAGCCACATGACGCTGTTTCTTGCCGAGGGCGCGGAAATCCTTGGC
ATTACGGTAAAGAGTTTACTGTTTCTATGAACTTATCCGTGGGGCTTTAGTAATTAGATA
GATCTTCTCGTGTGCATTTTCTACGCTCCAAGCCTCCAACCAAAAGTTAATGCTGCTTTC
ATGTCGTGCGGAAATTGCTTAGTTATTAACTTATTCTCCAGTAGAATAGTTTATGATTTG
GCTAGTTGACGACAAACATCATGAATTGATTGCTGCTGGGTTTACCAGCATGCGAAAACA
AGAAAACACTCCTCCAAAACGTCAGAAACCGGCCCTAACGGCAAGCGAACTATTACAAAC
AAAAACCATAGAGCAACTAACAACAAAACTAAAGGACGCCCTCCACATCTAAATCTTGAG
TCCTTTTCCAGAAATCCTTCGACCAGTTTTTCATTCTTCCCATTTTGTCATCCAAATATT
TTGCATCCTACTCTCTCAGAAGAACTCACCACTTCTGCAAAAACATAAAGATTTTATGAA
ACAAAACGGCGATACTTTGTGCTGAAAACATCTTGACTCTAGACTAGCATGCATTCAGAG
TTTGGTAGATGCCTATGGTGCTTATTTGGGGGCACAAATGCCCACATTTCAGATTGTGAG
CTTATTTAACTGGCATTGTGATGTTTTCTGGACAGGATGAGAAGTACTGGCCATTGATGC
CAGCATTACCGTCATATGGGTATGGGCGGGAGCGCAAAGGACCACGATTTGGGAGCCTCA
TTCATGGACAGAATTTGAGAGATGTAGTCATTACAGGTTTGCAATTTATCTCGATACCTA
GCAATCTAGCATTTAAGCGCCACAATACACAGCATCTTGCATAATTGTGGATTAAGCAAC
ATCAGTGCAAAAGTGCATCATGTTCATCTTAAACAATAAGTGTTGCAACTCTGTAGCATC
ATCTGTTTGTAGCATAAAAGCAACTATTTGACGCCCAGTCTATAATGTACCCTTGTGCTT
CCCATATTAATATACGGAGGTCCTGCAATTCTGTTGAAATAGGATCTAGCATTTTACAAC
TTGAACTGCCATATTCTGTAATGATATTTTGCTGATTAAACAGGCAAATTCATTAAAAAT
GCAACAAATGCACTTCTGTCACAAATCCAATGCTGTAGCTGTAGCTAGCTTATTAAGCTT
GTTTGCTCCCCAATCTTATGCTGCTAGAACAATGAGATTCTATTTCACTGACTAATTGAA
TATAGTTATCTGATCTCCTTCTTCGGTGTGTTAAATAGGACATAATGGGAGCATAAATGG
CCAGGGTGAAGTTTGGTGGATGAAGCATCGCAGAAGAATTCTGAATAACACAAGGCCTCC
TCTTGTGCAGCTGATGTGGTCCAAGGACATTATTGTTGCAAACATAACACTGAGGAACTC
ACCTTTCTGGCACTTGCATCCTTATGATTGCACAAATGTGACTGTTTCGAATGTTACTAT
CATGTCTCCTGTTTCTGGTGCTCCAAACACAGATGGCATAGATCCAGGTATTCTTGCTAC
ATCTGTATTTAACAGCTTTCGAGTACATCATTCATGTTTTAAACGCTTTGGCACTGTACC
AAACACTTGAAGTACCAGTAGATATACACCCATTTTTCTATAACATCCTGTCTATACTAA
CTCTTGCACTAGTTACAGGTAATGTTGAATTGTTGACCACAAATCATAATGTAGATTCCT
ATTTCTCACATATAAGTGTATGCAGATTCTTGTCAGGATGTGCTTATTGAGAATTGCTAC
ATATCGGTTGGCGATGATGCAATAGCTATAAAGAGTGGTTGGGATCAGTATGGTATTGCA
TACGGTCGTCCATCTTCTGACATTTTAATTCGCAATGTAACCGCCCGTTCTCTTGTGAGG

TGAGAATTGAGATATGTTGGCTACATTGTGTGTCTACAATAATCTACTTTTGTGCATAGT
CCTTTGAAGTCAAACCTCAAGAGTAGTAAATTACGACTATGGTGTGTATTGCTGAACATT
ATCAATCAGGCTTACACTATCTACCCTATCTTTGTGCAGTGCTGGAATTTCAATAGGCAG
TGAGATGTCTGGCGGAGTTGCAAATGTTACTGTGGAGAATGTGCGCATCTGGGAATCAAG
GAGAGGTGTGAGGATAAAGACTGCCACAGGAAGAGGCGGCTACATCCGTAACATCTCATA
CCGCAACATAACTTTCGACAATGTCCGTGCTGGGATTGTGATAAAGGTTGACTACAACGA
GCACGCTGATGATGGATACGACCGGACTGCCTTCCCAGACATCACAAGCATATCATTCAA
GGGAATCCATGGGCAGGGTGTTCGGGTGCCTGTCCGAGCTCATGGCAGCGACGTCATCCC
CATCAAGGACATCAGCTTCCAGGACATGTCGGTAGGCATCAGCTACAAGAAGAAGCACAT
CTTCCAGTGCTCCTACCTCGAGGGGCGTGTCATCAGGCCTGTGTTCCCGAAACCATGTGA
GAACCTGGACGTCTATGATGAGCAAGGGCAGCTTGTGAAGCGGGCAGTGGCCCTAAACAG
CACAGAACTTGACTACGATATATGAGGTGCACATATCATACCACAGCTTTGATAGTTGTA
GTGGAAATTGGTGCTCTCTACCCTCAGAAATCAATTTTCTCCGATTGTCCATGGCTTTTT
GGGTCTAGAATTGTAGATAGAGAGGAGATTCTCCTCTTGATAGGTTTGCTGCTTACAGTG
GCTGAATGTATCTCACCGGCGATTTACTGATTTGTCCATGAACATTTGTAATCGCCAGAA
CCTTCCTGGCGACAAATGTATGCTGTCAATATATTGTACACCTGAAGATTGTATTGGAGT
ATACACAGTACATGATCTCTAAATATCTGCCAGCAGATTTTTC
>ZmPG50
ATGGCGTACGGCGACCGCGTCATCACCTTCGAGGACTCCGAGAAGGAGAGCGAGTACGGC
TATGTCCGCAAGGTCCGCGCCTCCCCCTTCCTCTCCCGCCCGCATCGCTTGCGCCTAGAT
CCCGCTGCTCGGTTTCACGCGATCTATAGTGGGCGTGGGCTGCACGCCTGCACGGCACCG
TGTATGGGTCGTCGGCTTCTGGCGTGCTCGGATCGAGCTAAATTGCTGTCAGATTTGGTT
ACCTGCGGGTTCTAGGCATGGGATTTGGGTGATGTTGGCGGTTACTGTCATGGATCTCTT
AAGATCTAGCCAGCATTGGTGGTCAGGTGGGGGTGGCTTGGTGCATTGGTTTTGGAGATT
TGCGGGGTGGTGTTTTGATTGCAATGTTGTGATTTGACTACCTCGATTCTCGAGAGTTAT
TGGGGTCTTTGGTTCGCGCTGCCATGTAATTAGTTGAATTAGTGAAAGATGTGCATAAGT
TTACCATTTTGTAGAAGCTAGATTCATTTTTTGAGCTTGGTATTGATCGATCATGGCCAT
TTTCAGGTGAGTTTTACTACACTAATAATGTGTGTGATTTAGGTGGGGCAAGTGCGTGGA
CCAGCAGAGTGCTTTGTGTTGCATTTTGTTTGTAGAAGTATTAACATTGTTTTTCTTTGC
ATAATCAAAGAGAATGAAGTCCCATAGTGCACATACAACGCCAGAGGTTGGCAGTGGTTG
CAACGCCGAAAGCAGAAGTTGTTGGCTAACTGGCCATTGAATACTTTGTAGTACAACCAT
TGATCAACCTGTTAGTAGAGCCTTGGCTTACCAAGTTCATGTGTTGTGATCCTCTTTTCC
TTAGTTTCAATATAATGCAGACATGCTGGTTAGATTTGTTCTGCTACTGTCACCCACATC
TTGTTTGCACCATTTGCATCCGTCCAGTGATCCCTATGTTGTTCGTGTATCTTGTGTAGG
ACATGAAGCATTGGCCCCTCATAGCTCCCCTGCCATCCTACGGGAGAGGAAGGGACGAGC
CTGGCGCAAGGTACAGCAATTTCATTGGAGGATCCAATCTCACTGACGTCATCATCACAG
GTACAATACGTGCGCCTGCCTTCGAGTAGCGTATCCAAGGTCAGGTGGTAGCTACAGAAG
ACTGATGAGCTGCACAAATTTCAGGCAAAAATGGAACGATCAACGGGAAGGGGCAGGTCT
GGTGGGACAAGTTCCACGCCAAGGAGCTCAAGTTCATCCGCGGCCACCTCCTCGAGCTCC
TCTACTCTGAGAACATCATCATCTCCAATGTCACCTTCGTCAACGCGCCGTACTGGAACC
TCCACCCTACCTATTGCACGTATGGATCTCACTCTCAGCTCTGAAAACCTAAAGTCACTG
ATCATCTGTTCCCGTGGTGGTTTGTGATCATTGTTTCAGTCAGCGGTGAATCTCACATAT
CATGCCACTGTTTTGCGCATCAATGTGACCATCAGTGGCGTCACAATTCTCGCGCCGGTG
AATTCGCCTAACACCGATGGAATTGACCCGAGTAGCTAGACCAGTCAGCTTCCAGCCATG

TTCCTTTGACGCTAAGTGAGAAGCACATGTTTCTAATTCTGAATTTCATTGCCTGTGCGT
GTGTGCAGAGTCTTCCTCGCGTGTCAAGATCGAGGACTGCTACATCGTCTCCGGCGACGA
CTGCGTGGCCGTGAAGAGCGGGTGGGATGAGTACGGCATCAGGTTCAACATGCCGAGCCA
GCACATCGTCATCCAGAGGCTGACCAGCGTCTCCCCCACGAGCGCCATGATCGCGCTGAG
CAGCGAGATGTCGGGCGGCATCCGCGACGTGCGTGCCAAGGACAGTGTGGCCATCAACAC
GGAGTCGGCCATCAGGGTCAAGACGCTTGGAGCAATTGCTGGTGTTCTGTCGAGGAATGA
GCTCGTCGCTGCAGCTCTCTTCACTCTTGCAATCAATCACAAACAGGTTTGCCTCATGTT
TAGAAAATTGTGTTCATTCAGTCATTCATAAGCTTTGTTACCATACATGTACAATCAATC
ATCCTAGAGAATGCTATGTTTCTCTAAGCCCATGCGTTTCCAATTACACAATGTGAAATA
GTAGTTCTTATGGTGGATACTTCCTTATTTAGTTTAGGATGTTGTCACTGTGAATACAAA
GCAGACCCTATGATCCAACTGAAAATCATGTCAACGACCAACCAAATTGTTAATTTTATT
CTTTGATTATGATGTTGTCAGGCTAATACATAATGAGTATTTGGTTTTACACATGCAGGT
GGATGTGTTCTCTTTTGGGATAGTTTTATGGGAGATCTTGACTGGTGAGGAACCGTATGC
AAATATGCATTGTGGTGCTATCATA
>ZmPG24
ATGGCGTACGGCGACCGCGTCACCACCTTCGAGGACTCCGAGAAGGAGAGCGAGTACGGC
TATGTCCGCAAGGTCCGCGCCTCCCCCTTCCTTTCTCGCCCGCATCGCTTGCACCTAGAT
CCCGCTGCTCGGTTTCGCACGATCTATAGTGGGCATGGGCTGCACGCCTGCACGACACAG
TGTATGGGTCGTCGGCTTTTAGCGTGCTCGGATCGAGCTGAATTGCTCTCAGATTTGGTT
ACCTGTGGGTTCTAGGCTTGGGATTTGGGTGATGTTGGCGGTTACTGTCGTGGATCTCTT
GGGATCTAGCCAGCATTGGTGGTCAGGTGGGGGTGCCTTGGTGCAGTGGTTCTGGAGATT
TGCGGGGTGGTGTTTTGATTGCAATGTTGTGATTTGACTACCTCGATTCTCGAGAGCTAT
TGGGGTCTTTGGTTCGCGCTGCCATGTAATTAGTTGAATTAGTGAAAGATGTGCATAAGT
TTACCATTTTGTAGAAGCTAGATTCATTTTTTGAGCTTGGTATTGATCGATCATGGCCAT
TTTCAGGTGAGTTTTACTGCACTAATAATGTGTGATTTAGGTGGGGCAAGTGCGTGGACC
AACAGAGTGTTTTGTGTTGCATTTTGTTTGTAGAAGTATTAACATTGTTTTTCTTTGCAT
AATCAAAGAGAATGAAGTCCCATAGTGCACATACGACGCTAGAGGTTGGCAGTGGTTGCA
ACGCCGAAAGCAGAAGCTGTTGGCTAACTGGCCATTGAATACTTTGTAGTACAACCATTG
ATCAACCTGTTAGTAGAGCCTTGGCTTACCAAGTTCATGTGTTGTGATCCTCTTTTCCTT
AGTTTCAATATAATGCAGACATGCTGGTTAGATTTGTTCTGCTACTGTCACCCACATCTT
GTTTGCACCATTTGCATCCGTCCAGTGATCCCTATGCTGTTCGTGTATCTTGTGTAGGAC
ATGAAGCATTGGCCCCTCATAGCTCCCCTGTCGTCCTACGGGAGAGGAAGGGACGAGCCT
GGCCCAAGGTACAACAATTTCATCGGAGGTTCCAATCTCACTGATGTCATCATCACAGGT
ACAGTACGTGTGCCTGCCTTCGAGTAGCGCATCCAAGGTCAGGTGGTAGCTGCAGAAGAC
TGATGAGCTGCACAAATTTCAGGCAAAAATGGAACGATCAACGGGCAGGGGCAGGTCTGG
TGGGACAAGTTCCACGCCAAGGAGCTCAAGTTCATCCGCGGCCACCTCCTCGAGCTCCTC
TACTCTGACAACATCATCATCTCCAATGTCACCTTCGTCAACGCGCCGTACTGGAACCTC
CACCCTACCTATTGCACGTATGGATCTCACTCTTAGCTCTGAAAGCCTAAAGTCACTGAT
CATCTATTCCCGTGGTGGTTTGTGATCATTGTTTCAGTCAGTGGTGAATCTCACATGTCA
TGCCACTGTTTTGCGCAGCAATGTGACCATCAGTGGCGTCACAATTCCCGCGCCAGTGAA
TTCGCCTAACACCGATGGAATTGGCCCGGGTAGCTAGACCAGTCAGCTTCCAGCCATGTT
CCTTTGACGCTAAGTGAGAAGCACATGTTTCTGATTCTGAATTTCATTGCCTATGCGTGT
GTGCAGAGTCTTCCTCGCGTGTCAAGGTCGAGGACTGCTACATCATCTCCAGCGATGACT
GCGTGGCCGTGAAGAGCGGGTGGGACGAGTACGACATTAGGTTCAACATGCCGAGCCAGC

ACATCGTCATTCAGAGGCTGACCAGCATCTCCCCCACGCGAGCGCCATGATCGCGCTAGG
CAGCGAGATGTCGGACGACATCTGTGACGTGCGTGCCGAGGACAGCGTGGCCATCAACAC
GGAGTCGGGCATCAGGGTCAAGACGCTTGGAGCAATTGCTGGTGTTCTGTTGAGTAATGA
GCTCGTCGCTGCAGCTCTCTTCACTCATGCAATCAATCACAAACAG

>ZmPG6

ACTCTCTCCCGCGGGTCTCGGCCTCCTCTCCTCGGCTCGCTATAAGCAGGCTCCACCGCT
TCCGCAATGGCCACCTAATTCGCTCGGCGTCGGGGGAGAAGAGAAGCGCGCGCGATGGCA
CCGGGAAGATCTCTTCGCCCTGTAATAATTCCCTCGCCCCCGCGCTGCATCTTCGATGAC
GGACCAAAATCCGGGACGATGTTTTGGTGGTCAGTCTCGTCAGTCACTGATCAAGCGGCA
TTCGTACGTGTTGGTTGTTTTTGCAGGCGGGGACGGCGGCGGTTCTGTGGGCGGCGTCGC
TGCTGCTGCTGGCGACGTCGTGCGCGCGCGCAGGTGGCGCGTACGCGGGGCCGGGGTGCC
GGAAGCACGTGGCGAGGGTCACGGAGTACGGCGCGGTCGGGGACGGGAGGACGCTCAACA
CGGCGGCGTTCGCCAGGGCTGTGGCGGACCTGGCGCGGCGCGCGCCCGACGGCGGCGCTG
CGCTGGTGGTGCCGCCGGGGAAGTGGCTCACGGGGCCCTTCAACCTCACCAGCTGCTTCA
CGCTCTACCTCGACGAGGGCGCCGAGATCCTCGCGTCCCAGGTGAGTGAGAACCCAACCC
CAGATCCTCCTCCCGCCATTCCGCTCGCCGCGCCCGCTGGCCTTCCCCTGCCGTGGCTAC
GCCGCCGCGCTTAGCCGAGGAACTGTCCCCGTACTCGTAATGGGCCCGTGACCGTGCGGG
TGCGGTAGTTTAGCATGACATGTGGGGCCATTGAACACCGCTCGCAGACAGTCTCTTCAT
TCATCACGGATTCACGGGACACGTCCCGTCTGCGTAGGCCGTAGGCCGGTCTCTTTTTCA
CTAGGGACAACGGCTTTTGCCCTGGGGGACCCCGAACCCTTCTTTTCTAAGCAAAGCCAC
TGCCAATCCGACATATCTCTTGAACAGTTGAACTCAATGCCATGGTTCCATTCCATGACT
CCTCTCTGGGCCTTGTGCATCACTGCTTTGTATGGCAGAATGCATAGCTTGCAGATCTGC
ACATGTAAATAGAAATGTTTGTTTGTAGTGTATCATTTATTGAGAATCCAGAACGTTTCA
TCAAGACATGATAGTATACATTTTTCCCTGGAAAGACCAGTGGTAAATGTTTGCTCCGTC
TTTACTGAAGTTAATGTAGTCTGAGTAACGTAAGAAGGGCAATTAGAAACTTTTTAGTGC
TGCTGGTGTAGCGTTGATGACTAATCCAAAACAGACCCAGGTTTGAAGTTCGTTGGTACA
CATTTACACAAAAACTAATAACTACCTCTCTCAGAAAACAAGATCTAGTAATTACTCTTT
GTCTACCAAGGTGGCGCTTAAATATGTATCCCAAGGCAAGGACTCGGCTGGCTTTGATTG
ACACCCGACTTTTTGTACTAGTGCTTGGCAGTGGTTGCGACGCCGAAAGCAGAAGCTGTT
GGCTAACTGGCCATTGAATACTTTGTAGTACAACCATTGATCAACCTGTTAGTAGAGCCT
TGGCTTACCAAGTTCATGTGCTGTGATCCTCTTTTCCTTAGTTTCAATATAATGCAGACA
TGCTGGTTAGATTTGTTCTGCTACTGTCACCCATATCTTGTTTGCACCATTTGCATCCGT
CCAGTGACCCCTATGCTGTTCATGTATCTTGTGCAGGACATGAAGCATTGGCCCCTCATA
GCTCCCCTGCCGTCCTACGGGAGAGGAAGGGACGAGCCTGGCCCAAGGTACAGCAATTTC
ATCGGAGGATCCAATCTCACTGACGTCATCATCACGGGTACAGTATGTGTGCCTGCCTTC
GAGTAGCGTATCCAAGGTCAGGTGGTAGCTGCAGAAGACTGATGAGCTACACAAATTTCA
GGCAAAAATGGAACGATCAACGGGCAGGGGCAGGTCTGGTGGGACAAGTTCCACGCCAAG
GAGCTCAAGTTCACCCGCGGCCACCTCCTCGAGCTCCTCTACTCTGACAACATCATCATC
TCCAATGTCACCTTCGTCGACGCGCCGTACTGGAACCTCCACCCTACCTATTGCACGTAT
GGATCTCACTCTCAGCTCTGAAAGCCTAAAGTCACTGATCATCTGTTCCTGTGGTGGTTT
GTGATCATTGTTTCAGTCAGCGGTGAATCTCACGTGTCATGCCACTGTTTTGCGCAGCAA
TGTGACCATCAGTGGCGTCACCATTCTCGCGCCGGTGAATTCGCCTAATACCGATGGAAT
TGACCCGGGTAGCTAGAGACCAGTTAGCTTCCTTTGACGCTGAGAAGTGAGAAGCACATG
TTTCTGATTCTGAATTTCATTGCCTGTGCGTGTGTGCAGATTCTTCCTCGCGTGTCAAGA

TCGAGGACTGCTACATCGTCTCCGGCGACGACTGCGTGGCCGTGAAGAGCGGGTGGGACG
AGTACGGCATCAGGTTCAACATGCCGAGCCAGCACATCGTCATCCGGAGGCTGACCTGCG
TCTCCCCCACGAGCGCCATGATCGCGCTGGGCAGCGAGATGTCCGGCGGCATCCGCGACG
TGCGCGCCGAGGACAGCGTGGCCATCAACACGGAGTCGGCCGTCAGGGTCAAGTCCGGCG
CCGGGAGGGGCGGCTTCGTCAGGGACATCTTCGTGCGCGGCCTCAGCCTCCACACCATGA
AGTGGGTGTTCTGGATGACCGGCAACTACGGGCAGCACCCCGACAACACGTCCGACCCCA
ACGCCATGCCCGAGGTCACCGGCATCAACTACAGCGACGTGTTCGCCGAGAACGTGACCA
TGGCCGGCAGGATGGAGGGCATCCCTAAGGACCCCTACACCGGGATCTGCATATCCAACG
TGACCGCCCGCCTCGCGCCGGACGCCAAGGAGCTGCAGTGGAACTGCACCAACGTCAAGG
GGGTCACCTCCCACGTCTCGCCCAAGCCGTGCCCGGAGCTCGCCGCGGAGGGCAAGCCGT
GCGCCTTCCCGGAGGAAGAGCTCGTCATCGGCCCACCCGAGCTGCCGAAGTGTAGCTATT
GAGGCCGATTGCCAAAAGAGATCATTGATCATATAGGCTCTTCGGTGATAGTCTAGACAA
GCAGTATGAGAAATGCAGCTGAAATACGTTTGCATTACAGTGTCCGTCTCACATTACTGC
TTCCTCCGATTACAATGTCTGGCAGTGGTATAACTTGTGACTACAAATTATGCATCAAGA
AGAAATCAGCAAAACGGACATGATCATT
>ZmPG37
ACTCCACTCCATCATCCCGTGCCTCACGAGTCACGAGTCACTCCAGGTCCAGGCTGAGCA
GCAATGGCGCCGCGAACGCTCGGCCTCAACGCGGCCCCGCTCCTCCAGGTACGTAACGTA
ACTCACTGCCCCGAAACTCTCACCGCGTTCGTTCGTGGCGGCCGCGTGCGTGTTCCTTCT
TCCTCCTGCGCTAGCTGCGCGGTTCTGCTCTTCAGTACTCCTCTTGTCCTGACAGATCTC
ATCTAGCTCCCAGCTGAAAGTTTCACGCCTTTCCGGTCATTATGTGCGCATGGAGGTGCT
GAAAAGAAAAGTGCCGTCTTTTTGCCTCACAAACCAGAATGTTTTTTTTTTCAATTCAAG
ATCAGAATTTTTTTCCCCAGTCCACCGAACCAGATCGATCAGGTCATGTATGTGGTAGCA
GCCAGTTGTTGATCTGAAGCACACTCCTGTTTCCTGGAACAGATTCTGCGGTCCCTGTGC
CTCCTGGCCCTCGCCGCGGCGGCGGCGGTGTCCGGGCGGCGCCACGGGGCCTCCCCGGCG
CGCGGCAGCGGGCAGAGCATGTACCTGGCGCCGGCCTGCCGGGCGCACACGGCGTCGGTG
GCGGACTTCGGCGGCGTGGGCGACGGCACCACTTCCAACACCGCGGCGTTCCGGTCCGCG
GTGGACCACCTGTCGCAGTACTCGGGCGAGGGCGGCGGCGGTGGCATGCTGTACGTGCCC
GCGGGCAAGTGGCTGACGGGCCCCTTCAACCTGACCAGCCACTTCACCCTCTACCTCCAC
CAAGACGCCGTCATCCTCGGCTCCCAGGACGTAGGCGAGTGGCCAATCGTGGATCCCCTG
CCTTCTTACGGAAGAGGGAGGGACAAGGTCGGAGGGAGGTTCGCTAGCCTCATCGGCGGA
TCGAACCTGACCGACGTAGTCATCACTGGCAGCAACGGGACGATCGATGGGCAGGGCGCC
ATGTGGTGGTCCAAGTTCCACAAGAACCAGCTCAAGTACACGCGCGGCTACCTCATCGAG
CTGATGCACTCGGACACCATCTACATCTCCAACCTGACGCTGCTCAACTCGCCGGCGTGG
AACATCCACCCAGTCTACAGCAGCAACATCGTGGTGCAAGGCATCACCATCCTGGCGCCG
ACCAACTCGCCCAACACCGACGGCATCAACCCAGACTCCTGCTCTCACGTCCGCATCGAG
GACTGCTACATCGTGTCGGGCGACGACTGCGTGGCCATCAAGAGCGGCTGGGACGAGTAC
GGCATCTCGTACGGGATGCCCAGCCAGCACATCGTCATCCGCCGCCTCACGTGCGTGTCC
CCGACCAGCGCCGTCATCGCGCTGGGCAGCGAGATGTCGGGCGGCATCCAGGACGTGCGC
GCCGAGGACATCACGGCGATCAACACCGAGTCCGCCGTGCGCATCAAGACCGCCGTCGGC
CGCGGCGCCTACGTCAGGGACGTGTTCGCGCGCCGGATGACGCTCACCACCATGAAGCGG
GTGTTCTGGATGACGGGCGACTACAAGTCCCACCCGGACGACAAGTACGACCCCAACGCC
GTCCCCGTTGTCGCCAACATCAGCTACCAGGACGTGGTGGCCACGGGGGTCTACAAGGAG
GCGGCGCGCCTCCAGGGCATCCAGGGCGCACCGTTCCGGGGCATCTGCGTCGCCAACGTC

ACCGCCGACCTGTCCAAGTCCAGGAAGTACCCATGGAACTGCGCCGACATCGAGGGAGTG
TCGGCCAACGTCAGCCCCGCGCCGTGCGACCCGCTACAGGGCGCGCACGACGGCGCATGC
CCGTTCCCGACCGACACGCTGCCCATCGACCAGGTCACAGTGCAGCAGTGCGCATACGAC
ATCCCGGCCACCAACTAGCAGCTCAATCAAGGAGTGTATTCGGATGTGGATCAGCCGCCT
AGTACAACTTGTAGTTCAGGTCCAATGCAAGCGACTAGTAGTATCAAGTCTCATCAACAC
AATAACGACAGAATGAATCGGGCACTAGCCTAGAAAGCATTCATAAA
>ZmPG44
AACCTTTTGTTTGTTCTTTTGTTGCCTTCCTTTTCTCCGCACTCATCGCTTGTTGCGGTT
CGCTCTCTACAAGGAGGCGGCTTGCGCGGGGGGCATCGTGCAATGGCGCGGCGGTGCCCG
TGCAGGCTGCTGCTAGCCGTGGCGGCGGCGGCCGTGCTCGTGGCCGCGGCAGCGCAGGCG
CAGGAGACGTGCTCGGGGACCGTCCCCGCGCCGCCGCGCCGCGGGGCGCGGGTGTCCGTG
GCCAGCTTCGGCGGCGCGGGCGACGGACGGACGCTCAACACGGCGGCGTTCGCGCGCGCC
GTCGCCAGCATCGAGCGCCGCGCCGCGCCGGGCGGGGCGGAGCTCTACGTGCCGCCCGGG
GTGTGGCTCACGGGGCCCTTCAACCTCACCTCGCGCATGACGCTCTTCCTCGCGCGCGGC
GCCGTCATCCGCGCCACGCAGGTCAGCTGGCAAGCAAGCCTCCTGCTCTCTCCCGCGCCC
GCCATTGCTCTAGGGCTTCTGCCCAAGCCATGGCGTTCTCGTCTTTGATCGATCATCACA
AAGACAGTTGTTTTTACTACAAGGGTGGATCAGTATTTGGGGTTTTCCTCTTCTAGAGAT
CTAGAGACGCGATCGAATTATAAACGACCCTAGCGATTTCCCGGAATGGAATTAGAATGC
ATGGATCTAGTATTTGTGATATGGCCGTCGTTCATGTTCTTCCACTAACTTATAGACTTA
ATAAATCCGTCTTCCGTTTATTTTAGCGTTCATTAATATTTTGGAATTATGTGCGGTAAA
AGGCATAGAGAAGACAATTACCCAGGTTGATCTGAAACATTTGCTTTTCGACCTCTTGGT
GCAGGACACATCAAGCTGGCCTCTGATTGAACCGCTGCCCTCATACGGGAGAGGACGTGA
GCTGCCCGGCGGAAGATACACAAGTTTAATCCATGGCAATGGGCTTCAGGATGTTGTCAT
CACAGGTTCTTGCTTTGTGACTACTCAAATTCTCATGTTAAATTACTATGATTATTGTAG
AGGCATGAAACTACTACTAAAAAATGCCATGAAGTTACTCCTGTAGTGAACTCTTTTGGA
AGCTTAAGGTTCAAATTTCAATCTTCCAACATGTTGCAATTTCATAAAAACACCAGGACA
AAATAACCTGTGCACGCTCATATGTTCACATTCCTCCATATACAATAAAATCTTCAGTTA
AGTTATTGATGACGATGATAGTTTGTGAAGGAAACCGCATTGTGTGCAGGCGAGAATGGA
ACTATTGATGGTCAAGGCAGCGCGTGGTGGGATATGTGGAAGAACAGGACACTGCTCTAC
ACAAGGCCTCACCTTCTTGAGCTGATGAGCTCTTCTGATATCATTGTCTCCAATGTCGTC
TTTCAGGATTCGCCATTCTGGAACATCCATCCTGTTTATTGCAGGTGAGATGAACAATTC
TTTCTGCCGACTTCAGACCAGAATCTCCCAACTCTAACGTGTTCCTTATTCCTTATGAAT
AGTGCTTACAAAGAGGAAAACCACTGGACTACTCATGGATTTAGCAGAGATGATACACTG
CCATCTTGCTGCCATTTTTGCAGTCCAGATGCTTTTAGTTCCTTTGCGCACATAGTTGGT
TCGATCATTCGATGCCATCTTCAGTATCCATTGAATAGACGTACAACTTGGGCCCTCATT
CATTTGCACCATCATAGATTGCACAAGAACTACAGGCTGGGTGTCCTATCGTGTCATCAT
AAGAGTAAGACCACACAAACCGTTAGGTGCAACCAGTGAACCATGACCGAAGTACAGAAA
TTGCCAGATAATATACCCAACAAAGCCAGCACACTGATGTAGGTTCAGCTTGTTTTGTTG
TAGCTGTTTCTAGCAGCTGCATAGTTGTGGTCCAAAACCTGCTTTTATTGTTGTCCCAGG
CTCCCAGCTGAGAAATAAATGCTGGATTATTTTTCCTGTGTGCAGCAATGTTGTGATCAG
AAACGTGACTATCCTAGCTCCACATGACTCCCCCAACACGGATGGAATTGATCCAGGTAC
AGATATTTATCCCCTCCTGGTCAAGATACAGATGCACGAATGTGCTCCGTTCTCTCGATA
TTAAAGCTGGCGCCGTGGTCCGAAATTTTGCAGATTCCAGCAGCAACATCTGCATCGAGG
ATTGCTACATTTCTACTGGTGACGATTCCATCGCCATCAAGAGTGGCTGGGATGAGTATG

GAATCGCCTATGGCCGCGCCAGTTCTGGCATCACCGTGCGGCGGATCACAGGCTCCTCCC
CGTTTGCTGGCTTCGCGGTCGGAAGCGAGACATCAGGTGGCGTGGAGAACGTCCTTGCAG
AGCACTTGAACTTCTTCAACTCAGGGTTTGGGATCCATGTCAAGACCAACACAGGCAGGG
GCGGCTTCATCAGGAACATCACTGTCTCGGATGTGACGCTGGATAACGTCCGGTACGGCC
TGCGGATCGTTGGCGATGTCGGCAATCACCCCGACGACAGCTACAACCGGAGTGCGCTCC
CGATCGTCGACGCCCTTACGGTAAAGAATGTCCAGGGCCAGAACGTCAGGGAGGCTGGGC
TGATCAAGGGCATCCCCAACTCGGCCTTCTCCCGGATCTGCCTGTCGAACGTCAAGTTCA
CTGGTGGCGCACCTGTCCGGCCGTGGAAGTGCGAGGCTGTCAGCGGTGGCGCTCTCGACG
TGCAGCCGTCACCATGCACAGAACTGACTTCAACGTCTGGGACGAGCTTCTGTACAAATT
CGCTTTGATGCTTTGCCAGCTAGCTGAGCTGCTGCTATGCTTCTGTATCTACGAGTATGT
ATACTGGTGCTTTAAAGCTTGGGCACCCAAGTATTTATCTTCCCGAAAATTAATTTGGTT
TTACT
>ZmPG46
CTGCCGTGCCACTGCCATCCTCGAGCACAAGGGACCGACCAGACCCGATCCACAGCTCAT
CACACACACACACAGTGGCGAGCTCGTCAATTGCAGGCAAGACAGCAAGCGCATTTACAC
AGCACGCTGAAAGCCGGGACGCAGAGCAGCTTTTGCGCCTTGTCCCCTCCTGCTCGTCGC
TTCCCTCACTCTCACAGCCACGCCACGCCACGAGAGAAGAATGCCGCCGACGCTGGGCCT
GCTCCTCGCGGCGGCGGCGGCGGTCCTGCTGCTGCTGCTGCCGCCGCCACCATCGGCGGG
GGCGCAGGAGACGTGCTGGTCGGGGCCGGCGCCGCGGCGCGGGGCGTGGATGTCCGTGGC
CAGCTTCGGGGCGCGCGGGGACGGGCAGACGCTCAACACGGGCGCGTTCGCGCGCGCCGT
GGCGCGCATCGCGCGTCGCCGGGGCGCGCGGGGCGGCACGCTGCTGTACGTGCCGCCCGG
GGTGTGGCTCACGGGCCCCTTCAACCTCACCTCCCACATGACGCTCTTCCTCGCCCGCGG
CGCCATCGTCCGCGCCACCCAGGTACCGGCTACCGTACGGGCAATTGCCCATCGCCCTTC
GTGGTTCTTGGTCGCCGCCACTGCCAGCCATGCCTTGCTACCATCGAACTAATAAGTGAG
CAGGACACGTCGAGCTGGCCGCTCATCGACCCGCTGCCGTCGTACGGGAGAGGGCGCGAG
CTGCCTGGCGGGAGATACATGAGCTTGATCCACGGCCACGGTCTGCAGGACGTCTTCATC
ACAGGTTCTTTCTTACACTGCCAGCCAGACAGCCACCCACTGAGCCACTCCTCCATCGAT
CTGCTCAAAGCTCCGCATTCTGTTCTGGTCTGCGGCGTCAGGTGAGAACGGCACCATCGA
CGGCCAAGGCGGCGTGTGGTGGGACATGTGGAAGAAGCGCACGCTGCCCTTCACGAGGCC
GCACCTGCTGGAGCTCATGTACTCCACCGACGTCGTCGTCTCCAACCTCGTCTTCCAGGA
CTCGCCGTTCTGGAACATCCACCCGGTTTACTGCAGCAACGTAGTGATAGCAAACCTGAC
TGTGTTGGCGCCGCACGACTCCCCCAACACCGACGGGATCGATCTAGGTAAGGTAAGGTA
AATAAATACAATACCACCTGAGCTGAGCATTCCTTCCTGCTCTGCTGTGCTGACAAACAA
CATCTCCGCCCTGCAGATTCAAGCAGCAACGTGTGCGTCGAGGACTGCTACATCTCCGCC
GGCGACGACCTGATCTCCATCAAGAGCGGGTGGGACGAGTACGGCGTGGCCTTCGGCCGC
CCAAGCTCCGGCATCACGGTCCGGAGGATAACCGGGTCGGGCCCGTTCGCCGGCTTCGCG
GTCGGGAGCGAGACCTCGGGCGGCGTGGAGGACGTCGTGGCCGAGCACCTGAGCTTCTCC
GGCGTGGGCGTCGGGGTTCACGTGAAGACAAACTCGGGCAGGGGCGGCTTCATCCGGAAC
GTCACCGTCTCCCAGGTGACCCTGGACGGCGCCCGCTACGGCCTGAGGATCGCCGGCGAC
GTTGGGGGCCACCCCGGCGCGTCCTACAACGCGAGCCTGCTCCCCGTCATCGACGGCGTG
GCGGTCAGGAACGTCTGGGGCCGGAACGTCCGGCAGGCCGGGCTCATACGGGGCATCAGG
GACTCGGTCTTCAGTAACATTTGCCTCTCCAACGTGAAGCTCTATGGCATTGGCAGCGAC
TCCATCGGGCCGTGGAGATGCCGAGCCGTCAGTGGCAGCGCACTCGACGTGCAACCGTCG
CCATGTGCGGAGCTGGCTAGTACGTCCGGGACAGGTTTCTGTACCTGAATCAGTTTTTCC

GCCTCCTATAGCAGCAATAAAGACGTCAGCATTTCTCCATTTTGTTCATCACCTACAGGG
CCCTTTGTTTCTTTGGCAAACTCTTCTGCGATTCAGCTGTCACTGTTGAATTCTGCTGTT
GGCAGAGGATAATCTATTTTTACC
>ZmPG16
ATGCAGGCCAAAACTGGCTCTCTTCACAAGGCACATCCCTCTTCACTCTCTCCTCCGTCG
TCCCGTCACTCGTCAGTCCTCGCTCTCTTCACTCGCTGAAAGGCAGGCTGGTGGAGTGGA
AGCGCTTCACTCCCCTTCGTGCCACCCCAGCCCATCGCCTTCCACTGGACTCATCTGCTT
GGCACTCCTCGCCCCGTGCTTGGATTCTTGCACTTGGTGCTTCCCTTCTCCTACCTCACA
TCACCCGCCCAGATCCAATCCACACGAGATTTGCGACGCTCCGGCCTCCCGGCACGAAGC
CTTTCTAGGGATCGCCACTCGCCACACGCCGGCCGGGGCGAAAAAAGCTCTTCTTGGAGC
CCTTCTGCTTGGCCTCTCCCTCTCTTCTCTCCCGGCGTCATTGTCGCCGCTGCCTGCGAG
CAACAACAATTATGGCCGCCGCGACGTCGCGCGGGCCAGCGTCGGTCGCCATCTGTGCGC
TCGCCGTGCTCCACGCAATGGCTGCGCTGTGCGACTCCGCCACCGCGGCGGCGGCGACGT
GCGCGGGCCTGGCCCCGGCCAAGCACCGGCCGGAGGTGATATCCATCGCCGACTTCGGCG
GCGTGGGCGACGGGCGGACGCTGAACACGTGGGCGTTCCGCAAGGCCGTGTACCGCATCC
AGCACCAGCGGCGGCGCGGCGGCACGACACTGCACGTGCCGGCCGGCACCTGGCTCGCCG
GCAGCTTCAACCTCACCAGCCACATGACGCTCTTCCTCGCCAGAGGCGCCGTGCTCAAGG
CCACGCAGGTGCGTACTGCGTAGTGCGTACACGTACGCGAGATGCTACGAGCTGAAGCTG
ATGGATCGAGCGAAAAGGCCAATGGCATCGTGTGCAATGCAATGCGTGTTTCGAGTAGTA
GACGGGATGTGTGCTAATACTATGCATCGTGCGTGCATGGGCTGCCGCGCGCGCAGGACA
CGAGGGGATGGCCGCTGGTGGAGCCGCTGCCGTCGTACGGCCGGGGCAGGGAGCTGCCGG
GACCACGGTACGCCAGCTTCATCCACGGCGACGGCCTCCGCGACGTCGTCATTACAGGTG
AGCTGAGCCTCGCCTTCCCTTGCTCGGCTAGGCTTCCTCCTCTGCCTACTGCTCTGTCAA
TATGTTGCTTACTACTGTGTGCCCGTGAGTATCCTCTATCTATCTGGGAGGACAAAAAGG
CTGACATGCACAAGGTCTCCTTCCTCGTCGGAGAAGACTGAATCCTGCACAAACATATAT
TTGCCATACTACTGCCATAGTGCGTACCACGCGTCCACTCCAAAAGGTTCACAAGGCAGC
ACGGGACTTTGTATAAATTACTTACTACTGGATCACAATCACAACACACTTTTGTCAGTG
CTTTGCTTAAACTAAATTCTGAGGCCCATACCATACTCCTAGCTAGATACTAGCTGGTAC
CAGCAGACAGCAGAGCCTGAATTGTTAATTCCATTCACTATCCTCTGGGATCGTGATCTC
GTCCCTTTCCTTGGGCATTGTTAGGTGACAGGGGAGTCATCGACGGGCAGGGTGAGGTGT
GGTGGAACATGTGGAGGAGGCGGACCCTTGAGCACACCAGGCCAAACCTTGTGGAGTTCA
TGCATTCCACCGGCATTCACATCTCCAACATCGTCCTCAAGAACTCACCCTTCTGGAACA
TCCATCCTGTTTACTGCGAGTAAGCGCGCTAACTACTTAGAGAATCTAAAATCCACGGCC
CCAAAATCAATAATCCTGAATCATGATTACATACATTCTACCAAAATAGAGGATTTAAAT
TCCTTCTTTTCAGCAACGTGGTCGTAACCAACATGATGATCTTGGCACCACGTGACTCCC
CAAACACAGACGGAGTCGATCCAGGTATGTAAGCTATTGCATCTGCAGCTCCTGCCCGTG
TCACTCAGTCACACATGCGCGCTCGCTCAAATAAAGAAACCATGGCGGTGCGCATGCAGA
CTCCAGCTCCAACGTGTGCATCGAGGACTCGTACATATCCACCGGCGACGACCTGGTGGC
CATCAAGAGCGGGTGGGACGAGTACGGGATCGCGTACGGGCGGCCGAGCGCCGGCGTCAC
GGTCCGGCGCGTGAGGGGCTCGTCCCCCTTCAGCGGCATCGCCATCGGCAGCGAGGCCTC
CGGCGGGGTGCGCGACGTCCTGGTGGAGGACTGCGCCATCTTCGACAGCGGCTACGGCAT
CCACATCAAGACCAACGTCGGCCGGGGCGGCTACATCCGCAACGTCACCGTCGACGGCGT
GCGCCTGACCGGCGTGCGCAGCGGCGTCCGCATCGCCGGCGACGTCGGCGACCACCCCGA
CGCGCACTTCAGCCAGCTCGCCGTGCCCACGGTCGACGCCGTGCGCATCAGCAACGTGTG

GGGCGTCAACGTCCAGCAGCCCGGGTCACTGGAGGGGATCCGGGCCTCGCCCTTCACCCG
CATATGCCTCTCCAACGTCAAGCTCTTCGGGTGGCGGAGCGACGCCGCCTGGAAGTGCAG
GGACGTGCGCGGCGCTGCGCTCGGGGTGCAGCCGTCGCCCTGCGCCGAGCTCGCCACCAG
CTTCGCGTCCGCACGGTCCTCATGCAGCTAGTTAGTGTAGTGATGCCTAGCTAATTAATT
TCTGAATTCTGATGATGGGTGTGCTTTTAAGTTTGGGCTTTGTTTTACTTATTCTATGGC
ACCGATGTCTGTCTGTCTGTCTTTGCTTGCTCAATCACTTACTTTAGGCTTCCACTGCTA
AAGTGCTAATCGTTCAGAAAACCTTACCACTG
>ZmPG38
ATCGGCCGCGTCCGGCGTCCCGTCCAAAAAGGGAAAGCGATGCGCGTGGCGTGGCGGCAC
TTCCTCCTCCGCCTGCCTCGCCCGCCATGGCCGCGCGCGCCAAGGCCGCCGCCGTCGACG
ACCACGACCCCATGCCGCCACCGCTGCCGCTCCCATGGCCGCGCCCAGCGCCGCTCCTCG
TCATGCTGTCCCTGCTCGCCACGGCCTCCTACCTGGCCCTCACGCGCTTTCCCGCCGCGC
TCCTCCCGATCGCCCCTCGCTCTCGCCCCCACGACGCGCCCGCGCACGCGCCCGGGGACA
GCTGCGCGGGGTTCTACGCGGGCGCGGGGCCGGCGCGCGAGGTGTGGGCGTCCGTCGAGG
AGTTCGGGGCCGTGGGCGACGGGGCCACGCTCAACACCGCGGCGTTCCGGCGCGCCGTCG
CGGAGCTGGGCGCGAGGGCCGTCGGTGGGGGAGGGGCCAGGCTCGACGTGCCCCCCGGGA
GGTGGCTCACGGGCAGCTTCAACCTCACCAGCCGCTTCACCCTCTTCCTGCACCGCGGCG
CGGTCATCCTCGGCTCCCAGGTTAACGGTTCATTCATTTCTTCTTTCTTTCTTTGTTTAG
ACAGTAATTAATACAGTAGTAGCAGTGCCAAATGCTGCATCGGCTTCGCTGTAGGAGCTA
GGATTCTATTCTAGGAGGTGGTTTGAAATTTTGCTCAGCGTTTCTGCTCAAGCATAGCAT
GCAATGCATGCGCACAACAAAACGCGTGGTTTAATTTGCTTGCTCGATCAGTGGCCTGGC
TATGCATGGACAAGAAATTTGTTCGTTACATTGGTGGGTTCAGAAATTTCCAAAGCCTTG
CGTAACCGGCCCTCCAAACATTGGGATGCTCGTCGTCGTCAAACTAGGTAGATTCGGCGC
TTGTGCTGTCACATGCATGCCATGATCCGTGTGCAGACAGATACTAAATGTATTGGAGAA
GTCACATGCTTGGCCATGGACGGTCGCCTGGTTCTCTGCTGCTTCCTTGTGAACTCTGCC
ACCACATTATCTGTGTGCCTTTGCTTTGTCCCCACTGCATGCTATGCTGCTTCCATTCCA
CCTGGACTGGGGTTGCTGGCCCGAGTTGAAGCCTCTTTTGTACTTTACATTGCTTCCACC
TTCCTCTTATCTGTCGTCTGTGCTAAAGCCAATAGAGAGGGTAGCTGTGTCACTTCTCCT
GCTACAGCTACTTCCAATTCCAACAGAACAAACAAGCTAGTATTAGCTACTGCATGGGAA
ATGGTGAGCTTCAGTTGGAACACACTGATCATAATAATCGTAAGCCATCTTGCGTCTTTT
ACAGCTAATGCCTGTTCCAAAATATTGTGAACGCCAGTCTATGCCCATTTTGTTTGTTTG
TTTGTTTTTTCCCATCTGTGTCAACCAAGGCAAAAAGACACAGCTGCATGTGTGATCTGT
ATTACACTACTGTAGAGTCTGTAGTACAGTACTGTAGAGTCATTATCAGGCCATTCATTT
ATAATGGCAACAGGATGGCTTTATAATTGAATTGATACCCACCATGTTAGATATATATAT
TCTGTATTGAATACTTGTGTATTCAATCCACATTTAACTAGGCGAATGATGCCCAGCGCT
TCGATCGTAGCTGTGTACGTATGCTCATTACAAAGCTGTATAGAGTATTTTATTCACTAG
CAAGCTGGACTATCATGTGCAGGATCCAGAAGAGTGGCCTCTCATTACCCCTTTGCCGTC
TTACGGGCGCGGGAGAGAGCGGTTAGGACCACGCCACATCAGCCTCATTCACGGAGAGGG
CTTAAACGATGTTGTTATTACCGGTACGAACGAATTTCTAGCTATATATGTCTACTTTCA
TGATCGGGTAATAAATAAAATATTTTTTTTTTGCCTCATCGCGTTAACAAACATCGTCAG
GTAGCAACGGGACCATAGATGGGCAAGGTCATATGTGGTGGGAGCTATGGCGGAACCGAA
CCTTGAACCACACGAGAGGCCATCTCATCGAGCTTGTGAACTCAACCAACGTCCTGATAT
CCAGTGTCACGCTGAGCAACTCCCCTTTCTGGACAGTACACCCGGTCTACTGCAGGTCAG
TTCTAGCGAGCTGGTTCGATCCGTTGCATTTCCTGCTAGAGGAACATTTCCATTCTATTT

CTATGTAAATGAATGAAGTGCTGGGAAAGTGATTTTTTTTATTTTTGATATTCGTTTGTC
AGCAACGTGGTGATGAAGGATTTGACCATACTGGCTCCCCTGGATGCTCCAAACACAGAT
GGCATTGATCCAGGTCTCTGCGCTAGCTGTTACTTTCATTACCATTAACAACTCGATCTC
TACTGACAGCTTCTCTCGCTGATTGGAACAATAATAATGGCAGACTCGAGCTCTGAAGTT
TGCATCGAAGACTGCTACATCGAAAGCGGAGACGACCTCGTGGCCGTCAAGAGTGGCTGG
GACCAGTACGGGATCTCATTTGGCAAGCCGAGCACGAACATCGTCATCCAGAGGGTTTCG
GGCACGACTCCGACGTGCTCGGGCGTAGGCTTCGGCAGCGAGATGTCAGGAGGCATATCT
AACGTGCTCGTCCGTGACCTCCACGTGTGGAATTCGGCGCAGGCCGTGAGGCTCAAGACC
GACGTAGGGAGGGGTGGCTACATCACCAACATCACCATAGCCAGTGTGGCCATGGAGAAG
GTCAAGGTGCCGATAAGGTTCAGCCGAGGCGCAGATGACCATCCTGACGACAGGTATGAC
CCGGCCGCGCTGCCGAGGATCAGCAACGTGCTGGTCAGCGACGTCGTCGGTGTACACCTG
CAGCGAGCACCGATGCTGGAGGCAGTGCCTGGCGCAGTTTATGAGGGGATCTGCTTCAGG
AACTTCAGCTTCAGAGGGATAAGGCGGCAGCAGGACAGCAGGTGGCATTGCGAGTCTGTG
TATGGAGAGGCGCATGACGTTTTCCCTGCACCGTGTGAAGAGTTCAGGAGAGATGGATCC
TCCTCCTGGTGTGGACTTCTTTGACCAACTACTCTATCTAGTAGTAGTACATACTACAGC
GCCCACAAGTTTTGGGAGTGGATGATATAGGTTTTTTAGATTATACAGACAAACTCAATT
TTTTAAACTTTAA
>ZmPG19
ATGAATGGCGGGCGGGCCCTGATCGCCACCGCTGGAGATCTTGTGGTGACGGACGGTGGT
GCGACTCCGACTCCCATGCGCCTGCGCTGTGGGTTTTTGGTTCCGGTTCAGCCTGGTTTC
GGGAGGGGGAGCCTTGCACATGCACTCTCGAGTGGTTTCATCTTCCTAATCAAATGCGCC
GTAGATAGAAAGATCGCTGACTCTTGCAATGCCGCTCTGCTCCGCAGGTCGTCCTGGCAT
TGGTGCTGGCCGCCGCGCTCGGCCCGGCCACGGCCCCGGCCCCGGCGCGCGCCCAGCTGC
GGCGGGACCACCAGGCGCCGCCGGGCCCGCGCCCGCACAGCGTCACCATCACCGAGTTCG
GCGCCGTCGGGGACGGCAGGACGCTCAACACGGTCCCCTTCCAGAACGCCGTCTTCTACG
TCCGCTCCTTCGCCGACAAGGGCGGCGCGCAGCTGTACGTGCCCAGGGGCCGCTGGCTCA
CCGGCAGCTTCAACCTCACCAGCCACCTCACCCTCTACCTGGAGGAAGGCGCCGTCATCG
TCGGCGCAAAGGTAATGATGATGCCCCCCGCACTGGGCGGGGCCTTTTCCATTCCGTTTC
CTGTCACGGGTTGTTAAGGCAGTGGTAGTTACAGAGATTCTGCTCGCGTTGGCATTCAGA
GCGAGTGTTTGGTTGCATGTGTGTGAGGGTACCTATCCTTTTCAACCTGCATTGGCTGAA
TGTGAGCTGTTAGGATTGGTATTGCTGATCTGATCATCCTAGTTTGTTTAGATATGGTTG
TTGGATGTACCTCAGGAAGGAGTGATTTTGACATCTGCGAGGTGAAGTCACATGCATGCC
ATGGATTTGGACGCCATCTTTCTTTCTCTTATGCTCTTCGTTACTGCTTTATATGTTGGG
GAAAAAAATCCTTTGCAGATATTATGATAACAAGTTCTGTTCTAGATGACGCTTGTAGTG
GTTTCTGTTTGCTTTTACTGTATGGTGTTTTTAGGAGGTGGAACATAGCTATTGCTGCCA
GGGAGGTAGCCAAGAATATCACATAGTTTTGAAGTCAAGCTTACCTCATTAATCTTGTAC
CAAGAAAAGGCTAGGCATGTTTCTTAGGTCCTGCCATGGAGATTGTGGTCACTTCTCTGT
TCTCTATTCAACCATTTTCTCTTTTGACATGCACAACTCTTTACAAACGTAGACTGACGT
AAGTATCTTATCTTTGTATTGGGATGTAAAAAGGAGCAATGCCATGATCTTCATATACAA
AGATTTCTGAATTTTTAATTATTATTAAAGGTGCTGACATAAGTATCCTTTCTTTGTTTT
CCTGCACAATGTAGGACTCATCACAATGGCTGATCGTTGAACCTTTGCCATCTTATGGCC
AAGGGCTAGACCTTCCTGGTCCTAGACATCAAAGCTTGATCAGCGGATACAACTTAACTG
ATGTTGTCATAACTGGTAAGTGCTTTCTTCTATCTTGCATATGGGTTTTCCAGTTCTGCT
GGGGGTTCTTTAGTAAAAAAATTGATGGCCCTTGCATGTCGTATGTCCACATATTCCAGG

GAACAATGGAGTTATTGATGGCCAGGGCTTGGTATGGTGGCAGTGGTTGCGCTCCCATGA
GCTGAACCATAGCCGACCCAACCTTCTGGAGTTCCTGTATTCTGAAGATATTGTCATCTC
AAACTTGACATTCTTAAATTCACCAGCCTGGAGCATACACCCGGTGTACTGCAGGTGCAG
CGCAATTAACTTAGCTTTGTGTGTAAGGTGTGGTTTGGTTGATATTAGTTGCATCTGTAC
TAAAGCATAGTGTCTTTGTATTCTCTAGTAATGTAAAGGTCCACAATGTGACGATTAAGA
CCTCATTGGACGCTCCACTGACTGATGGCATAGTTCCAGGTATGATTTTCCCCCCCTCTA
GGCTGAATACACTTTTTATGTTTTATTTTCATATAGCATAGATATTTGAATTCATTCTTG
CTGGTAGGATGGTAGGACTTAATGACTTAGTGCTGAATGCAGCAAAAAAAACATCTTGAT
CCCTGAAGAAAACTGATTCTCCTTTCCCCTTTTCCTGCTGTACTATGGTATCATCCAAGC
CTGGATAATAATATGCCCTAACTGTTCCTCCATAGGCTTAACAAAGAATCTCAACTACTC
ACACTTTGTAACTCTTAAATGCACTGGACAGCGATGTCGAGAGGGTTTTACTAATTATTC
ATTCTTGCTCCAGATTCATGTTCAAACTTATGCATTGAGGACAGCACCATAAGTGTCAGC
CATGAAGCCATCTCCCTGAAAAGCGGGTGGGACAGGTATGGCATTTCCTTCGGGAGGCCT
ACCTCCGACATTCGCATCAGCAGAGTGGACCTGCTATCATCTTCCGGAGCCGCTCTTGCA
TTCGGGAGTGAGATGTCTGGTGGTATCTCAGACATTCATGTTAACCACCTCCGGATCCAC
GACTCCTCCAAAGGTATTTCTTTCAAGACCTCACCCGGCCGTGGGGGTTACATCGAGGAC
GTGGTCATCTCGGAGGTACAGATGGAAAATGTGCATGTCGGCATCGAGTTCACCGGCAAC
TGCTCGACCCACCCAGATGACAGCTTCGATCCGTCCGACCTCCCGGCGATCGACCATGTC
ACCATGAAGAACATGGCCGGCACGAACATCTCGGTCGCCGGAGTTTTGTCGGGAATCGAA
GGAGCCCCATTCACAGCCATCTGCCTTTCCAACCTTAACTTCTCTATGGCTGCCGGTTCT
GGTCCGAGTTCCTGGTCTTGCTCCGACGTTTCCGGCTACTCCGAGGCGGTCTTTCCTGAA
CCCTGCACAGAGCTCCGTGATCCGTCCTCCAGCTCTTCCGTCTGCTATTCCCTGGCCAGC
TACAGCGCCATTGAAACAGCATAAATGTCCATCCATCCTTCCATCCATTTCTTGTTCTCC
CCTTGAGCCTGCATCGCCGCCATCTTTTGTCTCAGTTCTTTTTTTTCCAATACACACAGA
CAGACCAGGAAAAAGAACAACATCACTACTCTCATGGCCCTGAGGCTCGGATCATAAGCT
GCATGAATGATGACCTAGCTTGAAGGGGAAGAAGAATCAAGTGGGGGGTTGTGGACAGCT
AGGTGCTTGCTTGTACAGCTACATTTCTTTATTCAATCTGTAAGAAGGAAGGATTTTTTT
TTGGTTTGGTAGGTGTTGAGCGCAGAACAGTTGCTTCATACTATTGGCATGTGGGTATAA
ATTATCAAACAGCATGATTAAATTATACGTGTATATTCCATCCCCCCTGTTGAATGATTG
ATTGAGAAGGCATCATCATC
>ZmPG54
ATGGCTCCGTGCTGGTTGCAGGTGGCTCTGGCATCGATGGTGGCAGTTCTGGTACTCGGC
CTCGCCTGTGCAGAGGCGGAGCCACAGTGCACGCGGCGCCGCCCGCCCGCGCCGCCGCGG
CCGCACAGCGTGACGATCACCGAGTTCGGGGCCGTTGGTGACGGCGTCACCCTCAACACC
GTGTCCTTCCAGAACGCCGTCTTCTACCTGCGCTCCTTCGCCGACAAGGGCGGCGCGCAG
CTGTACATTCCCAGGGGCCGGTGGCTCACCGGCAGTTTCAATCTCACCAGCCACCTTACC
CTCTTCCTGGAGAGTGGCGCTGTCATCGTTGGCACGCAGGTTGTTGTCCACCTCAATTTT
GCAATTGTTTTCTCTCTCTTCTGTCCGCGTGAGCATTTTGCTGTGTCATTACTTGTCCAA
TTCCGTTCCGTAGAGCATAGGCAGCTGGGTGTTTAGGATGTTGTTTGGCCATCAATTTTC
GATTTGCATTTGCATTTGCATTGAGAGTTGCTAGCAACGCAATGGCCAGCCCATGCCACC
ATTTGTTTGTTTGGATACGGTTGGTGGAAGATGTCCTACTGGAGCGATATTTTTCCAGCC
TTCCTGGTAAACCTCATAGGTGAGGCTTCTATTTCTAGGATTTAATCTGAAAATATTTTG
CTTGGGAGTGAGGTATATGTTTTGTTCTTCCATATTTTCGTTAAGTACTACAACGTTCCA
TATTCTTTCGGATCTTTGCTAGCGTAATGGTATGCGACTTTGCTAATCAAGACAGGAGCA

CTAGATGATGGTGACACGTAGTTATCGATGTGAGGGGCAGTAGCTAAGGAAAGACAAATA
CCATTTGCATTGATTTGAAGTCAAGCTGCTTTATGCACTGGGCTGATTGAGATTGTTTGT
CTGTAATATATAGTTTTCTGCATGGTGAGGACATGTCTAGTATCTGTTCCATTGCATGCT
GGTGAAGTTTTGTTTGTTCGGAAAGAGCAGTCTAAGATCTTTAATAGGCGTTGCAAATCT
TTGAACATTGAAAGGAACATATTTTGTATGCATTTCACTTTTTCTCCTTATAGCTGTGAA
ATTAGATGCCTTATCGTAAGTTAAACATTTAAACTAACTGTAAGCACTTGTGTTTTTACT
TCAGGACGTTTCACAGTGGCCTGTTGTCGAACCTTTGCCATCATATGGACGAGGAATGGA
CCTTCCAGGTTCGAGGCATCGAAGCTTAATAAACGGGCAGAATCTAGTTGATGTTGTAAT
TACGGGTAAATGAAATCTTAAAGCACGCTTGGTTTGAGACATATCAGTATTTTATCTAGT
AGTTATTTGATTTACATACTTAAAGAACACACTTCTTTCAGGAAACAATGGAACAATCGA
TGGCCAGGGTTCTACATGGTGGAACTGGTTTCGTTCGAATAAGTTGAACTATAGTCGCCC
TCATCTTGTGGAGTTTGTGGACTCTGAGCAAATCGTCATTTCGAACATAACATTTTTAAA
TTCCCCTGCTTGGAGTATACATCCAGTGTATTGCAGGTACGCTAACATTGTTTGCAACTA
TAGAATCTGCTCTGCTCTGTCCTGCTAGATTTTTGAGACAGAGATTGTCAAAAATTACTT
ACTGACATGTTTCCGTTGTTTTCCATCAGCAATGTAGTGGTCCATAGTGTCACAATTCAG
ACTTCATTGGATGCTCCACTTAATCATGGAATCGTTCCAGGTAACGCAGCACTTGATCAT
CATGCATTCCTTACCTTAGTCAAAATCCATGTGACCGGAACATTTTTTTGGATATGTATC
CTGAGTATCATCTACTTAAAGTATGTCTTAACTGAGACAAGTTATACTAGCCAGTTTCAG
TTGTGGCTGTGTACTTGGTGGAGACAGCAAATTATTCTATGCTCTACGTTTAACATTTTA
ACCCATCACCTGAGGTGATTCGCCGCAGAACCATGGCCAAAACCTGATCCATTCCTCTCT
GCGTATGTGTTTTTTTTTCTGTAGATTCATGCTCAAATATGTGCATCGAGGACAGCAGTA
TCAGTGTTAGCCACGACGCCATATCACTAAAAAGCGGGTGGGACAAGCAAGGCATTGCTT
TCGGGAGGCCAACCTCAGACATCCACATAAGCAGACTGGATCTGCAGTCCTCACTGGGCG
CCGCTCTCGCGTTCGGGAGCGAGATGTCGGGCGGGATCTCGGACATCCATGCGGACCGCC
TCCTCATCCACTCTTCGTCCAGAGGCGTCTTCTTCAAGACCGCGCCGGGACGCGGAGGCT
ATATCAGAGACACCGTGATCTCAGACGTCCAGATGGAAGACGTCGACGTCGCCATCGCAT
TCACCGGTGACTGGCCAAGCCACCCAGATGACCAGTTCGACCCGGCCGCGCTCCCGGTGG
TCAGCCACATCACCCTGAAGAACATGACTGGAACAAGAATCTCGGTCGCTGGCGTTTTGT
CAGGAATCGCCGGCGACCCGTTCACCGACATCTGCCTCTCCAACATCAACTTCTCACTGG
CCGATTCGGCCAGTCCCACCTCCCACTGGTCTTGCTCCAACGTCTCCGGATACTCCGAGC
TGGTCTTCCCCGAGCCTTGCCTGGATCTGCAGACCCAATCCTCAGACGCTTCCGTCTGCT
CCACCCTCCCTAGCTTCCACGCCGCCGCCATTGCAGCAGCGTAAATGCACAGAGAGTACA
AAGTGTCCATCCTTTTTCCTGTTCCCGGTCCCCTGTACACTTCAGAGGATCTCTCAAGCC
GCCTTTTCCTCCCCCGACAATTCTTTGAAGCTATAGGCGCGCCCCTGTCGTTGAAGAAAA
GAATCGTTCACCGTTTTTCTCCGGCCGTCGGCTTCGAGGGCTCAGCTGCATCGCCTTTCT
TGCGGGAGGCAAGCCTGGCGGCGGACGGTCGACATCGAGCTGTTAATTTGCTTGTACAGT
TATGATTTCTTCATTCAATCTGTAAGAAGGAATTTTTGTGGTAGTAGAGGGTTGAGTCGA
GTCCAGAGAGTTGCTTCATACGATTGTCGATTTGGTGAATAAAACACATGATGAATTCAT
GATCCAGTGGCTCTGTATACATCCTGTCATCCTTGTGGGTACATCCATCCATGATCCATC
CAATCTAAC
>ZmPG1
AGGTTTATGATTCGGCGGCCGTTGTGAGGAGCGCGGCTGTGCGGGCATGCCACCGGGGCC
GGCCGGTCCCTCCCTCCTCCGCTGGGCTCGCGCGGGAGGAGAGGGGGAGGCCCTGCTCCT
CCGGTCGTGGTCCTGGTCCTGGTCCTCCTTCACGGCCGGGCGGGCGCCACCCACCATCAC

GCACACTCATTCACGGCGGCGGCAGCAGTAGCAGCAGCAGCCAACCACCGTCCCGTCCAG
TCCAGGTGCAGCAGTGGCAGGAGCAGGGGGTGCCGCGACGGGGGCGCAGCCGGGCCCCAG
CGCGGTCAGACAGCCACGCCGCCTCTGCGCTGCACTGCACAGCGTGGCATGATTGGGTCC
CTGGCCCCCCCTTTCCCCCCGCACTGCTCTCTGCCTCAATGCCCGCCCCAGCCTCCCACC
CTCTCACTCCAGTCAGTCCCTCCCAGCGGCTCGCCAGCCACCTGCTCCTCCAGTCCTCTC
TCCATCCCTCTCCGGCTCTCCGCCTCTCCCCCTCGTGAGGAGGTGAGGAGAGGAGAAGCA
ACAGAGGGGGCCGCGGCGACCGACCGACCGACCGACGTGTCCTGAGTCCTGGTGCTGGTG
GTGGGTGCTTCTCGTTCCTGGTACGTTCCTGCCCTCGCGCCACTGTTCGTTACAGTTACA
TTTACAGCCCCCGCTCTTTCTCTTCCTGATTGATTAACTGGTTGATGGGCCTGCTGCCGG
CTCGTCGGTGTTGCAGGCGGGTCCATACACGGCGGGGTGCTAGCTAAGGGAGAGGTGCCG
GCGTCGGCGTCGGCTGCGAGGAGCAGGAGCAGCAGGCCACCGACGGATTCGGAGATCTCG
CCGGAGAAGATGAAGAGATCTGCATCTGTGAGTGCCCCTCCCTACTGTCCGTGCCCGTGC
CGCGCTCCTCGTCTCCCGGGATCGCGGGAGGCCCAAGGGACGATGGATCGTGCCGTTACC
CGTCACGGGGTTCCGGTGAGCAGTGCCCGACCGACCCGTTGCCCTTTCTGCTGCATCTCA
ATACCGCCGTTGGCCCGAGGAGATTGGCGGACGGCCTCACGCGCTGCTGCCGTCCTAGAT
CGATCCTGCATTCCTGCGTGGCGCCTGCTGGCATTGCCGCGTGATCTGCCGTTTGTTTTC
CCCTGTCACTTGATCGAATATACATATTTATTTCTTTTTGCAAAACCGGTCCGTGTTCCA
AACAATTGGTATATCGCCTTAGTTGAAGGTGTCACATTGATCCACAATTCAATAAAGCAA
TTTTGAAGATAGGTAACACGAGAGGAAACTAAATGCGTTGGCCCTCTCCAGCTTTGTGTG
CCATGTTCAGATGCTATAAAAACCATCTCGACGAAGATTCAGTGAAACTTTTTTTCCAGA
TGGATATAATTCCGTTCAGTTTCATTGCAAAGTTCTTCACGTTACATGTTTTGCTGGTGA
CGCAAATTACAAGCACAGATATACTACATGTGATTGTTACTGAATCTCCATTTTCTTTTT
TTTTCCTTTGGAGTTTCTGCCAGCTGTTTCAAGTCCTTCTGGTTTTCACAGCCGTGATTG
AGATCCAATGGTCCACTGTATCCAGCATTTACTGCAAGGACATGCCGCCGAACGTGTATC
GGCCTCACAGTGTCACAATAACTGAATTTGGTGCCGTTGGGGATGGTGTCACCCTCAATA
CAAAGGCATTCCAGAATGCAATCTTCTATCTCAATTCATTTGCGGACAAGGGTGGCGCGC
AGCTTTTCGTGCCTGCGGGAAGGTGGTTGACGGGGAGTTTTCATCTTATCAGCCATCTCA
CTTTATCACTGGACAAGGATGCAATAATAATTGGATCTCCGGTAAGATGATTTGCAGAGT
TTGCACTATGATAATCTGTACTCCAATATATTCTTTGGATCGTATGCTTGTTATGTTATG
CCCATGAACTAGTCAAACTTATATTGCTGAATCATGTTATCAATCTAGCTTTCTTCTAGG
AAACAATACAAAGCCAACTGCATATGTTATATCTATTACTAAAAGGACAGATCCAGTGCT
GACAGCTCCCACACTTGTGGTCTGGGAAAGGGATAACCTAGTCAAACCTTGCTCCTGCAT
TTTGCGGAGAGGCTGCATCGAACCCAAGACCTTTTTAGCTCAGTGGGAAGTCTTTAGATG
CTAAAAAAACCACATTCCTTAAAGGTTCAAAACCCTTTTGTTTTTCCGCGTTGATCCTGG
TTATACTTCCATGGTAATGCCTTGAATGCATCCTGCAGGATTCATCTCATTGGCCAGTTA
TCGATCCTCTTCCATCCTATGGTCGAGGTAGAGACCTTCCTGGAAAAAGACATCAGAGTC
TGATTTTTGGATTAAATCTGACGGATGTAATTATAACTGGTTAGTAAATACTATAGTTGT
GTCGCCCCCATCTTCTTCAAGTATCATTTAGTTAGCTTGTAAGTATTCTAAATGTTGCTA
CTCTTGGGCAGGTGCTAATGGTTCAATTGATGGTCAAGGAGCTATTTGGTGGGGCTGGTT
CCACAACCACACATTGAATTATACTAGACCACATCTTGTGGAGTTGATGTATTCTACCAA
TGTCGTCATATCTAATCTAACCTTCAAGAACTCGCCGTTTTGGAATATCCACCCTGTGTA
CTGCAGGTTGGTTTTCTAGATAGCCACTTGCTTTCTGTTCAAACATAAAAAAAAATTCAT
TCACAATACAAATATATTATCTGGAGTGCTTTTGCTACATAAACAGCCAAGTGCTTGTCC
GGCATGTCACAATCCTAGCGCCTTTGAATTCGCCAAACACCGACGGCGTTACTCCAGGTA

AGATGCCACTTTTAATTATTCGAGATTTCTAAGCTTTCTTAATGTTTTACATGCTTGCTC
CTTTCCCTCCATTTTGTTCTGCACTAGGGAACTTTTGTTATTAAACACACTAGGGAAACA
TTGGTGGCTGATATAATTTGGCATGTTTTCCCTCCAAGAATTTTTACTCTTTGTGCTTTC
CAGATTCATTTACTTTTGATTTGTGTAGTGTAGCTGAAATATCTGAAATATGCAATGCAA
GCAATTCTTTTGTGAGGTGACGGCATACTTTTAGGTTTCCGATTGCTGTTACGCAATGTT
AAAAATAGTAATAACATAAGCATGAACTATCATAAGATGTGACTGGTCTTTGCTTGAAAT
GTGACCTTATCCCAGTCCTCCAAATCTATGGTACACCAATAGTTAAGAGGTTCATTGAAA
AACATGTTTGTTTTTTTTAACTTATGTTGGAATTGCATAGCCATCAATCATATTTGCATC
TATGGTGCTAATACAACTCCAAACCCATCCAGACTCTTCCACGAATGTGTGTATCAATCA
TTGCTATGTAAGAAATGGCGGTGATGTTATTGTCATTAAAAGTGGCTGGGATGAATATGG
CATTTCATTTGCTCAACCAAGCTCCAATATCAGCATAAGCGACATCACAGGGGAGACAAG
GGGTGGTTCAGGGATTGCCTTTGGAAGTGAGATGTCAGGTGGCATATCGGAAGTACGGGC
TGTGGGCCTCCGCATTGTCAACTCCCTTCACGGGATCAGAATCAAGACGGCCCCGGGGCG
CGGAGGGTACGTGGAGAATGTGTACATAGCAGATGTGAGCATGGACAATGTTTCGATGGC
CATCAGGATCACTGGAAACTATGGTGAACATCCTGATGACAAATATGACAGCACTGCTCT
CCCTGTGATAAGCAATATTACAATCAAGGATGTTGTTGGCGTTAACATTGGGGTTGCTGG
TATATTGGAAGGCATTCAGGGGGACAATTTCAGCAACATTTGTCTGTCCAATGTTTCCCT
CAGTGTGCAATCTGCACATCCATGGAATTGTTCACTTATTGAAGGGTATTCGAACTCGGT
GATCCCAGAATCATGCGAACAGCTCAGAAGCAATTGTAGACAGACATCCATTTGCTACGA
TGGAAGCAGTTCTTCAGTAATGAGTGTGCAGCAACCAAGACATACATCGCCCACCAGCCG
GTTGCTAGATCTTTTTACTGGAGTTGGCTTCATTTTAGTGTAGGTAGAGCTGTGCTTGTT
CTTGCTGCAAAGGGGGCTTGCTTCATGTAGTGAGAGCCCTATTAGTCATGTATATCTGAT
TCTTGCAATTATTTGTGATTAGAGTGCCACTTGCTTCATGTATATAGCTGTTGAGATGAC
TGTCGGTGTATCTTTGCCTGTTTTGTTCCAATTTTTGGTATGTGAATGCAATCTGCATAA
CTTTATGGTTTTGAACACACTCCCTCTGTTTCTTATCATCTG
>ZmPG42
TTTCGTTCTGGACGCTGCTTGCTTCCACAGCTCCACCGTCATCCATTGCATCGCGTAATC
CTGCAGGTGCAGCTACCGGCATCCTATCGCGGCTTCGCTTCGAGACGGAAGGCTGTCCCG
CGAGGTGTGGAGCCGAGAGCGGTTCGCCGTCCGAGAAGGGTTCACATCACCTCTTCCTCT
GCCCCCCTCCGGTGAGCGAAACTCGACGGTTCCTCTGCTCTCAGTTCGCCGTTCCCCTGA
CACGCTCTTCGCTGGTCTTGTGGCGCTAATAACCTGCAGGTCTGTCTTCGGGGTCTCCCA
TTGGTGGCTGCAGTTGTTTTGATGGGAACTCAGTAACCGCTCGAGAGGAGAGGAAAGGAG
CGGATGCTCAACTGTAGCAGGAAGATGGGTAGATCCGCGAGTGTAAGGAGACCGTATTTC
TTGTTGGGTATCTGGATTGGGGTTGCGATTCAGTTTTTGGTGTTTCCTCGCGAATTGCTC
CGCCGTCAGCGCCTTTCCTGAATGGGGGTCAATTTGGCTCTCGGGGTGCTCGGATGTAGA
GCCAGAGATTTTTTTTGGGGGAAAGAATCAAATGGGCATATTCACGCAGATCATTAAGAA
TGGTAGAAACTTCAATTCTAAGGCTACTGACGCGGACACTTTGAATTTCGCGCAGGTTCT
TCAAGTCCTTCTGGTTTCCGCAACCATCGTTGTTGCTCAGACGCAATGGGCTAGTGGTGT
ATGGGGCATGTACTGCGAGGACTTGACAGCGAGTGTAGAAAGGCCGCACAGGGCCTCGGT
AACCGATTTCGGCGCCGTCGGAGATGGTGCGACTCTTAACACGAAGGCGTTTCAGAATGC
GCTCTTCCACCTCGACTCATTCGCGAAGAAGGGCGGGGCGCAGCTGTTCGTGCCTGCTGG
GAGGTGGTTGACTGGGAGTTTTAGCCTGATCAGCCATCTCACGCTATCGCTGGACAAGGA
CGCAGTGATTCTTGGATCACCAGTAAGATGATTTCACAAATGGTTCCGTAGGCAATAATA
ATTCACCTACTTTAAGTAGAGTTGCTTCTGTTTTGCCTCGTCGCTGTGGCTTTAGACCGT

TGTCTTTTTCTTTTCGTACAGTTCTCACGTATTGAAACTAAGAGAATGTATAATCGCCTG
TACATCTTGACAATAAATAGTACCAATAATGCCTCATCACAAGTTTACTGTAAGTGCATC
TCATGTTTTGATGAGTAAGTTTCTACTGAAACAACAGTGCAGCCGTTTGGTTATGGTGTT
CTTATGAGGATATTTGCAGGACTCCTCTGACTGGCCAGTGATTGATGCTCTTCCATCTTA
TGGGCGCGGTAGAGAACTCCCCGGCAAAAGACATCAAAGTCTAATATTTGGATCCAATCT
TACCGATGTGATAATAACCGGTAAGCATTTATAGTTGCATCGAGCACTTCATACGGTTTT
CCTTTTGGTCTTCTGGTGTGTAACAAAAACAAACTACTGATCTGCCTCTTTTCAGGTGCC
AATGGCACCGTCGATGGCCAGGGTGCGGTTTGGTGGGACTGGTTTCACAACCACACATTG
AACTATACTAGACCACCGCTCGTTGAGTTGATGTACTCTACCAGAGTTGTTATTTCCAAC
CTGACCTTCATAAATTCGCCGTTCTGGAATATCCATCCTGTGTACTGCAGGTTCCTTTCG
AGGATTTCCTCTTGTCATTTATTACCGAAGCACCGTTTCAATCGTGATGATAATAATTAT
CCTTTGTACCGTATTTTTTCCCCCGAATGAACAGCCAGGTTTTGGTCCAGCATCTCACAA
TCCTAGCACCTATCAGTTCACCAAACACCGACGGCATCGATCCAGGTGTGCGTTCTTGGG
CTTGCTATGGCTCACTGAATTCTTTATTTAAATGGATAGTTGCTTTAGTTTATGTGCTGT
TGGCCTGTTTGCTCCATTTAGCAGTTGGCCCTGCACTTTGTAAAAATAAAAATCAGTATC
ATGAACAATCGCAACGCATGACTGTGTTCTGGCCTGAAAGAAAGAAACTAACACTAGGAT
GATTTGCATGTCAGTACTCGAGACAGGCATCATATATGTGCTTGAATGTATCTTTGTTTC
TTTCCTTCTTATATATAAAAACGGTAGACCTAGTATCTGTGGGGTCTAGGGAAGTGATAA
GCCAAGGTAATCCCCCCACCTCCAAAAACAATGCGGAGAGGCTACTTCGAACCTTGACTC
GGTTACTCAGTGGGATAGCTATCATAATTGCTCTGAGCTTGCCCTTCTTTTCTCTGATAT
ATATATGTCCTAATTAAGTGAATGACCATAGTTATTGCATAAAGTTCAAAAACTTCAAAA
TGTTATTTCTTGATGTATGGCACCATGACACCAGTATTTCCTGTCAGACACATTTCATGT
ATGTCATGAAGTCCTGAACTGTGTGCTTTCCTTTATGATCTCCTTTTTCGTTTTCTCCGT
CGCATGTGTGGTACTAATGCAGCTTGAAACCCCATCATCCAGACTCGTCCACAAACGTCT
GCATCGAAGACTGCTACGTCAGAAACGGCGACGACATTATCGTCATCAAGAGCGGGTGGG
ACGAGTACGGCATCTCCTTCGCCCACCCCAGCTCCAACATCAGCATCCGCAACATCACAG
GACAGACGAGGAACAGCGCCGGGCTCGCCTTTGGAAGCGAGATGTCAGGCGGCATATCGG
ACGTCCGAGCGGAGGGTGTCCGGATCGTCAACTCGGTGCACGGTATCCGCATCAAGACGG
CCCCAGGGCGTGGAGGGTACGTGAAGAACGTGTATGTAGCTGACGTCAGCTTCGACAACG
TTTCCATAGCCATCAGGATCACTGGAAACTATGGTGAACACCCTGACGATGGCTACGACA
GGAACGCGCTTCCCACCATAAGCAACATAACCATCAAGGATGTCGTGGGTGTCAACATTG
GTGTTGCAGGAATGCTGCAGGGCATTCCGGGGGATAGCTTCAGTGGCATCTGCTTGTCAA
ATGTTTCTCTAAGTGTCAGGTCTACTGATCCATGGAATTGTTCACTTGTCGAAGGGTATT
CGAGCTCTGTGTCACCTGAGGTGTGTGAGCAACTGAGAGCCACTCCTGGTCCTGGGTCCG
GACAGATGTGCTATGGAGGTAACTATCCGGCAGCTGCGGCACAACCGCAGCCGCCGCAGA
AGTCAGGTGCTAGCCGGCTGGTAAGTCCTTTCCTCTCGATAAGTTGGTTTCTACGTAGGT
AACCTTGAGCATAGTATCTACTTATCATTTTCCCCCCCTGTTCCCACTTCCCAGCAGCTT
GGTAATTGTAGTTTATGGAGTTCAGTACCATGTACACTTGTTCCAAAAACTATTTGCTGG
GGCATGTAGCTACTGATTATTCTTACTCAGAAATGGAATGGATTTGTTTGACTTGAACAG
TTCTGTTGAAACACGTCAGGTTCTGGGCTGGCTTGTCCTTGTAAGATACGGGTGCCGGCA
TATAACTTGGTTTCTCCTCGCAAACGAACTTCCAGGGTTATTCAACAAAGGCTAAATTGC
ATTACAAGACCGTGTAAAAATAATTTTGTTTTCTACCGTAAGTACCTCTGCTGCA
>ZmPG7
AGTCACACCACCTATATATCCTCCTCCTCCTCCATGACTACTCTCTGCTGTGCTGCGCAG

GTGCAAGTGCGACCCAACCCAGCCGGGAAAGGAAAGAGGAAAGGGAGGGAGGGAGGGGAC
CGTTGCAGTTGCAGCCATTGATGCGCCAGCAGTAGCGCCAGCAGAGCGGAGCGGAGCGGC
ACCGGCCTGCAGCTCAGCGCCAGGCTCAAGAGACCGATAGGGCAAGGGGGTTAAGCATGG
AGCCTGCGGCGGGACGGAGAGCGAGATGTCTGCTGTTGCCTCTACTTGTGGGGGCTCTCG
CCCTCGCATTGGCCGGCGCTGCGACTGCGACCGCGTGGCCTCACCCTCACGGCGGCGCCG
CCGGTCTTGGACATGGCGCTGGCGCCGCAGCCGCGGGTGCGGGCAGGCCGGGCGGCGCCG
AGCGCTGGTACAGGGATCTCGCGCTCAGGAGGATGGAGAGCGTCAGATCCTCCTTCGGGG
CCAGAAGAGATTTGGCGACGGTGCGTCTTTCTTCGTCGCACGCTTTTCTTTCCTCCAGCT
TTCGCTTCTTTGTTCTTGCTTGTCACTCGAGTGCGGGGTGTTCGTTGCTCTTTGCTCCTG
CCCTCTTTTTTTAAAATAAAAAAAAACATAATATATTGTTTTTTAATCCATCCTACGGTC
CCGTCCTCTTTTCCCTTTACAGTAATGAGTTCTTTAATGGCCAGCTCGTAGTAGTCTCGT
AGTACACAGACACCTGGCTGGCTCTGATCATGCTCTTGGGAGGGTGTATGTACGGTACGT
GCACCAGCTAATTAGAGATACACGGACAGATGCGTCGGTTTTGCTTTGCAGCTAAGCTTC
CACTACCACTACTGCTGCTCCTGCCAGTACAATATCTCTCGCATTTTGCAAGAAATGTCA
CGGCGTTTAGCAGTCACCAACCAAGGTTGGAGCTTTGCGATTAAAAGTAACAAACAAAAC
CAACCCCCCACCGTCTCGTTTTCCGCCGGCCGTCACTGTCGATGCTCAGAGGATCCAATC
ATCCGAGCCTTCCTGGAGAAACATGCTGAAAACGACGTCAGGCTCGTCGCCTCGTCGGTC
AGCCAAGCCAACCACCACCACCACCCATAGGCCATAGGGCACGAGTCAATAGGCGAGCCG
TTTCACCGCCGTTGCATCAGGTTCTTGACCGCAGCAAAACTACCCCCGCCTGCATTTTCG
TCAAACCAAACTTAGTACGGCATTGCCTCCCAGTTTCCCCATCGCCAGCACCAGGGCCAG
TGCCACGCCAACCACCGCTACCTGCTCCGCGCCAAACGCCAGTTGCTTTGCAGCTACTCG
TGCAGCCGTGCCCGCCGCACACGGTTGGCGCCAGTCTTAAGTTCACCGGCAAAGCTTGTT
TCTTTTTTTTTTTTCTTTTCTACCCTTGGGAAGTCTGAACTGTACTGCCACGTCTGTCTG
ACCTGGCGTGGATGGACGGCGCAGGCTTCCGCGAGCGCGCGGGTGTACCACGTGACGGAC
TACGGCGCGGACCCGACCGGCGCCGCCGACGCCACGGCGGCGATCAGCAAGGCCATAGCT
GACGCGTTCCGCCCGCCCACCAACGCCACCATGACCGGCGGCATCCCCGACCTCGGCGGC
GCCGAGGTGCACCTCGACGGCGGCACGTACCTCATCAAGGGGCCCCTCACGCTGCCGGCA
TCCGGCGGCGGCAACTTCAAGGTGCCGATCATCCTCCTCGTTTTTGCTTGTTTGTATACT
ATAGTAATCATGAGGTTCCCTGTTGCTGCCTGGCCCTTGCACCGCTTAGTGACACGTACG
TTGCGTGGCGCAGATCCACAGCGGGTCGCTGCGCGCGTCCGACGACTTCCCGACGGACCG
GTACCTGATCGAGCTGTCGGCGGCCAAGAGCGGCCGGAGCTACGACTACGAGTACGCCAC
GCTGCGGGACCTGATGCTGGACTGCAGCTACCGCGGCGGCGGCGTGGCGGTGGTGGACTC
GCTCCGCGTCGCCGTCGACAACTGCTACGTGGCGCACTTCGCGTCCGACGGCGTCGCGGT
GCGCGGCGGCCACGAGACGCTCATCCGCAACACCTACCTCGGCCAGCACATGACGGCCGG
GGGCGACCCGGGGGAGCGCGGCTTCACGGGCACGGCCATCCGCCTCGACGGCAACGACAA
CTCCGTGTCCGACGTCGTCATCTTCTCCGCGGCCACGGGGATCATGGTCACCGCGCCGGC
CAACTCCATCTCCGGCGTGCACTGCTACAACAAGGCCACGGGCTTCGGCGGCACCGGCAT
CCACCTCAAGATCCCGGGCCTCACGCAGGCGTGGATCAGCAACTGCTACATGGACTACAC
CAGCATCGTCGCCGAGGACCCCGTGCTGCTCCACGTCTCCGGATCCTTCTTCCTCGGCGA
CGCCAACGTCGTGCTCAAGGCCGTCAGCGGCGTCGCCAGGGGCGTCCAGGTCGTCGGCAA
CATCTTCAGCGGCCGGGACAAGGGCGTCGACATCGTGCAGCTGGACGGCGCCTTCGCCAC
CGTGGACCAGGTGTACGTGCAGCAGAACTCCGCCACGGGGATGACCGTCAGGTCCACCTC
CGCGCGCGCATCGCTCGAGGGCAACGGGACCTCCTGGACGCTCGACTTCTCGCCGGTGCT
GCTGTTCCCGGACCGCATCGGCCACGTGCAGTACTCGCTCGTCGCCGGCGACGAGTTCCC

GGGCCACACGCTCCGGAACGTGTCCGGGAACCAGGTCGTGGTCGCCACCGACAAGGCCGT
CTCGGCCACGGTCCACGTCTTGGTGGACCAGAACAGCGACTGAGATAAGATAAGAGACAC
TCGAGAGTGCCTGCGTGCACGCAGTATGCGTGCTGCTGCCTGCTCGCGCGGCTGCTGCGG
AGCAAGGCGTTATTCCTTCGTTTCTTTCTTCTCCAAAATTTCAGCCATTCATGTCGTCGG
GAAGATGACAATTGTTTGATGCGACAAAATGCTCCAGAATGGATCATTCTTTTGCTGTAT
GAAATGAAATGATTCAGAAGCTCC
