# Supplementary figures and images for: Genome-Wide Identification and Characterization of Polygalacturonase Gene Family in Maize (Zea mays L.)
Source: Int J Mol Sci. 2021 Oct 3;22(19):10722. doi: 10.3390/ijms221910722 (PMC8509529; doi:10.3390/ijms221910722)

Figure S2

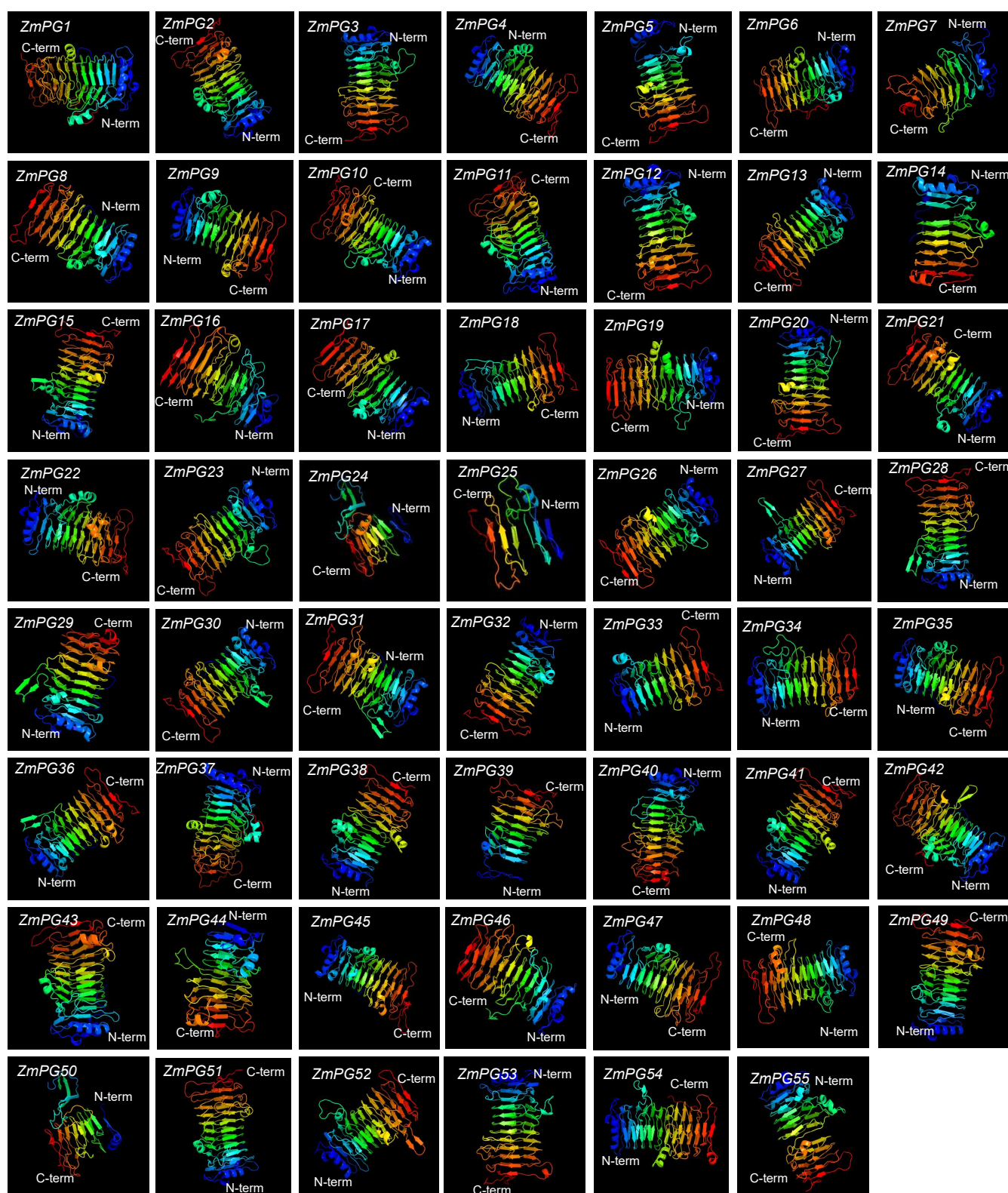

Figure S2. Predicted protein structures of 55 ZmPGs.

Supplement: Supplementary file 1 [file ijms-22-10722-s001.zip › Figure S2. Predicted protein structures of 55 ZmPGs.pdf]

Figure S3

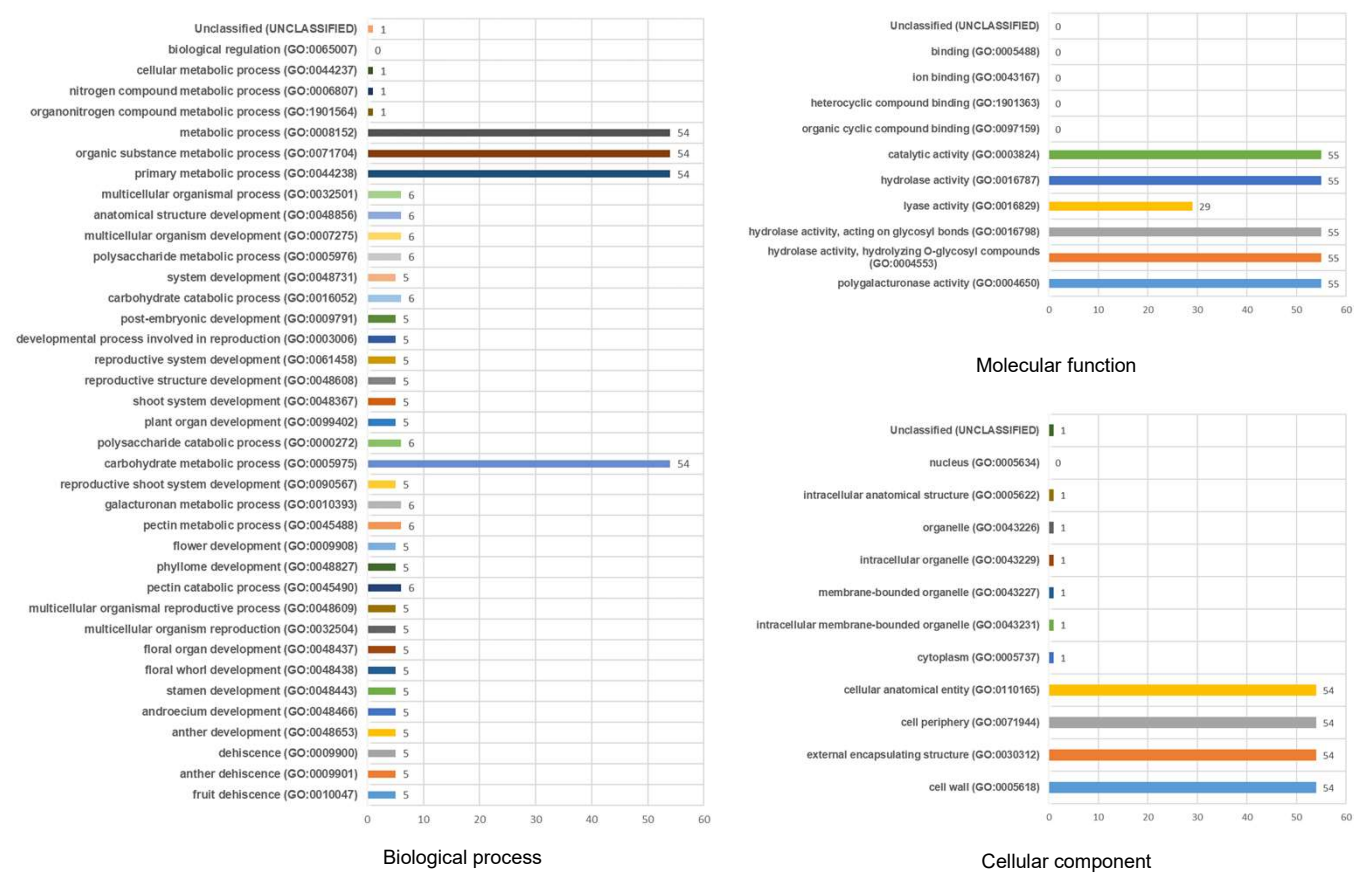

Figure S3. Gene ontology analysis of polygalacturonase (PG) gene family in Maize.

Supplement: Supplementary file 1 [file ijms-22-10722-s001.zip › Figure S3. Gene ontology analysis of polygalacturonase (PG) gene family in Maize.pdf]
